# Electrophilic Vinylation of Thiols under Mild and Transition Metal‐Free Conditions

**DOI:** 10.1002/anie.202002936

**Published:** 2020-06-15

**Authors:** Laura Castoldi, Ester Maria Di Tommaso, Marcus Reitti, Barbara Gräfen, Berit Olofsson

**Affiliations:** ^1^ Department of Organic Chemistry Arrhenius Laboratory Stockholm University 10691 Stockholm Sweden

**Keywords:** alkenyl sulfides, benziodoxolones, hypervalent compounds, synthetic methods, vinylbenziodoxolones

## Abstract

The iodine(III) reagents vinylbenziodoxolones (VBX) were employed to vinylate a series of aliphatic and aromatic thiols, providing *E*‐alkenyl sulfides with complete chemo‐ and regioselectivity, as well as excellent stereoselectivity. The methodology displays high functional group tolerance and proceeds under mild and transition metal‐free conditions without the need for excess substrate or reagents. Mercaptothiazoles could be vinylated under modified conditions, resulting in opposite stereoselectivity compared to previous reactions with vinyliodonium salts. Novel VBX reagents with substituted benziodoxolone cores were prepared, and improved reactivity was discovered with a dimethyl‐substituted core.

## Graphical abstract

**Novel vinylation reagent**: The iodine(III) reagents vinylbenziodoxolones (VBX) have been employed to vinylate aliphatic and aromatic thiols, as well as mercaptothiazoles. The methodology proceeds under mild and transition metal‐free conditions to provide alkenyl sulfides with excellent stereoselectivity. A novel VBX reagent with a substituted benziodoxolone core had superior efficiency.

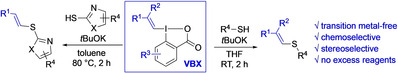

Hypervalent iodine compounds have emerged as sustainable alternatives to metal‐based oxidants and organometallic catalysts. Most iodine(III) reagents are nontoxic, easily synthesized, and reactive under mild conditions.^1^ Iodonium salts have a unique ability to form C−C and C‐heteroatom bonds through transfer of one carbon ligand to a variety of nucleophiles.^2^ Although vinyl(aryl)iodonium salts can be employed to vinylate nucleophiles,^3^ their reactivity is difficult to control under metal‐free conditions, often leading to product mixtures.^4^ Benziodoxolones have enhanced stability and more controllable reactivity compared to iodonium salts. This feature has been demonstrated by the Togni trifluoromethylation reagents and Waser's alkynylations using alkynylbenziodoxolones (EBX).^5^ While the corresponding vinylbenziodoxolones were reported as products from the addition of azide to EBX already in 1996,^6^ they have remained unexplored as synthetic reagents. In 2016, we reported a one‐pot synthesis of vinylbenziodoxolones from 2‐iodobenzoic acid and abbreviated these novel reagents VBX (Scheme 1 a).^7^ Their unique reactivity was demonstrated in the vinylation of nitrocyclohexane, with opposite regioselectivity to the corresponding vinyliodonium salt^8^ (Scheme 1 b).^7^ In parallel, Yoshikai and co‐workers developed the synthesis of β‐oxygen‐functionalized VBX reagents through Pd‐catalyzed hydrocarboxylation of EBX‐type reagents (Scheme 1 c).^9^ The scope of VBX has since increased further by addition of heteroatom nucleophiles to various iodine(III) precursors,^10^ and the reagent class has been employed in metal‐catalyzed cross couplings and C−H vinylations, as well as in metal‐free reactions.^9^, ^10^, ^11^

**Scheme 1:**
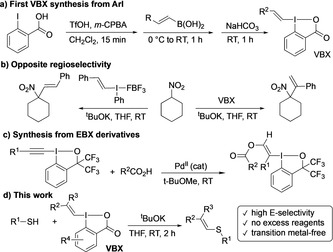
Preparation of vinylbenziodoxol(on)es and vinylations with VBX.

Vinyl sulfides are important building blocks in organic synthesis,^12^ natural products and biologically active compounds.^13^ Their reactivity is interesting since they can be considered as enolate equivalents^14^ and Michael acceptors.^15^ Most synthetic routes to vinyl sulfides involve the use of transition metals, such as Ru‐catalyzed hydrothiolation of terminal alkynes,^16^ and Cu‐catalyzed cross coupling reactions at elevated temperature.^17^ Whereas metal‐free additions to alkynes proceed under mild conditions, other synthetic routes require strong base, and often give diastereo‐ or regioisomeric mixtures.^18^ Ochiai and co‐workers reported a single vinylation of PhSNa with a phenyl(4‐*tert*‐butylcyclohexenyl)iodonium under mild conditions,^19^ which has not been further explored.

Intrigued by the different regiochemical outcome with VBX and vinyliodonium salts (Scheme 1 b), and inspired by Waser's EBX‐alkynylation of thiols,^20^ we have investigated the reactivity of VBX with thiols, and herein report our results. The reaction was found to proceed under mild and transition metal‐free conditions, and contrary to the vinylation of nitrocyclohexane, regiospecific formation of (*E*)‐1,2‐substituted vinyl sulfides was observed (Scheme 1 d). During the course of our investigation, three types of metal‐free S‐vinylations with VBX were reported, although only 1–2 examples were given in each case: vinylation of sulfenate anions to (*E*)‐alkenyl sulfoxides,11d thiophenol vinylation with a sulfonamide‐substituted VBX,10d and with a regular VBX using a large excess of thiophenol.11e

The vinylation of thiophenol (**1 a**) with VBX **2 a** was first attempted in THF with TMG as base,^20^ resulting in 68 % of the vinylated product **3 a** with disulfide **4 a** as byproduct (Table 1, entry 1). For atom efficiency reasons, equimolar conditions were maintained in the optimization to suppress the formation of **4 a**.^21^ Considerable amounts of **4 a** were obtained with various bases, as well as in the absence of base (entries 2–5). Reactions in THF with *t*BuOK with 2 h reaction time proved best. The *E*/*Z* ratio of **3 a** increased to >20:1 when VBX was added before the base (entry 6), and **4 a** was further suppressed in anhydrous and degassed solvent, delivering **3 a** in 87 % yield (entry 7). Vinylation of the corresponding TMS‐protected thiophenol **7** was feasible by in situ‐deprotection with TBAF prior to addition of **2 a** (entry 8).^21^ This strategy could be beneficial with base‐sensitive thiols.

**Table 1:** Optimization on thiophenol.^[a]^ 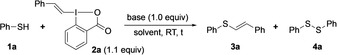

Entry	Solvent	Base	*t* [h]	Yield of **3 a** [%]^[b]^	*E*/*Z* ratio	Yield of **4 a** [%]^[b]^
1	THF	TMG	15	68	15:1	18
2	Toluene	TMG	15	53	>20:1	34
3	THF	–	15	54	20:1	30
4	THF	NaHCO_3_	15	36	9:1	30
5	THF	*t*BuOK	15	78	10:1	18
6	THF	*t*BuOK	2	76^[c]^	>20:1	13
7	THF	*t*BuOK	2	87^[c,d]^	>20:1	7
8^[e]^	THF	–	2	77^[d]^	>20:1	12

[a] Reaction conditions: **1 a** (0.3 mmol) and base were stirred in solvent for 5 min before addition of **2 a**. [b] ^1^H NMR yield using trimethoxybenzene as internal standard. [c] Addition of VBX, then base. [d] Anhydrous and degassed solvent. [e] PhS‐TMS (**7**) and TBAF (1.0 equiv) used instead of **1 a** and base. TMG=1,1,3,3‐tetramethylguanidine.

The reactivity of iodine(III) compounds can be influenced by *ortho*‐substituents,^22^ and EBX reagents with substituted benziodoxolone cores have been investigated.^23^ Hence, a series of novel, substituted VBX derivatives were synthesized using Nachtsheim's procedure.11a, 21 Electronic factors were investigated through **2 b**–**2 e** with *p*‐substituted benziodoxolone cores, and steric effects were screened with *o*‐substituted VBX **2 f, 2 g** (Table 2). The chemoselectivity was poor in reactions with nitro‐substituted reagents **2 b** and **2 f**, with preferential transfer of the aryl group to yield diaryl sulfide **5** (entries 2,6). The other reagents all delivered product **3 a** with complete chemoselectivity and *E*/*Z* ratios ranging from 11:1 (**2 c**) to >20:1. Me_2_‐VBX reagent **2 e** provided **3 a** in 90 % yield (entry 7), indicating that moderately electron‐donating substituents can be favorable in benziodoxolone chemistry. A control reaction with vinyliodonium salt **6** delivered **3 a** in poor yield with 1:1 *E*/*Z*‐ratio (entry 8).

**Table 2:** Influence of substituents on the benziodoxolone core.^[a]^ 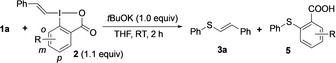

Entry	**2**	R	Yield of **3 a** [%]	*E*/*Z* ratio	Yield of **5** [%]
1	**2 a**	H	87	>20:1	0
2	**2 b**	*p*‐NO_2_	11	>20:1	40
3	**2 c**	*p*‐Br	67	11:1	0
4	**2 d**	*p*‐OMe	75	>20:1	0
5	**2 e**	*m*,*p*‐Me_2_	90	>20:1	0
6	**2 f**	*o*‐NO_2_	9	>20:1	18
7	**2 g**	*o*‐Me	68	>20:1	0
8	**6**	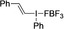	20	1:1	0

[a] Reaction conditions: see Table 1 entry 7; NMR yields given.

The scope of the reaction was examined with VBX reagent **2 a**, due to its considerably less expensive precursor than **2 e**. Thiophenols containing both electron‐donating and electron‐withdrawing substituents could be employed to provide products **3 a**–**k** in good yields, with excellent *E*‐stereoselectivity (Scheme 2 a). While sterically hindered thiophenols reacted sluggishly at RT, efficient vinylation to **3 g** was possible at 50 °C for 2 h.

**Scheme 2:**
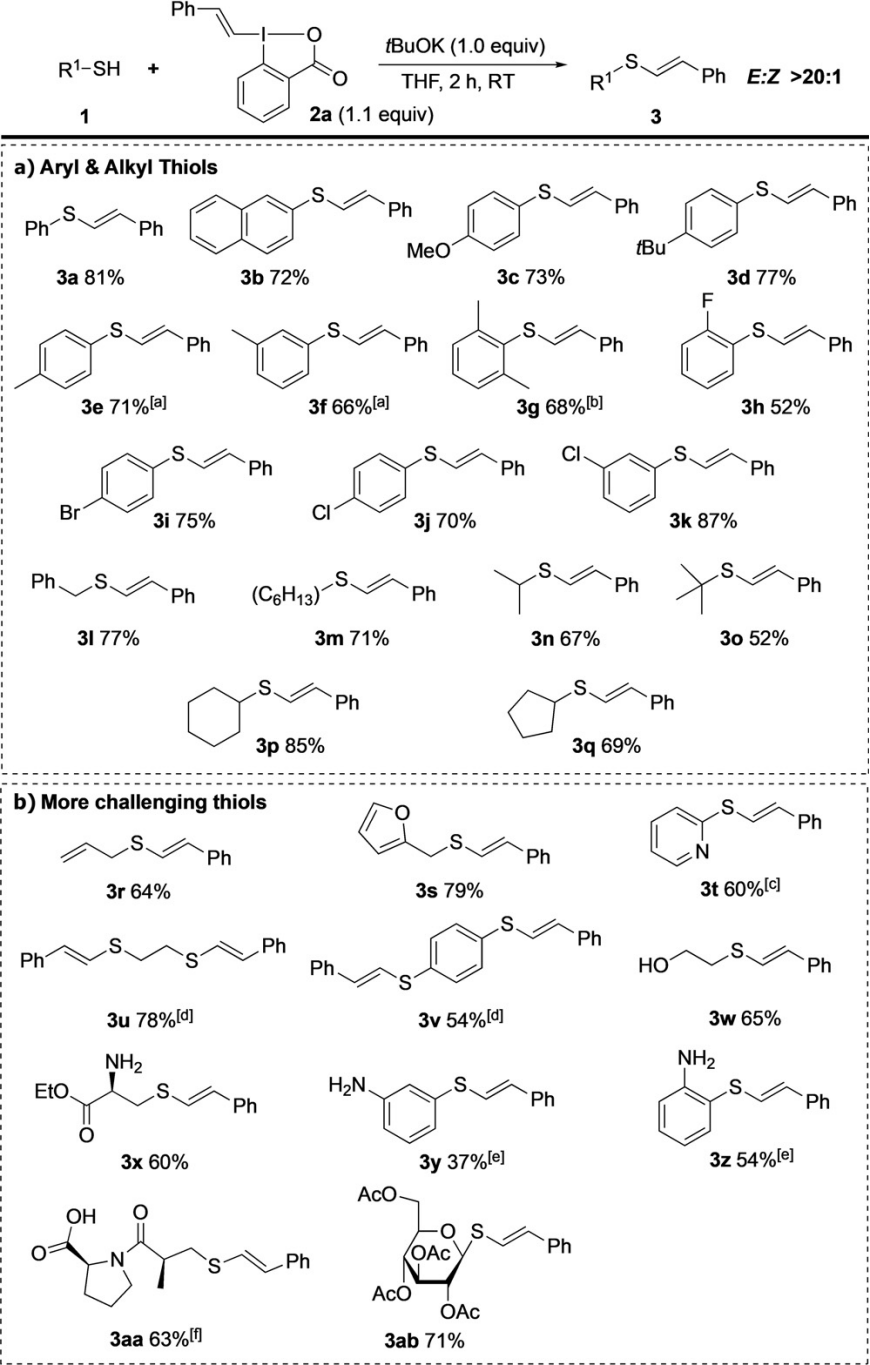
Scope of thiol vinylation with VBX, products were obtained with *E*/*Z*>20:1 unless specified. [a] *E*/*Z* 16:1 [b] At 50 °C. [c] *E*/*Z* 5:1 [d] With **2 a** (2.1 equiv) and base (2.0 equiv). [e] With **2 a** (1.5 equiv) at 50 °C. [f] With 2.0 equiv base.

Halide substituents were well tolerated, also in the *ortho* position (**3 h**–**3 k**). Both linear and cyclic aliphatic thiols could be vinylated at rt to provide (*E*)‐thioethers **3 l**–**3 q** with complete *E*‐selectivity, even with sterically demanding substituents (**3 o**).

More challenging substrates were subsequently examined to evaluate the functional group tolerance (Scheme 2 b). Allyl, furanyl, and pyridyl substituents were well tolerated, providing **3 r**–**3 t**, and double vinylation to products **3 u**,**3 v** could be achieved. The S‐vinylation proceeded with complete chemoselectivity in the presence of unprotected hydroxy‐ and amino groups, as demonstrated by the vinylation of 2‐mercaptoethan‐1‐ol and cysteine ethyl ester to give products **3 w** and **3 x** with complete *E*‐selectivity. Under slightly modified conditions, also the S‐vinylation of amino thiophenols to provide products **3 y** and **3 z** was achieved. The high functional group tolerance was further demonstrated by late stage functionalization of the ACE inhibitor Captopril,^24^ which could be vinylated without protection of the carboxylic acid moiety to provide **3 aa**. Moreover, the carbohydrate thio‐β‐d‐glucose tetraacetate was vinylated in good yield (**3 ab**).^25^ Vinylations of cysteine and thio‐β‐d‐glucose to provide the unprotected derivatives of **3 x** and **3 ab** were low‐yielding, likely due to solubility problems.^21^

A set of substituted VBX reagents was synthesized to demonstrate the feasibility to transfer other vinyl groups (Scheme 3). Indeed, reactions with *E*‐VBX reagents **2 h**–**2 l**, having different electronic properties, resulted in thioethers **3 ac**–**3 ag** in good yields. High *E*‐selectivities were obtained in all cases except **3 ae**. Vinylations with cyclohexyl‐substituted VBX **2 m** proved less reactive and gave a modest yield.^21^ Attempts to synthesize the Z‐stereoisomer of **2 a** were in vain due to isomerization to *E*‐**2 a** under the reaction conditions.^26^

**Scheme 3:**
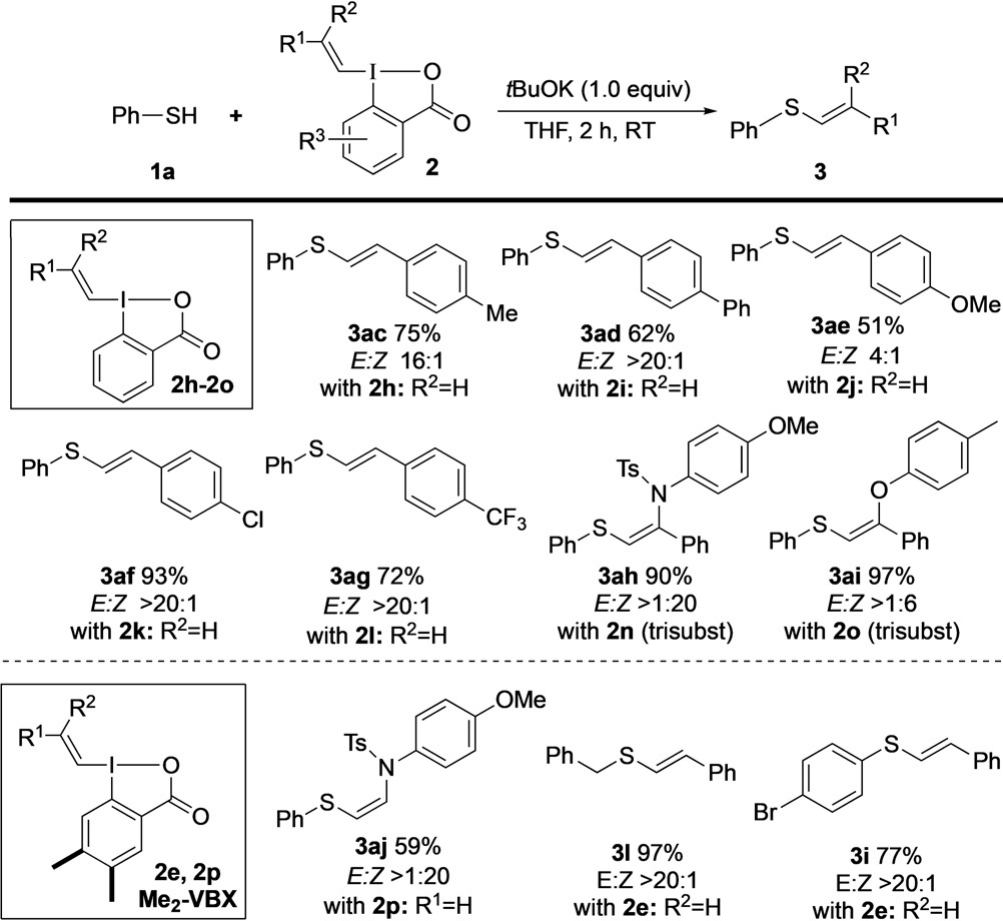
Scope with substituted VBX reagents.

Waser and co‐workers recently reported a vinylation of thiophenol with a *Z*‐configured sulfonamide‐substituted VBX to provide a thioenamide with moderate *Z*‐selectivity.10d Considering the excellent stereoselectivity of our methodology, we were intrigued to investigate the reactivity of such reagents under our conditions. Indeed, trisubstituted thioenamide **3 ah** and thioenol ether **3 ai** were obtained in excellent yields with good to complete Z‐selectivity.^27^ However, the corresponding disubstituted thioenamide **3 aj** only formed in modest yield with 1,2‐bis(phenylthio)ethene11e as the main byproduct, and attempts to optimize the reaction conditions were in vain. Pleasingly, the corresponding Me_2_‐substitued VBX reagent **2 p** (*cf* **2 e** in Table 2) proved more efficient, delivering thioenamide **3 aj** in 59 % yield with complete Z‐selectivity and suppressed byproduct formation.

Me_2_‐VBX reagent **2 e** was thus investigated in selected *E*‐selective vinylations as alternative to **2 a**, and indeed provided product **3 l** in increased yield (97 vs. 77 %). While vinyl sulfide **3 i** formed in similar yields with **2 a** and **2 e**, reactions with Me_2_‐VBX are more convenient as column chromatography is not needed. We are currently investigating the Me_2_‐VBX backbone in other transformations, and will report the results in due time. The formed iodobenzoic acid can be recovered and reused in formation of VBX, thus increasing the sustainability and economy of the process.^21^

Ochiai and co‐workers have demonstrated that metal‐free vinylation of various nucleophiles with *E*‐alkylvinyl(phenyl)iodonium salts result in *Z*‐vinylated products through a vinylic S_N_2 mechanism.3a In this fashion, vinylation of mercaptobenzothiazole in the absence of base resulted in selective formation of the corresponding *Z*‐vinylsulfide.3a To compare the reactivity of VBX with vinyliodonium salts, the vinylation of a small series of mercaptothiazoles **8** (X=S) was investigated. This substrate class could indeed be vinylated in moderate yields and high stereoselectivity (*E*/*Z* 10:1 to 20:1) under modified reaction conditions (Scheme 4).^21^ Interestingly, we observed opposite stereochemistry compared to previous results with the vinyliodonium salt. The methodology was also applied to mercaptooxazole (X=O) to give **9 d**.

**Scheme 4:**
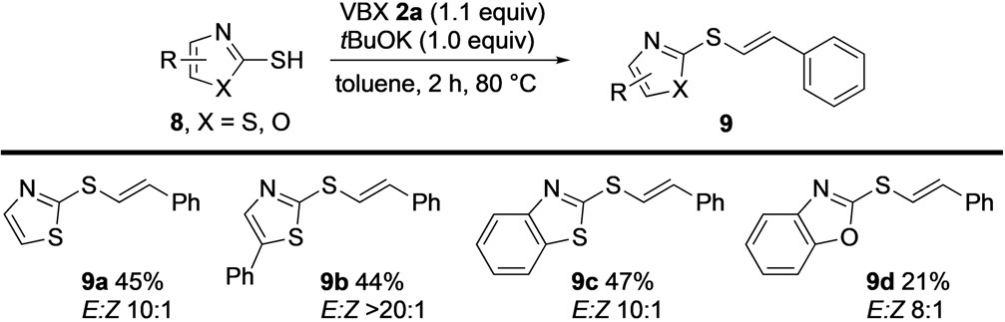
*S*‐Vinylation of heterocycles with VBX.

The observed regioselectivity of the *S*‐vinylation is intriguing, as the *C*‐vinylation of nitrocyclohexane with VBX **2 a** gave a terminal alkene as the main product (see Scheme 1 b).^7^ Furthermore, the high *E*‐stereoselectivity is opposite to reactions with vinyliodonium salts and shows that VBX does not react through a vinylic S_N_2 mechanism.3a While preliminary radical trap experiments were inconclusive,^21^ isomerization of **3 e** was observed upon purification on column chromatography (from *E*/*Z*>20:1 to 16:1), and we hence propose that the main reaction pathway gives the *E*‐product, while the *Z*‐product is formed by isomerization. We are currently investigating the mechanisms of VBX vinylations with various nucleophiles by DFT calculations and ^13^C‐labelling studies to detect any carbene pathways, and will report the results in due time.

To conclude, we have reported a high‐yielding method for vinylation of aromatic and aliphatic thiols with the recently discovered hypervalent iodine(III) reagents VBX. This transition metal‐free methodology uses equimolar amounts of reagents and proceeds under mild conditions with complete chemo‐ and regioselectivity, as well as high stereoselectivity. Mercaptoheterocycles could be vinylated under modified conditions. Moreover, the synthesis and reactivity of several novel, substituted VBX reagents was described to illustrate the influences of steric and electronic factors on the vinylation. The Me_2_‐VBX backbone proved superior to the parent VBX, a discovery that could have impact on reactions with other benziodoxolone reagents too, such as alkynylations and trifluoromethylations. Results from our ongoing mechanistic studies of metal‐free vinylations with VBX and various nucleophiles will be reported in due time.

## Experimental Section

***General Procedure for Vinylation of Thiols***: Thiol **1** (1.0 equiv, 0.3 mmol) was placed in an oven‐dried microwave vial with magnetic stirring bar under argon, followed by the addition of anhydrous and degassed THF (2.0 mL). Subsequently, VBX **2** (1.1 equiv) and *t*BuOK (1.0 equiv) were sequentially added and the vial was rinsed with THF (1.0 mL). The mixture rapidly turns yellow and it was stirred at RT for 2 h. The reaction was quenched with water (2.0 mL) and the aqueous phase was extracted with CH_2_Cl_2_ (2×10 mL) and the combined organic phases were dried over Na_2_SO_4_, filtered and concentrated under reduce pressure. The crude reaction was purified via column chromatography to provide product **3**.

## Conflict of interest

The authors declare no conflict of interest.

## Supporting information

As a service to our authors and readers, this journal provides supporting information supplied by the authors. Such materials are peer reviewed and may be re‐organized for online delivery, but are not copy‐edited or typeset. Technical support issues arising from supporting information (other than missing files) should be addressed to the authors.

Supplementary
