# Supplementary material for: Electrophilic Vinylation of Thiols under Mild and Transition Metal‐Free Conditions
Source: Angew Chem Int Ed Engl. 2020 Jun 15;59(36):15512–6. doi: 10.1002/anie.202002936 (PMC7497129; doi:10.1002/anie.202002936)
Supplement: Supplementary file 1 — Supplementary [file ANIE-59-15512-s001.pdf]

Angewandte Chemie
Eine Zeitschrift der Gesellschaft Deutscher Chemiker

Supporting Information

Electrophilic Vinylation of Thiols under Mild and Transition Metal-Free Conditions

*Laura Castoldi, Ester Maria Di Tommaso, Marcus Reitti, Barbara Gräfen, and Berit Olofsson**

anie_202002936_sm_miscellaneous_information.pdf

Author Contributions

L.C. Methodology: Lead; Writing—Original Draft: Equal
E.D. Methodology: Supporting
M.R. Supervision: Supporting
B.G. Methodology: Supporting
B.O. Funding acquisition: Lead; Project administration: Lead; Supervision: Lead; Writing—Original Draft: Equal; Writing—Review & Editing: Lead.

Table of Contents

1. General Experimental Procedures 2

2. Synthesis of Iodine(III) reagents 2

2.1 One-pot synthesis of VBX reagents 4

2.2 Step-wise synthesis of Core-Substituted VBX 5

2.3 Synthesis of the novel Me_2-VBX derivatives 6

3 Investigation on S-Vinylation 8

3.1 Optimization on Thiols 8

3.2 Mechanistic studies 9

3.3 Reaction of silyl protected thiophenol 10

3.4 Optimization on Mercaptothiazoles and byproduct formation 10

4 Analytical Data 13

4.1 Products from Vinylation of Thiols 13

4.2 Attempted vinylation of unprotected complex structures 20

4.3 Products from Vinylation of Mercaptothiazoles 20

5 References 21

1. General Experimental Procedures

Chemicals

mCPBA (<77 wt.%) was purchased from Sigma-Aldrich and dried under high vacuum at rt for three hours. The weight percent active oxidant was then determined by iodometric titration[1] and varied between 84-88% in different batches. 2-iodobenzoic acid was purchased from Sigma-Aldrich and used as received.

Trifluoromethanesulfonic acid (TfOH) and trimethylsilyl trifluoromethanesulfonate (TMSOTf) were purchased from TCI, stored under argon and handled using dry glass Hamilton syringes with oven-dried metal needles. $BF_3.OEt_2$ was purchased from Sigma-Aldrich, stored under argon and handled using dry glass Hamilton syringes with oven-dried metal needles.

The purity of the boronic acids is of high importance for reproducible yields and the quality was determined by ^{1}H-NMR with internal standard. All the boronic acids used were purchased from Sigma-Aldrich and were used without any purification.

Thiols were purchased from different suppliers (Sigma -Aldrich, Fluorochem, TCI) and purity was determined by ^{1}H-NMR. In some cases, due to the presence of >10% of the corresponding disulphide as impurity, it was needed to purify by distillation/column chromatography and, for solids, recrystallization. Nevertheless, we could not observe reactivity of disulphides at our reaction conditions.

Solvents

DCM, Et_2O and MeCN were used as received. Toluene and THF were dried using a VAC-purification system and degassed for 30 minutes by bubbling argon through a long needle prior to all reactions.

Procedures and Analysis

TLC analysis were performed on pre-coated silica gel 60 F254 plates using either UV light. The crude products were purified by flash column chromatography using 40-60 µm 60A silica gel as stationary phase or using automated flash system Teledyne ISCO CombiFlash Rf 200 with RediSep Rf columns.

Melting points were measured using a STUART SMP3 and are reported uncorrected. The melting point measurements refer to the solidified materials as the result of the given experimental procedures, no additional recrystallization was done.

Chiral SFC was performed using Chiralpak IF column (3 mm × 150 mm × 3 µm) eluting with MeOH/CO_2 and monitored by DAD (Diode Array Detector). Retentions times (tR) are quoted in minutes.

NMR spectra were recorded using a 400 MHz Bruker AVANCE II with a BBO probe at 298 K using $CDCl_3$, MeOD-d_4 or DMSO-d_6 as solvent. Chemical shifts are given in ppm relative to the residual solvent peak (^{1}H NMR: $CDCl_3$ δ 7.26; MeOD-d_4 δ 3.31; DMSO-d_6 δ 2.50; ^{13}C NMR: $CDCl_3$ δ 77.16; MeOD-d_4 δ 49.00; DMSO-d_6 δ 39.52) with multiplicity (br = broad, s = singlet, d = doublet, t = triplet, m = multiplet, app = apparent), coupling constants (in Hz) and integration. Full analytical data is given for compounds that are novel or not fully characterized in the literature; ^{1}H NMR and ^{13}C NMR are given for literature reported compounds.

High-resolution mass analyses were obtained using a Bruker microTOF ESI.

2. Synthesis of Iodine(III) reagents

The synthesized iodine(III) reagents are shown in Figure S1.

The synthesis of VBX **2a** and **2g-2m** were obtained using our reported procedure (Section 2.1).[2] The core-substituted VBX **2b-2f** and **2n** were synthetized using Nachtsheim's two-step procedure[3] (Section 2.2) since our one-pot procedure is optimized for 2-iodobenzoic acid and yields of vinylbenziodoxolones with substituted iodobenzoic acid varied considerably.

The vinyl salt **6** was synthetized according to the reported procedure[3]. The open salt was used to compare its reactivity with the VBX in terms of both regio- and stereo-selectivity.

The novel compound **2n, 2o and 2p** were obtained using a modified procedure[4] and a full description is given (*vide infra*).

EBX derivatives **12** and **13** has been prepared following literature procedures[4] and a full description is given (*vide infra*).

a) VBX derivatives

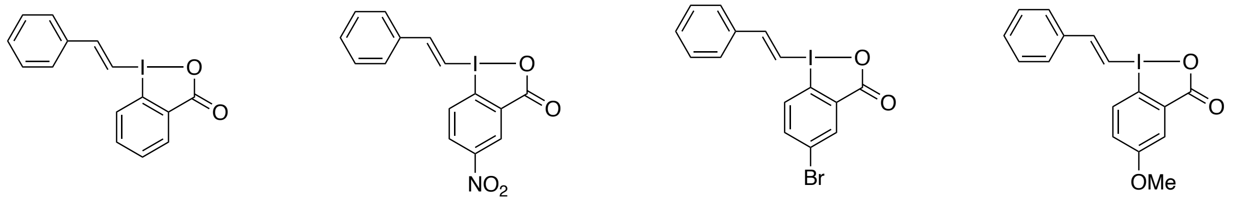

2a **2b** **2c** **2d**

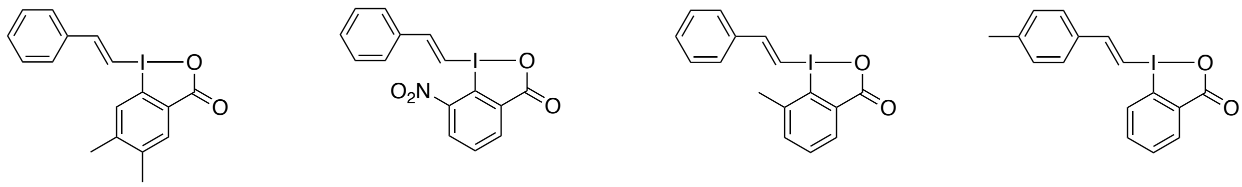

2e **2f** **2g** **2h**

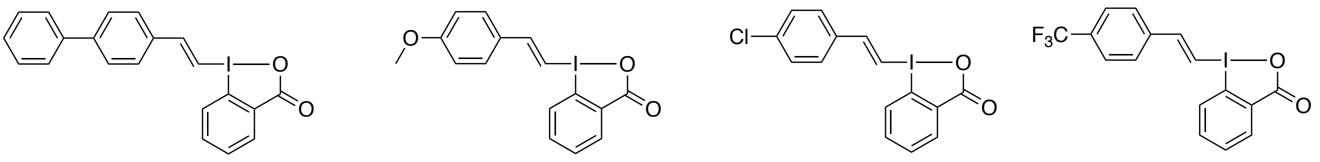

2i **2j** **2k** **2l**

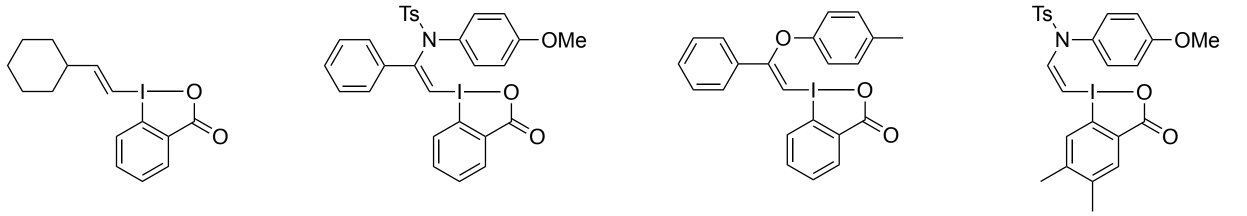

2m **2n** **2o** **2p**

b) Iodosyl derivatives

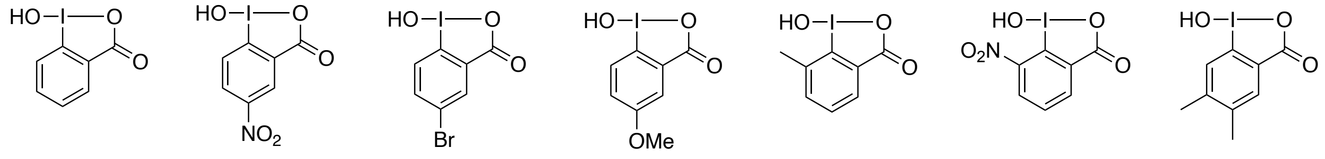

11a **11b** **11c** **11d** **11e** **11f** **11g**

c) EBX derivatives

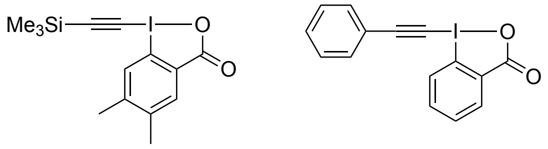

12 **13**

d) Open salt

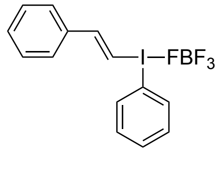

6

Figure S1. VBX reagents, iodosyl and TMS-EBX derivatives.

2.1 One-pot synthesis of VBX reagents

VBX reagents with unsubstituted benziodoxolone core were prepared by employing our reported one-pot synthesis.[2]

General procedure S1:

2-iodobenzoic acid (1 equiv) was added to a round bottom flask followed by DCM. *m*CPBA (85%, 1.1 equiv) was added, and the mixture was cooled to 0 °C followed by the addition of TfOH (1.5 equiv). The mixture was stirred at RT for 15 minutes and then cooled to 0 °C for 5 minutes. The corresponding boronic acid (1.4 equiv) was added in one portion and rinsed down with DCM (1-5 mL). The mixture was stirred at room temperature for 1 h. Saturated $NaHCO_3$ was added and the mixture was stirred vigorously at rt for 1 h. The reaction mixture was transferred to a separation funnel, diluted with DCM and H_2O. *Note: dilution helped to avoid emulsions in the separation.* The layers were separated the aqueous phase was extracted three times with DCM. The combined organic phases were washed with H_2O and brine and then dried over Na_2SO_4. The drying agent was filtered off and the solvent was removed *in vacuo.* Et_2O was added to the white precipitate and the mixture was stirred vigorously at RT for approx. 30 min. The solid was filtered off (glass filter funnel, porosity 3) and washed with Et_2O to obtain VBX reagents.

(*E*)-1-Styryl-1λ³-benzo[*d*][1,2]iodaoxol-3(1*H*)-one (2a)

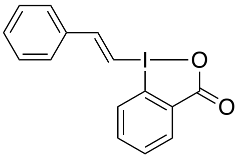

Prepared according to General procedure S1[2] (1255 mg, 3.6 mmol, 72%). ^{1}H-NMR (400 MHz, MeOD-*d₄*): δ 8.31-8.22 (m, 1H), 7.96 (d, *J* = 15.5 Hz, 1H), 7.76-7.61 (m, 6H), 7.55-7.42 (m, 3H). ^{13}C-NMR (101 MHz, MeOD-d_4): δ 170.1, 155.8, 136.7, 135.3, 134.5, 133.3, 132.1, 131.8, 130.2, 129.0, 129.0, 115.5, 100.0.

(*E*)-1-(4-methylstyryl)-1,2-benziodoxol-3-(1*H*)-one (2h)

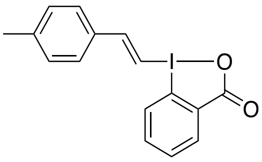

Prepared according to General procedure S1[2] (109.2 mg, 0.30 mmol, 76%). ^{1}H-NMR (400 MHz, MeOD-d_4): δ 8.32-8.22 (m, 1H), 7.91 (d, *J* = 15.5 Hz, 1H), 7.78-7.63 (m, 3H), 7.63-7.51 (m, 3H), 7.31 (d, *J* = 7.9 Hz, 2H), 2.41 (s, 3H). ^{13}C-NMR (101 MHz, MeOD-d_4): δ 170.1, 155.9, 143.0, 135.2, 134.5, 134.0, 133.3, 131.8, 130.8, 129.0, 128.9, 115.5, 98.4, 21.5.

(*E*)-1-(2-([1,1'-biphenyl]-4-yl)vinyl)-1λ³-benzo[*d*][1,2]iodaoxol-3(1*H*)-one (2i)

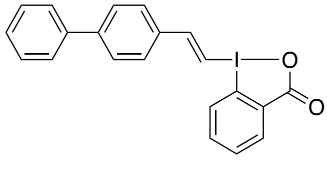

Prepared according to General procedure S1[2] (109 mg, 0.25 mmol, 64%). Mp: 186-187° C. ^{1}H NMR (400 MHz, DMSO-d_6) δ 8.20 – 8.11 (m, 1H), 7.98 (d, *J* = 15.6 Hz, 1H), 7.88 (d, *J* = 15.6 Hz, 1H), 7.81 (s, 4H), 7.78 – 7.72 (m, 2H), 7.71 – 7.60 (m, 3H), 7.50 (t, *J* = 7.6 Hz, 2H), 7.45 – 7.37 (m, 1H). ^{13}C NMR (101 MHz, DMSO-d_6) δ 165.6, 151.4, 142.0, 139.2, 134.6, 134.5, 133.4, 131.4, 130.3, 129.1, 128.3, 128.1, 127.7, 127.2, 126.8, 115.1, 103.1. HRMS(ESI) m/z: calcd for $C_{21}H_{15}IO_2Na^+$ [M+Na]$^+$ 449.0011; found 449.0009.

(*E*)-1-(4-methoxystyryl)- 1λ³-benzo[d][1,2]iodaoxol-3(1*H*)-one (2j)

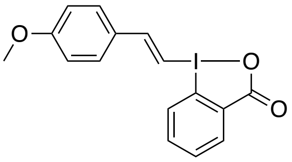

Prepared according to General procedure S1[2] (99 mg, 0.26 mmol, 65%). ^{1}H-NMR (400 MHz, MeOD-*d4*): δ 8.31-8.23 (m, 1H), 7.88 (d, *J* = 15.4 Hz, 1H), 7.76-7.59 (m, 5H), 7.44 (d, *J* = 15.4 Hz, 1H), 7.03 (d, *J* = 8.8 Hz, 2H), 3.86 (s, 3H). ^{13}C-NMR (101 MHz, MeOD-*d4*) δ 170.1, 163.6, 155.8, 135.2, 134.5, 133.3, 131.8, 130.8, 129.4, 128.8, 115.6, 115.5, 96.0, 56.0.

(*E*)-1-(4-(trifluoromethyl)styryl)-1,2-benziodoxol-3-(1*H*)-one (2k)

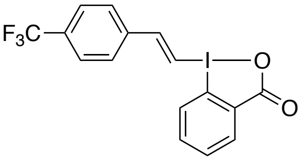

Prepared according to General procedure S1[2] (103.1 mg, 0.25 mmol, 64%). ^{1}H-NMR (400 MHz, MeOD-d_4): δ 8.32-8.24 (m, 1H), 8.03 (d, *J* = 15.6 Hz, 1H), 7.93-7.66 (m, 8H). ^{13}C-NMR (101 MHz, MeOD-d_4) δ 170.0, 153.5, 140.27 (d, *J* = 1.0 Hz), 135.4, 134.4, 133.3, 133.2 (q, *J* = 32.7 Hz), 131.9, 129.5, 129.1, 127.1 (q, *J* = 3.9 Hz), 125.3 (q, *J* = 272.4 Hz), 115.5, 104.0. ^{19}F-NMR (377 MHz, MeOD-*d4*): δ - 64.4.

(*E*)-1-(4-chlorostyryl)-1λ^3-benzo[*d*][1,2]iodaoxol-3(1*H*)-one (2l)

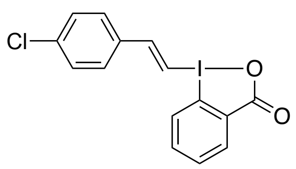

Prepared according to General procedure S1[2] (433 mg, 1.13 mmol, 56%). Mp: 173-174° C. ^{1}H-NMR (400 MHz, MeOD-*d4*): δ 8.33- 8.25 (m, 1H), 7.95 (d, *J* = 15.5 Hz, 1H), 7.71 (dq, *J* = 9.6, 5.8, 4.7 Hz, 6H), 7.55 – 7.49 (m, 2H). ^{13}C-NMR (101 MHz, MeOD-*d4*): δ 170.0, 154.1, 138.0, 135.4, 135.3, 134.5, 133.3, 131.9, 130.4, 130.4, 129.9, 129.0, 115.5, 101.0. HRMS(ESI) m/z: calcd for $C_{15}H_{10}ClIO_2Na^+$ [M+Na]$^+$ 406.9306; found 406.9335

(*E*)-1-(2-cyclohexylvinyl)- 1,2-benziodoxol-3-(1*H*)-one (2m)

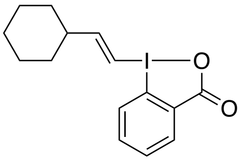

Prepared according to General procedure S1[2] (41.3 mg, 0.12 mmol, 27%). ^{1}H-NMR (400 MHz, MeOD-*d4*): δ 8.31-8.23 (m, 1H), 7.88 (d, *J* = 15.4 Hz, 1H), 7.76-7.59 (m, 5H), 7.44 (d, *J* = 15.4 Hz, 1H), 7.03 (d, *J* = 8.8 Hz, 2H), 3.86 (s, 3H). ^{13}C-NMR (101 MHz, MeOD-*d4*): δ 170.1, 163.6, 155.8, 135.2, 134.5, 133.3, 131.8, 130.8, 129.4, 128.8, 115.6, 115.5, 96.0, 56.0.

2.2 Step-wise synthesis of Core-Substituted VBX

The core-substituted VBX 2b-2f and 2l were synthetized using Nachtsheim's two-step procedure[3] since our one-pot procedure is optimized for 2-iodobenzoic acid and yields of vinylbenziodoxolones with substituted iodobenzoic acid varied considerably.

General procedure S2:[3]

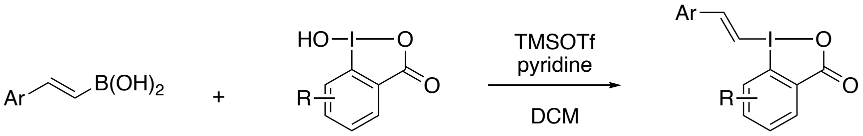

To a suspension of 2-iodosylbenzoic acid **11** (0.6-2.0 mmol, 1 equiv) in dry DCM or MeCN (10-20 mL) was added TMSOTf (0.67-2.30 mmol, 1.15 equiv) dropwise over 10 min and stirred for 30 min at room temperature. Afterwards (*E*)-styrylboronic acid (0.67-2.30 mmol, 1.15 equiv) was added over 5 min and the reaction mixture was stirred for 1.5 h at room temperature. Pyridine (1.15 equiv) was added and after further 10 min stirring the solvent was removed under reduced pressure. The residue was dissolved in DCM and washed with a solution of HCl 1M. The aqueous phase was extracted three times with DCM and the combined organic phases were washed with a saturated solution of $NaHCO_3$, dried over Na_2SO_4, filtered and concentrated under reduce pressure. The residue was dissolved in a minimum amount of DCM and precipitate in Et_2O, stirred vigorously for 30 min and stored at 4 °C for 2-16 h. The precipitate was filtered and washed with Et_2O to afford the corresponding core-substituted VBX reagent.
The iodosyl compounds required for this procedure were prepared following literature procedures: **11a**,[3] **11b**,[5] **11c**,[6] **11d**,[7] **11e**,[5] **11f**.[8]

(*E*)-5-nitro-1-styryl-1λ^3-benzo[*d*][1,2]iodaoxol-3(1*H*)-one (2b)

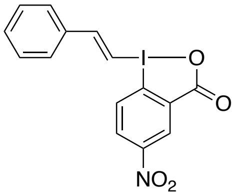

Prepared according to General procedure S2,[3] (241 mg, 0.61 mmol, 47 %). Mp: 189-191 °C. ^{1}H NMR (400 MHz, DMSO-*d$_6$*) δ 8.71 (d, *J* = 2.8 Hz, 1H), 8.44 (dd, *J* = 8.8, 2.8 Hz, 1H), 8.05 – 7.88 (m, 3H), 7.75 – 7.69 (m, 2H), 7.51 (qd, *J* = 7.6, 6.4, 3.6 Hz, 4H). ^{13}C NMR (101 MHz, DMSO-*d$_6$*) δ 163.8, 152.7, 149.5, 136.5, 135.3, 130.7, 129.9, 129.1, 127.8, 127.2, 124.9, 121.7, 103.1. HRMS(ESI) m/z: calcd for $C_{15}H_{10}INaNO_4^+$ [M+Na]$^+$ 417.9547; found 417.9517.*N.B.: the reaction was done in MeCN.*

(*E*)-5-bromo-1-styryl-1λ^3-benzo[*d*][1,2]iodaoxol-3(1*H*)-one (2c)

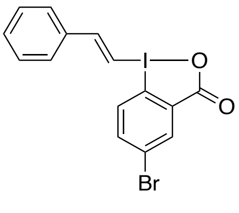

Prepared according to General procedure S2,[3] (625 mg, 1.46 mmol, 73 %). Mp: 111-112 °C. ^{1}H NMR (400 MHz, DMSO-*d$_6$*) δ 9.02 (d, *J* = 2.4 Hz, 1 H), 8.79 (d, *J* = 15.6 Hz), 8.72-8.65 (m, 2H), 8.56-8.50 (m, 2H), 8.40 (d, *J* = 8.6 Hz, 1H), 8.37-8.28 (m, 3H); ^{13}C NMR (400 MHz, DMSO-*d$_6$*) δ 164.1, 152.2, 136.7, 135.7, 135.4, 133.6, 130.6, 129.9, 129.0, 127.7, 124.2, 113.7, 102.7. HRMS(ESI) m/z: calcd for $C_{15}H_{10}BrINaO_2^+$ [M+Na]$^+$ 450.8801; found 450.8818.

(*E*)-5-methoxy-1-styryl-1λ^3-benzo[*d*][1,2]iodaoxol-3(1*H*)-one (2d)

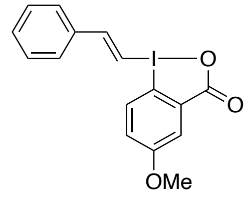

Prepared according to General procedure S2,[3] (330 mg, 0.870 mmol, 60 %). Mp: 122-124 °C. ^{1}H NMR (400 MHz, MeOD-*d4*) δ 7.93 (d, *J*=15.5 Hz, 1H), 7.77 (d, *J* = 3.0 Hz, 1H), 7.72-7.67 (m, 3H), 7.62 (d, *J* = 15.5 Hz, 1H), 7.53-7.45 (m, 3H), 7.20 (dd, *J* = 9.0, 3.0 Hz, 1H), 3.85(s, 3H);^{13}C NMR (400 MHz, MeOD-*d4*) 169.8, 163.6, 155.5, 136.7, 135.9, 132.1, 130.2, 129.6, 129.0, 121.6, 117.8, 103.6, 99.6, 56.4. HRMS(ESI) m/z: calcd for $C_{16}H_{13}INaO_3^+$ [M+Na]$^+$ 402.9802; found 402.9789.

(*E*)-7-nitro-1-styryl-1λ^3-benzo[*d*][1,2]iodaoxol-3(1*H*)-one (2f)

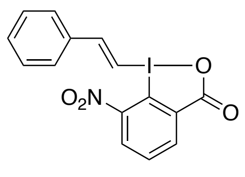

Prepared according to General proce S2,[3] (74 mg, 0.19 mmol, 32 %). Mp: 190-192 °C.^{1}H NMR (400 MHz, MeOD-*d4*) δ 8.44 (dd, *J* = 7.6, 1.6 Hz, 1H), 8.10 (dd, *J* = 7.8, 1.6 Hz, 1H), 7.88-7.78 (m, 2H), 7.52-7.39 (m, 6H);^{13}C NMR (400 MHz, MeOD-*d4*) δ 168.6, 153.7, 152.0, 140.8, 136.2, 136.1, 133.2, 132.1, 130.9, 130.0, 128.8, 108.7, 102.1. HRMS(ESI) m/z: (M+H) calcd for $C_{15}H_{10}INaNO_4^+$ [M+Na]$^+$ 417.9547; found 417.9554. *N.B.: the reaction was done in MeCN.*

(*E*)-7-methyl-1-styryl-1λ^3-benzo[*d*][1,2]iodaoxol-3(1*H*)-one (2g)

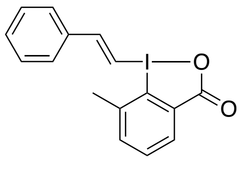

Prepared according to General procedure S2,[3] (202 mg, 0.55 mmol, 49 %). Mp: 115-117 °C. ^{1}H NMR (400 MHz, MeOD-*d4*) δ 7.99 (dd, *J* = 6.9, 2.4 Hz, 1H), 7.90 (q, *J* = 15.1 Hz, 2H), 7.61-7.49 (m, 4H), 7.43 (dt, *J* = 5.4, 3.0 Hz, 3H), 2.59 (s, 3H); ^{13}C NMR (400 MHz, MeOD-*d4*) δ 171.2, 151.5, 142.0, 139.8, 136.9, 136.5, 132.0, 131.9, 130.7, 130.2, 128.7, 119.0, 102.9, 25.7. HRMS(ESI) m/z: calcd for $C_{16}H_{13}INaO_2^+$ [M+Na]$^+$ 386.9852; found 386.9780.

2.3 Synthesis of the novel Me_2-VBX derivatives

5,6-dimethyl-1-((trimethylsilyl)ethynyl)- 1λ^3-benzo[*d*][1,2]iodaoxol-3(1*H*)-one (12)

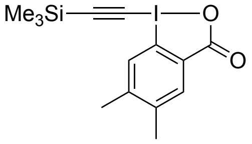

Following a modified procedure,[4] (335 mg, 0.9 mmol, 90%). Mp: 175-176 °C.^{1}H NMR (400 MHz, $CDCl_3$) δ 8.17 (s, 1H), 7.90 (s, 1H), 2.44 (d, *J* = 9.2 Hz, 6H), 0.35 (s, 9H).^{13}C NMR (101 MHz, $CDCl_3$) δ 167.1, 145.1, 141.2, 133.3, 129.0, 126.5, 116.6, 112.0, 20.7, 19.5, -0.2. HRMS(ESI) m/z: (M+H) calcd for $C_{14}H_{17}IO_2SiNa^+$ [M+Na]$^+$ 394.9935; found 394.9932.

1-Hydroxy-4,5-methyl-1λ^3-benzo[*d*][1,2]iodaoxol-3(1*H*)-one (11g)

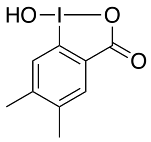

Following a modified procedure,[3] $NaIO_4$ (449 mg, 2.10 mmol, 1.05 equiv) and 4,5-methyl-2-iodobenzoic acid (552 mg, 2.0 mmol, 1 equiv) were suspended in 30% (v:v) aq. AcOH (4 mL). The mixture was vigorously stirred and refluxed for 4 h. The reaction mixture was then diluted with cold water (15 mL) and allowed to cool to RT, protecting it from light. The crude product was collected by filtration, washed on the filter with ice water (3 x 10 mL) and acetone (3 x 10 mL), and air-dried in the dark to give **11g** (549 mg, 1.88 mmol, 94%). Mp: 256-257 °C. ^{1}H NMR (400 MHz, DMSO-*d*$_6$) δ 7.91 (s, 1H), 7.79 (s, 1H), 7.55 (s, 1H), 2.38 (d, J = 5.4 Hz, 6H).^{13}C NMR (101 MHz, DMSO-*d*$_6$) δ 167.9, 144.2, 139.4, 131.8, 129.2, 126.3, 117.0, 20.1, 18.9. HRMS(ESI) m/z: calcd for $C_9H_9INO_3Na^+$ [M+Na]$^+$ 314.9489; found 314.9513.

(E)-5,6-dimethyl-1-styryl-1λ^3-benzo[*d*][1,2]iodaoxol-3(1*H*)-one (2e)

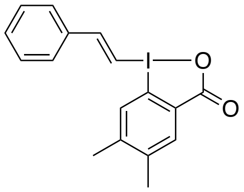

Prepared according to General procedure S2,[3] (326 mg, 0.86 mmol, 58 %). Mp: 174-175 °C. ^{1}H NMR (400 MHz, MeOD-*d*$_4$) δ 8.00 (s, 1H), 7.94 (d, *J* = 15.5 Hz, 1H), 7.75 – 7.69 (m, 2H), 7.65 (d, *J* = 15.5 Hz, 1H), 7.53 – 7.46 (m, 3H), 7.37 (s, 1H), 2.35 (s, 3H), 2.31 (s, 3H);^{13}C NMR (101 MHz, MeOD-*d*$_4$) δ 170.4, 155.5, 145.4, 141.3, 136.8, 134.1, 132.1, 132.0, 130.2, 129.2, 129.0, 112.0, 99.8, 20.2, 19.4. HRMS(ESI) m/z: (M+H) calcd for $C_{17}H_{15}IO_2Na^+$ [M+Na]$^+$ 401.009; found 401.0053.

(*Z*)-*N*-(2-(5,6-dimethyl-3-oxo-1λ^3-benzo[*d*][1,2]iodaoxol-1(3*H*)-yl)vinyl)-*N*-(4-methoxyphenyl)-4-methylbenzenesulfonamide (2p)

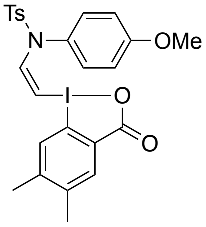

Following a reported procedure,[4] (343 mg, 0.59 mmol, 66%). Mp: 134-135 °C. ^{1}H NMR (400 MHz, $CDCl_3$) δ 8.20 (d, *J* = 8.3 Hz, 1H), 8.12 (s, 1H), 7.60 – 7.52 (m, 2H), 7.37 – 7.30 (m, 2H), 7.15 (s, 1H), 6.78 – 6.66 (m, 4H), 5.48 (d, *J* = 8.3 Hz, 1H), 3.78 (s, 3H), 2.48 (s, 3H), 2.37 (d, *J* = 13.3 Hz, 6H). ^{13}C NMR (101 MHz, CDCl3) δ 166.8, 161.9, 145.6, 143.2, 141.2, 139.9, 133.8, 133.7, 131.9, 130.6, 130.0, 128.0, 125.9, 125.2, 116.0, 112.3, 77.3, 76.7, 74.3, 55.9, 21.8, 20.3, 19.3. HRMS(ESI) m/z: (M+H) calcd for $C_{25}H_{24}INO_5SNa^+$ $[M+Na]^+$ 600.0312; found 600.0342.

1-[Phenylethynyl]-1,2-benziodoxol-3(1*H*)-one (13)

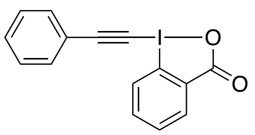

Following the reported procedure,[9] 69 mg, 0.2 mmol, 74%).^{1}HNMR (400 MHz, $CDCl_3$) δ 8.45 – 8.39 (m, 1H), 8.28 – 8.23 (m, 1H), 7.81 – 7.72 (m, 2H), 7.63 – 7.58 (m, 2H), 7.52 –7.40 (m, 3H). ^{13}C NMR 101 MHz, $CDCl_3$) δ 166.7, 135.1, 133.0, 132.7, 131.8, 131.5, 130.9, 128.9, 126.4, 120.7, 116.3, 106.8, 50.3. Analytical data are in accordance to literature.[3]

(*Z*)-*N*-(4-methoxyphenyl)-4-methyl-*N*-(2-(3-oxo-1λ^3-benzo[*d*][1,2]iodaoxol-1(3*H*)-yl)-1-phenylvinyl)benzenesulfonamide (2n)

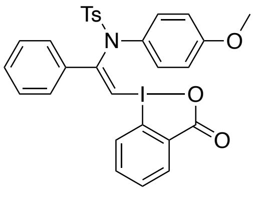

Following a reported procedure,[4] (241 mg, 0.385 mmol, 39%). Mp: 130-131 °C. ^{1}HNMR (400 MHz, $CDCl_3$) δ 8.45 (dd, *J* = 7.2, 2.0 Hz, 1H), 7.69 – 7.58 (m, 2H), 7.53 (dd, *J* = 7.8, 1.4 Hz, 1H), 7.44 (m, 5H), 7.38 – 7.30 (m, 2H), 7.24 – 7.17 (m, 2H), 7.15 – 7.06 (m, 3H), 6.67 – 6.60 (m, 2H), 3.66 (s, 3H), 2.41 (s, 3H). ^{13}C NMR (101 MHz, $CDCl_3$) δ 167.4, 159.3, 156.3, 145.3, 135.9, 135.6, 134.0, 133.2, 131.1, 131.0, 130.9, 129.9, 129.6, 129.0, 128.9, 128.3, 126.7, 115.8, 114.8, 103.6, 55.5, 21.8.

(*Z*)-1-(2-phenyl-2-(*p*-tolyloxy)vinyl)-1λ^3-benzo[*d*][1,2]iodaoxol-3(1*H*)-one (2o)

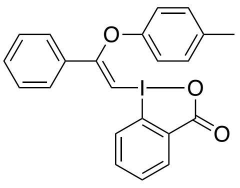

Following a reported procedure,[4] (380 mg, 0.83 mmol, 83%). Mp: 128-129 °C.^{1}H NMR (400 MHz, $CDCl_3$) δ 8.42 – 8.37 (m, 1H), 7.61 – 7.51 (m, 5H), 7.40 – 7.30 (m, 3H), 6.96 – 6.89 (m, 2H), 6.69 – 6.65 (m, 2H), 6.53 (s, 1H), 2.15 (s, 3H). ^{13}C NMR (101 MHz, $CDCl_3$) δ 166.7, 165.7, 153.7, 133.6, 133.2, 131.8, 131.5, 131.0, 130.6, 129.3, 127.9, 125.5, 117.0, 114.7, 86.6, 20.70. HRMS(ESI) m/z: (M+H) calcd for $C_{22}H_{17}IO_3Na^+$ $[M+Na]^+$ 479.0115; found 479.0069.

(*E*)-Styryl(phenyl)iodonium tetrafluoroborate (6)

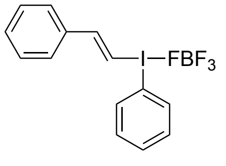

Following the reported procedure,[3] (0.47 g, 1.2 mmol, 79%).^{1}H NMR (400 MHz, $CDCl_3$): δ 8.01 – 8.06 (m, 2H), 7.78 (d, *J* = 14.2 Hz, 1H), 7.57 – 7.63 (m, 1H), 7.42 – 7.50 (m, 5H), 7.31 – 7.38 (m, 3H). Analytical data are in accordance to literature.[3]

3 Investigation on *S*-Vinylation

3.1 Optimization on Thiols

Optimization of thiols were performed on thiophenol **1a**. All reactions were carried out in anhydrous solvents. It was established two different procedures on setting up the reactions. The thiophenol (0.1 mmol, 1.0 equiv) was dissolved in the solvent of choice followed by the addition of the base (1.0 equiv). After 5 minutes the VBX **2a** (1.1equiv, if not otherwise stated) was added to the reaction mixture and stirred at indicated time. Adding the base at the end showed less amount of the product **4a**. Differently, thiophenol (0.1 mmol, 1.0 equiv) was dissolved in the solvent of choice, subsequently VBX **2a** (1.1equiv, if not otherwise stated) was added followed by the addition of the base (1.0 equiv).

Table S1. Additional optimization experiments on thiophenol.

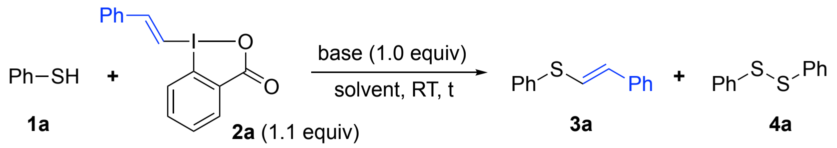

Entry	Solvent	Base	Time (h)	Yield of 3a (%)[a]	*E:Z* ratio	Yield of 4a (%)
**1**	THF	TMG	15	68	15:1	18
**2**	Toluene	TMG	15	53	>20:1	34
**3**	2-MeTHF	TMG	15	38	11:1	31
**4**	THF	-	15	54	20:1	30
**5**	THF	$NaHCO_3$	15	36	9:1	30
**6**	THF	KOH	15	31	9:1	31
**7**	THF	DBU	15	49	16:1	30
**8**	THF	*t*BuOK	15	75	10:1	18
**9**	THF	*t*BuOK	6	72	13:1	18
**10**	THF	*t*BuOK	4	80	15:1	13
**11**	THF	*t*BuOK	2	76[b], [73] [f]	>20:1	13
**12**	THF	*t*BuOK	2	87[b,c], [81] [f]	>20:1	7
**13**	THF	*t*BuOK	2	75	10:1	-
**14**	THF	*t*BuOK	1	61	14:1	-
**15**	THF	*t*BuOK	2	63[d]	14:1	-
**16**	THF	-	15	18[e]	5:1	-
**17**	THF	-	2	6[b]	-	-
**18**	THF	*t*BuOK	2	68[b,c,e]	10:1	-
**19**	THF	TDB	2	79[b,c]	16:1	-
**20**	Toluene	*t*BuOK	2	80[b]	13:1	-

[a] ^{1}H-NMR yield calculated using TMB as internal standard. [b] Addition of VBX followed by the base. [c] Dry and degassed solvent. [d] In the dark. [e] 2 equiv of thiophenol. TMG = 1,1,3,3-Tetramethylguanidine, DBU = 1,8-Diazabicyclo[5.4.0]undec-7-ene. [f] Isolated yield.

General Procedure A for Vinylation of Thiols.

Thiol **1** (1.0 equiv, 0.3 mmol) was placed in an oven-dried microwave vial with magnetic stirring bar under argon, followed by the addition of dry and degassed THF (2.0 mL). Subsequently, VBX **2** (1.1 equiv) was added followed by *t*BuOK (1.0 equiv) and the vial rinsed with THF (1.0 mL), the mixture rapidly turns yellow and it was stirred at RT for 2 h. The reaction was quenched with water (2.0 mL) and the aqueous phase was extracted with DCM (2 x 10 mL) and the combined organic phases were dried over Na_2SO_4, filtered and concentrated under reduce pressure. The reaction crude was purified via column chromatography.

Recovery of Iodoarene

The formed iodobenzoic acid can be recovered and reused in formation of VBX, thus increasing the sustainability and economy of the process both when we use the normal VBX **2a** and the Me_2VBX **2e** after acid work-up and extraction with DCM.

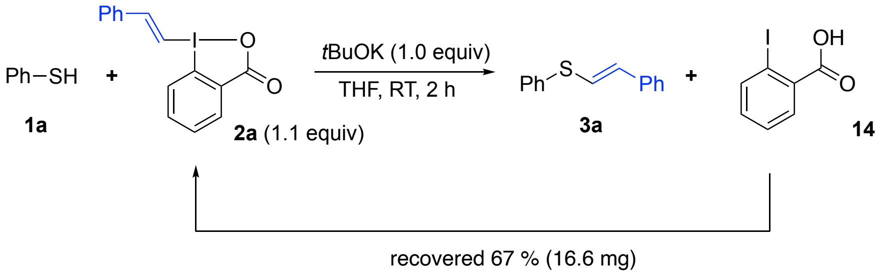

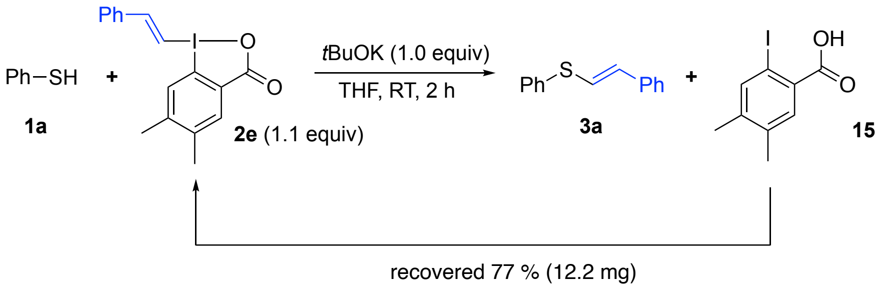

3.2 Mechanistic studies

To investigate if the reaction proceeds through radical species, the radical scavengers (2,2,6,6-tetramethylpiperidin-1-yl)oxyl (TEMPO) and 1,1-diphenylethylene (DPE) were added to the reaction mixture. As displayed in Table S2**,** a lower yields and *E:Z* ratios were obtained with TEMPO. Instead with DPE the yields were almost the same as in standard condition but a slightly lower *E:Z*-ratios were obtained. Based on these results a radical mechanism does not seem to be operating. To investigate the formation of an arene species from the VBX reagent, 5.0 equivalents of furan were added under the optimized reaction conditions. This would allow for trapping the alkyne species through a [4+2] cycloaddition. ^{1}H-NMR of the crude reaction did not show any new products being formed.

Table S2. Additional optimization experiments on thiophenol.

Entry	Additive	Additive (equiv)	Yield of 3a (%)[b]	*E:Z* ratio
**1**	TEMPO	1.0	57	14:1
**2**	TEMPO	3.0	36	10:1
**3**	DPE	1.0	82	15:1
**4**	DPE	3.0	72	14:1
**5**	Furan	5.0	80	18:1

3.3 Reaction of silyl protected thiophenol

Having demonstrated the scope with thiols, the vinylation of silyl-protected thiol was investigated to allow for facile functionalization of complex substrates. Indeed, treatment of **7** with VBX **2a** in the absence of base resulted in 29% yield of **3a**, which could be increased to 77% NMR yield by *in situ*-deprotection with TBAF.

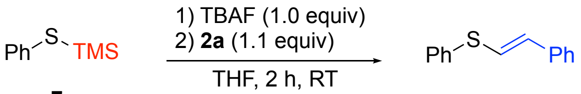

7

3a 77% NMR yield
E:Z >20:1

Procedure: thiohenol **1** (0.1 mmol, 1.0 equiv) was placed in an oven-dried microwave vial with magnetic stirring bar under argon, followed by the addition of dry and degassed THF (0.75 mL). Subsequently, VBX **2a** (1.1 equiv) was added followed by addition drop by drop of TBAF (1.0 equiv) and the vial rinsed with THF (0.25 mL), the mixture rapidly turns yellow and it was stirred at RT for 2 h. The reaction was quenched with water (2.0 mL) and the aqueous phase was extracted with DCM (2 x 10 mL) and the combined organic phases were dried over Na_2SO_4, filtered and concentrated under reduce pressure giving 77% NMR yield with an *E:Z* ratio > 20:1. The yield was calculated employing TBD as internal standard. The crude was purified on column chromatography (pentane) and **3a** was isolated as colorless oil in 76% yield (16 mg), *E:Z*>20:1.

3.4 Optimization on Mercaptothiazoles and byproduct formation

Vinylation of tautomers of thioamides required further optimization, which was performed on 2-mercaptothiazole (**8**). All reactions were carried out in anhydrous and degassed solvents. The mercaptothiazole (0.1 mmol, 1.0 equiv) was dissolved in the solvent of choice, subsequently, VBX **2a** (1.1 equiv, if not otherwise stated) was added, finally the base (1.0 equiv) was added. After the indicated time, the reaction was concentrated at the rotavapor, followed by the addition of water (1.0 mL) and DCM, followed by extraction with DCM (3x5.0 mL). The combined organic phases were dried over Na_2SO_4. Afterwards, the reaction was concentrated at the rotavapor. The reaction crude was purified via column chromatography. NMR yields were determined with TMP as internal standard.

Table S3. Optimization on 2-Mercaptothiazoles

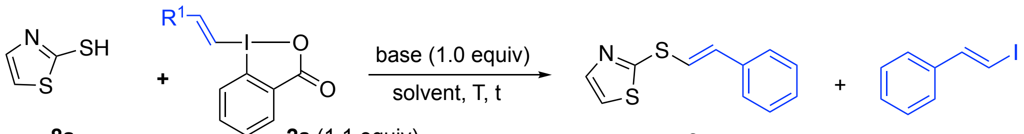

8a **2a** (1.1 equiv) **9a** **10**

Entry	Solvent	Base	Temperature (°C)	Time (h)	NMR Yield of 9a (%)[a,b]	NMR Yield of 10 (%)
**1**	THF	*t*BuOK	RT	2	Traces	-
**2**	THF	*t*BuOK	60	6	44 (43)	-
**3**	Toluene	*t*BuOK	80	6	47	Signals overlapping
**4**	Toluene	*t*BuOK	80	2	48 (45)	9
**5**	Toluene	*t*BuOK	110	2	46	10
**6**	Toluene	*t*BuONa	80	2	23	9
**7**	Toluene	NaH	80	2	29	7
**8**	Toluene	K_2CO_3	80	2	31	5
**9**	Toluene	TBD	80	2	41	7
**10**[c]	Toluene	*t*BuOK	80	2	40	9
**11**	THF	-	RT	2	-	-
**12**	DCM	-	RT	on	- (37)	-

[a] TMB as internal standard. [b] Isolated yield in parenthesis. [c] 1.5 equiv of VBX.

The standard conditions for thiol vinylation did not work in case of 2-mercaptothiazole (Table S3; entry 1). Increasing the temperature to 60° C afforded 44% yield (entry 2). We applied the conditions previously reported in our group for arylation of thioamides[10] by exchanging the solvent from THF to toluene and increasing the temperature to 80 °C, which proved beneficial (entry 3). Increasing the

temperature to 110 °C did not improve the yields (entry 5). Exchanging the base did not increase the yield (entries 6-9). We observed the formation of side product **10** at higher temperature, so we tried increasing the equivalents of VBX (entry 10) but it did not influence the outcome positively. We could not observe the formation of the *N*-vinylated product. The *E/Z* ratio was consistent around 9:1 towards the *E* product.

General Procedure B for Vinylation of Mercaptothiazoles:

The mercaptothiazole (1.0 equiv, 0.3 mmol) was placed in a oven-dried microwave vial with magnetic stirring bar under argon, followed by the addition of anhdrous and degassed toluene (2.0 mL). Subsequently, VBX **2a** (1.1 equiv) was added followed by *t*BuOK (1.0 equiv) and the vial was rinsed with toluene (1.0 mL); then the mixture was stirred at 80 °C for 2 h. Afterwards, the reaction was concentrated at the rotavapor followed by addition of DCM (5.0 mL) and water (5 mL). The aqueous phase was extracted with DCM (2 x 10 mL) and the combined organic phases were dried over Na_2SO_4, filtered and concentrated under reduce pressure. The reaction crude was purified via column chromatography.

Interestingly, VBX reactions of **8a** without base resulted in a different product according to ^{1}H NMR of the crude product, which formed the desired product **9a** in 37% yield during isolation performed on column chromatography (Scheme S1). The crude product is believed to be the unusually stable iodine(III) species **11** with the nucleophile coordinated to iodine, as previously reported in certain reactions with diaryliodonium salts.[11] To the best of our knowledge, such intermediates remain unknown with benziodoxolone reagents. The crude ^{1}H NMR is given in Figure S2, and the spectra in $CDCl_3$ of desired product **9a**, VBX **2a**, intermediate **11** and mercaptobenzothiazole are compared in Figure S3.

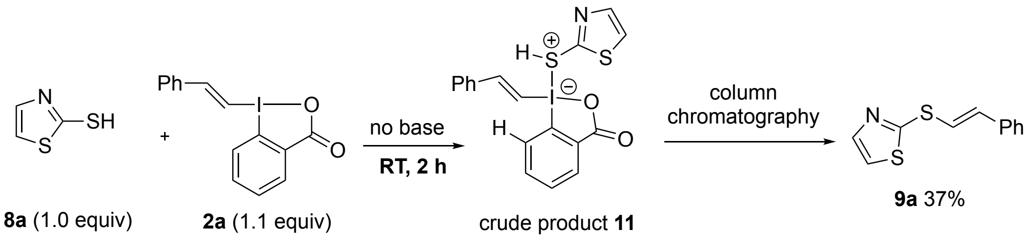

Scheme S1: Proposed formation of intermediate **11**, with subsequent transformation to **9a**.

Compound 11: ^{1}H NMR (400 MHz, $CDCl_3$) δ 13.35 (s, 1H), 8.36 (m, 1H, *ortho H*), 7.76 (d, *J* = 15.6 Hz, 1H), 7.59 – 7.24 (m, 9H), 7.16 (d, *J* = 4.5 Hz, 1H), 6.46 (d, *J* = 4.5 Hz, 1H).

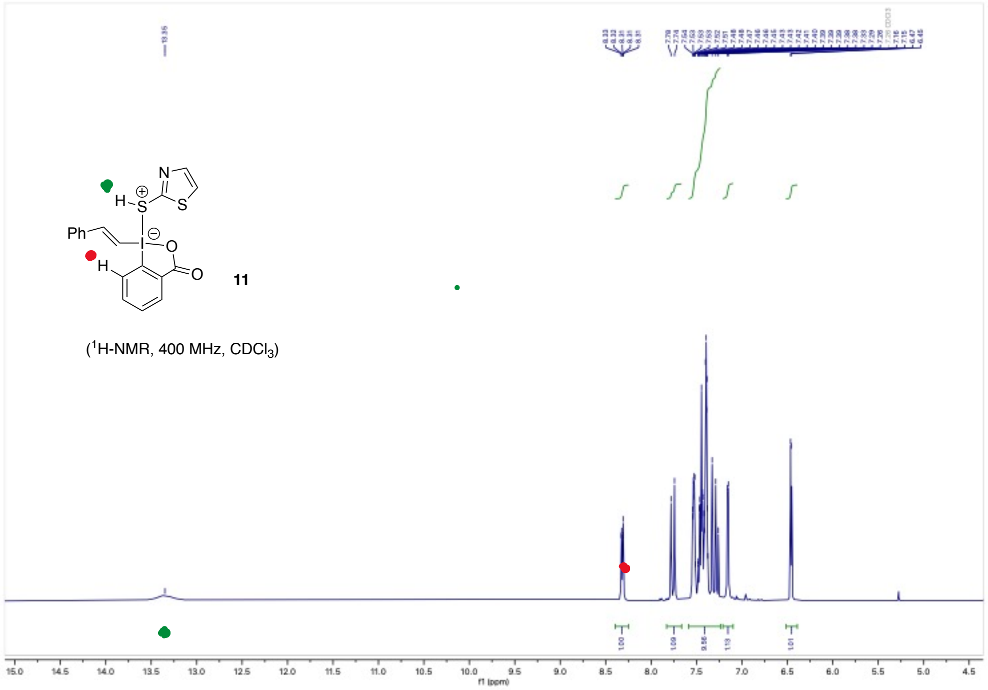

Figure S2. ^{1}H-NMR spectra (in $CDCl_3$) of product **11.**

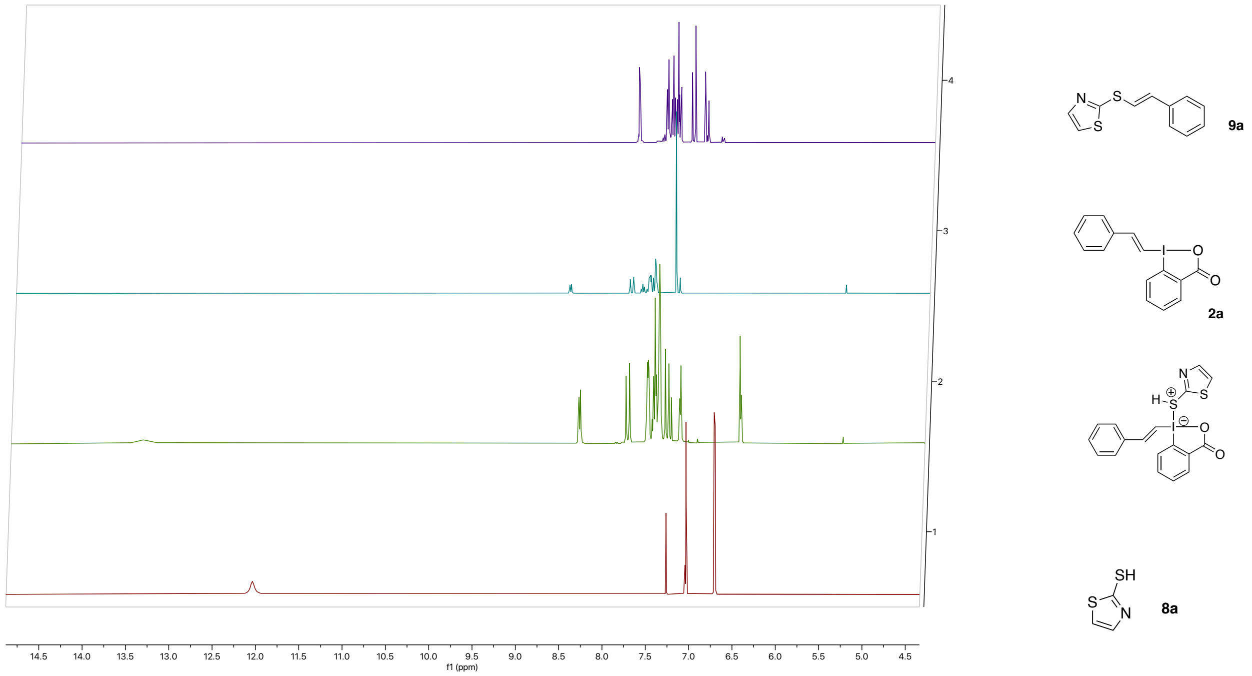

Figure S3. Comparison of ^{1}H-NMR spectra (in $CDCl_3$) of desired product **9a**, VBX **2a**, product **11**, and 2-mercaptothiazole **8a**.

4 Analytical Data

4.1 Products from Vinylation of Thiols

(*E*)-phenyl(styryl)sulfane (3a)

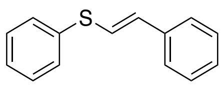

Following *General Procedure A,* thiophenol (33 mg, 0.3 mmol, 1.0 equiv), VBX **2a** (116 mg, 0.33 mmol, 1.1 equiv) and finally *t*BuOK (34 mg, 0.3 mmol, 1.0 equiv) were allowed to react in THF (3.0 mL) for 2 h at RT. The crude was purified on column chromatography (pentane) and **3a** was isolated as colorless oil in 78% yield (50 mg), *E:Z*>20:1.

The reaction was done also at 0.1 mmol following *General Procedure A.* Thiophenol (11 mg, 0.1 mmol, 1.0 equiv), VBX **2a** (39 mg, 0.11 mmol, 1.1 equiv) and finally *t*BuOK (11 mg, 0.1 mmol, 1.0 equiv) were allowed to react in THF (3.0 mL) for 2 h at RT. The crude was purified on column chromatography (pentane) and **3a** was isolated as colorless oil in 81% yield (17 mg), *E:Z*>20:1.

When the reaction was done with VBX **2e** following a *General Procedure A,* 90% yield of the **3a** was obtained. The crude did not need further purification.

^{1}H NMR (400 MHz, $CDCl_3$) δ 7.53 – 7.36 (m, 2H), 7.40 – 7.19 (m, 8H), 6.90 (d, *J* = 15.5 Hz, 1H), 6.75 (d, *J* = 15.5 Hz, 1H).^{13}C NMR (101 MHz, $CDCl_3$) δ 136.7, 135.4, 132.0, 130.0, 129.3, 128.8, 127.7, 127.1, 126.2, 123.5. HRMS(ESI) m/z: calcd for $C_{14}H_{12}SH^+$ $[M+H]^+$ 212.0654; found 212.0651. Analytical data are in accordance with literature values.[12]

(*E*)-naphthalen-2-yl(styryl)sulfane (3b)

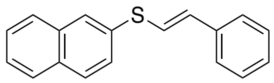

Following *General Procedure A,* naphthalene-2-thiol (48 mg, 0.3 mmol, 1.0 equiv), VBX **2a** (116 mg, 0.33 mmol, 1.1 equiv) and finally *t*BuOK (34 mg, 0.3 mmol, 1.0 equiv) were allowed to react in THF (3.0 mL) for 2 h at RT. The crude was purified on column chromatography (pentane) and **3b** was isolated as white solid in 72% yield (56 mg), *E:Z* 20:1.

Mp: 67-69 °C.^{1}H NMR (400 MHz, $CDCl_3$) δ 7.85 – 7.77 (m, 4H), 7.54 – 7.30 (m, 8H), 6.99 (d, *J* = 15.4 Hz, 1H), 6.81 (d, *J* = 15.4 Hz, 1H). ^{13}C NMR (101 MHz, $CDCl_3$) δ 136.7, 133.9, 132.7, 132.4, 129.1, 128.91, 128.87, 128.3, 127.9, 127.8, 127.7, 127.5, 126.9, 126.4, 126.2, 123.3. Analytical data are in accordance with literature values.[13]

(*E*)-(4-methoxyphenyl)(styryl)sulfane (3c)

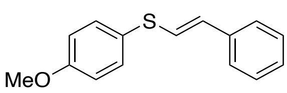

Following *General Procedure A,* 4-methoxylbenzenethiol (37 mg, 0.3 mmol, 1.0 equiv VBX **2a** (116 mg, 0.33 mmol, 1.1 equiv) and finally *t*BuOK (34 mg, 0.3 mmol, 1.0 equiv), were allowed to react in THF (3.0 mL) for 3 h at RT. The crude was purified on column chromatography (pentane) and **3c** was isolated as white solid in 73% yield (51 mg), *E:Z* >20:1.

Mp: 56-57 °C.^{1}H NMR (400 MHz, $CDCl_3$) δ 7.45 – 7.38 (m, 2H), 7.32 – 7.27 (m, 4H), 7.23 – 7.17 (m, 1H), 6.94 – 6.88 (m, 2H), 6.84 (d, *J* = 15.4 Hz, 1H), 6.52 (d, *J* = 15.4 Hz, 1H), 3.83 (s, 3H).^{13}C NMR (101 MHz, $CDCl_3$) δ 159.7, 136.9, 133.6, 129.1, 128.8, 127.3, 125.9, 125.9, 124.6, 115.0, 55.5. Analytical data are in accordance with literature values.[14]

(*E*)-(4-tertbutylphenyl)(styryl)sulfane (3d)

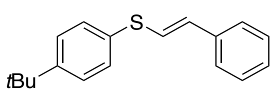

Following *General Procedure A,* 4-tertbutylbenzenethiol (49 mg, 0.3 mmol, 1.0 equiv), VBX **2a** (116 mg, 0.33 mmol, 1.1 equiv) and finally *t*BuOK (34 mg, 0.3 mmol, 1.0 equiv), were allowed to react in THF (3.0 mL) for 2 h at RT. The crude was purified on column chromatography (pentane) and **3c** was isolated as white solid in 77% yield (62 mg), *E:Z* >20:1.

Mp: 45-46 °C. ^{1}H NMR (400 MHz, $CDCl_3$) δ 7.44 – 7.28 (m, 8H), 7.27 – 7.19 (m, 1H), 6.89 (d, *J* = 15.5 Hz, 1H), 6.70 (d, *J* = 15.5 Hz, 1H), 1.33 (s, 9H). ^{13}C NMR (101 MHz, $CDCl_3$) δ 150.6, 136.8, 131.6, 130.9, 130.3, 128.8, 127.6, 126.4, 126.1, 124.5, 34.7, 31.4. Analytical data are in accordance with literature values.[15]

(*E*)-styryl(*p*-tolyl)sulfane (3e)

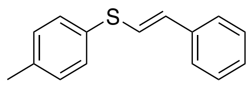

Following *General Procedure A,* 4-methylbenzenethiol (37 mg, 0.3 mmol, 1.0 equiv), VBX **2a** (116 mg, 0.33 mmol, 1.1 equiv) and finally *t*BuOK (34 mg, 0.3 mmol, 1.0 equiv) were allowed to react in THF (3.0 mL) for 2 h at RT. The crude was purified on column chromatography (pentane) and **3e** was isolated as light yellow oil in 71% yield (48 mg), *E:Z* 16:1.

^{1}H NMR (400 MHz, $CDCl_3$) δ 7.38 – 7.28 (m, 6H), 7.24 (dd, *J* = 6.9, 1.8 Hz, 1H), 7.19 – 7.14 (m, 2H), 6.87 (d, *J* = 15.5 Hz, 1H), 6.66 (d, *J* = 15.5 Hz, 1H), 2.37 (s, 3H). Minor: ^{1}H NMR (400 MHz, $CDCl_3$) δ 6.56 (d, *J* = 10.8 Hz, 1H), 6.48 (d, *J* = 10.8 Hz, 1H), 2.33 (s, 3H).^{13}C NMR (101 MHz, $CDCl_3$) δ 137.4, 136.8, 131.3, 130.8, 130.6, 130.1, 128.8, 127.5, 126.1, 124.6, 21.2. Analytical data are in accordance with literature values.[13]

(*E*)-(3-methyl)(styryl)sulfane (3f)

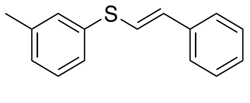

Following *General Procedure A,* 3-methylbenzenethiol (37 mg, 0.3 mmol, 1.0 equiv), VBX **2a** (116 mg, 0.33 mmol, 1.1 equiv) and finally *t*BuOK (34 mg, 0.3 mmol, 1.0 equiv) were allowed to react in THF (3.0 mL) for 2 h at 50 °C. The crude was purified on column chromatography (pentane) and **3f** was isolated as pale yellow oil in 66% yield (44 mg), *E:Z* 16:1.

^{1}H NMR (400 MHz, $CDCl_3$) δ 7.40 – 7.30 (m, 4H), 7.29 – 7.22 (m, 4H), 7.10 (m, *J* = 5.3, 3.5, 2.0 Hz, 1H), 6.92 (d, *J* = 15.5 Hz, 1H), 6.76 (d, *J* = 15.5 Hz, 1H), 2.37 (s, 3H).Minor: ^{1}H NMR (400 MHz, $CDCl_3$) δ 6.62 (d, *J* = 10.8 Hz, 1H), 6.55 (d, *J* = 10.8 Hz, 1H), 2.34 (s, 3H). ^{13}C NMR (101 MHz, $CDCl_3$) δ 139.2, 136.7, 135.0, 131.6, 130.5, 129.1, 128.8, 128.0, 127.7, 127.0, 126.2, 123.8, 21.5. Analytical data are in accordance with literature values.[16]

(*E*)-(2,6-dimethylphenyl)(styryl)sulfane (3g)

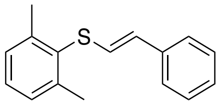

Following a modified *General Procedure A,* 2,6-dimethylbenzenethiol (42 mg, 0.3 mmol, 1.0 equiv), VBX **2a** (116 mg, 0.33 mmol, 1.1 equiv) and finally *t*BuOK (34 mg, 0.3 mmol, 1.0 equiv) were allowed to react in THF (3.0 mL) for 2 h at 50 °C. The crude was purified on column chromatography (pentane) and **3g** was isolated as pale yellow oil in 68% yield (49 mg), *E:Z* >20:1.

^{1}H NMR (400 MHz, $CDCl_3$) δ 7.30 – 7.14 (m, 8H), 6.69 (d, *J* = 15.3 Hz, 1H), 6.01 (d, *J* = 15.3 Hz, 1H), 2.53 (s, 6H). ^{13}C NMR (101 MHz, $CDCl_3$) δ 143.5, 137.2, 130.0, 129.4, 128.7, 128.5, 126.8, 125.6, 125.3, 124.7, 21.9. Analytical data are in accordance with literature values.[17]

(*E*)-(2-fluorophenyl)(styryl)sulfane (3h)

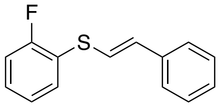

Following *General Procedure A,* 2-fluorobenzenethiol (38 mg, 0.3 mmol, 1.0 equiv), VBX **2a** (116 mg, 0.33 mmol, 1.1 equiv) and finally *t*BuOK (34 mg, 0.3 mmol, 1.0 equiv) were allowed to react in THF (3.0 mL) for 2 h at RT. The crude was purified on column chromatography (pentane) and **3h** was isolated as colorless oil in 52 % yield (36 mg), *E:Z.*>20:1.

^{1}H NMR (400 MHz, $CDCl_3$) δ 7.43 (td, *J* = 7.5, 1.7 Hz, 1H), 7.37 – 7.22 (m, 6H), 7.17 – 7.08 (m, 2H), 6.81 (d, *J* = 15.5 Hz, 1H), 6.75 (d, *J* = 15.5 Hz, 1H). ^{13}C NMR (101 MHz, $CDCl_3$) δ 160.9 (d, *J* = 247.4 Hz), 136.5, 132.8, 132.2 (d, *J* = 1.6 Hz), 129.2 (d, *J* = 7.3 Hz), 128.8, 127.9, 126.2, 124.9 (d, *J* = 3.8 Hz), 122.4 (d, *J* = 17.0 Hz), 121.7 (d, *J* = 1.9 Hz), 116.0 (d, *J* = 21.6 Hz). ^{19}F NMR (377 MHz, $CDCl_3$) δ –109.7. Analytical data are in accordance with literature values.[16]

(*E*)-(4-bromophenyl)(styryl)sulfane (3i)

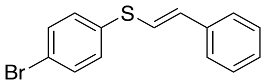

Following *General Procedure A,* 4-bromobenzenethiol (57 mg, 0.3 mmol, 1.0 equiv), VBX **2a** (116 mg, 0.33 mmol, 1.1 equiv) and finally *t*BuOK (34 mg, 0.3 mmol, 1.0 equiv) were allowed to react in THF (3.0 mL) for 2 h at RT. The crude was purified on column chromatography (pentane) and **3i** was isolated as white solid in 75% yield (65 mg), *E:Z* >20:1.

When the reaction was done with VBX **2e** following a *General Procedure A,* 77% yield of the **3i** was obtained. The crude did not need further purification.

Mp: 75-76 °C. ^{1}H NMR (400 MHz, $CDCl_3$) δ 7.47 – 7.45 (m, 2H), 7.37 – 7.31 (m, 4H), 7.28 – 7.26 (m, 3H), 6.84 (d, *J* = 15.4 Hz, 1H), 6.78 (d, *J* = 15.4 Hz, 1H).^{13}C NMR (101 MHz, $CDCl_3$) δ 136.4, 134.8, 133.2, 132.4, 131.3, 128.9, 128.0, 126.3, 122.4, 121.0. Analytical data are in accordance with literature values.[14]

(*E*)-(4-chlorophenyl)(styryl)sulfane (3j)

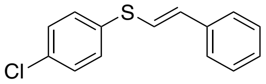

Following *General Procedure A,* 4-chlorobenzenethiol (43 mg, 0.3 mmol, 1.0 equiv), VBX **2a** (116 mg, 0.33 mmol, 1.1 equiv) and finally *t*BuOK (34 mg, 0.3 mmol, 1.0 equiv) were allowed to react in THF (3.0 mL) for 2 h at RT. The crude was purified on column chromatography (pentane) and **3j** was isolated as white solid in 70% yield (51 mg), *E:Z* >20:1.

Mp: 46-47 °C.^{1}H NMR (400 MHz, $CDCl_3$) δ 7.37 – 7.29 (m, 8H), 7.28 – 7.25 (m, 1H), 6.82 (d, *J* = 15.4 Hz, 1H), 6.75 (d, *J* = 15.5 Hz, 1H).^{13}C NMR (101 MHz, $CDCl_3$) δ 136.4, 134.0, 133.2, 132.9, 131.1, 129.5, 128.9, 128.0, 126.3, 122.7.
Analytical data are in accordance with literature values.[14]

(*E*)-(3-chlorophenyl)(styryl)sulfane (3k)

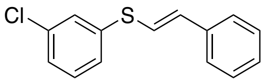

Following *General Procedure A,* 3-chlorobenzenethiol (43 mg, 0,3 mmol, 1.0 equiv) and VBX **2a** (116 mg, 0.33 mmol, 1.1 equiv) and finally *t*BuOK (34 mg, 0.3 mmol, 1.0 equiv) were allowed to react in THF (3.0 mL) for 2 h at RT. The crude was purified on column chromatography (pentane) and **3k** was isolated as light yellow oil in 87% yield (64 mg), *E:Z* >20:1.

^{1}H NMR (400 MHz, $CDCl_3$) δ 7.41 – 7.33 (m, 5H), 7.31 – 7.19 (m, 4H), 6.86 (d, *J* = 15.6 Hz, 2H), 6.81 (d, *J* = 15.6 Hz, 1H).^{13}C NMR (101 MHz, $CDCl_3$) δ 137.9, 136.4, 135.1, 134.1, 130.3, 128.9, 128.9, 128.2, 127.3, 127.0, 126.4, 121.7. HRMS(ESI) m/z: calcd for $C_{14}H_{12}S$ [M] 246.0283; found 246.0265.Analytical data are in accordance with literature values.[18]

(*E*)-benzyl(styryl)sulfane (3l)

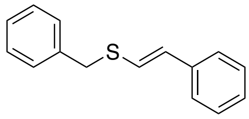

Following *General Procedure A,* phenylmethanethiol (35 mg, 0,3 mmol, 1.0 equiv) and VBX **2a** (116 mg, 0.33 mmol, 1.1 equiv) and finally *t*BuOK (34 mg, 0.3 mmol, 1.0 equiv) were allowed to react in THF (3.0 mL) for 2 h at RT. The crude was purified on column chromatography (pentane) and **3l** was isolated as colorless oil in 77% yield (52 mg), *E:Z* >20:1.

When the reaction was done with VBX **2e** following a *General Procedure A,* 97% yield of the **3l** was obtained. The crude did not need further purification.

Mp:70-71 °C. ^{1}H NMR (400 MHz, $CDCl_3$) δ 7.41 – 7.32 (m, 4H), 7.31 – 7.23 (m, 5H), 7.22 – 7.15 (m, 1H), 6.72 (d, *J* = 15.6 Hz, 1H), 6.53 (d, *J* = 15.5 Hz, 1H), 4.02 (s, 2H). ^{13}C NMR (101 MHz, $CDCl_3$) δ 137.4, 137.1, 129.0, 128.8, 128.8, 128.1, 127.5, 127.1, 125.7, 124.5, 37.5. Analytical data are in accordance with literature values.[13]

(*E*)-hexyl(styryl)sulfane (3m)

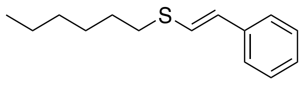

Following *General Procedure A,* hexane-1-thiol (35 mg, 0,3 mmol, 1.0 equiv) and VBX **2a** (116 mg, 0.33 mmol, 1.1 equiv) and finally *t*BuOK (34 mg, 0.3 mmol, 1.0 equiv) were allowed to react in THF (3.0 mL) for 2 h at RT. The crude was purified on column chromatography (pentane) and **3m** was isolated as colorless oil in 71% yield (47 mg), *E:Z* >20:1.

^{1}H NMR (400 MHz, $CDCl_3$) δ 7.30 (d, *J* = 4.3 Hz, 4H), 7.21 – 7.17 (m, 1H), 6.73 (d, *J* = 15.6 Hz, 1H), 6.47 (d, *J* = 15.5 Hz, 1H), 2.82 – 2.79 (t, *J* = 14.8, 7.4 Hz 2H), 1.75 – 1.65 (m, 2H), 1.49 – 1.40 (m, 2H), 1.39 – 1.27 (m, 4H), 0.95 – 0.87 (m, 3H). ^{13}C NMR (101 MHz, $CDCl_3$) δ 137.3, 128.8, 126.9, 126.8, 125.6, 125.5, 32.8, 31.5, 29.6, 28.6, 22.7, 14.2. Analytical data correspond with literature values.[19]

(*E*)-isopropyl(styryl)sulfane (3n)

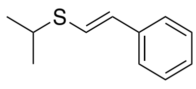

Following *General Procedure A,* propane-2-thiol (7.6 mg, 0,3 mmol, 1.0 equiv) and VBX **2a** (116 mg, 0.33 mmol, 1.1 equiv) and finally *t*BuOK (34 mg, 0.3 mmol, 1.0 equiv) were allowed to react in THF (3.0 mL) for 2 h at RT. The crude was purified on column chromatography (pentane) and **3n** was isolated as colorless oil in 67% yield (36 mg), *E:Z* >20:1.

^{1}H NMR (400 MHz, $CDCl_3$) δ 7.34 – 7.27 (m, 4H), 7.23 – 7.16 (m, 1H), 6.77 (d, *J* = 15.6 Hz, 1H), 6.58 (d, *J* = 15.6 Hz, 1H), 3.25 (p, *J* = 6.7 Hz, 1H), 1.38 (d, *J* = 6.8 Hz, 6H).^{13}C NMR (101 MHz, $CDCl_3$) δ 137.3, 129.0, 128.8, 127.1, 125.8, 124.2, 37.0, 23.6. Analytical data are in accordance with literature values.[12]

(*E*)-*tert*-butyl(styryl)sulfane (3o)

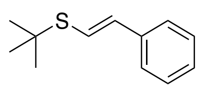

Following *General Procedure A,* 2-methylpropane-2-thiol (27 mg, 0,3 mmol, 1.0 equiv) and VBX **2a** (116 mg, 0.33 mmol, 1.1 equiv) and finally *t*BuOK (34 mg, 0.3 mmol, 1.0 equiv) were allowed to react in THF (3.0 mL) for 2 h at RT. The crude was purified on column chromatography (pentane) and **3o** was isolated as colorless oil in 52% yield (30 mg), *E:Z* >20:1.

^{1}H NMR (400 MHz, $CDCl_3$) δ 7.39 – 7.28 (m, 4H), 7.24 – 7.19 (m, 1H), 6.88 (d, *J* = 15.5 Hz, 1H), 6.72 (d, *J* = 15.5 Hz, 1H), 1.41 (s, 9H).^{13}C NMR (101 MHz, $CDCl_3$) δ 137.2, 132.2, 128.8, 127.4, 126.0, 122.2, 44.5, 31.2. Analytical data are in accordance with literature values.[12]

(*E*)-cyclohexyl(styryl)sulfane (3p)

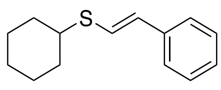

Following *General Procedure A,* cyclohexanethiol (35 mg, 0,3 mmol, 1.0 equiv) and VBX **2a** (116 mg, 0.33 mmol, 1.1 equiv) and finally *t*BuOK (34 mg, 0.3 mmol, 1.0 equiv) were allowed to react in THF (3.0 mL) for 2 h at RT. The crude was purified on column chromatography (pentane) and **3p** was isolated as colorless oil in 85% yield (55 mg), *E:Z* >20:1.

^{1}H NMR (400 MHz, $CDCl_3$) δ 7.28 – 7.23 (m, 4H), 7.19 – 7.11 (m, 1H), 6.73 (d, *J* = 15.6 Hz, 1H), 6.53 (d, *J* = 15.6 Hz, 1H), 2.98– 291 (m, 1H), 2.07 – 1.97 (m, 2H), 1.76 (m, 2H), 1.66 – 1.55 (m, 1H), 1.46 – 1.19 (m, 5H). ^{13}C NMR (101 MHz, $CDCl_3$) δ 137.3, 128.8, 128.7, 127.0, 125.7, 124.2, 45.5, 33.7, 26.2, 25.8. Analytical data are in accordance with literature values.[12]

(*E*)-cyclopentyl(styryl)sulfane (3q)

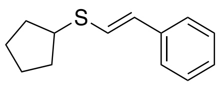

Following *General Procedure A,* cyclopentanethiol (31 mg, 0,3 mmol, 1.0 equiv) and VBX **2a** (116 mg, 0.33mmol, 1.1 equiv) and finally *t*BuOK (34 mg, 0.3 mmol, 1.0 equiv) were allowed to react in THF (3.0 mL) for 2 h at RT. The crude was purified on column chromatography (pentane) and **3q** was isolated as colorless oil in 69% yield (42 mg), *E:Z* >20:1.

^{1}H NMR (400 MHz, $CDCl_3$) δ 7.38 – 7.27 (m, 4H), 7.24 – 7.10 (m, 1H), 6.78 (d, *J* = 15.6 Hz, 1H), 6.51 (d, *J* = 15.6 Hz, 1H), 3.56 – 3.38 (m, 1H), 2.18 – 2.02 (m, 2H), 1.84 – 1.72 (m, 2H), 1.71 – 1.59 (m, 4H). ^{13}C NMR (101 MHz, $CDCl_3$) δ 137.4, 128.8, 127.8, 126.9, 125.6, 125.3, 44.9, 33.7, 25.1. Analytical data are in accordance with literature values.[20]

(*E*)-allyl(styryl)sulfane (3r)

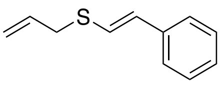

Following *General Procedure A,* prop-2-ene-1-thiol (37 mg, 0,3 mmol, 1.0 equiv) and VBX **2a** (116 mg, 0.33 mmol, 1.1 equiv) and finally *t*BuOK (34 mg, 0.3 mmol, 1.0 equiv) were allowed to react in THF (3.0 mL) for 2 h at RT. The crude was purified on column chromatography (pentane) and **3r** was isolated as colorless oil in 71% yield (47 mg), *E:Z* >20:1.

^{1}H NMR (400 MHz, $CDCl_3$) δ 7.29 (d, *J* = 4.8 Hz, 4H), 7.23–7.16 (m, 1H), 6.69 (d, *J* = 15.6 Hz, 1H), 6.55 (d, *J* = 15.6 Hz, 1H),), 5.91 (ddt, *J* = 16.9 Hz, 10.0 Hz, 6.9 Hz, 1H), 5.26 (dq, *J* = 17.0 Hz, 1.4 Hz, 1H), 5.18 (dq, *J* = 10.0 Hz, 1.2 Hz, 1H), 3.43 (dt, *J* = 6.9, 1.2 Hz, 2H). ^{13}C-NMR (101 MHz, CDCl3) δ 137.1, 133.8, 128.8, 128.1, 127.1, 125.7, 124.2, 118.0, 36.0. Analytical data are in accordance with literature values.[21]

(*E*)-2-((styrylthio)methyl)furan (3s)

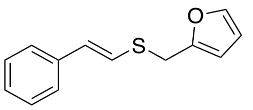

Following *General Procedure A,* furan-2-ylmethanethiol (33 mg, 0,3 mmol, 1.0 equiv) and VBX **2a** (115 mg, 0.33 mmol, 1.1 equiv) and finally *t*BuOK (34 mg, 0.3 mmol, 1.0 equiv) were allowed to react in THF (3.0 mL) for 2 h at RT. The crude was purified on column chromatography (pentane:Et_2O 98:2 *v/v*) and **3s** was isolated as colorless oil in 79% yield (51 mg), *E:Z* >20:1.

^{1}H NMR (400 MHz, $CDCl_3$) δ 7.38 (dd, *J* = 1.9, 0.9 Hz, 1H), 7.32 – 7.24 (m, 4H), 7.20 (m, 1H), 6.74 (d, *J* = 15.6 Hz, 1H), 6.57 (d, *J* = 15.6 Hz, 1H), 6.33 (dd, *J* = 3.2, 1.9 Hz, 1H), 6.29 – 6.23 (m, 1H), 4.00 (s, 2H). ^{13}C NMR (101 MHz, $CDCl_3$) δ 150.9, 142.4, 136.8, 128.7, 128.6, 127.1, 125.7, 123.8, 110.6, 107.9, 29.7. Analytical data are in accordance with literature values.[12]

2-(styrylthio)pyridine (3t)

Following *General Procedure A,* pyridine-2-thiol (33 mg, 0,3 mmol, 1.0 equiv) and VBX **2a** (115 mg, 0.33 mmol, 1.1 equiv) and finally *t*BuOK (34 mg, 0.3 mmol, 1.0 equiv) were allowed to react in THF (3.0 mL) for 2 h at 50°C. The crude was purified on column chromatography (pentane:Et_2O 90:10 *v/v*) and **3t** was isolated as yellow solid in 60% yield (38 mg), *E:Z* 5:1.

Analytical data for *E*-3t

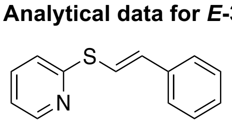

Mp: 117-119 °C.^{1}H NMR (400 MHz, $CDCl_3$) δ 8.51 (ddd, *J* = 4.9, 1.9, 0.9 Hz, 1H), 7.57 (ddd, *J* = 8.1, 7.4, 1.9 Hz, 1H), 7.50 (d, *J* = 15.9 Hz, 1H), 7.48 – 7.43 (m, 2H), 7.38 – 7.32 (m, 2H), 7.31 – 7.24 (m, 2H), 7.08 (ddd, *J* = 7.4, 4.9, 1.1 Hz, 1H), 6.91 (d, *J* = 15.9 Hz, 1H).^{13}C NMR (101 MHz, $CDCl_3$) δ 158.2, 150.0, 136.7, 136.6, 132.2, 128.8, 127.9, 126.4, 122.2, 120.4, 119.9. Analytical data are in accordance with literature values.[15]

Analytical data for *Z*-3t

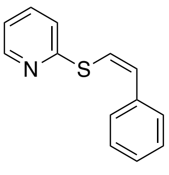

Mp: 60-62 °C.^{1}H NMR (400 MHz, $CDCl_3$) δ 8.53 (ddd, *J* = 4.9, 1.9, 1.0 Hz, 1H), 7.62 – 7.50 (m, 3H), 7.46 – 7.36 (m, 3H), 7.32 – 7.24 (m, 2H), 7.08 (ddd, *J* = 7.3, 4.9, 1.1 Hz, 1H), 6.75 (d, *J* = 11.0 Hz, 1H).^{13}C NMR (101 MHz, $CDCl_3$) δ 156.7, 149.9, 136.9, 136.7, 128.9, 128.5, 127.6, 127.3, 123.0, 120.7, 120.3. Analytical data are in accordance with literature values.[22]

1,3-bis-(*E*)-(styrylthio)propane (3u)

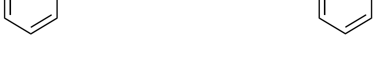

Following *General Procedure A,* propane-1,3-dithiol (33 mg, 0,3 mmol, 1.0 equiv) and VBX **2a** (221 mg, 0.66 mmol, 2.2 equiv) and finally *t*BuOK (68 mg, 0.6 mmol, 2.0 equiv) were allowed to react in THF (3.0 mL) for 2 h at RT. The crude was purified on column chromatography (pentane) and **3u** was isolated as colourless oil in 78% yield (73 mg), *E:Z* >20:1.

^{1}H NMR (400 MHz, $CDCl_3$) δ 7.35 (m, 8H), 7.26 (m, 2H), 6.77 (d, *J* = 15.6 Hz, 2H), 6.60 (d, *J* = 15.6 Hz, 2H), 3.03 (t, *J* = 7.0 Hz, 4H), 2.16 (p, *J* = 7.0 Hz, 2H). ^{13}C NMR (101 MHz, $CDCl_3$) δ 137.0, 128.8, 127.9, 127.1, 125.7, 124.5, 31.3, 29.2. HRMS(ESI) m/z: calcd for $C_{14}H_{12}SH^+$ $[M+H]^+$ 313.1079; found 313.1088.

1,4-bis(((*E*)-styryl)thio)benzene (3v)

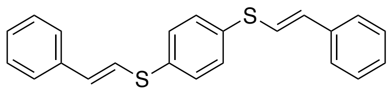

Following *General Procedure A,* propane-1,4-dithiol (33 mg, 0,3 mmol, 1.0 equiv) and VBX **2a** (221 mg, 0.66 mmol, 2.2 equiv) and finally *t*BuOK (68 mg, 0.6 mmol, 2.0 equiv) were allowed to react in THF (3.0 mL) for 2 h at RT. The crude was purified on column chromatography (pentane) and **3v** was isolated as colorless oil in 54% yield (56 mg), *E:Z* >20:1.

Mp:.117-119 °C.^{1}H NMR (400 MHz, $CDCl_3$) δ 7.43 – 7.32 (m, 12H), 7.28 (td, *J* = 3.9, 1.4 Hz, 3H), 6.88 (d, *J* = 15.4 Hz, 2H), 6.78 (d, *J* = 15.4 Hz, 2H). ^{13}C NMR (101 MHz, $CDCl_3$) δ 136.5, 134.3, 132.7, 130.5, 128.8, 127.9, 126.2, 122.9. HRMS(ESI) m/z: calcd for $C_{22}H_{18}S_2H^+$ $[M+H]^+$ 347.0923; found 347.0936.

(*E*)-2-(styrylthio)ethan-1-ol (3w)

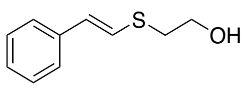

Following *General Procedure A,* 2-mercaptoethan-1-ol (24 mg, 0,1 mmol, 1.0 equiv) and VBX **2a** (115 mg, 0.33 mmol, 1.1 equiv) and finally *t*BuOK (34 mg, 0.3 mmol, 1.0 equiv) were allowed to react in THF (3.0 mL) for 2 h at RT. The crude was purified on column chromatography (pentane:Et_2O 90:10 *v/v*) and **3w** was isolated as colorless oil in 65% yield (35 mg), *E:Z* >20:1.

^{1}H NMR (400 MHz, $CDCl_3$) δ 7.34 – 7.27 (m, 4H), 7.21 (ddt, *J* = 7.4, 6.2, 2.2 Hz, 1H), 6.68 (d, *J* = 15.5 Hz, 1H), 6.59 (d, *J* = 15.5 Hz, 1H), 3.85 (q, *J* = 5.7 Hz, 2H), 3.00 (t, *J* = 5.9 Hz, 2H), 2.01 (s, 1H). ^{13}C NMR (101 MHz, $CDCl_3$) δ 136.8, 129.2, 128.8, 127.4, 125.8, 123.8, 61.2, 36.2. The analytical data are in accordance with literature values.[13]

Ethyl (*E*)-*S*-styrylcysteinate (3x)

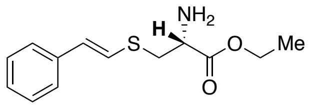

Following *General Procedure A,* ethyl L-cysteinate (45 mg, 0,3 mmol, 1.0 equiv) and VBX **2a** (115 mg, 0.33 mmol, 1.1 equiv) and finally *t*BuOK (34 mg, 0.3 mmol, 1.0 equiv) were allowed to react in THF (3.0 mL) for 2 h at RT. The crude was purified on column chromatography on deactivated silica (2% TEA) (DCM:NH_3 in MeOH 99:1 *v/v*) and **3x** was isolated as colorless oil in 60% yield (45 mg), *E:Z* >20:1. Chiral SFC (Chiralpak IF-3, 25 °C, 0.3 cm ϕ, 15 cm column, 10% MeOH in CO_2, flow rate: 0.8 mL/min; tR: 14.7 min (major enantiomer) , 15.9 (minor enantiomer); ee (major enantiomer) = 99%

^{1}H NMR (400 MHz, $CDCl_3$) δ 7.29 (d, *J* = 4.9 Hz, 4H), 7.21 (ddd, *J* = 6.4, 4.6, 3.0 Hz, 1H), 6.69 (d, *J* = 15.5 Hz, 1H), 6.59 (d, *J* = 15.6 Hz, 1H), 4.18 (qd, *J* = 7.1, 0.8 Hz, 2H), 3.74 (dd, *J* = 7.1, 4.5 Hz, 1H), 3.21 (dd, *J* = 13.8, 4.5 Hz, 1H), 3.03 (dd, *J* = 13.8, 7.2 Hz, 1H), 1.75 (s, 2H), 1.28 (t, *J* = 7.1 Hz, 3H). ^{13}C NMR (101 MHz, $CDCl_3$) δ 173.8, 136.8, 129.3, 128.8, 127.4, 125.8, 124.2, 61.6, 54.5, 38.3, 14.3. HRMS(ESI) m/z: calcd for $C_{13}H_{17}NO_2SNa^+$ $[M+Na]^+$ 274.0872; found 274.0857. The analytical data are in accordance with literature values.[23]

(*E*)-3-(styrylthio)aniline (3y)

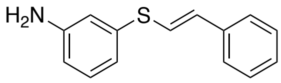

Following a modified *General Procedure A,* 3-aminobenzenethiol (39 mg, 0,3 mmol, 1.0 equiv) and VBX-**2a** (189 mg, 0.45 mmol, 1.2 equiv) and finally *t*BuOK (34 mg, 0.3 mmol, 1.0 equiv) were allowed to react in THF (3.0 mL) for 2 h at 50 °C. The crude was purified on column chromatography (pentane:Et_2O 90:10 *v/v*) and **3y** was isolated as pink solid in 37% yield (25 mg), *E:Z* >20:1.

Mp: 44-46 °C. ^{1}H NMR (400 MHz, $CDCl_3$) δ 7.40 – 7.30 (m, 4H), 7.26 (d, *J* = 0.9 Hz, 1H), 7.14 (t, *J* = 7.8 Hz, 1H), 6.92 (d, *J* = 15.5 Hz, 1H), 6.83 (ddd, *J* = 7.7, 1.7, 0.9 Hz, 1H), 6.80 – 6.74 (m, 2H), 6.60 (ddd, *J* = 8.0, 2.3, 0.9 Hz, 1H), 3.73 (s, 2H).
^{13}C NMR (101 MHz, $CDCl_3$) δ 147.2, 136.8, 136.3, 131.8, 130.1, 128.8, 127.7, 126.2, 123.6, 119.9, 115.9, 113.9. HRMS(ESI) m/z: calcd for $C_{14}H_{13}NSH^+$ $[M+H]^+$ 228.0841; found 228.0844.

(*E*)-2-(styrylthio)aniline (3z)

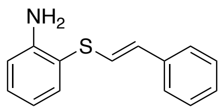

Following *General Procedure A,* 2-aminobenzenethiol (39 mg, 0,3 mmol, 1.0 equiv) and VBX-**2a** (189 mg, 0.45 mmol, 1.5 equiv) and finally *t*BuOK (34 mg, 0.3 mmol, 1.0 equiv) were allowed to react in THF (3.0 mL) for 2 h at 50 °C. The crude was purified on column chromatography (pentane:Et_2O 80:20 *v/v*) and **3z** was isolated as pink solid in 57% yield (39 mg), *E:Z* >20:1.

Mp: 55-56 °C. ^{1}H NMR (400 MHz, $CDCl_3$) δ 7.43 (dd, *J* = 7.7, 1.6 Hz, 1H), 7.32 – 7.15 (m, 6H), 6.77 (ddd, *J* = 12.9, 7.7, 1.3 Hz, 2H), 6.69 (d, *J* = 15.5 Hz, 1H), 6.37 (d, *J* = 15.4 Hz, 1H), 4.28 (s, 2H). ^{13}C NMR (101 MHz, $CDCl_3$) δ 148.3, 136.8, 136.1, 130.8, 128.7, 128.1, 127.2, 125.9, 124.1, 118.9, 115.5, 114.8. HRMS(ESI) m/z: calcd for $C_{14}H_{13}NSH^+$ $[M+H]^+$ 228.0841; found 228.0849.

((*S*)-2-methyl-3-(((*E*)-styryl)thio)propanoyl)-*L*-proline (3aa)

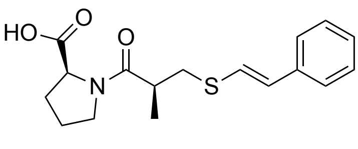

Following *General Procedure A, N*-[(*S*)-3-Mercapto-2-methylpropionyl]-L-proline (22 mg, 0,1 mmol, 1.0 equiv) and VBX **2a** (39 mg, 0.11 mmol, 1.1 equiv) and finally *t*BuOK (22 mg, 0.2 mmol, 2.0 equiv) were allowed to react in THF (1.0 mL) for 2 h at RT. The reaction was quenched with 1.0 mL of HCl solution 1M, diluted with water (1.0 mL) and extracted with DCM (3x5.0 mL).The crude was purified on column chromatography (DCM:MeOH 95:5 + 1% AcOH) and **3aa** was isolated as white resin in 62% yield (20 mg), *E:Z* >20:1.

^{1}H NMR (400 MHz, $CDCl_3$) δ 7.34 – 7.25 (m, 4H), 7.24 – 7.18 (m, 1H), 6.69 (d, *J* = 15.6 Hz, 1H), 6.51 (d, *J* = 15.6 Hz, 1H), 4.52 (dd, *J* = 8.3, 2.6 Hz, 1H), 3.61 (ddt, *J* = 17.9, 9.9, 4.3 Hz, 2H), 3.21 (dd, *J* = 13.5, 9.5 Hz, 1H), 2.97 (ddd, *J* = 9.5, 6.9, 4.7 Hz, 1H), 2.81 (dd, *J* = 13.5, 4.7 Hz, 1H), 2.36 (dq, *J* = 9.5, 3.1 Hz, 1H), 2.07 – 1.94 (m, 2H), 1.92 – 1.80 (m, 1H), 1.28 (d, *J* = 6.8 Hz, 3H).
^{13}C NMR (101 MHz, $CDCl_3$) δ 176.6, 172.7, 136.5, 128.8, 128.2, 127.3, 125.5, 125.5, 124.6, 60.0, 39.5, 36.2, 27.4, 24.7, 17.7. HRMS(ESI) m/z: calcd for $C_{17}H_{21}NO_3SH^-$ [M-H]$^-$ 318.1166; found 318.1159.

(2*R*,3*R*,4*S*,5*R*,6*S*)-2-(acetoxymethyl)-6-(((*E*)-styryl)thio)tetrahydro-2*H*-pyran-3,4,5-triyl triacetate (3ab)

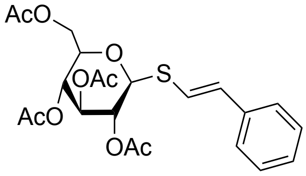

Following *General Procedure A,* 1-thio-β-d-glucose tetraacetate (36 mg, 0,1 mmol, 1.0 equiv) and VBX **2a** (39 mg, 0.11 mmol, 1.1 equiv) and finally *t*BuOK (11 mg, 0.3 mmol, 1.0 equiv) were allowed to react in THF (1.0 mL) for 2 h at RT. The crude was purified on column chromatography (pentane:Et_2O 50:50 *v/v*) and **3ab** was isolated as white solid in 71% yield (32 mg), *E:Z* >20:1.

Mp:110-112 °C.^{1}H NMR (400 MHz, $CDCl_3$) δ 7.43 – 7.22 (m, 5H), 6.75 (s, 2H), 5.33 – 5.23 (m, 1H), 5.12 (ddd, *J* = 10.3, 9.4, 8.3 Hz, 2H), 4.65 (d, *J* = 10.0 Hz, 1H), 4.26 (dd, *J* = 12.4, 4.9 Hz, 1H), 4.17 (dd, *J* = 12.4, 2.4 Hz, 1H), 3.78 (ddd, *J* = 10.1, 4.9, 2.3 Hz, 1H), 2.07 (s, 3H), 2.06 (s, 3H), 2.03 (s, 3H), 2.00 (s, 3H). *To control the E:Z ratio the NMR had to be performed in deuterated benzene*. ^{1}H NMR (400 MHz, C_6Cl_6) δ 7.12 (m 2H), 7.08 – 6.94 (m, 3H), 6.82 (d, *J* = 15.5 Hz, 1H), 6.72 (d, *J* = 15.5 Hz, 1H), 5.38 (dd, *J* = 6.9, 2.7 Hz, 2H), 5.21 (ddd, *J* = 9.7, 6.8, 2.7 Hz, 1H), 4.27 (dd, *J* = 7.0, 2.8 Hz, 1H), 4.18 (dd, *J* = 12.4, 4.9 Hz, 1H), 4.01 (dd, *J* = 12.4, 2.2 Hz, 1H), 3.21 (dt, *J* = 7.9, 2.6 Hz, 1H), 1.75 (s, 3H), 1.68 (s, 3H), 1.65 (s, 3H), 1.63 (s, 3H).
^{13}C NMR (101 MHz, $CDCl_3$) δ 170.6, 170.2, 169.4, 169.2, 141.7, 136.2, 134.8, 128.6, 126.2, 118.2, 83.6, 76.1, 73.8, 69.9, 68.1, 62.0, 20.67, 20.64, 20.56, 20.53. HRMS(ESI) m/z: calcd for $C_{22}H_{26}O_9SNa^+$ [M+Na]$^+$ 489.1190; found 489.1198. The analytical data are in accordance with literature values.[24]

1-Methyl-4-[(1*E*)-2-(phenylthio)ethenyl]benzene (3ac)

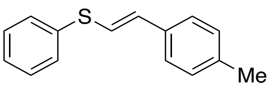

Following *General Procedure A,* thiophenol (10 μL, 0,1 mmol, 1.0 equiv) and VBX **2h** (39 mg, 0.11 mmol, 1.1 equiv) and finally *t*BuOK (11 mg, 0.1 mmol, 1.0 equiv) were allowed to react in THF (1.0 mL) for 2 h at RT. The crude was purified on column chromatography (pentane) and **3ac** was isolated as light yellow wax in 75% yield (17 mg), *E:Z* 16:1.

Major isomer (E): ^{1}H NMR (400 MHz, $CDCl_3$) δ 7.43 – 7.39 (m, 2H), 7.35 – 7.31 (m, 2H), 7.27 – 7.24 (m, 3H), 7.15 – 7.10 (m, 2H), 6.83 (d, *J* = 15.4 Hz, 1H), 6.74 (d, *J* = 15.5 Hz, 1H), 2.34 (s, 3H). Minor isomer: ^{1}H NMR (400 MHz, $CDCl_3$) δ 6.58 (d, *J* = 10.7 Hz, 1H), 6.44 (d, *J* = 10.7 Hz, 1H), 2.37 (s, 3H). ^{13}C NMR (101 MHz, $CDCl_3$) δ 137.7, 135.8, 133.9, 132.6, 129.7, 129.5, 129.3, 126.9, 126.1, 122.0, 21.4. Analytical data are in accordance with literature values.[13]

(*E*)-(2-([1,1'-biphenyl]-4-yl)vinyl)(phenyl)sulfane (3ad)

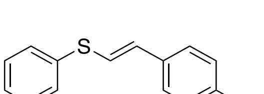

Following *General Procedure A,* thiophenol (10 μL, 0,1 mmol, 1.0 equiv) and VBX **2i** (47 mg, 0.11 mmol, 1.1 equiv) and finally *t*BuOK (11 mg, 0.1 mmol, 1.0 equiv) were allowed to react in THF (1.0 mL) for 2 h at RT. The crude was purified on column chromatography (pentane) and **3ad** was isolated as white solid in 62% yield (18 mg), *E:Z* >20:1.

Mp: 89-90 °C. ^{1}H NMR (400 MHz, $CDCl_3$) δ 7.67 – 7.55 (m, 4H), 7.48 – 7.41 (m, 6H), 7.39 – 7.33 (m, 3H), 7.31 – 7.26 (m, 1H), 6.95 (d, *J* = 15.4 Hz, 1H), 6.78 (d, *J* = 15.5 Hz, 1H). ^{13}C NMR (101 MHz, $CDCl_3$) δ 140.7, 140.5, 135.7, 135.37, 131.40, 130.1, 129.3, 129.0, 127.5, 127.2, 127.05, 126.6, 123.8. Analytical data are in accordance with literature values.[25]

(*E*)-(4-methoxystyryl)(phenyl)sulfane (3ae)

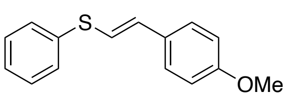

Following *General Procedure A,* thiophenol (11mg, 0.1 mmol, 1.0 equiv), VBX **2j** (39 mg, 0.11 mmol, 1.1 equiv) and finally *t*BuOK (11 mg, 0.1 mmol, 1.0 equiv) were allowed to react in THF (1.0 mL) for 2 h at RT. The crude was purified on column chromatography (pentane) and **3ae** was isolated as white solid in 51% yield (12mg), *E:Z* 4:1.

Major isomer (*E*): ^{1}H NMR (400 MHz, $CDCl_3$) δ 7.43 – 7.37 (m, 2H), 7.36 – 7.23 (m, 5H), 6.76 (d, *J* = 15.4 Hz, 1H), 6.71 (d, *J* = 15.4 Hz, 1H), 3.82 (s, 3H). Minor isomer: ^{1}H NMR (400 MHz, $CDCl_3$) δ 6.56 (d, *J* = 10.6 Hz, 1H), 6.38 (d, *J* = 10.6 Hz, 1H). ^{13}C NMR (101

MHz, $CDCl_3$) δ 159.5, 136.1. 132.9, 129.5, 129.4, 129.2, 127.5, 126.7, 120.2, 114.3, 55.5.Analytical data are in accordance with literature values[26]

(*E*)-(4-chlorostyryl)(phenyl)sulfane (3af)

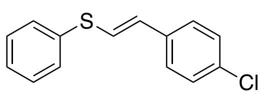

Following *General Procedure A,* thiophenol (11mg, 0.1 mmol, 1.0 equiv), VBX **2k** (42 mg, 0.11 mmol, 1.1 equiv) and finally *t*BuOK (11 mg, 0.1 mmol, 1.0 equiv) were allowed to react in THF (1.0 mL) for 2 h at RT. The crude was purified on column chromatography (pentane) and **3af** was isolated as colourless oil in 93% yield (23mg), *E:Z* >20:1.

^{1}H NMR (400 MHz, $CDCl_3$) δ 7.45 – 7.40 (m, 2H), 7.38 – 7.33 (m, 2H), 7.31 – 7.23 (m, 5H), 6.87 (d, *J* = 15.5 Hz, 1H), 6.64 (d, *J* = 15.5 Hz, 1H). ^{13}C NMR (101 MHz, $CDCl_3$) δ 135.2, 134.8, 133.3, 130.4, 129.9, 129.4, 129.0, 127.4, 127.3, 124.8. Analytical data are in accordance with literature values[16]

(*E*)-phenyl(4-(trifluoromethyl)styryl)sulfane (3ag)

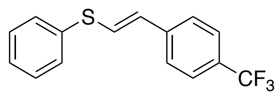

Following *General Procedure A,* benzenethiol (10 μL, 0,1 mmol, 1.0 equiv) and VBX **2l** (46 mg, 0.11 mmol, 1.1 equiv) and finally *t*BuOK (11 mg, 0.1 mmol, 1.0 equiv) were allowed to react in THF (1.0 mL) for 2 h at RT. The crude was purified on column chromatography (pentane) and **3ag** was isolated as white solid in 72% yield (20 mg), *E:Z* >20:1.

Mp: 67-68 °C. ^{1}H NMR (400 MHz, $CDCl_3$) δ 7.55 (d, *J* = 8.2 Hz, 2H), 7.48 – 7.44 (m, 2H), 7.43 – 7.28 (m, 5H), 7.02 (d, *J* = 15.5 Hz, 1H), 6.65 (d, *J* = 15.5 Hz, 1H). ^{13}C NMR (101 MHz, $CDCl_3$) δ 139.9, 134.0, 130.8, 129.5, 129.3, 129.1, 128.4, 127.7 (d, *J* = 5.3 Hz), 127.5, 125.6 (q, *J* = 3.6 Hz), 124.5 (q, *J* = 271.7 Hz). ^{19}F NMR (377 MHz, $CDCl_3$) δ – 64.47 (*J* = 271.7 Hz). HRMS(ESI) m/z: calcd for $C_{15}H_{11}F_3SH^+$ [M+H]$^+$ 281.0606; found 281.0616.

(*Z*)-*N*-(4-methoxyphenyl)-4-methyl-*N*-(1-phenyl-2-(phenylthio)vinyl)benzenesulfonamide (3ah)

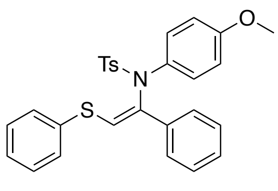

Following *General Procedure A,* thiophenol (11 mg, 0.10 mmol, 1.0 equiv), VBX **2n** (68.8 mg, 0.11 mmol, 1.1 equiv) and finally *t*BuOK (11.2 mg, 0.1 mmol, 1.0 equiv) were allowed to react in THF (1.0 mL) for 2 h at RT. The crude was purified on column chromatography (PE:EtOAc 8:2) and **3ah** was isolated as slightly yellow oil in 90 % yield (44.1 mg), *E:Z*>1:20.

^{1}H NMR (400 MHz, $CDCl_3$) δ 7.72 – 7.67 (m, 2H), 7.58 – 7.53 (m, 2H), 7.45 – 7.38 (m, 4H), 7.38 – 7.25 (m, 6H), 7.22 – 7.16 (m, 2H), 6.91 (s, 1H), 6.79 – 6.73 (m, 2H), 3.75 (s, 3H), 2.39 (s, 3H). ^{13}C NMR (101 MHz, $CDCl_3$) δ 158.6, 143.7, 138.8, 137.5, 137.5, 135.2, 132.5, 130.4, 129.4, 129.2, 128.9, 128.8, 128.6, 128.5, 128.2, 127.5, 126.8, 114.3, 55.5, 21.7. HRMS(ESI) m/z: calcd for $C_{28}H_{25}NO_3S_2Na^+$ [M+Na]$^+$; 510.1168; found 510.1251.

(*Z*)-phenyl(2-phenyl-2-(*p*-tolyloxy)vinyl)sulfane (3ai)

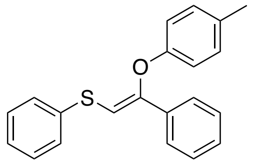

Following *General Procedure A,* thiophenol (11 mg, 0.10 mmol, 1.0 equiv), VBX **2o** (50.2 mg, 0.11 mmol, 1.1 equiv) and finally *t*BuOK (11.2 mg, 0.1 mmol, 1.0 equiv) were allowed to react in THF (1.0 mL) for 2 h at RT. The crude was purified on column chromatography (PE:EtOAc 8:2) and **3ai** was isolated as slightly yellow oil in 97% yield (30.9 mg), *E:Z*>1:6. *N.B: This reaction is sensitive to longer reaction time giving decomposition of the desired product, as well as the purification should be done in a short column.*

^{1}H NMR (400 MHz, $CDCl_3$) δ 7.52 – 7.43 (m, 4H), 7.37 – 7.23 (m, 6H), 7.09 – 7.04 (m, 2H), 6.95 – 6.91 (m, 2H), 6.64 (s, 1H). ^{13}C NMR (101 MHz, $CDCl_3$) δ 154.5, 148.6, 135.6, 134.5, 131.5, 130.2, 129.9, 129.3, 128.8, 128.3, 127.0, 125.1, 115.6, 113.2, 20.7. HRMS(ESI) m/z: calcd for $C_{21}H_{18}OSNa^+$ [M+Na]$^+$; 341.0971; found 341.0988.

(*Z*)-N-(4-methoxyphenyl)-4-methyl-N-(2-(phenylthio)vinyl)benzenesulfonamide (3aj)

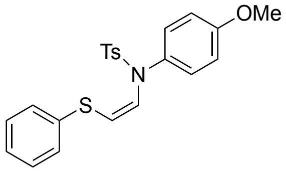

Following *General Procedure A,* thiophenol (11 mg, 0.10 mmol, 1.0 equiv), Me_2-N-zVBX **2p** (63.5 mg, 0.11 mmol, 1.1 equiv) and finally *t*BuOK (11.2 mg, 0.1 mmol, 1.0 equiv) were allowed to react in THF (1.0 mL) for 2 h at RT. The crude was purified on column chromatography (DCM:MeOH 9:1) and **3aj** was isolated as colourless oil in 59 % yield (24.4 mg), *E:Z*>1:20.

^{1}H NMR (400 MHz, $CDCl_3$) δ 7.58 – 7.51 (m, 2H), 7.32 – 7.11 (m, 7H), 7.07 – 7.01 (m, 2H), 6.92 – 6.80 (m, 3H), 5.62 (d, *J* = 8.2 Hz, 1H), 3.84 (s, 3H), 2.45 (s, 3H). ^{13}C NMR (101 MHz, $CDCl_3$) δ 159.6, 144.2, 136.5, 135.0, 131.0, 130.6, 129.7, 129.4, 129.0, 127.9, 126.7, 126.1, 114.2, 111.0, 55.6, 21.8. HRMS(ESI) m/z: calcd for $C_{22}H_{21}NO_3S_2Na^+$ [M+Na]$^+$; 434.0855; found 434.0858.

4.2 Attempted vinylation of unprotected complex structures

Unprotected Cysteine

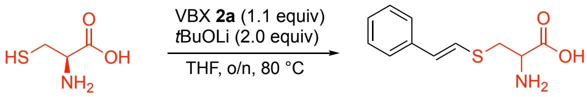

Procedure: L- cysteine (1.0 equiv, 0.1 mmol) was placed in a oven-dried microwave vial with magnetic stirring bar under argon, followed by the addition of anhydrous and degassed THF (1.0 mL). Subsequently, VBX 2a (1.1 equiv) was added followed by *t*BuOLi (2.0 equiv) and the vial was rinsed with THF (1.0 mL); then the mixture was stirred at 80 °C o/n. Afterwards, the reaction was quenched with HCl 1M (2 mL) followed by addition of DCM. The organic phase was washed with H_2O (2x2 mL) and the aqueous phase was dried at the rotavapor. The solid obtained was dissolved in MeOD and NMR yield was calculated using TMB as IS.
NMR yield corresponds to 11% and the presence of the corresponding vinylated product has been confirmed with HRMS. HRMS(ESI) m/z: calcd for $C_{11}H_{13}NO_2SNa^+$ $[M+Na]^+$: 246.0559; found 246.0546.

1-thio-beta-D-glucose

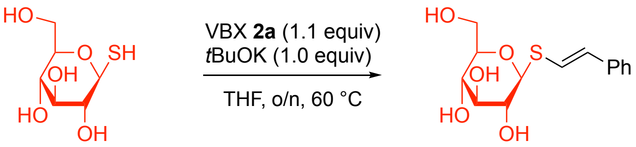

Procedure: 1-thio-beta-D-glucose (1.0 equiv, 0.1 mmol) was placed in a oven-dried microwave vial with magnetic stirring bar under argon, followed by the addition of anhydrous and degassed THF (1.0 mL). Subsequently, VBX 2a (1.1 equiv) was added followed by *t*BuOK (1.0 equiv) and the vial was rinsed with THF (1.0 mL); then the mixture was stirred at 60 °C o/n. Afterwards, the reaction was quenched with water (2 mL) followed by addition of DCM. The aqueous phase was extracted with DCM (2x10 mL) and the organic phase was dried at the rotavapor. The solid obtained was dissolved in MeOD and NMR yield was calculated using TMB as IS.
NMR yield corresponds to 18% and the presence of the corresponding vinylated product has been confirmed with HRMS. HRMS(ESI) m/z: calcd for $C_{14}H_{18}NO_5SNa^+$ $[M+Na]^+$: 321.0767; found 321.0729.

4.3 Products from Vinylation of Mercaptothiazoles

(*E*)-2-(styrylthio)thiazole (9a)

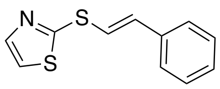

Following *General Procedure B,* thiazole-2-thiol (12 mg, 0,1 mmol, 1.0 equiv) and VBX **2a** (39 mg, 0.11 mmol, 1.1 equiv) and finally *t*BuOK (11 mg, 0.1 mmol, 1.0 equiv) were allowed to react in Tol (1.0 mL) for 2 h at 80 °C. The crude was purified on column chromatography/preparative TLC (pentane:Et_2O 90:10 *v/v*) and **9a** was isolated as colourless wax in 45% yield (9 mg), *E:Z* 10:1.

^{1}H NMR (400 MHz, $CDCl_3$) δ 7.74 (d, *J* = 3.4 Hz, 1H), 7.43 – 7.27 (m, 6H), 7.12 (d, *J* = 15.5 Hz, 1H), 6.97 (d, *J* = 15.5 Hz, 1H). ^{13}C NMR (101 MHz, $CDCl_3$) δ 163.9, 143.5, 135.9, 135.6, 128.9, 128.6, 126.7, 119.9, 119.2. HRMS(ESI) m/z: calcd for $C_{11}H_9NSH^+$ $[M+H]^+$ 220.0249; found 220.0255.

(*E*)-5-phenyl-2-(styrylthio)thiazole (9b)

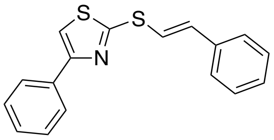

Following *General Procedure B,* 3-phenylmercapto-2-thiol (17 mg, 0,1 mmol, 1.0 equiv) and VBX **2a** (39 mg, 0.11 mmol, 1.1 equiv) and finally *t*BuOK (11 mg, 0.1 mmol, 1.0 equiv) were allowed to react in Tol (1.0 mL) for 2 h at 80 °C. The crude was purified on column chromatography/preparative TLC (pentane:Et_2O 90:10 *v/v*) and **9c** was isolated as pale yellow solid in 44% yield (13 mg), *E:Z* 10:1.

Mp: 95-97 °C. ^{1}H NMR (400 MHz, $CDCl_3$) δ 7.90 (dd, *J* = 8.3, 1.3 Hz, 2H), 7.48 – 7.28 (m, 9H), 7.17 (d, *J* = 15.5 Hz, 1H), 7.01 (d, *J* = 15.5 Hz, 1H). ^{13}C NMR (101 MHz, $CDCl_3$) δ 163.8, 156.4, 136.0, 135.8, 134.1, 129.0, 128.9, 128.6, 128.5, 126.8, 126.5, 119.1, 113.4. HRMS(ESI) m/z: calcd for $C_{17}H_{13}NS_2H^+$ $[M+H]^+$ 296.0562; found 296.0561.

(*E*)-2-(styrylthio)benzo[*d*]thiazole (9c)

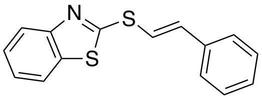

Following *General Procedure B,* benzo[*d*]thiazole-2-thiol (17 mg, 0,1 mmol, 1.0 equiv) and VBX **2a** (39 mg, 0.11 mmol, 1.1 equiv) and finally *t*BuOK (11 mg, 0.1 mmol, 1.0 equiv) were allowed to react in Tol (1.0 mL) for 2 h at 80 °C. The crude was purified on column chromatography/preparative TLC (pentane:Et_2O 90:10 *v/v*) and **9b** was isolated as pale yellow solid in 47% yield (13 mg), *E:Z* 10:1.

Mp:105-107 °C. ^{1}H NMR (400 MHz, $CDCl_3$) δ 7.93 (ddd, *J* = 8.1, 1.2, 0.6 Hz, 1H), 7.78 (ddd, *J* = 7.9, 1.3, 0.6 Hz, 1H), 7.51 – 7.29 (m, 8H), 7.06 (d, *J* = 15.5 Hz, 1H). ^{13}C NMR (101 MHz, $CDCl_3$) δ 166.2, 153.8, 137.2, 135.8, 135.5, 129.0, 128.9, 126.9, 126.4, 124.6, 122.1, 121.3, 117.6. HRMS(ESI) m/z: calcd for $C_{15}H_{11}NS_2H^+$ $[M+H]^+$270.0406; found 270.0415.

(*E*)-2-(styrylthio)benzo[*d*]oxazole (9d)

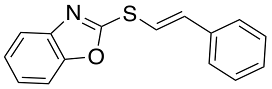

Following *General Procedure B,* benzo[*d*]oxazole-2-thiol (17 mg, 0,1 mmol, 1.0 equiv) and VBX **2a** (39 mg, 0.11 mmol, 1.1 equiv) and finally *t*BuOK (11 mg, 0.1 mmol, 1.0 equiv) were allowed to react in Tol (1.0 mL) for 2 h at 80 °C. The crude was purified on column chromatography/preparative TLC (pentane:Et_2O 90:10 *v/v*) and **9d** was isolated as pale yellow wax in 21% yield (5 mg), *E:Z* 10:1.

^{1}H NMR (400 MHz, $CDCl_3$) δ 7.67 – 7.62 (m, 1H), 7.50 – 7.43 (m, 3H), 7.42 – 7.34 (m, 2H), 7.34 – 7.27 (m, 4H), 6.99 (d, *J* = 15.9 Hz, 1H). ^{13}C NMR (101 MHz, $CDCl_3$) δ 162.9, 152.1, 142.1, 135.8, 135.5, 128.9, 128.7, 126.8, 124.7, 124.4, 118.9, 115.7, 110.2. HRMS(ESI) m/z: calcd for $C_{15}H_{11}NOSH^+$ [M+H]$^+$; 254.0642; found 254.0634

5 References

[1] B. S. A. I. F. Vogel, A. J. Hannaford, V. Rogers, P. W. G. Smith, A. Tatchell, R., *Vogel's Textbook of Practical Organic Chemistry, 4th ed.*, **1978**.
[2] E. Stridfeldt, A. Seemann, M. J. Bouma, C. Dey, A. Ertan, B. Olofsson, *Chem. Eur. J.* **2016**, *22*, 16066-16070.
[3] A. Boelke, L. D. Caspers, B. J. Nachtsheim, *Org. Lett.* **2017**, *19*, 5344-5347.
[4] P. Caramenti, N. Declas, R. Tessier, M. D. Wodrich, J. Waser, *Chem. Sci.* **2019**, *10*, 3223-3230.
[5] D. Fernández González, J. P. Brand, R. Mondière, J. Waser, *Adv. Synth. Catal.* **2013**, *355*, 1631-1639.
[6] B. Lu, J. Wu, N. Yoshikai, *Journal of the American Chemical Society* **2014**, *136*, 11598-11601.
[7] S. Bertho, R. Rey-Rodriguez, C. Colas, P. Retailleau, I. Gillaizeau, *Chem. Eur. J.* **2017**, *23*, 17674-17677.
[8] J. P. Brand, C. Chevalley, R. Scopelliti, J. Waser, *Chem. Eur. J.* **2012**, *18*, 5655-5666.
[9] M. J. Bouma, B. Olofsson, *Chem. Eur. J.* **2012**, *18*, 14242-14245.
[10] P. Villo, G. Kervefors, B. Olofsson, *Chem. Commun.* **2018**, *54*, 8810-8813.
[11] a) A. Ozanne-Beaudenon, S. Quideau, *Angew. Chem. Int. Ed.* **2005**, *44*, 7065-7069; b) N. Lucchetti, M. Scalone, S. Fantasia, K. Muñiz, *Angew. Chem. Int. Ed.* **2016**, *55*, 13335-13339; c) G. Kervefors, A. Becker, C. Dey, B. Olofsson, *Beilstein J. Org. Chem.* **2018**, *14*, 1491-1497.
[12] C. G. Bates, P. Saejueng, M. Q. Doherty, D. Venkataraman, *Org. Lett.* **2004**, *6*, 5005-5008.
[13] Y. Zheng, X. Du, W. Bao, *Tetrahedron Lett.* **2006**, *47*, 1217-1220.
[14] Y. Yang, R. M. Rioux, *Chem. Commun.* **2011**, *47*, 6557-6559.
[15] R. Singh, D. S. Raghuvanshi, K. N. Singh, *Org. Lett.* **2013**, *15*, 4202-4205.
[16] H.-Y. Tu, B.-L. Hu, C.-L. Deng, X.-G. Zhang, *Chem. Commun.* **2015**, *51*, 15558-15561.
[17] A. Kövér, O. Boutureira, M. I. Matheu, Y. Díaz, S. Castillón, *J. Org. Chem.* **2014**, *79*, 3060-3068.
[18] K. Choudhuri, M. Pramanik, A. Mandal, P. Mal, *Asian J. Org. Chem.* **2018**, *7*, 1849-1855.
[19] S. Kobayashi, H. Kuroda, Y. Ohtsuka, T. Kashihara, A. Masuyama, K. Watanabe, *Tetrahedron* **2013**, *69*, 2251-2259.
[20] A. A. Heredia, A. B. Peñéñory, *Eur. J. Org. Chem.* **2013**, *2013*, 991-997.
[21] L. Benati, L. Capella, P. C. Montevecchi, P. Spagnolo, *J. Org. Chem.* **1994**, *59*, 2818-2823.
[22] Y. Liao, S. Chen, P. Jiang, H. Qi, G.-J. Deng, *Eur. J. Org. Chem.* **2013**, *2013*, 6878-6885.
[23] L. Tommaso, M. Matteo, M. Alessandro, N. Daniele, S. Piero, C. Cinzia, *Curr. Org. Chem.* **2009**, *13*, 1726-1732.
[24] B. Liu, J. V. Alegre-Requena, R. S. Paton, G. M. Miyake, *Chem. Eur. J.*, *n/a*.
[25] S. Nurhanna Riduan, J. Y. Ying, Y. Zhang, *Org. Lett.* **2012**, *14*, 1780-1783.
[26] D. G. Bachmann, C. C. Wittwer, D. G. Gillingham, *Adv. Synth. Catal.* **2013**, *355*, 3703-3707.

2b

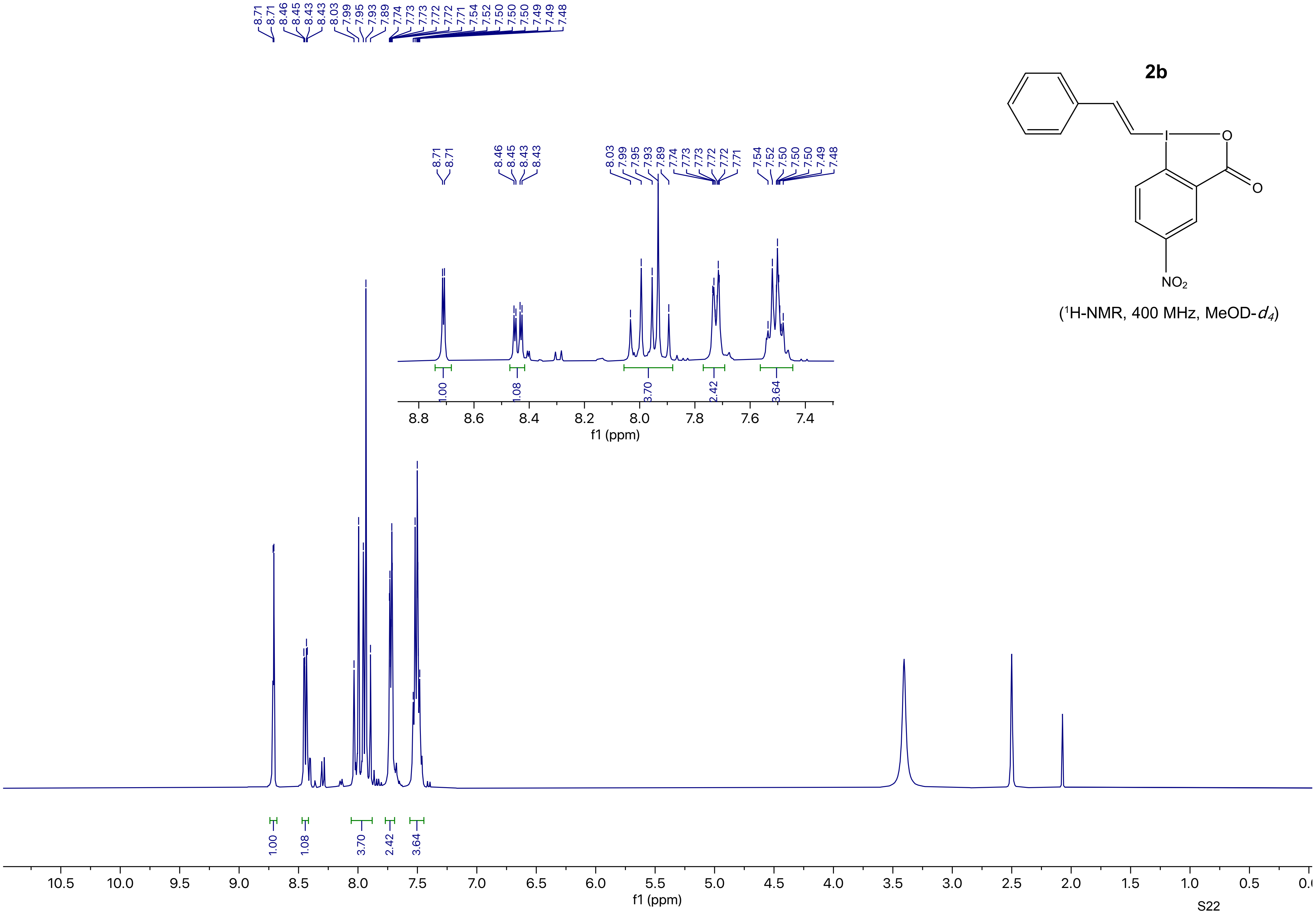

(^{1}H-NMR, 400 MHz, MeOD-d_4)

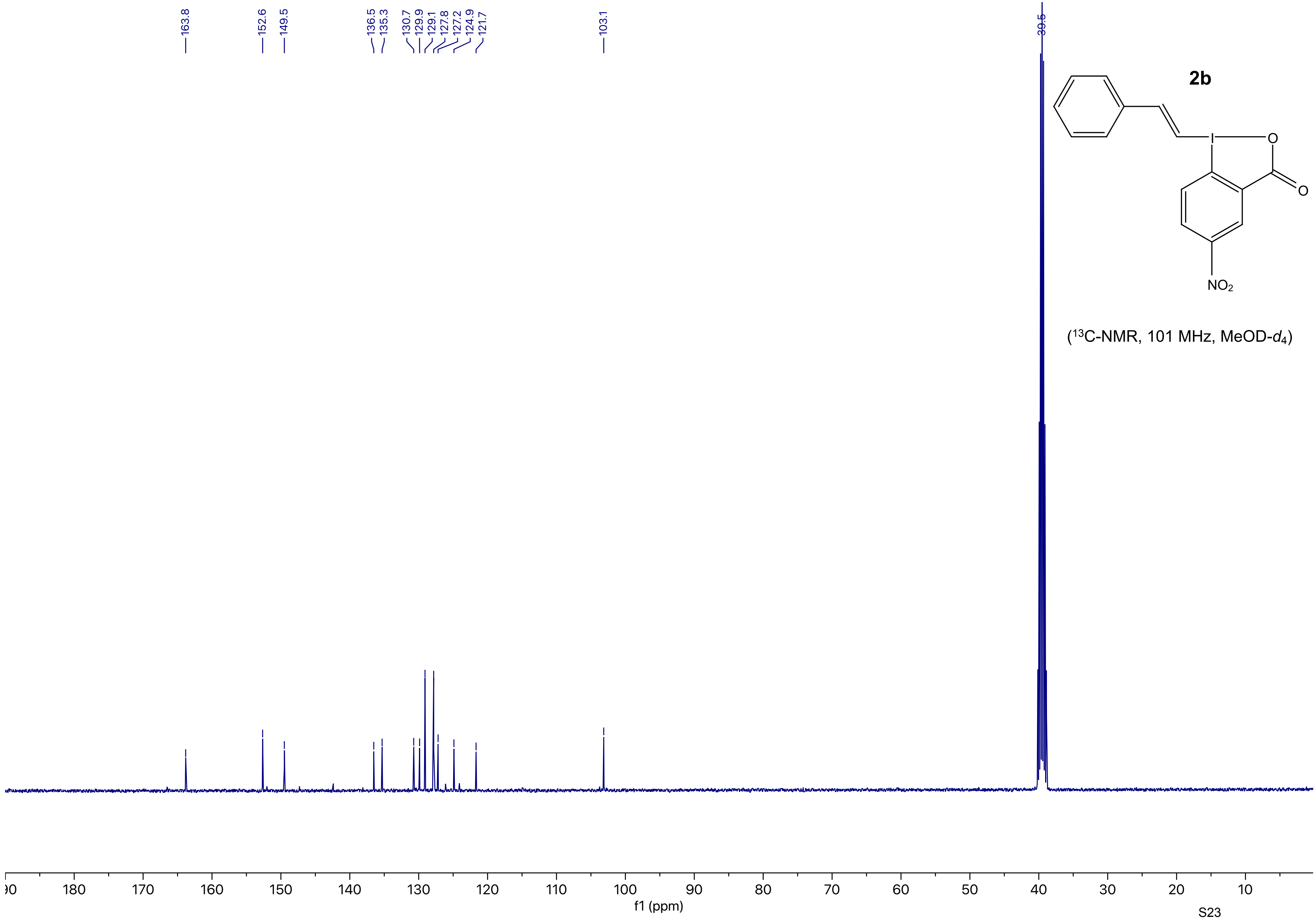
163.8
152.6
149.5
136.5
135.3
130.7
129.9
129.1
127.8
127.2
124.9
121.7
103.1
39.5
2b
O
O
I
NO2
(13C-NMR, 101 MHz, MeOD-d4)
180
170
160
150
140
130
120
110
100
90
80
70
60
50
40
30
20
10
f1 (ppm)

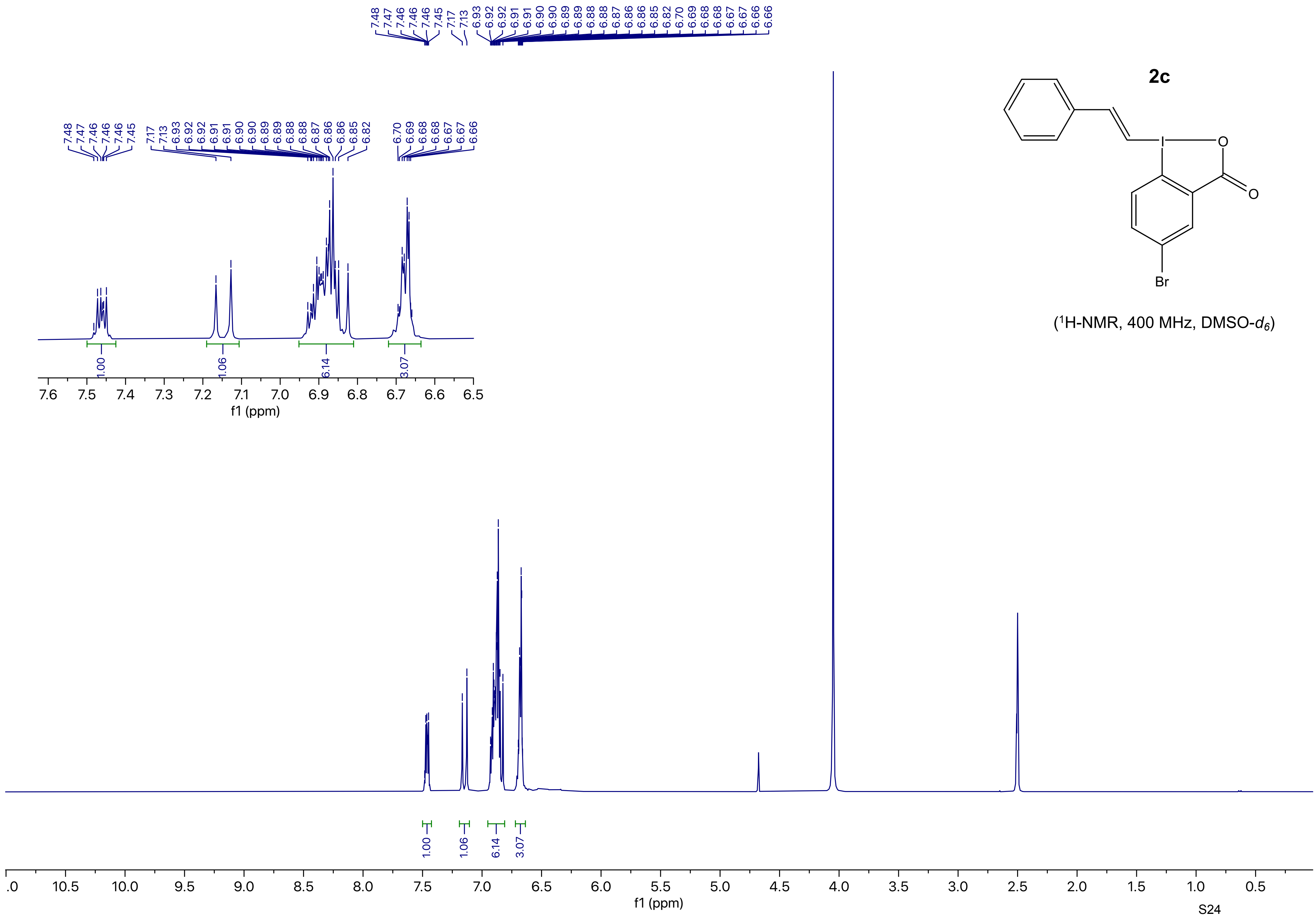

2c
Br
(¹H-NMR, 400 MHz, DMSO-d6)
7.48
7.47
7.46
7.46
7.46
7.45
7.17
7.13
6.93
6.92
6.92
6.91
6.91
6.90
6.90
6.89
6.89
6.88
6.88
6.87
6.86
6.86
6.85
6.82
6.70
6.69
6.68
6.68
6.67
6.67
6.66
1.00
1.06
6.14
3.07
f1 (ppm)

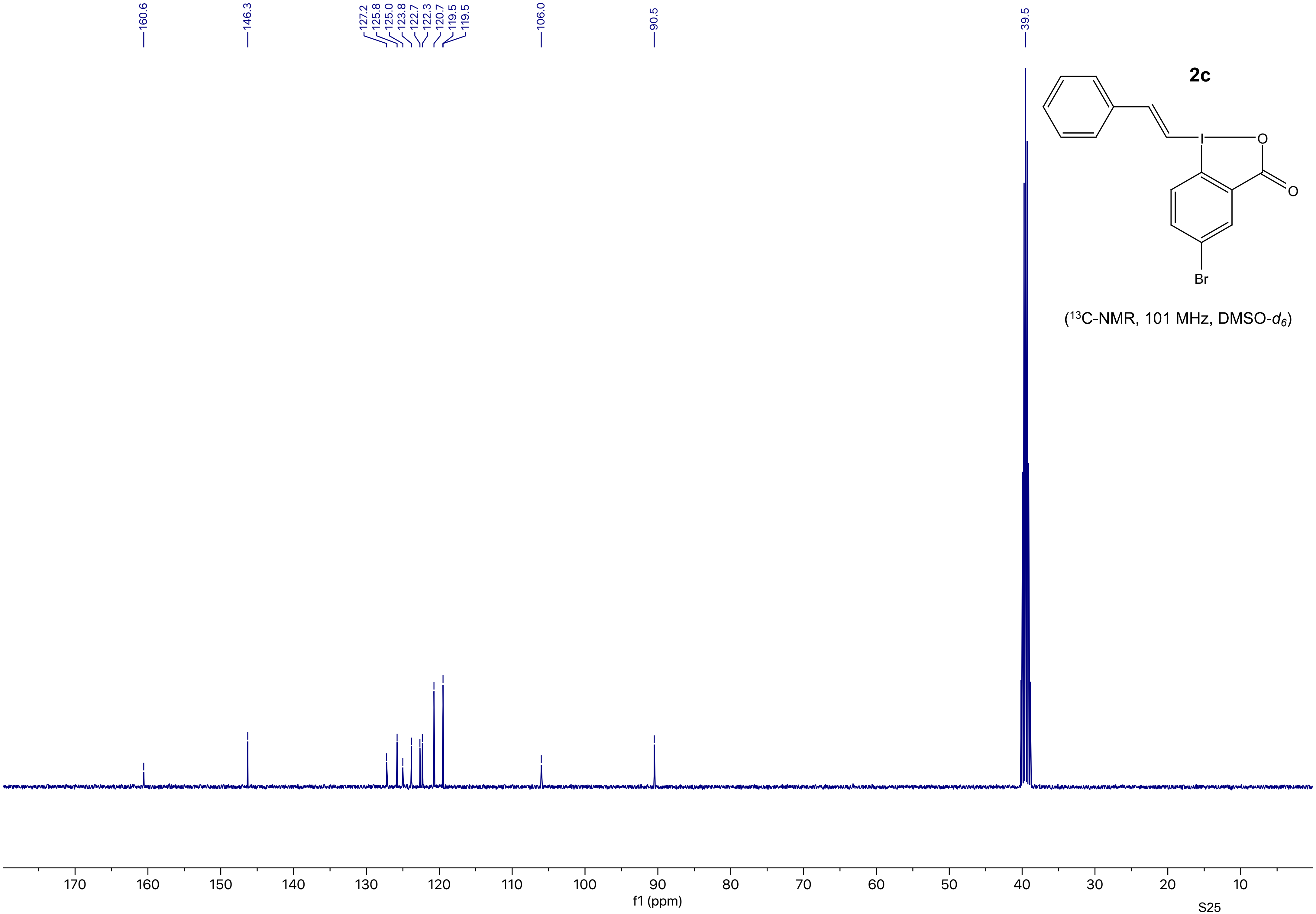

2c

(^{13}C-NMR, 101 MHz, DMSO-d_6)

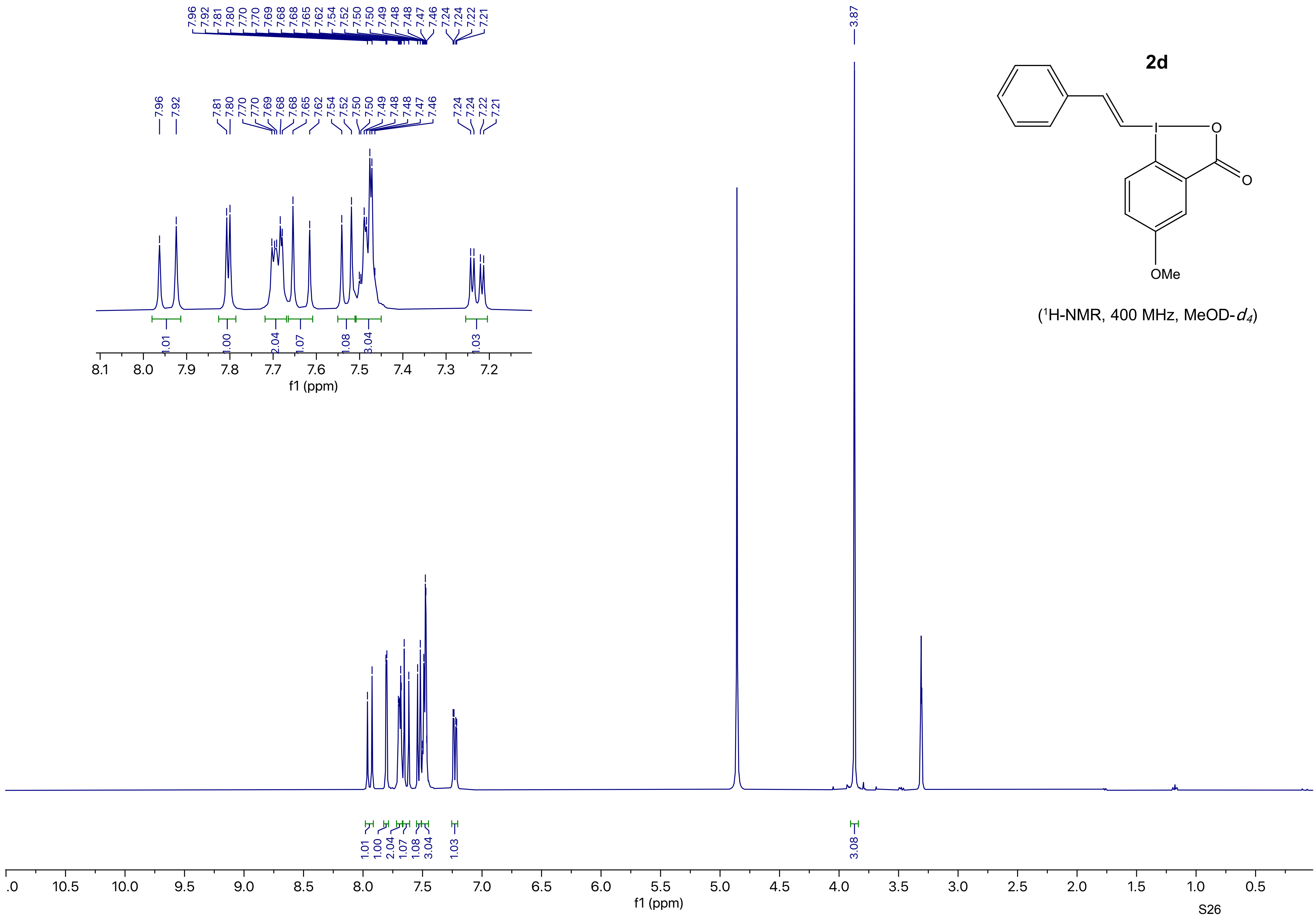

2d

(^{1}H-NMR, 400 MHz, MeOD-d_4)

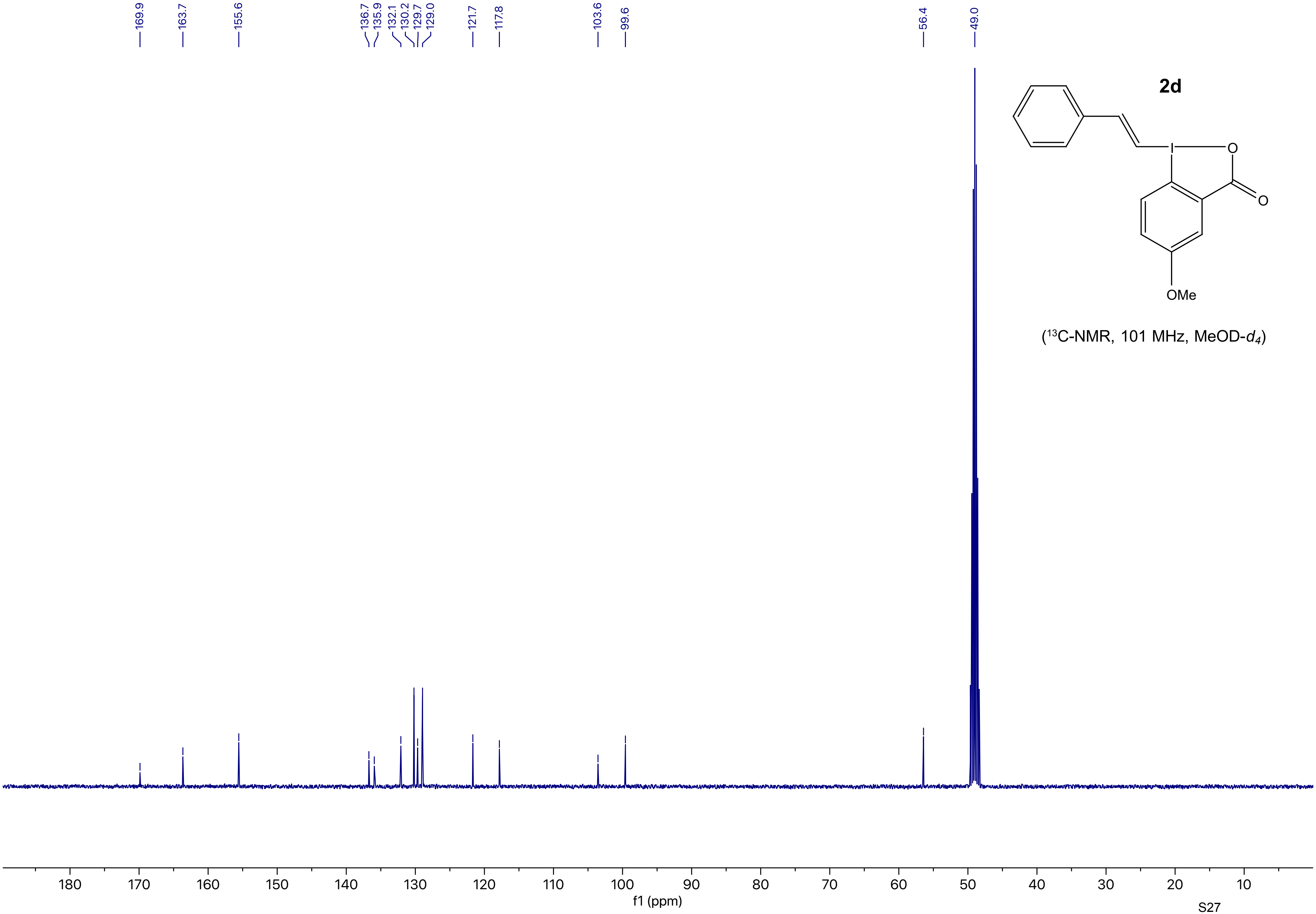

2d

(^{13}C-NMR, 101 MHz, MeOD-d_4)

2e

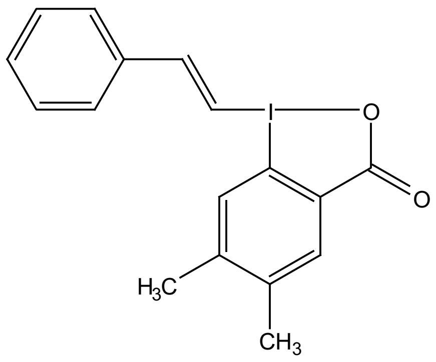

(1H-NMR, 400 MHz, MeOD-d_4)

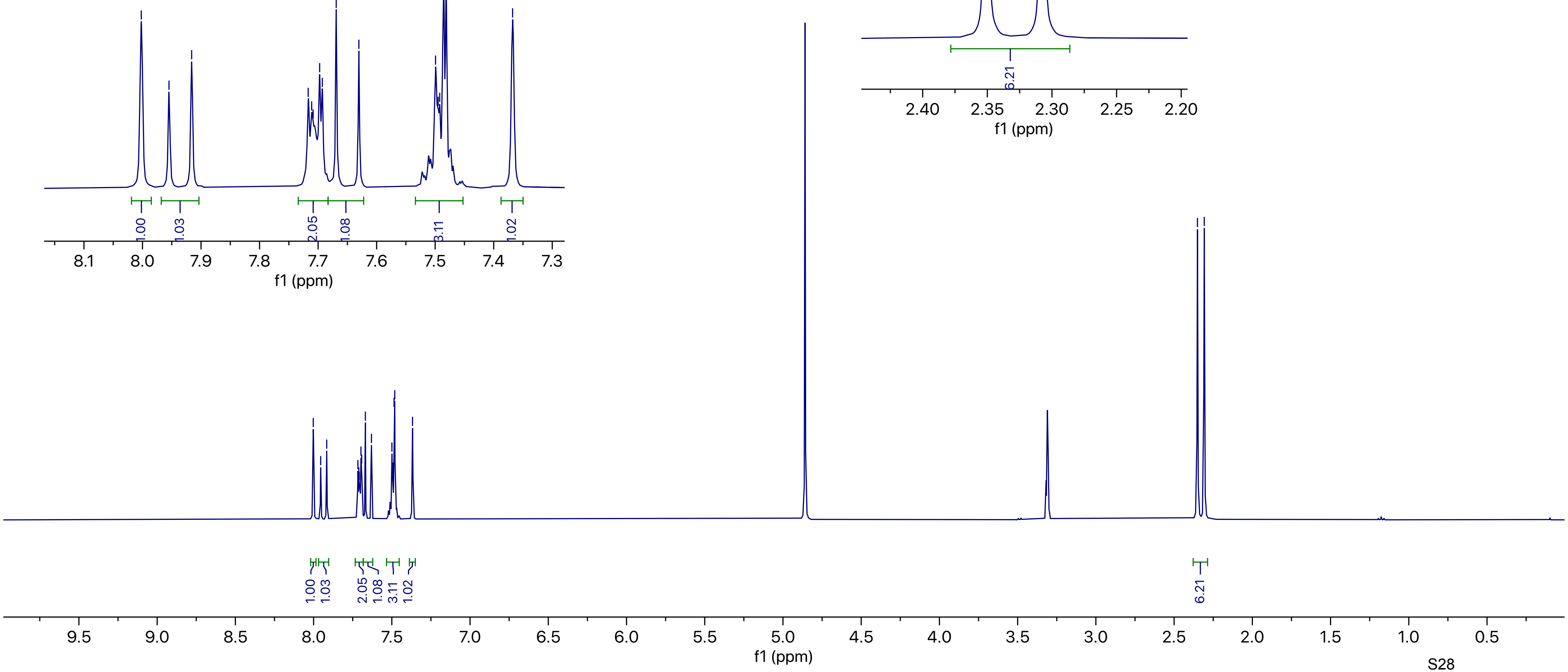

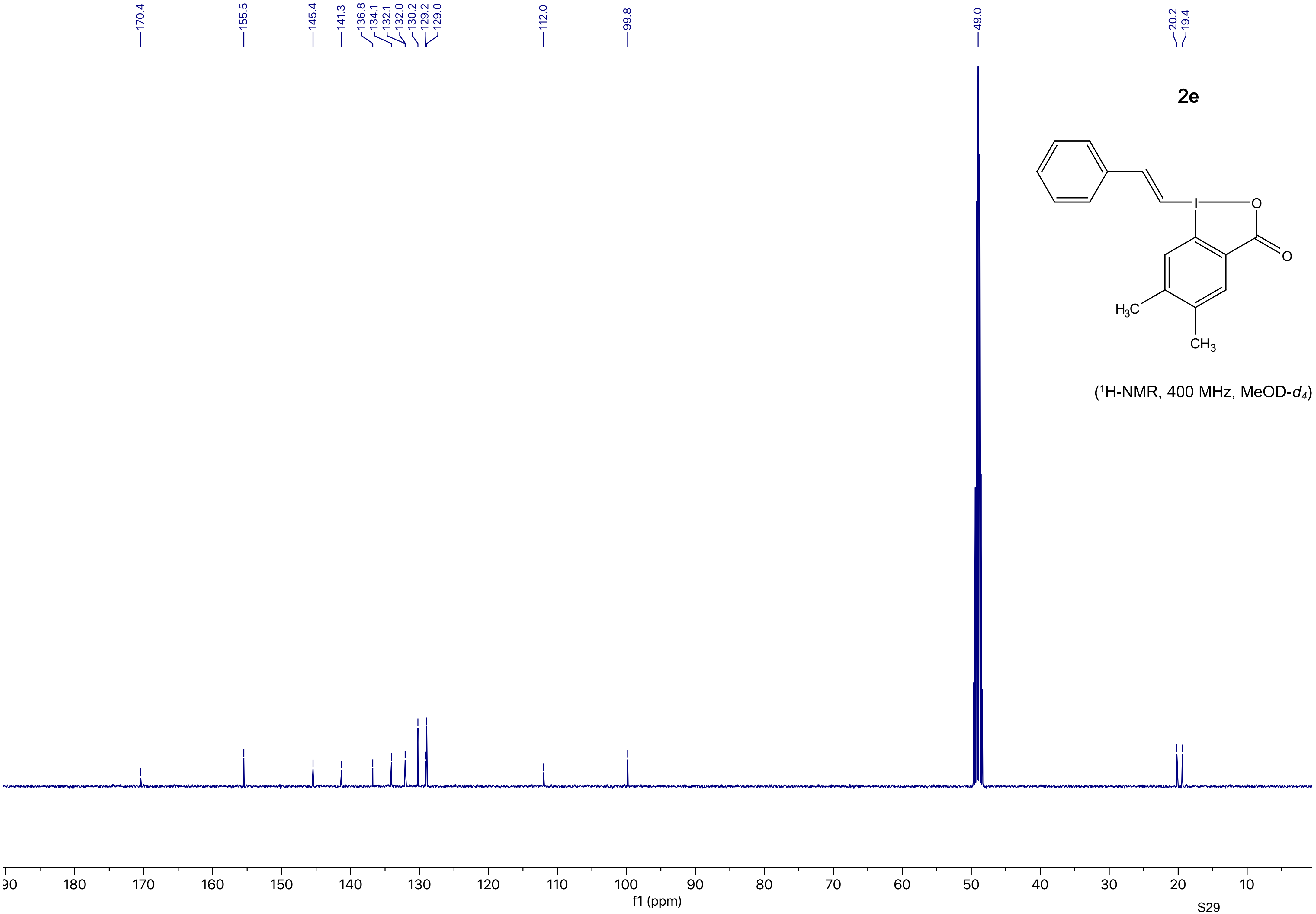

(^{1}H-NMR, 400 MHz, MeOD-d_4)

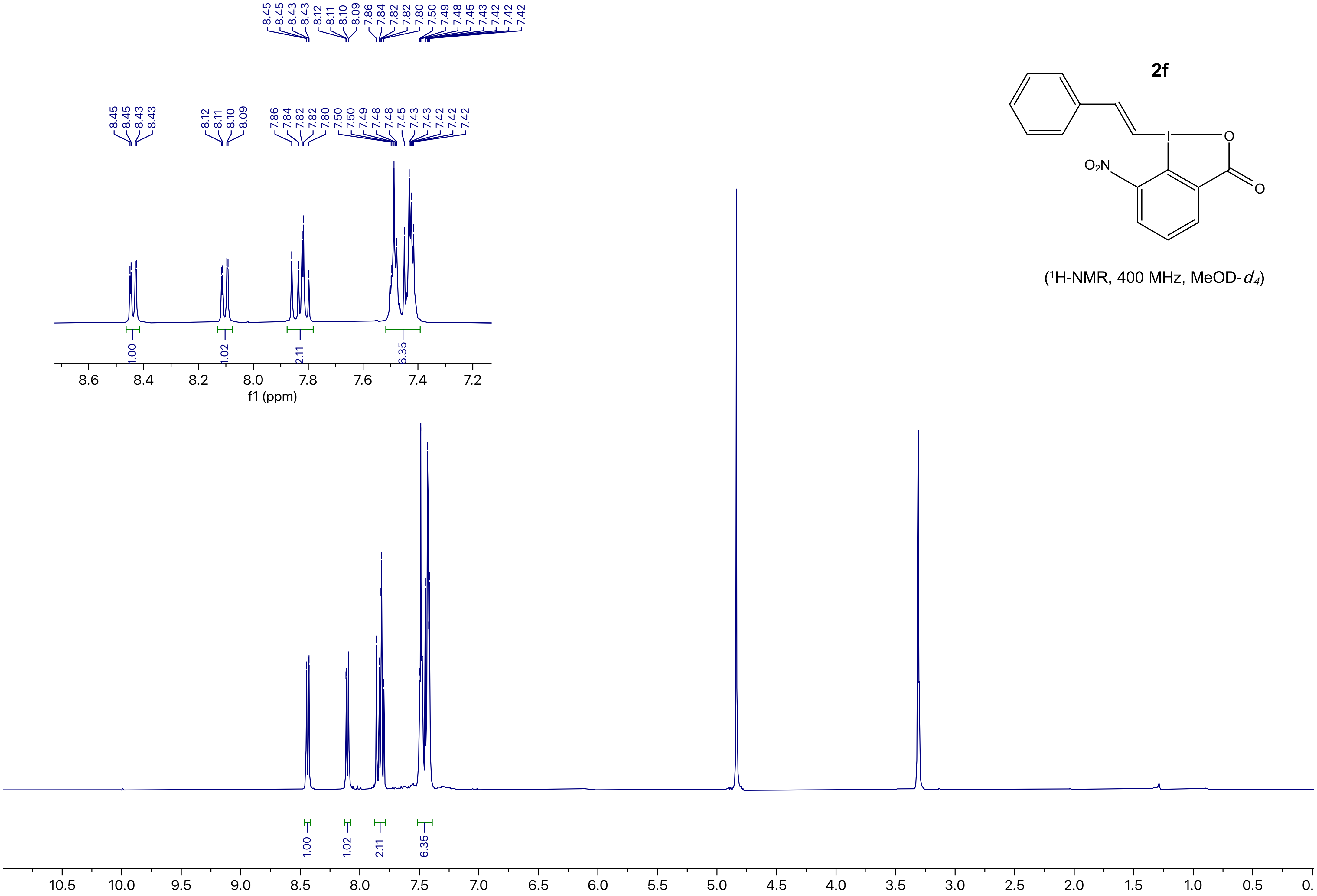

2f
O2N
I
O
O
(1H-NMR, 400 MHz, MeOD-d4)
8.45 8.45 8.43 8.43 8.12 8.11 8.10 8.09 7.86 7.84 7.82 7.82 7.80 7.50 7.49 7.48 7.45 7.43 7.42 7.42 7.42
1.00
1.02
2.11
6.35
8.6 8.4 8.2 8.0 7.8 7.6 7.4 7.2
f1 (ppm)
10.5 10.0 9.5 9.0 8.5 8.0 7.5 7.0 6.5 6.0 5.5 5.0 4.5 4.0 3.5 3.0 2.5 2.0 1.5 1.0 0.5 0.
f1 (ppm)

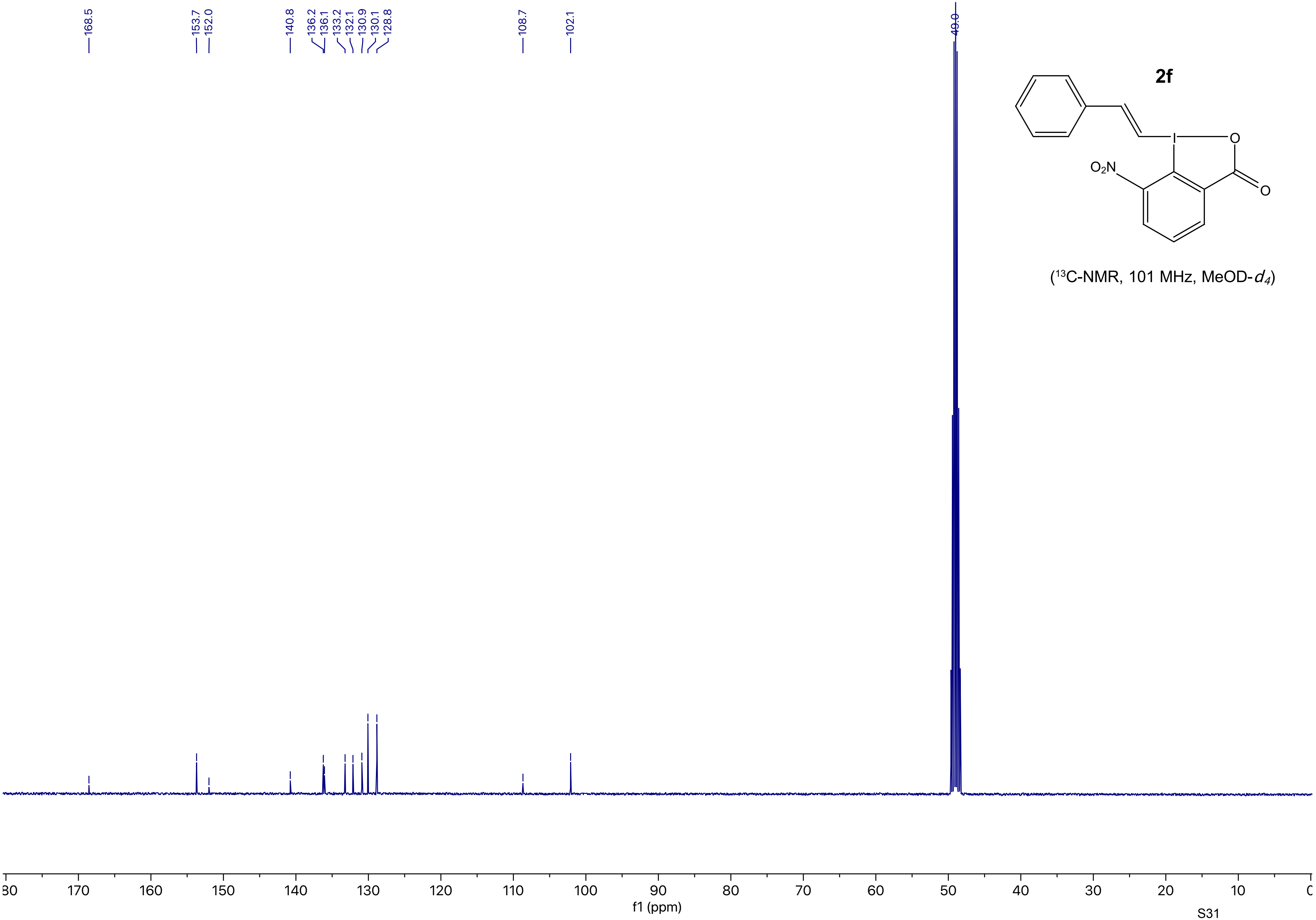

2f

(^{13}C-NMR, 101 MHz, MeOD-d_4)

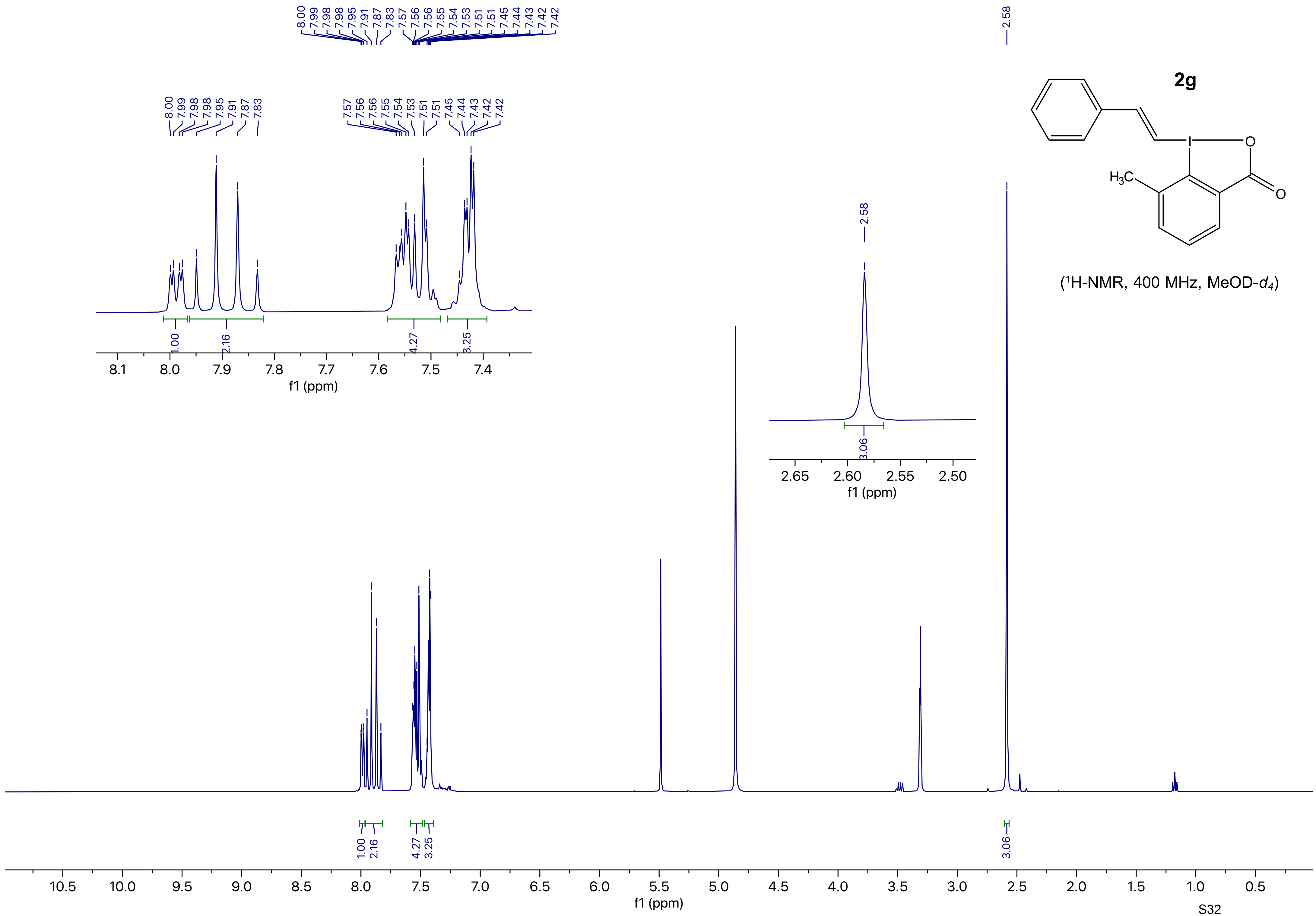

2g
H3C
I
O
O
(1H-NMR, 400 MHz, MeOD-d4)
8.00 7.99 7.98 7.98 7.95 7.91 7.87 7.83 7.57 7.56 7.56 7.55 7.54 7.53 7.51 7.51 7.45 7.44 7.43 7.42 7.42
2.58
1.00 2.16 4.27 3.25 3.06
f1 (ppm)

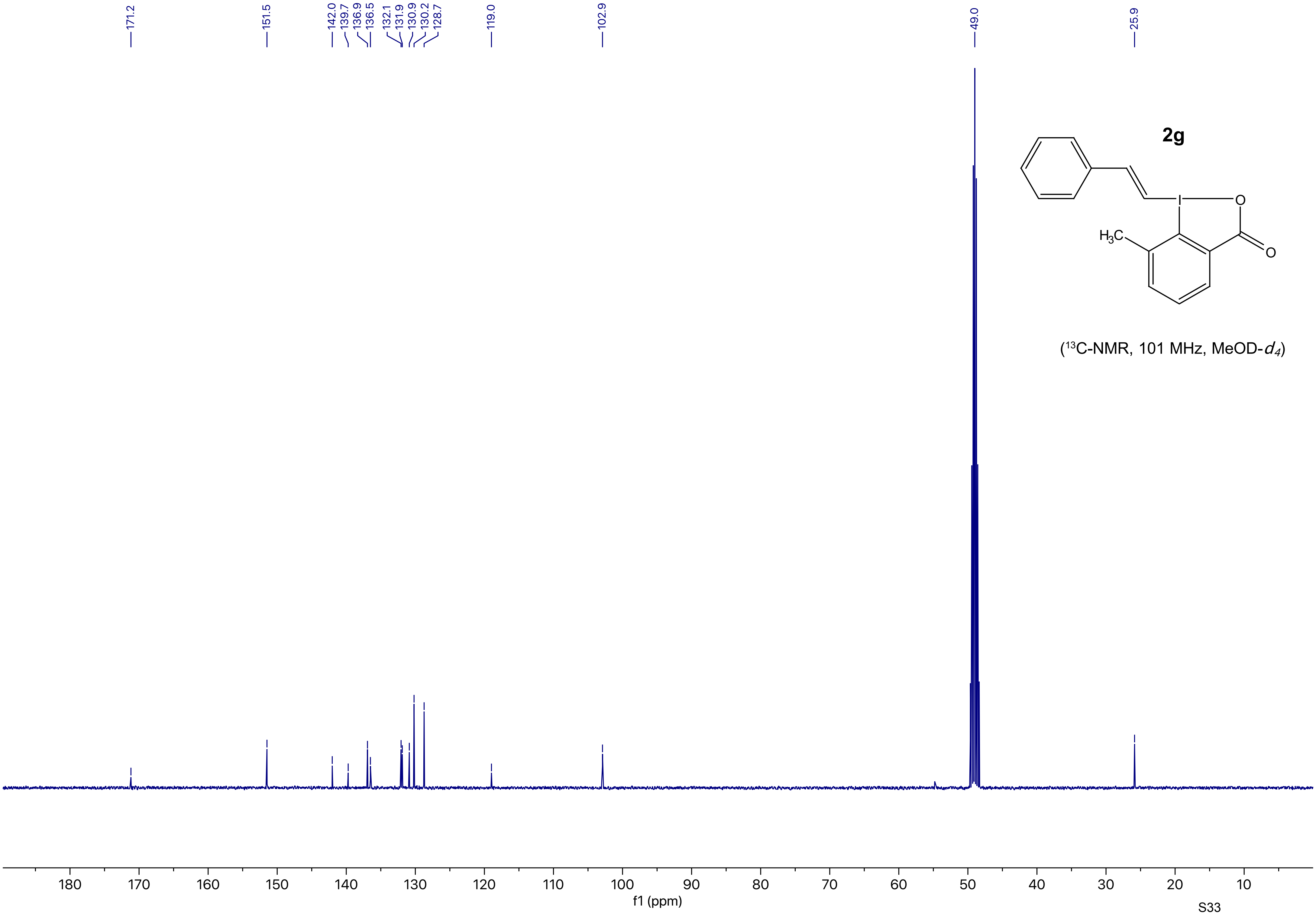

2g

(^{13}C-NMR, 101 MHz, MeOD-d_4)

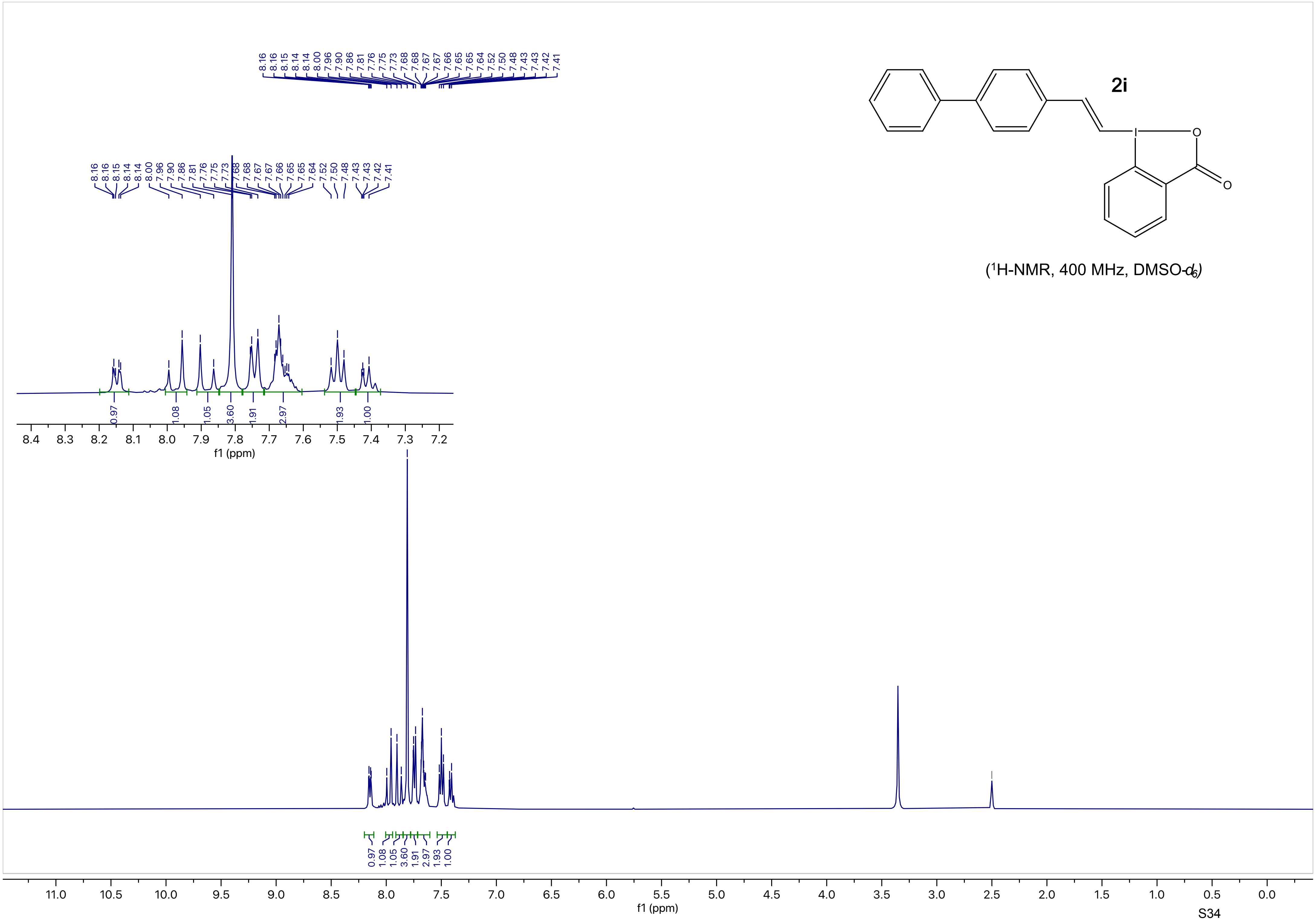

2i

(^{1}H-NMR, 400 MHz, DMSO-d_6)

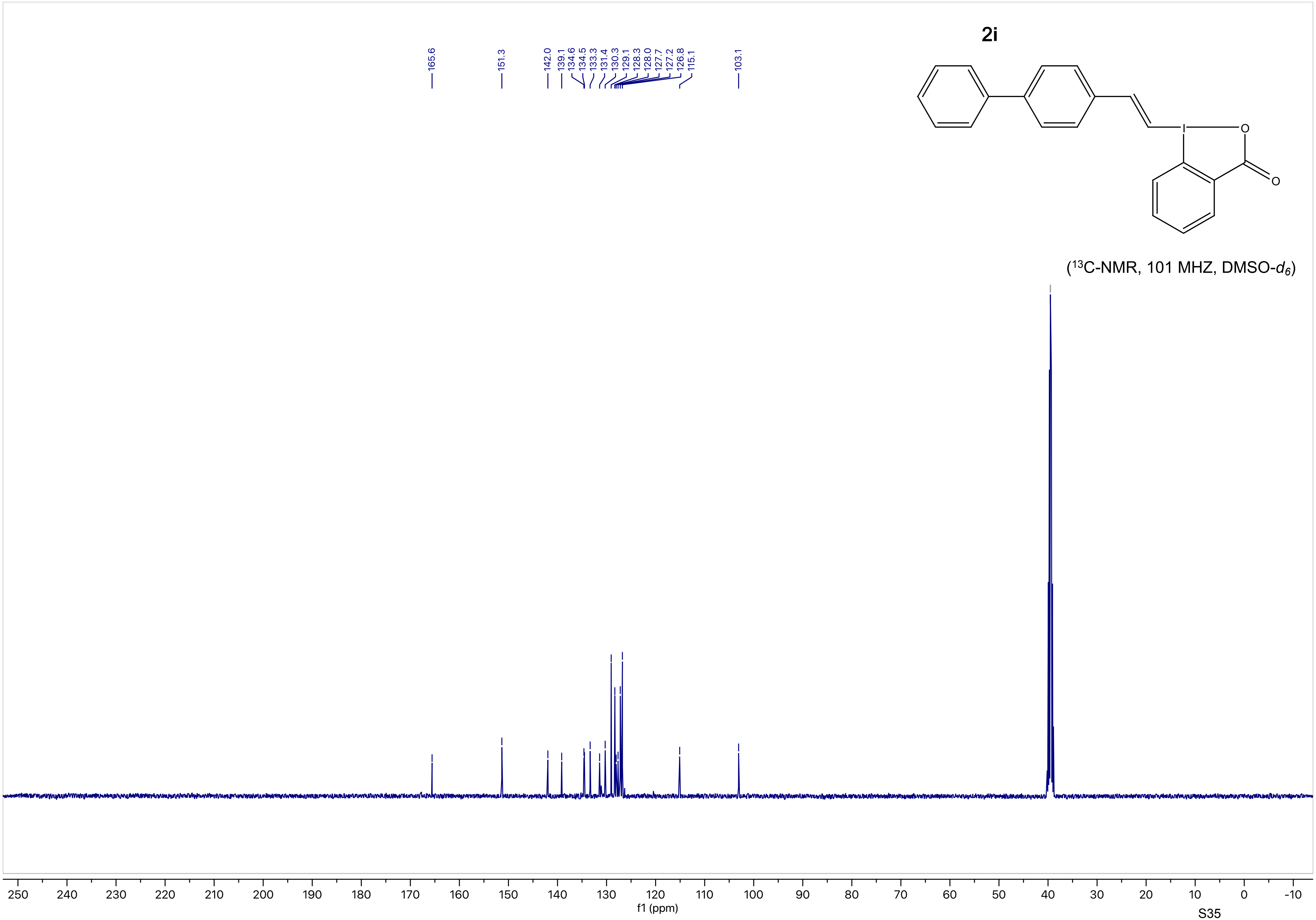

2i

(^{13}C-NMR, 101 MHZ, DMSO-d_6)

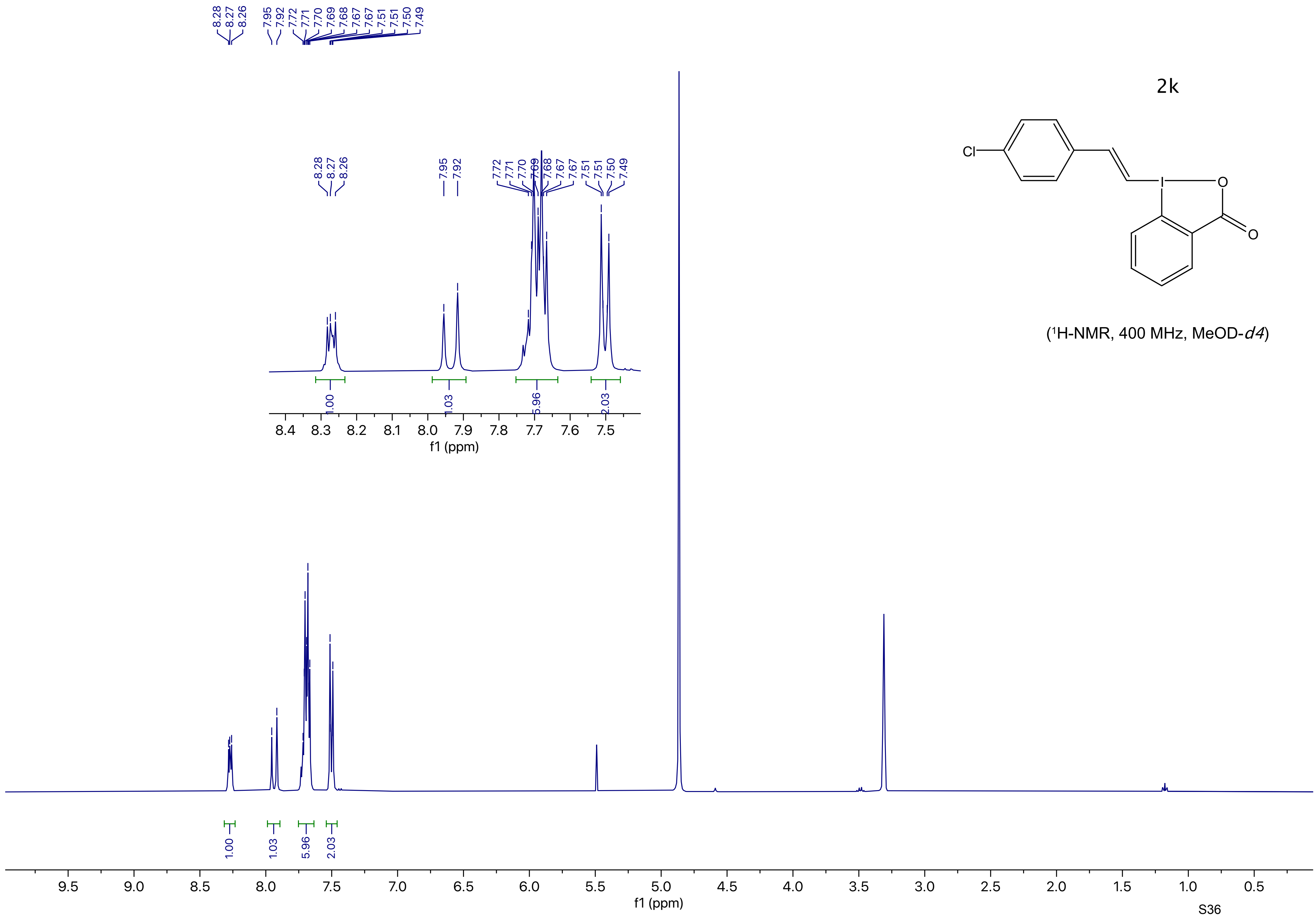
2k
Cl
I
O
O
(¹H-NMR, 400 MHz, MeOD-d4)
8.28
8.27
8.26
7.95
7.92
7.72
7.71
7.70
7.69
7.68
7.67
7.67
7.51
7.51
7.50
7.49
1.00
1.03
5.96
2.03
f1 (ppm)

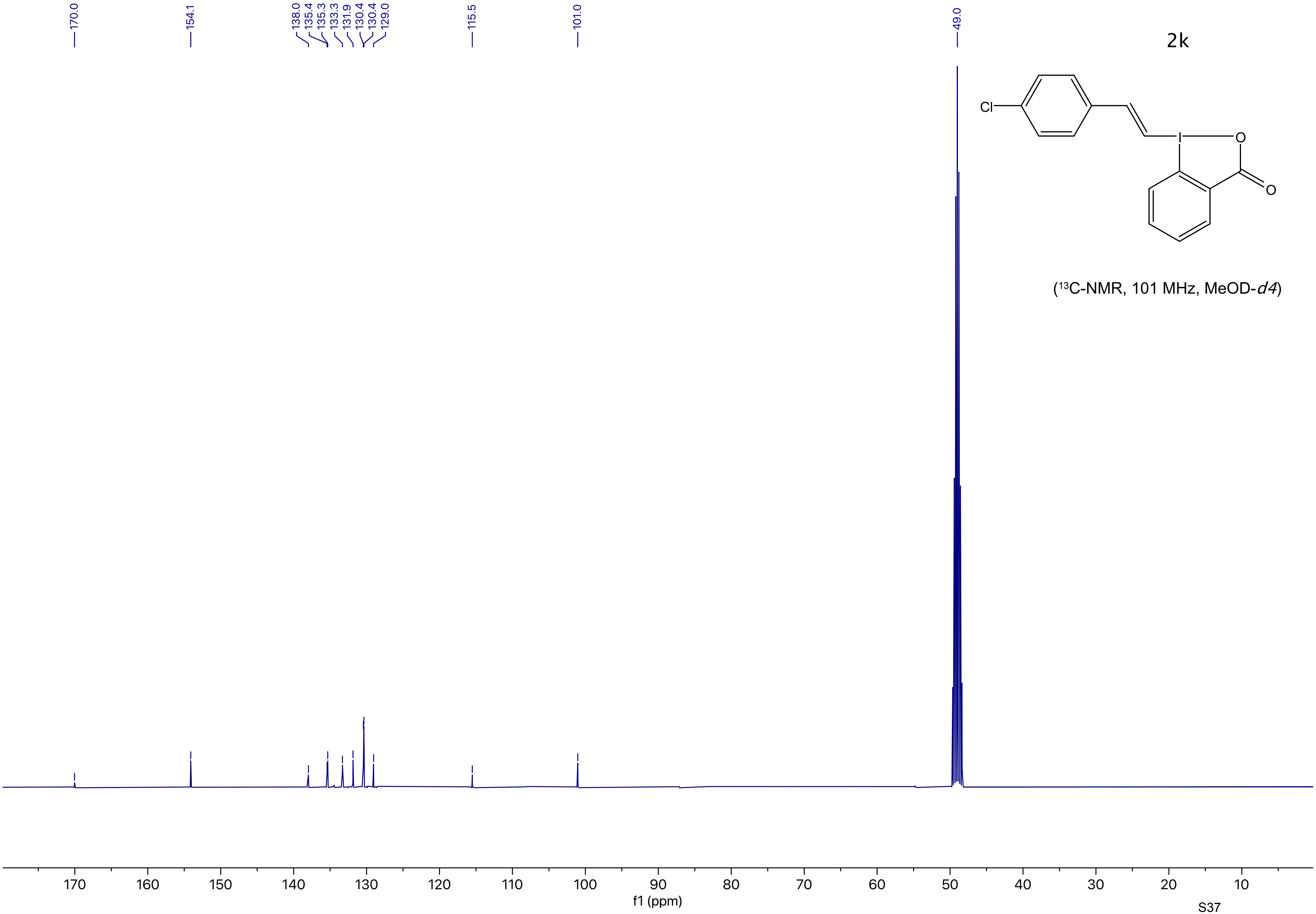

2k

(^{13}C-NMR, 101 MHz, MeOD-*d4*)

2n

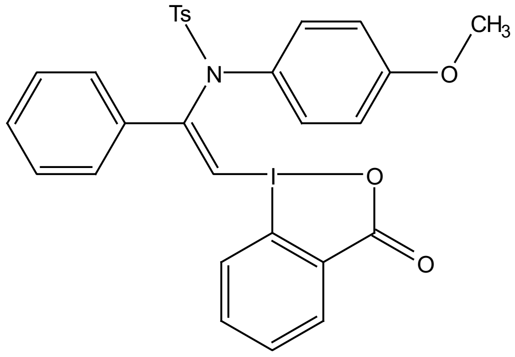

(¹H-NMR, 400 MHz, $CDCl_3$)

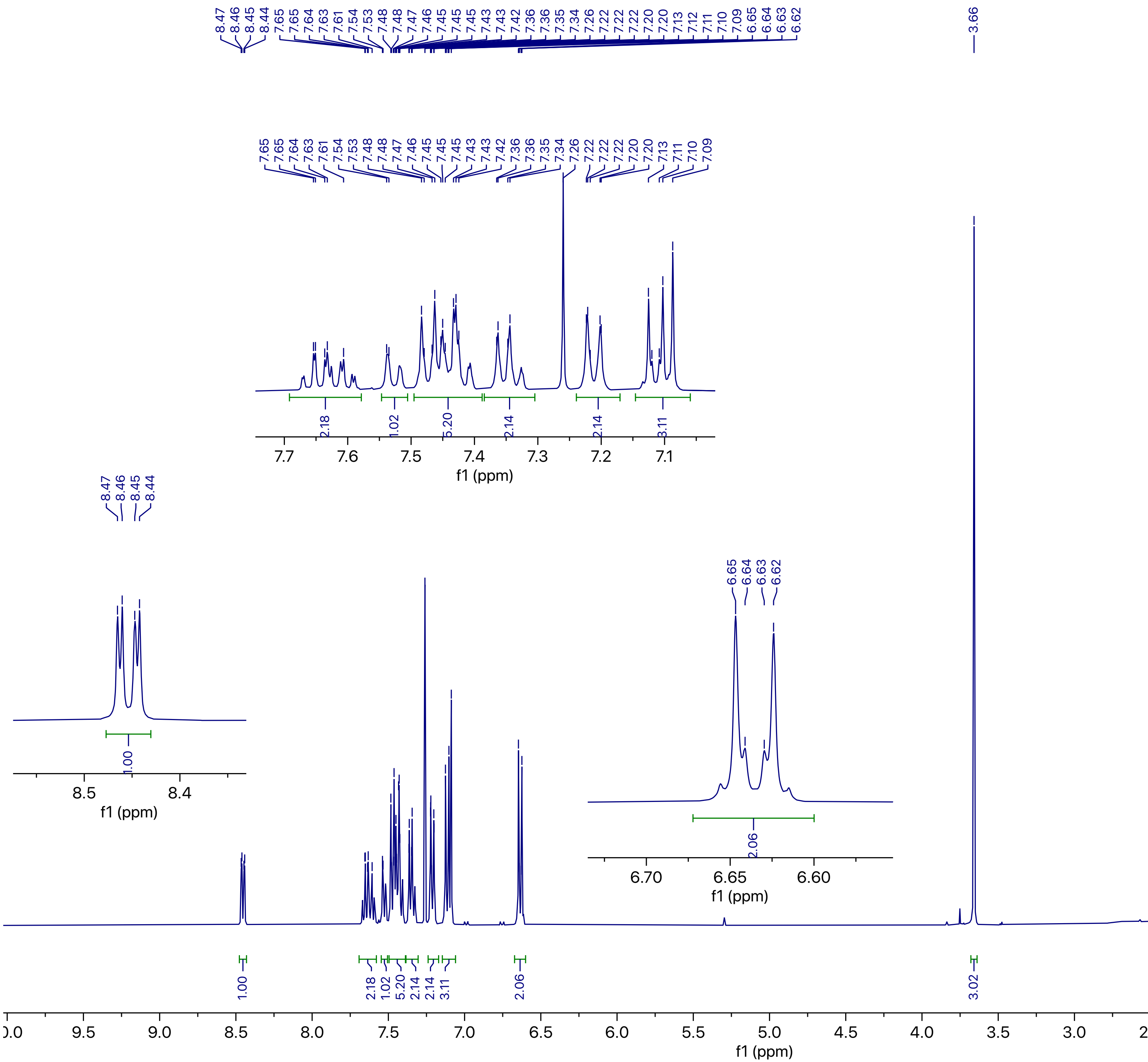

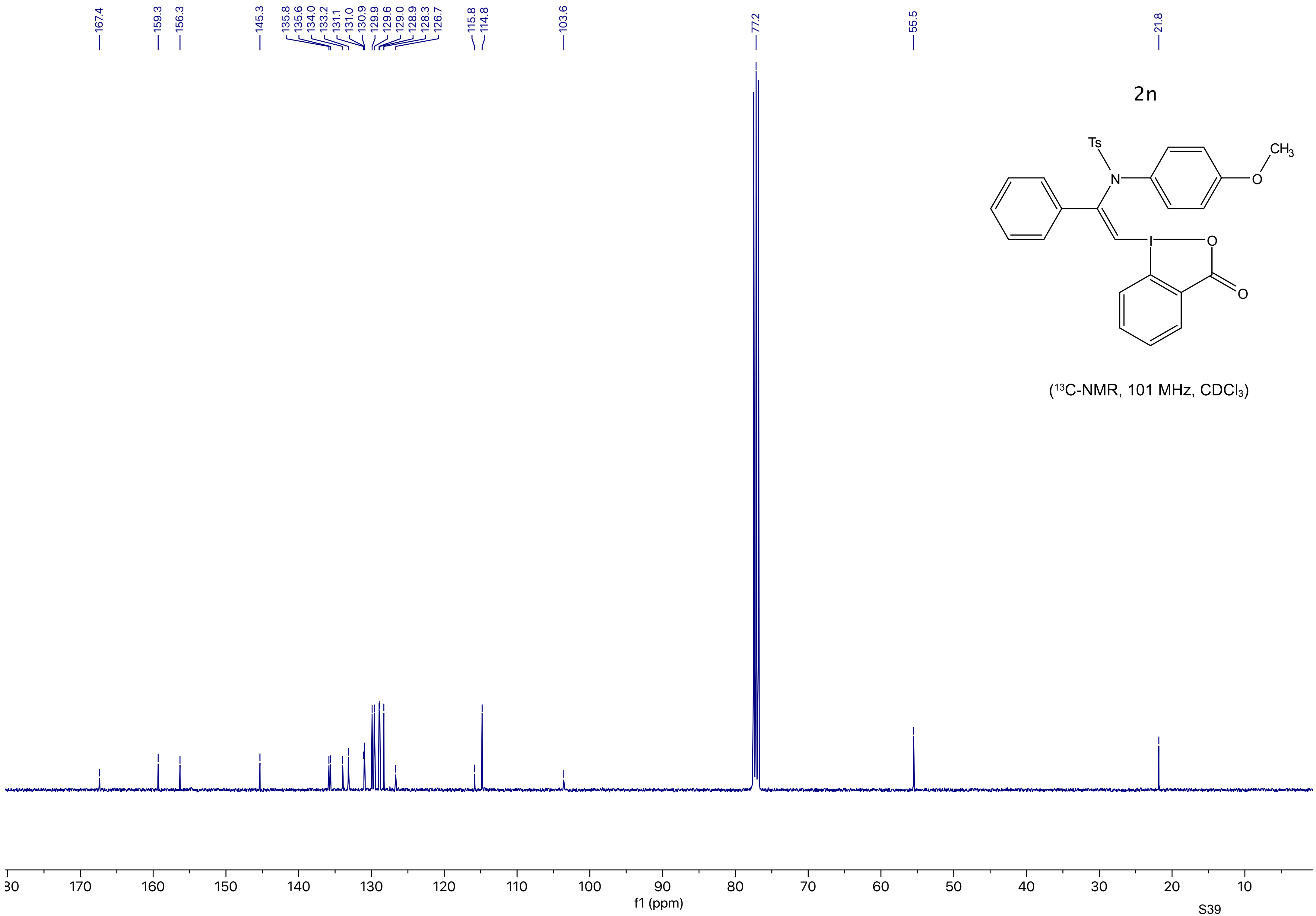
167.4
159.3
156.3
145.3
135.8
135.6
134.0
133.2
131.1
131.0
130.9
129.9
129.6
129.0
128.9
128.3
126.7
115.8
114.8
103.6
77.2
55.5
21.8
2n
Ts
N
CH3
O
I
O
O
(13C-NMR, 101 MHz, CDCl3)
f1 (ppm)

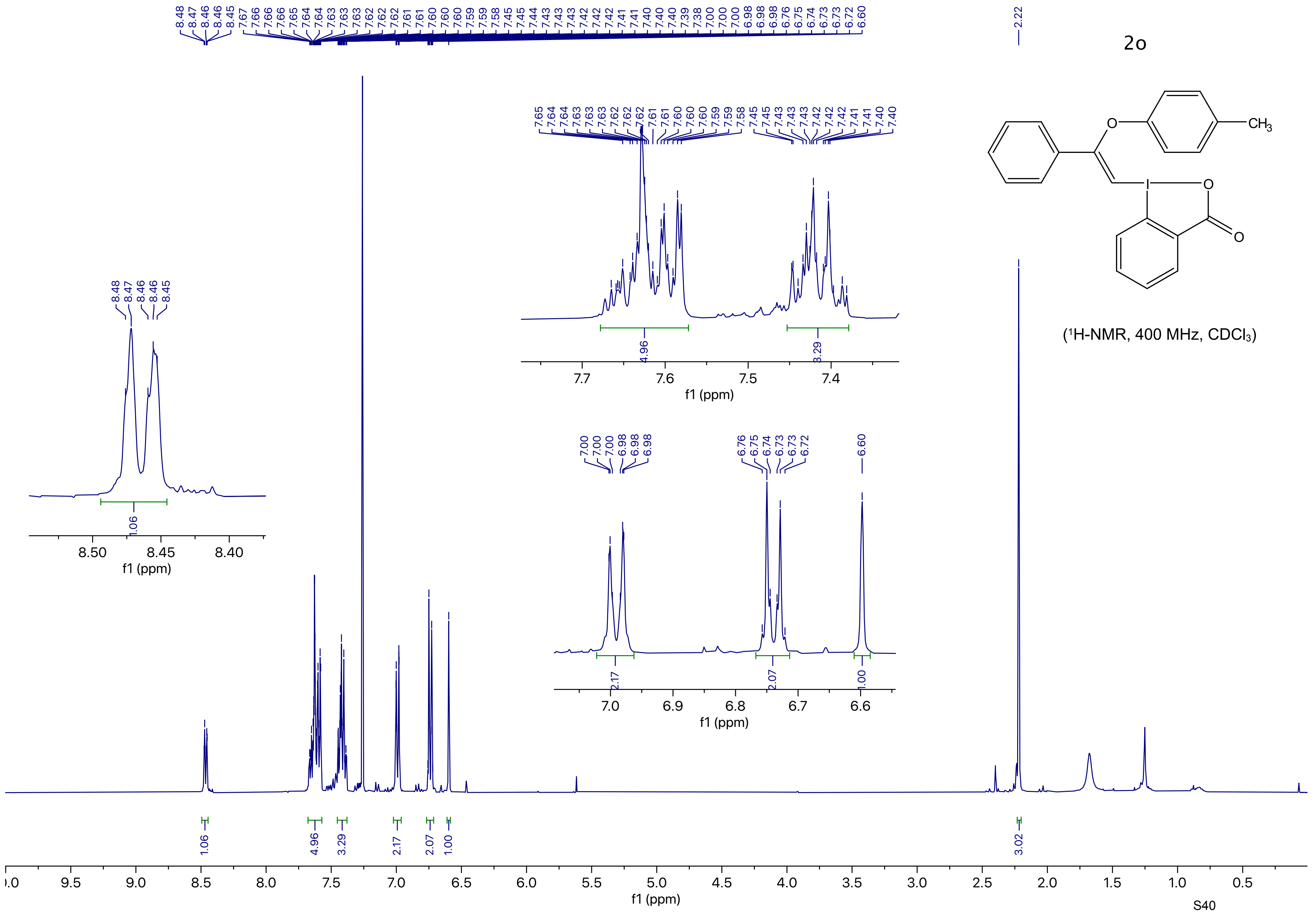
2o
CH3
O
I
O
O
(1H-NMR, 400 MHz, CDCl3)
f1 (ppm)

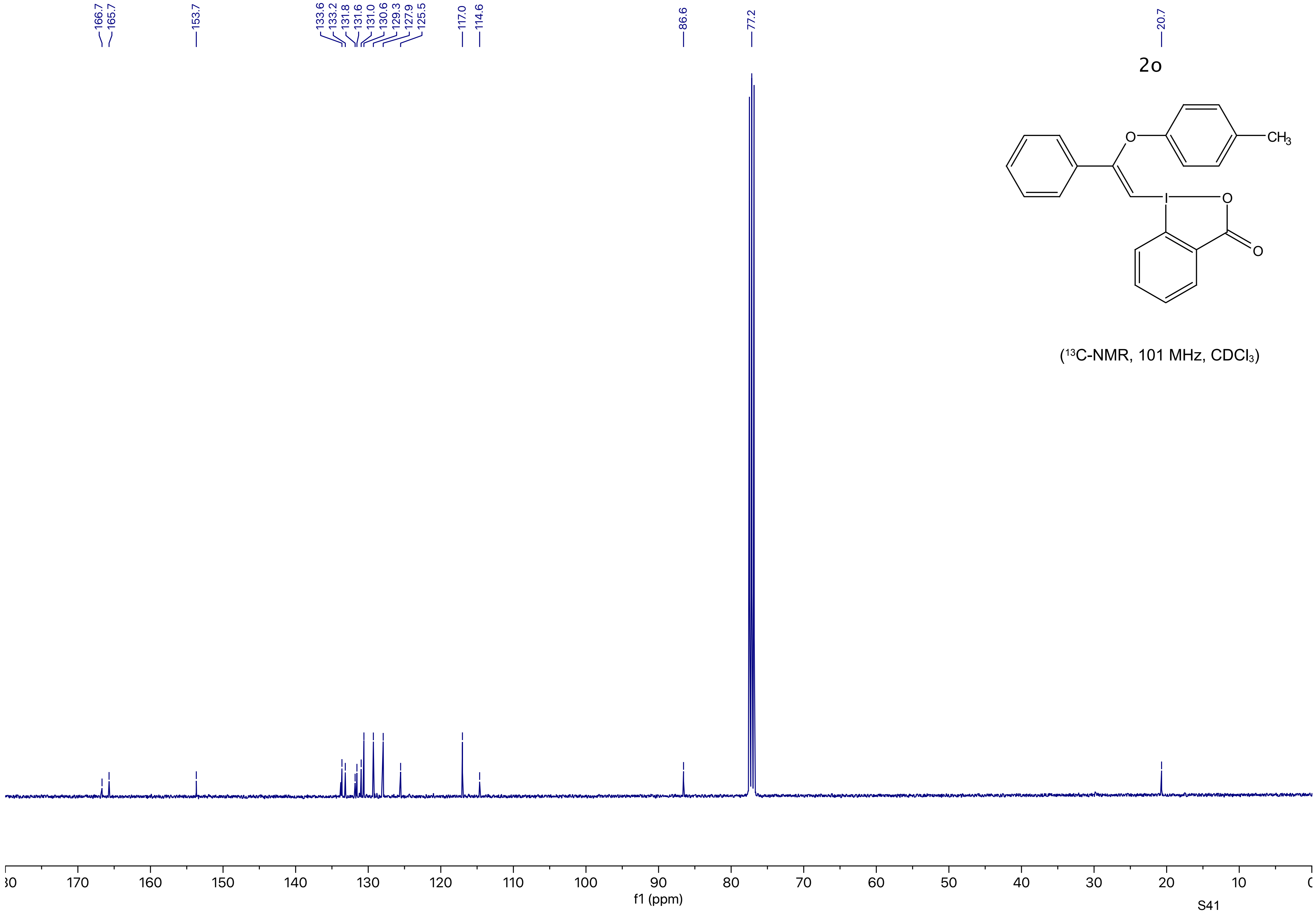
166.7
165.7
153.7
133.6
133.2
131.8
131.6
131.0
130.6
129.3
127.9
125.5
117.0
114.6
86.6
77.2
20.7
2o
O
CH3
I
O
O
(13C-NMR, 101 MHz, CDCl3)
170
160
150
140
130
120
110
100
90
80
70
60
50
40
30
20
10
f1 (ppm)

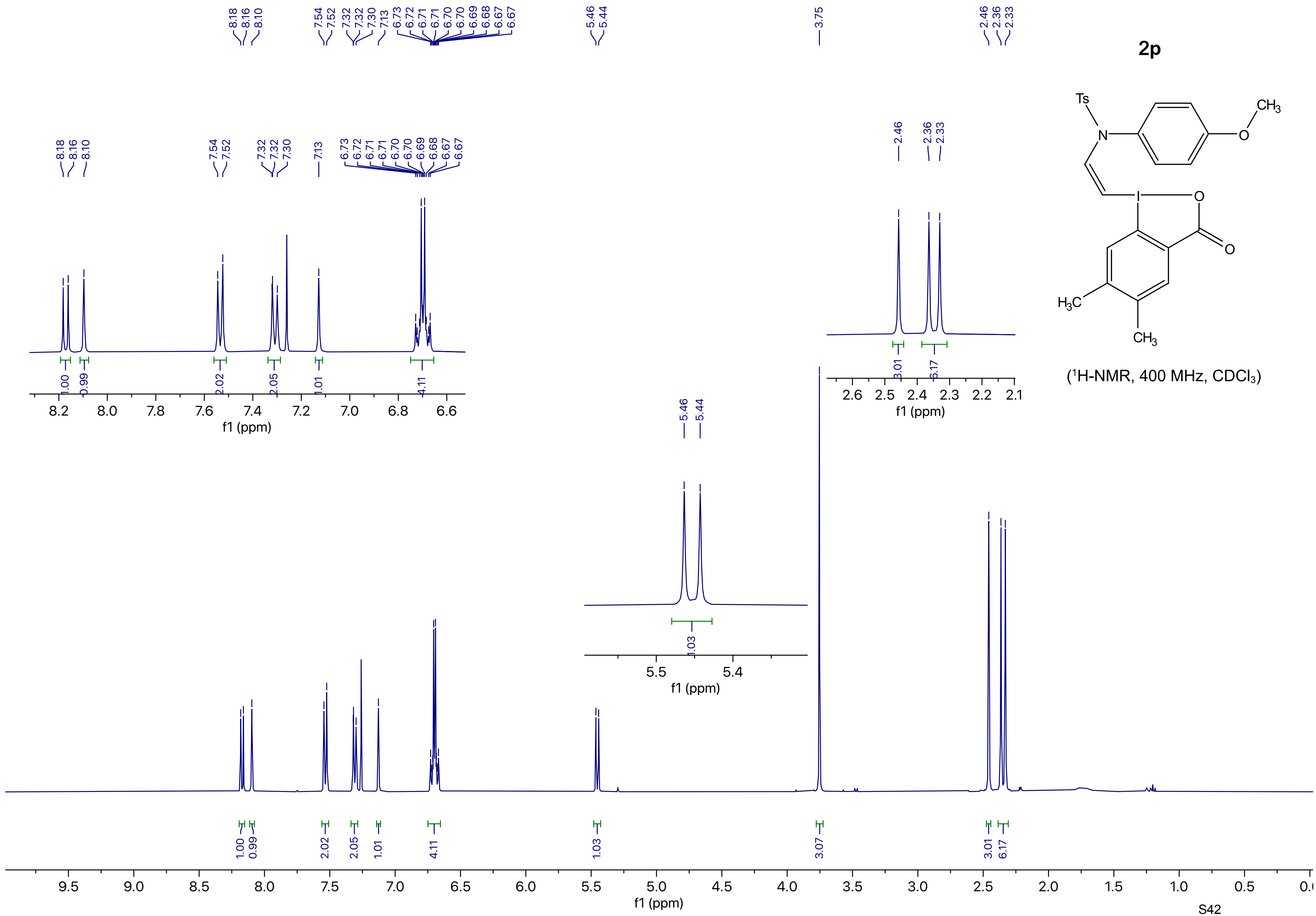

2p
Ts
N
O
CH3
I
O
O
H3C
CH3
(1H-NMR, 400 MHz, CDCl3)
f1 (ppm)

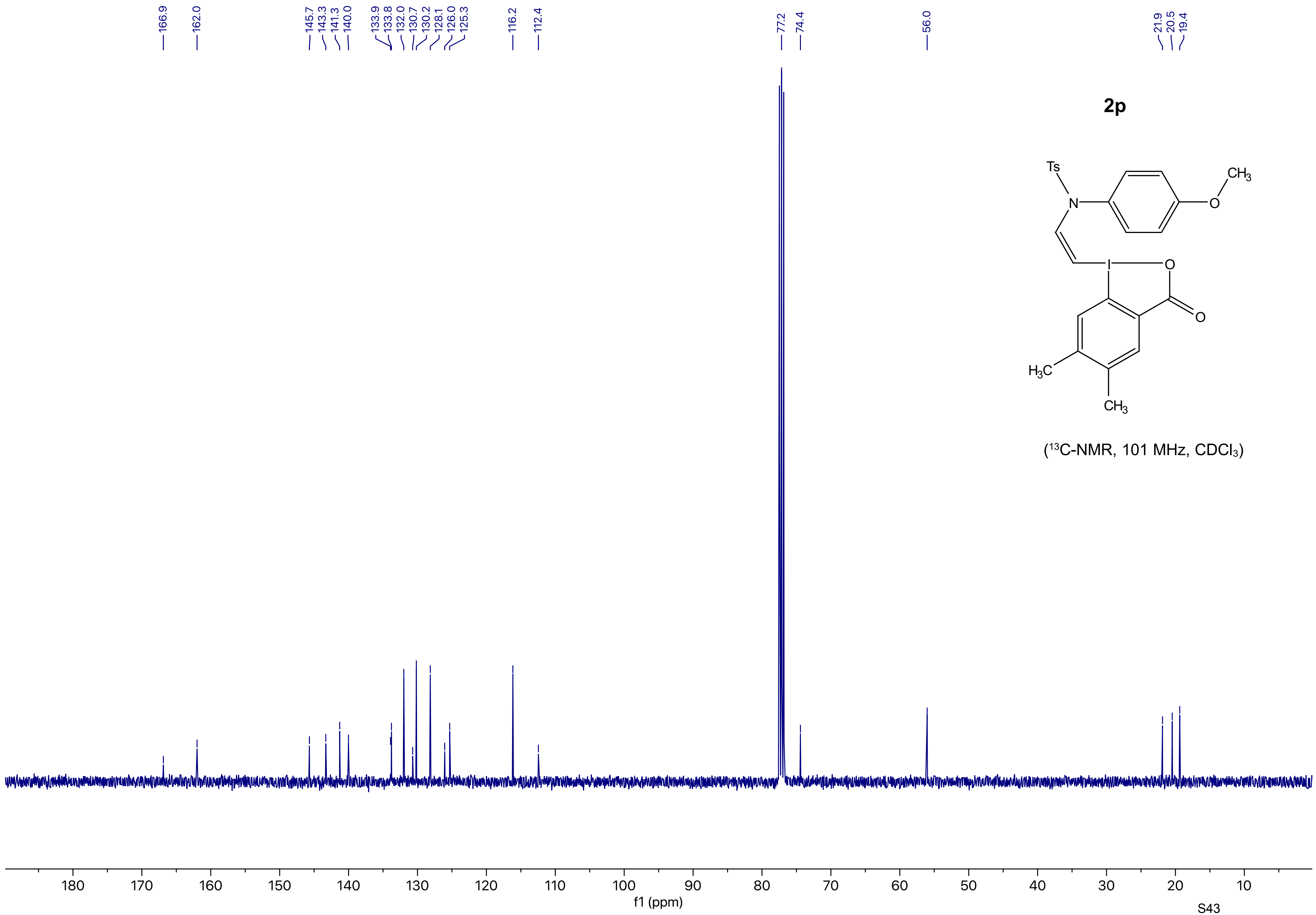

2p

(^{13}C-NMR, 101 MHz, $CDCl_3$)

3a

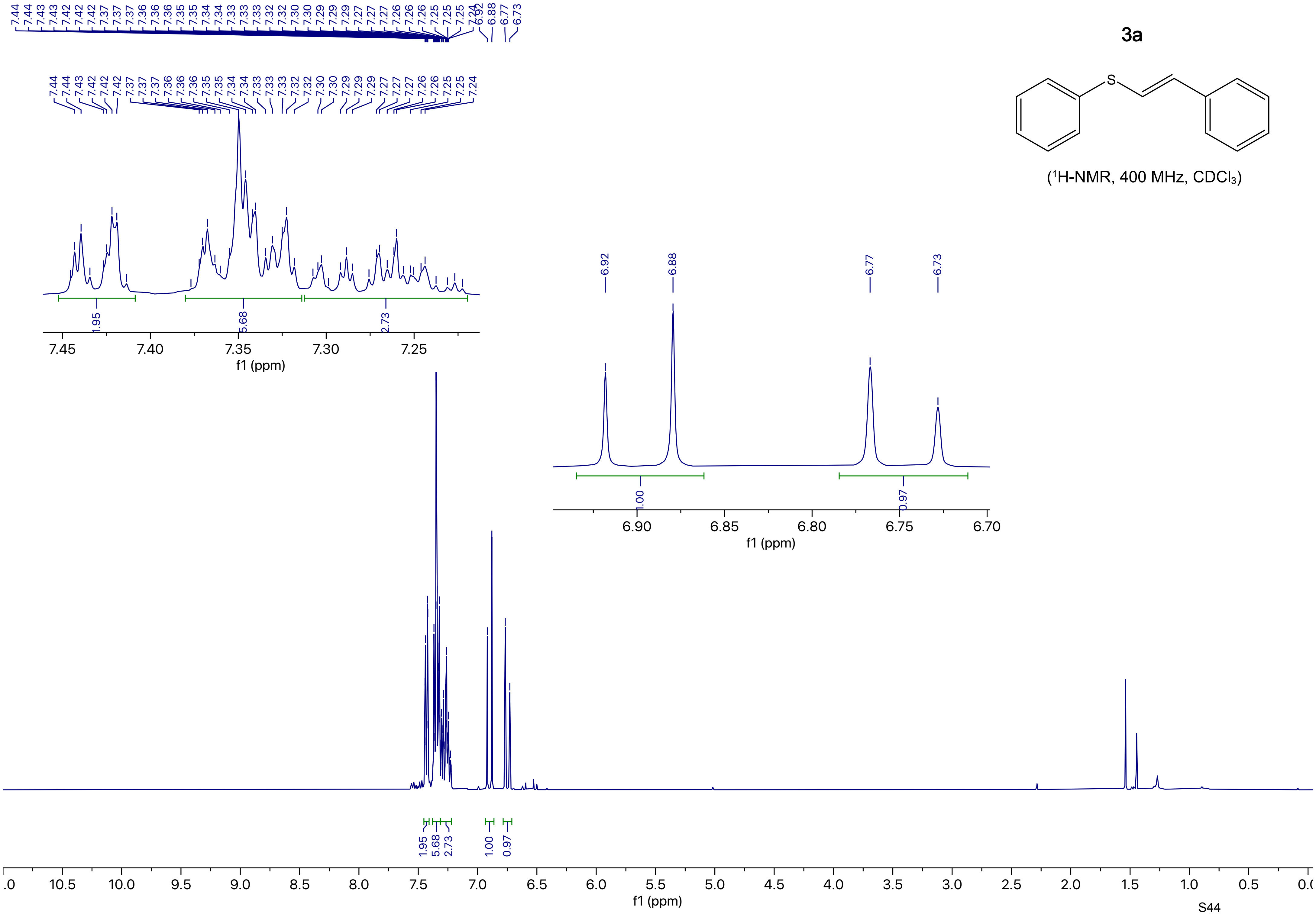

(^{1}H-NMR, 400 MHz, $CDCl_3$)

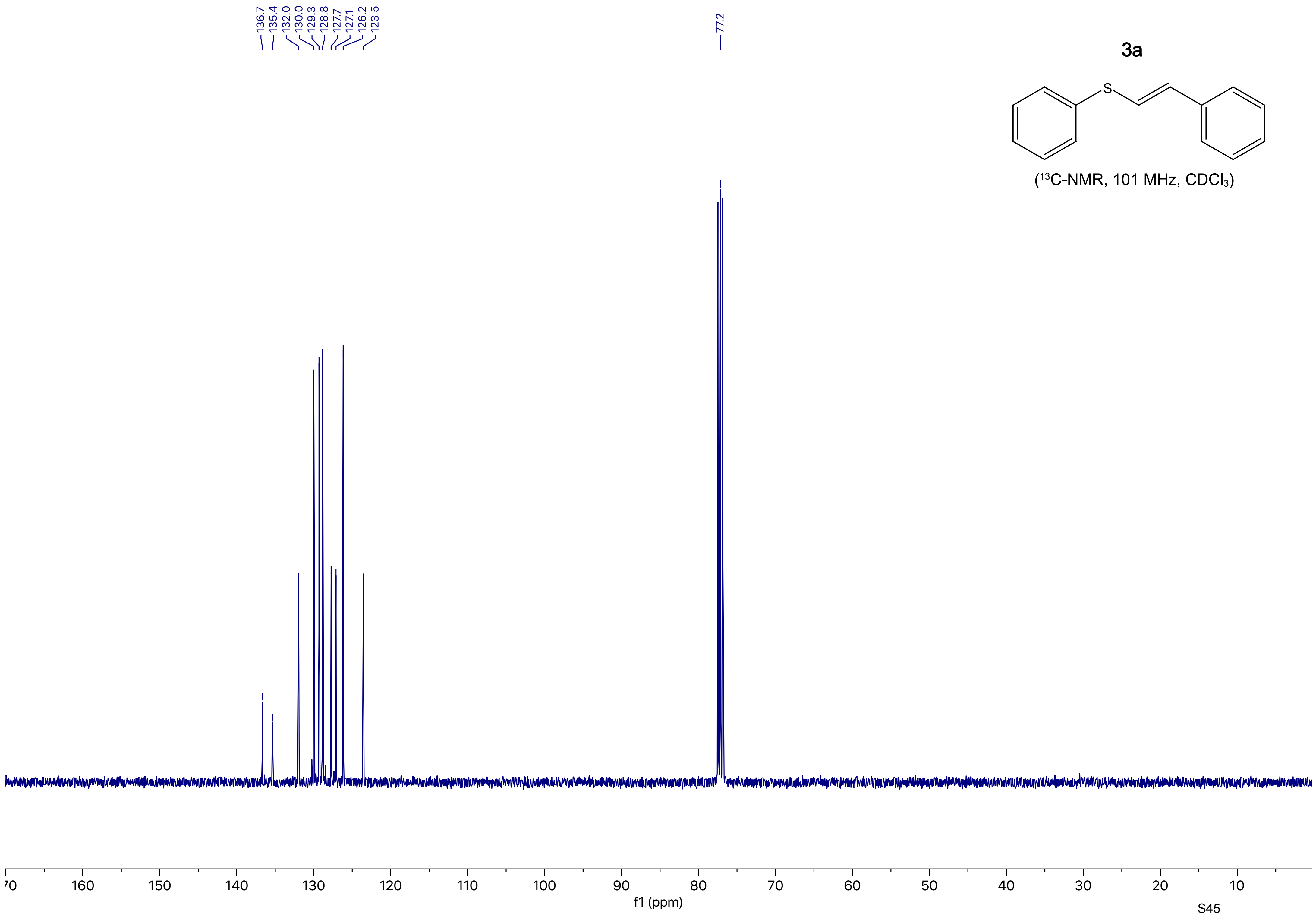
136.7
135.4
132.0
130.0
129.3
128.8
127.7
127.1
126.2
123.5
77.2
3a
S
(^{13}C-NMR, 101 MHz, $CDCl_3$)
160
150
140
130
120
110
100
90
80
70
60
50
40
30
20
10
f1 (ppm)

3b

(^{1}H-NMR, 400 MHz, $CDCl_3$)

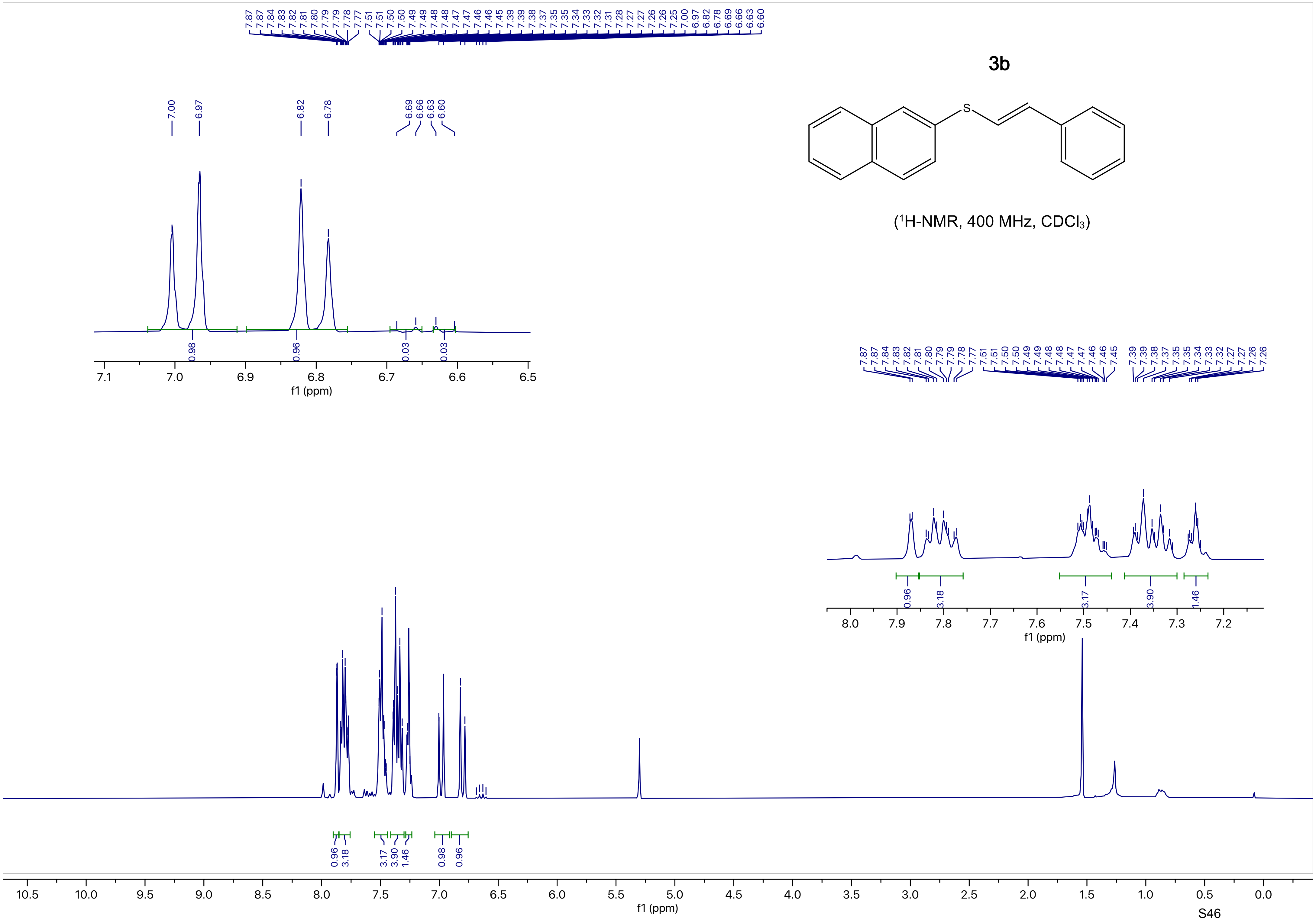

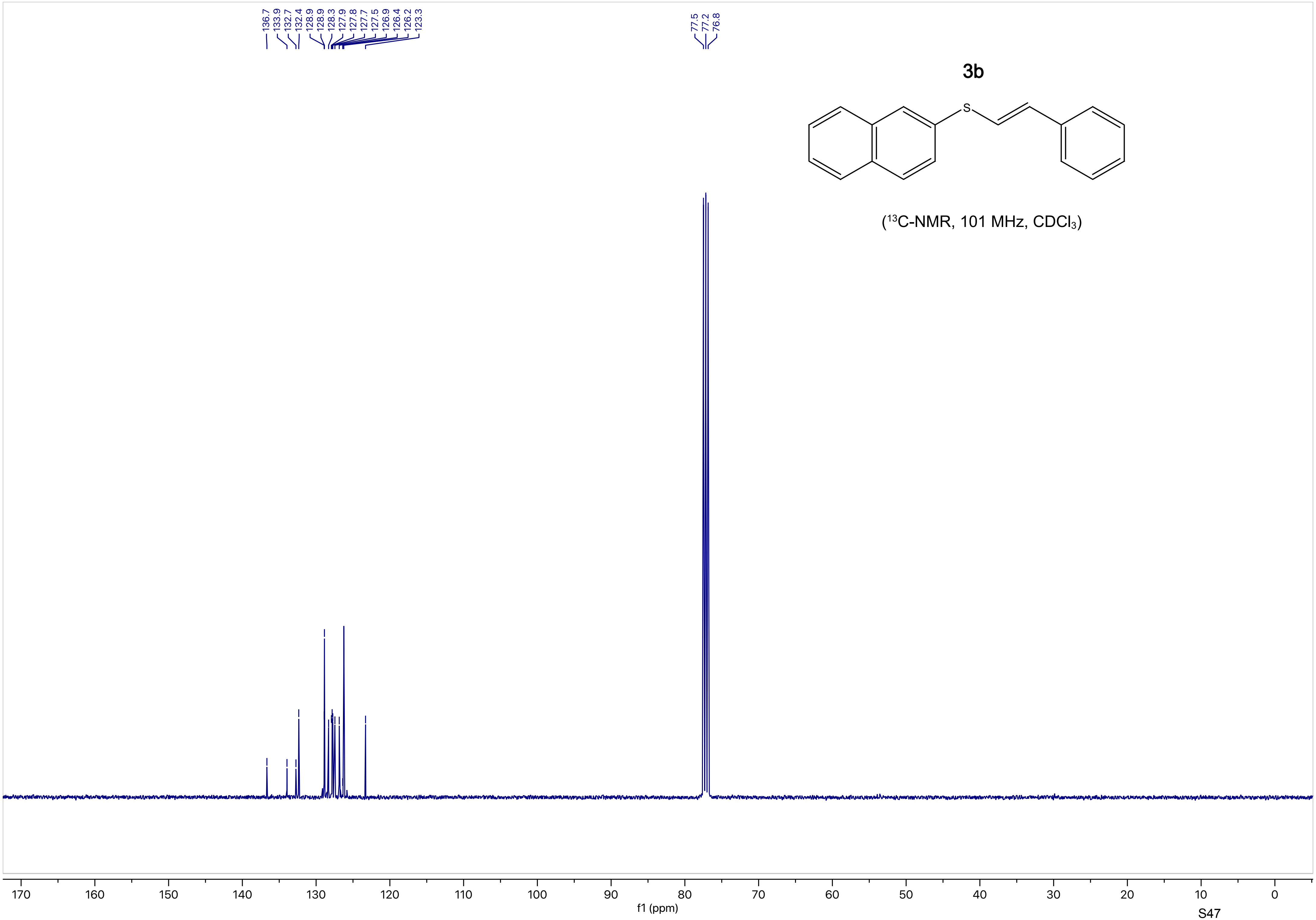
136.7
133.9
132.7
132.4
128.9
128.9
128.3
127.9
127.8
127.7
127.5
126.9
126.4
126.2
123.3
77.5
77.2
76.8
3b
S
(13C-NMR, 101 MHz, CDCl3)
170
160
150
140
130
120
110
100
90
80
70
60
50
40
30
20
10
0
f1 (ppm)

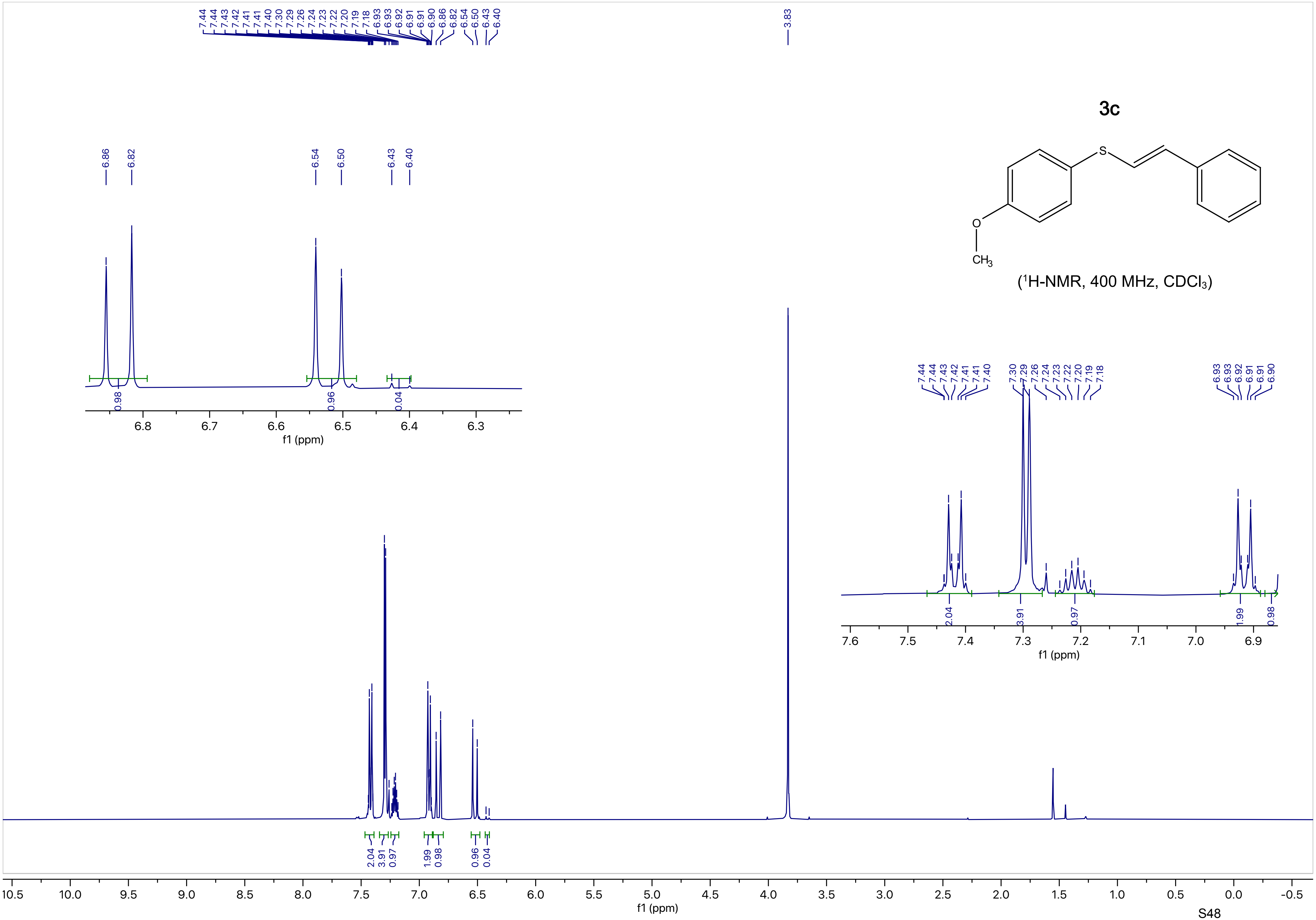
3c
S
O
CH3
(1H-NMR, 400 MHz, CDCl3)
7.44 7.44 7.43 7.42 7.41 7.41 7.40 7.30 7.29 7.26 7.24 7.23 7.22 7.20 7.19 7.18 6.93 6.93 6.92 6.91 6.91 6.90 6.86 6.82 6.54 6.50 6.43 6.40
3.83
6.86 6.82 6.54 6.50 6.43 6.40
0.98 0.96 0.04
f1 (ppm)
2.04 3.91 0.97 1.99 0.98
2.04 3.91 0.97 1.99 0.98 0.96 0.04
f1 (ppm)

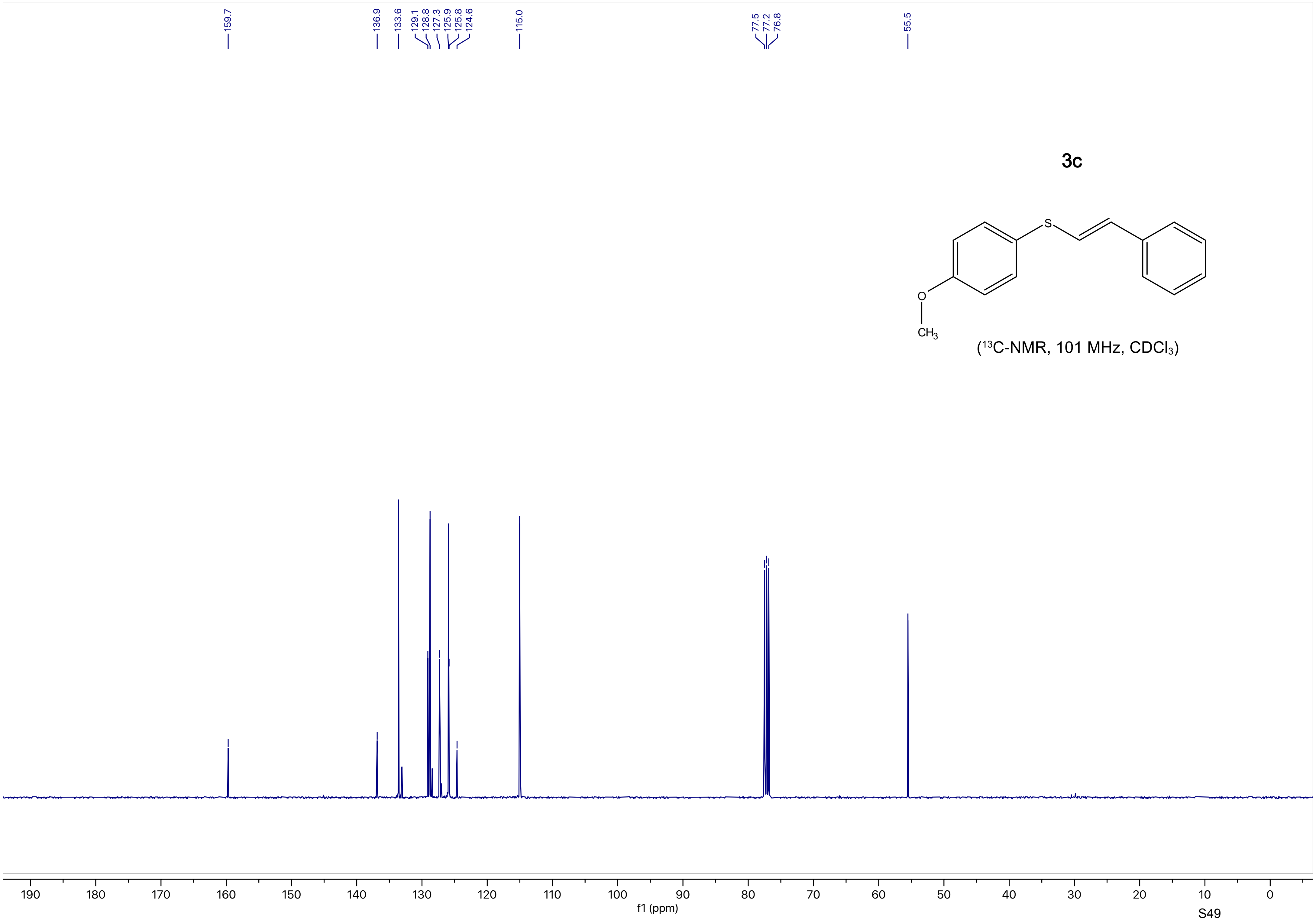
159.7
136.9
133.6
129.1
128.8
127.3
125.9
125.8
124.6
115.0
77.5
77.2
76.8
55.5
3c
S
O
CH3
(13C-NMR, 101 MHz, CDCl3)
190 180 170 160 150 140 130 120 110 100 90 80 70 60 50 40 30 20 10 0
f1 (ppm)

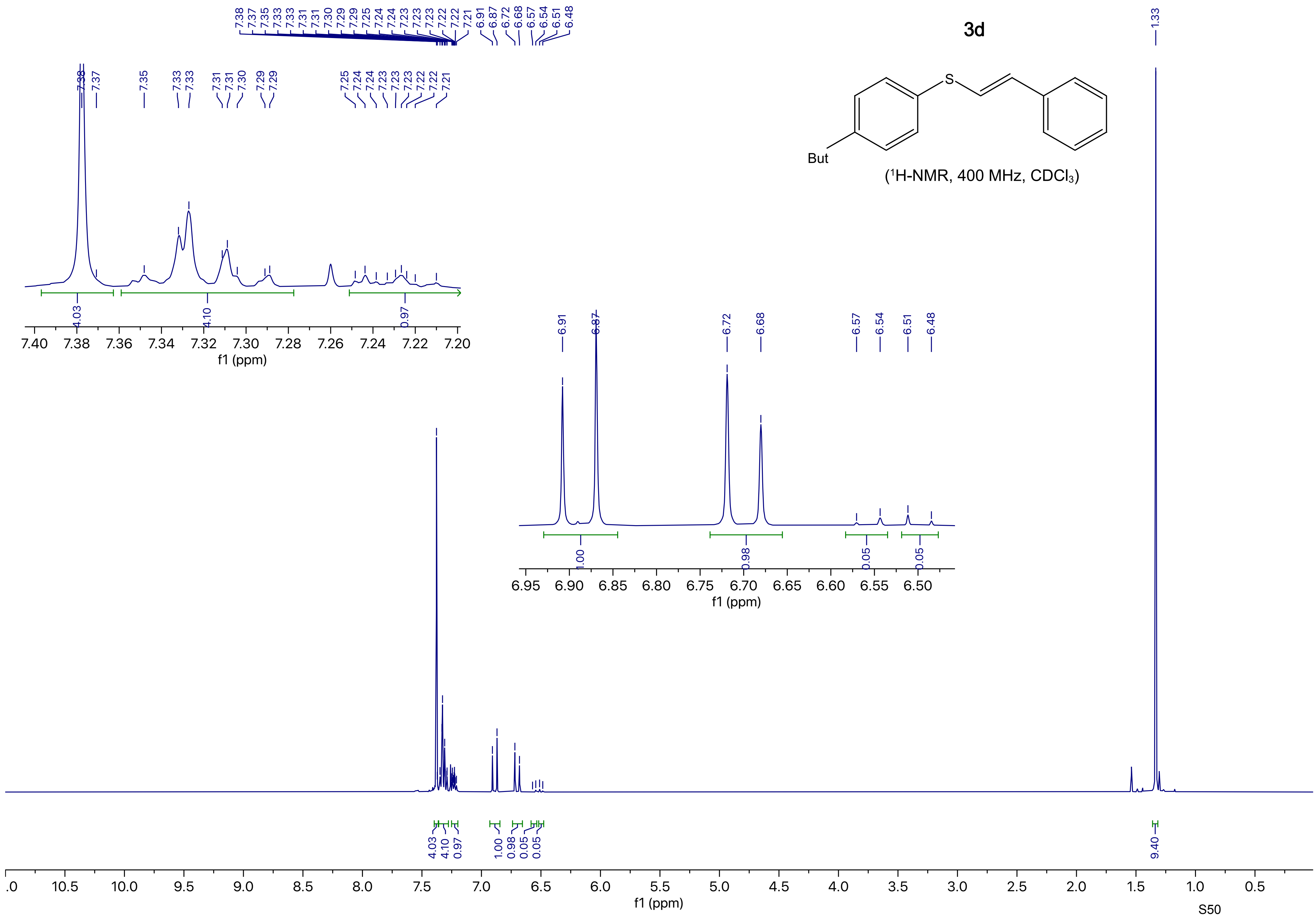
3d
S
But
(1H-NMR, 400 MHz, CDCl3)
7.38 7.37 7.35 7.33 7.33 7.31 7.31 7.30 7.29 7.29 7.25 7.24 7.24 7.23 7.23 7.23 7.22 7.22 7.21 6.91 6.87 6.72 6.68 6.57 6.54 6.51 6.48
1.33
4.03 4.10 0.97 1.00 0.98 0.05 0.05 9.40
f1 (ppm)

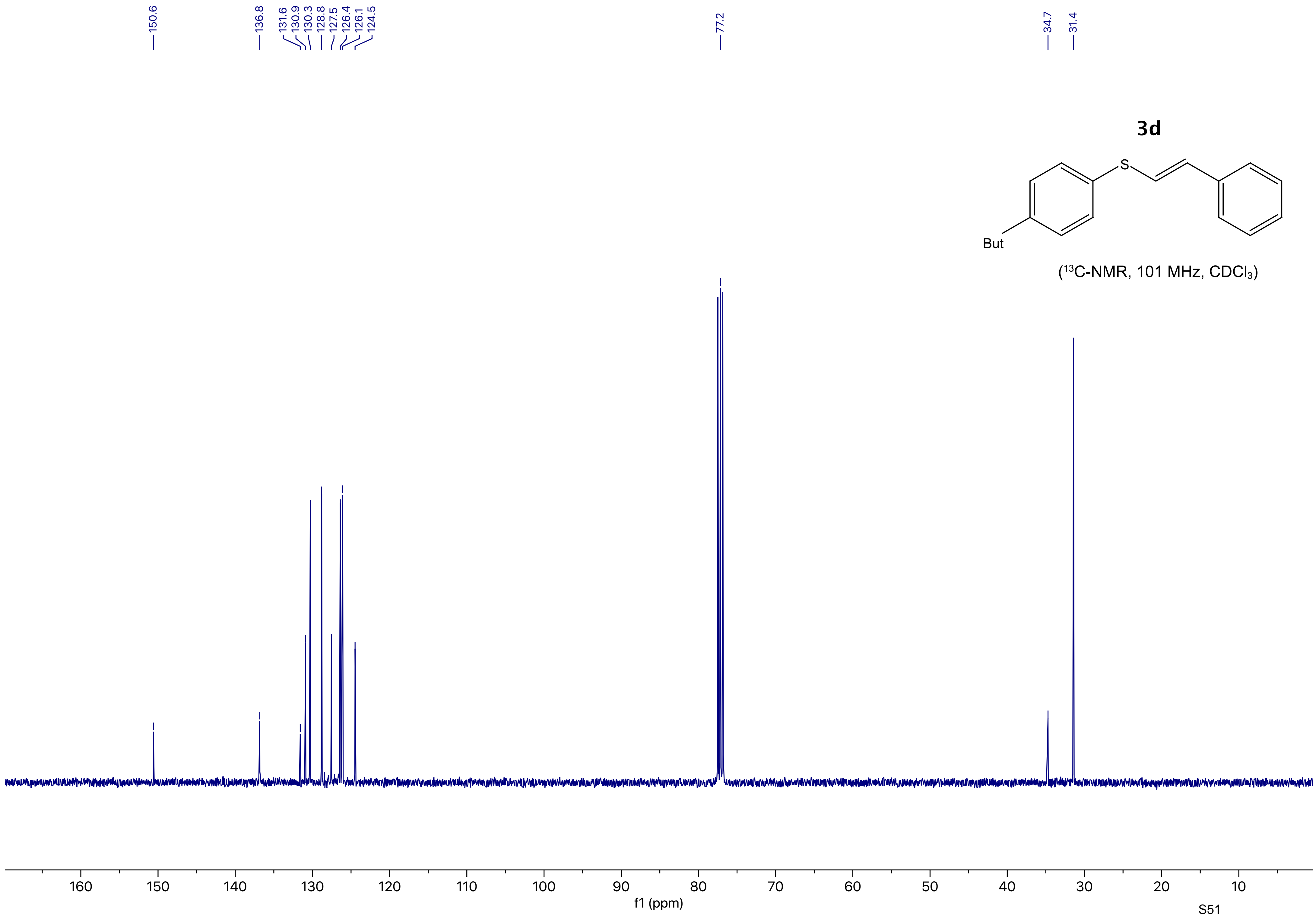
150.6
136.8
131.6
130.9
130.3
128.8
127.5
126.4
126.1
124.5
77.2
34.7
31.4
3d
S
But
(^{13}C-NMR, 101 MHz, CDCl$_3$)
160
150
140
130
120
110
100
90
80
70
60
50
40
30
20
10
f1 (ppm)

3e

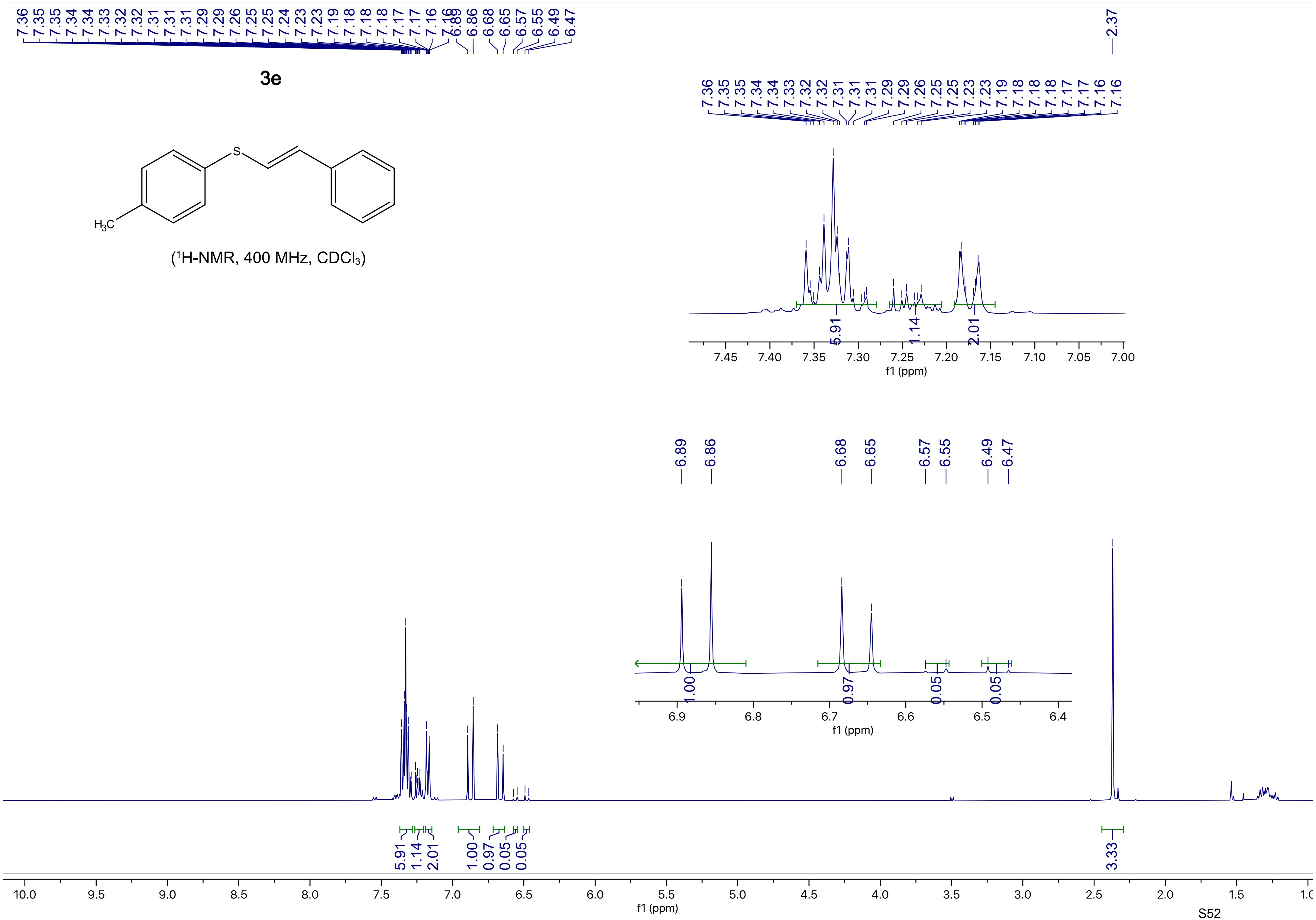
S
H3C
(1H-NMR, 400 MHz, CDCl3)
f1 (ppm)
5.91
1.14
2.01
1.00
0.97
0.05
0.05
3.33
2.37

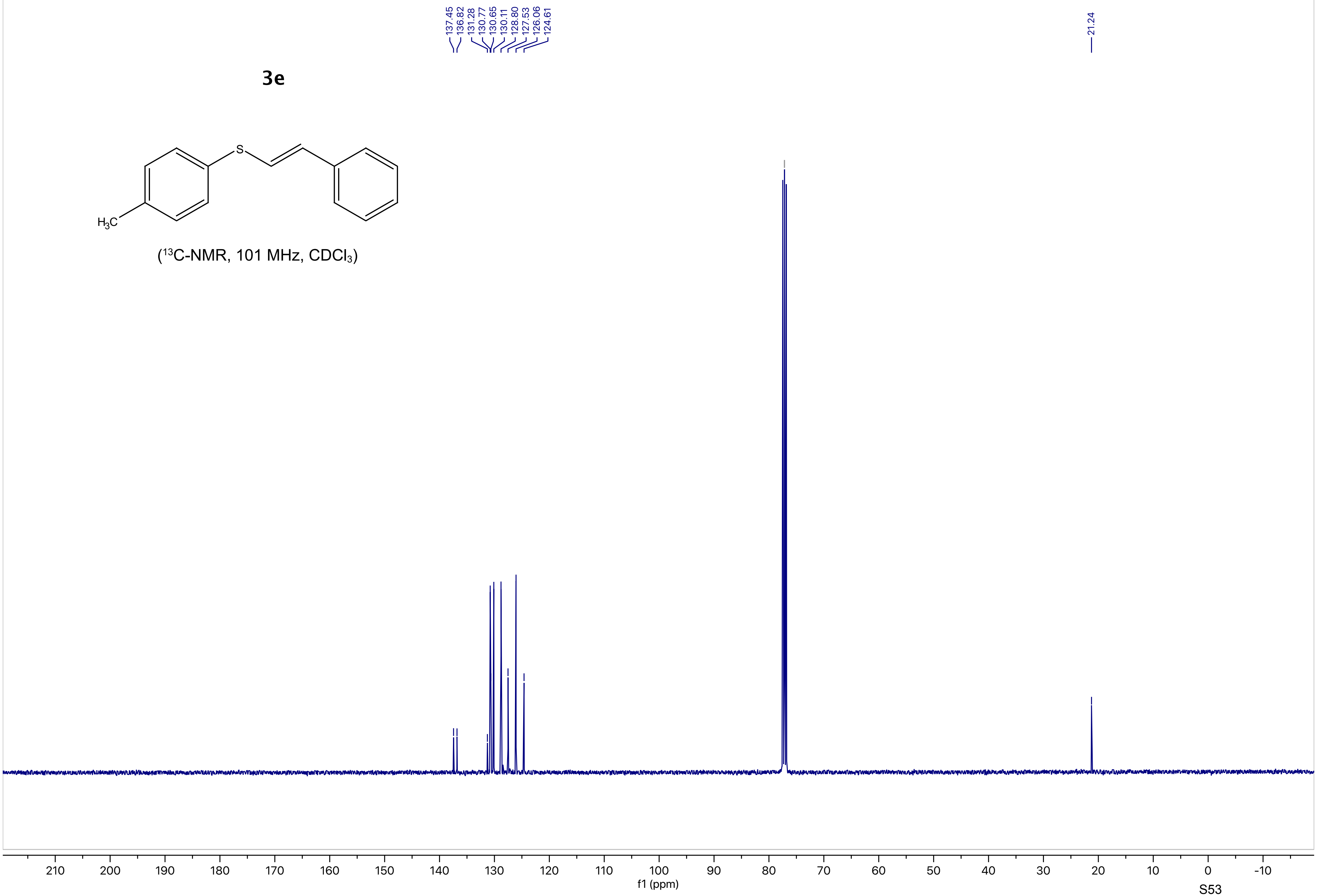

3e

(^{13}C-NMR, 101 MHz, $CDCl_3$)

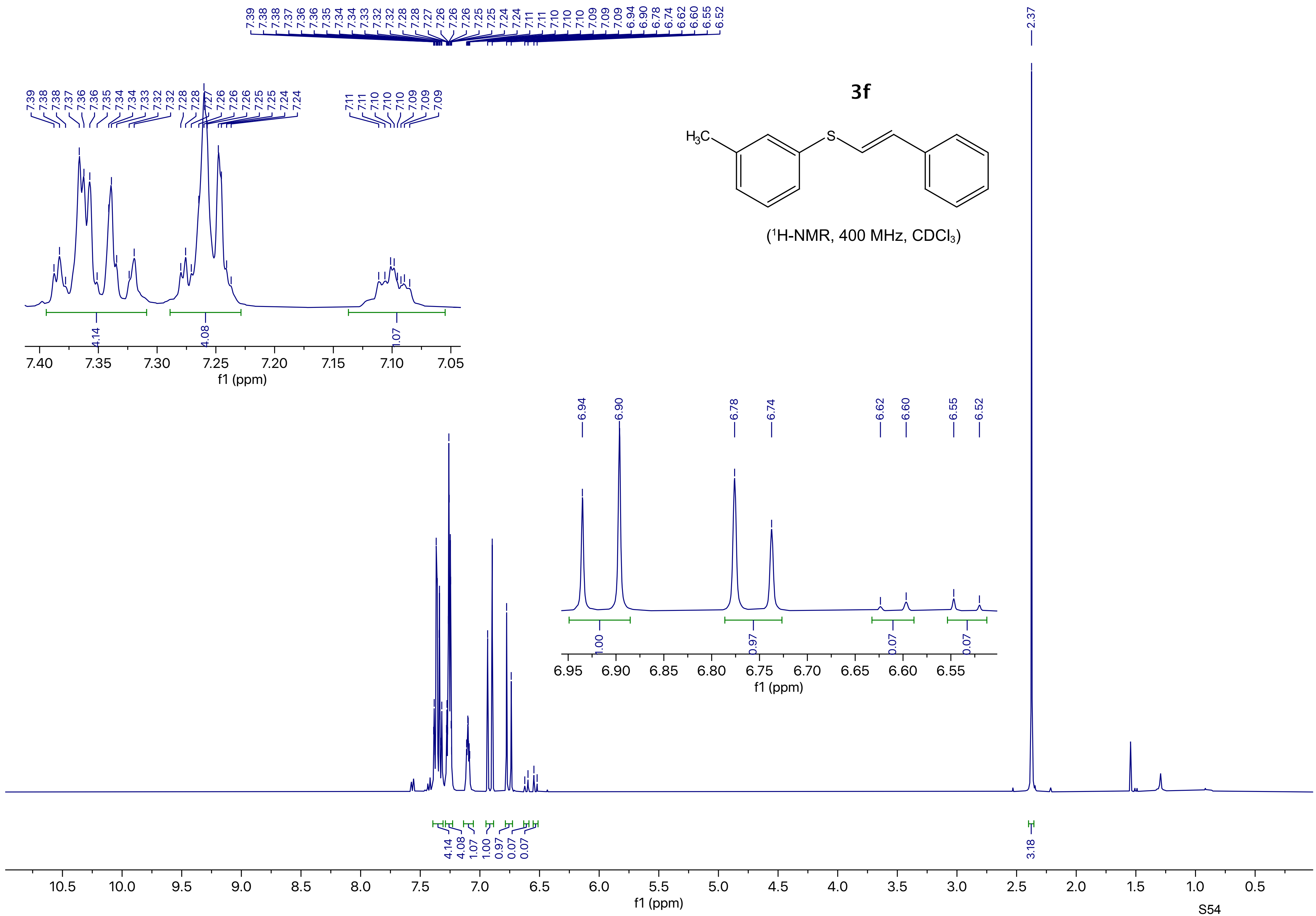

3f
H3C
S
(1H-NMR, 400 MHz, CDCl3)
2.37
4.14
4.08
1.07
1.00
0.97
0.07
0.07
3.18
f1 (ppm)

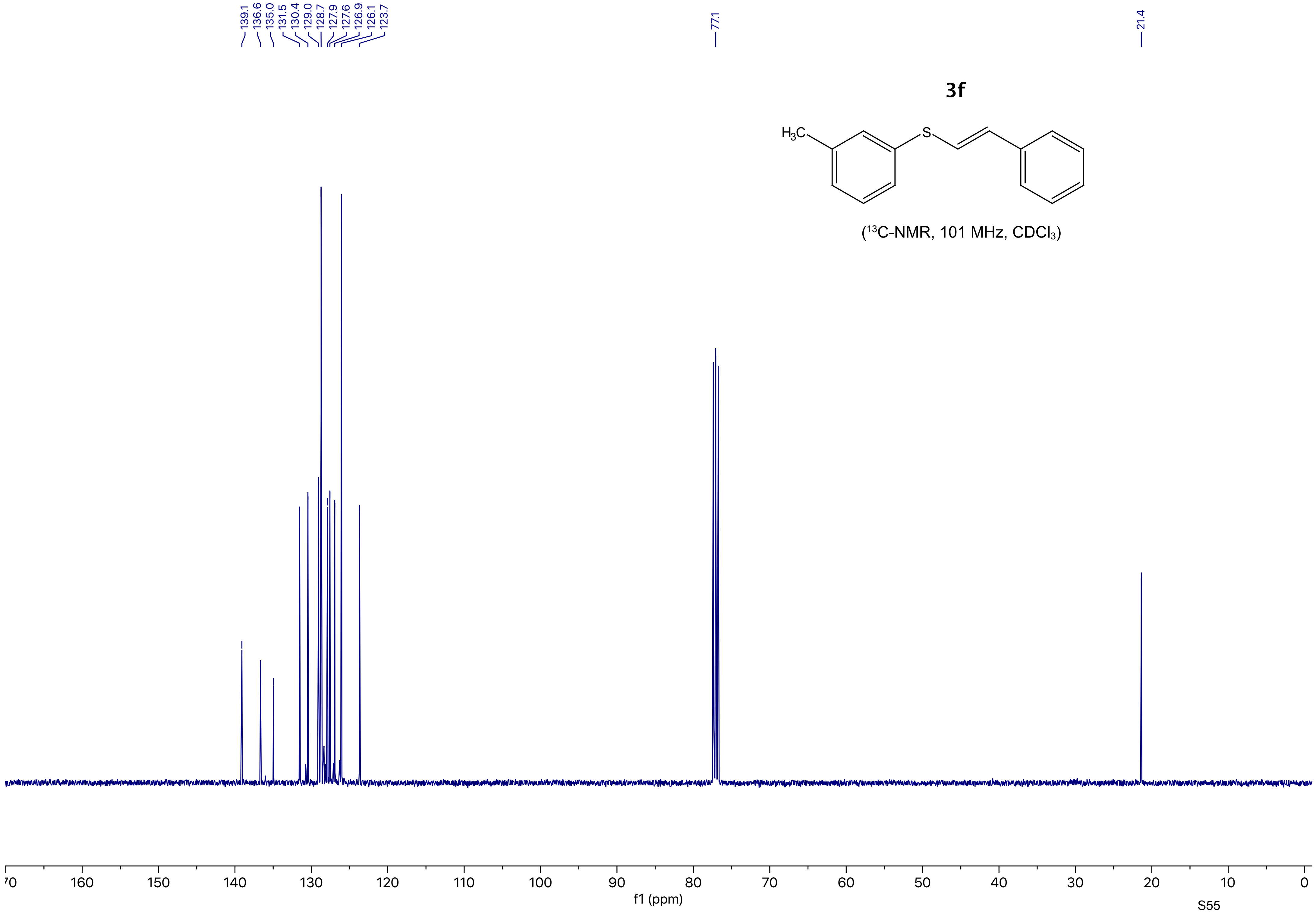

3f

(^{13}C-NMR, 101 MHz, $CDCl_3$)

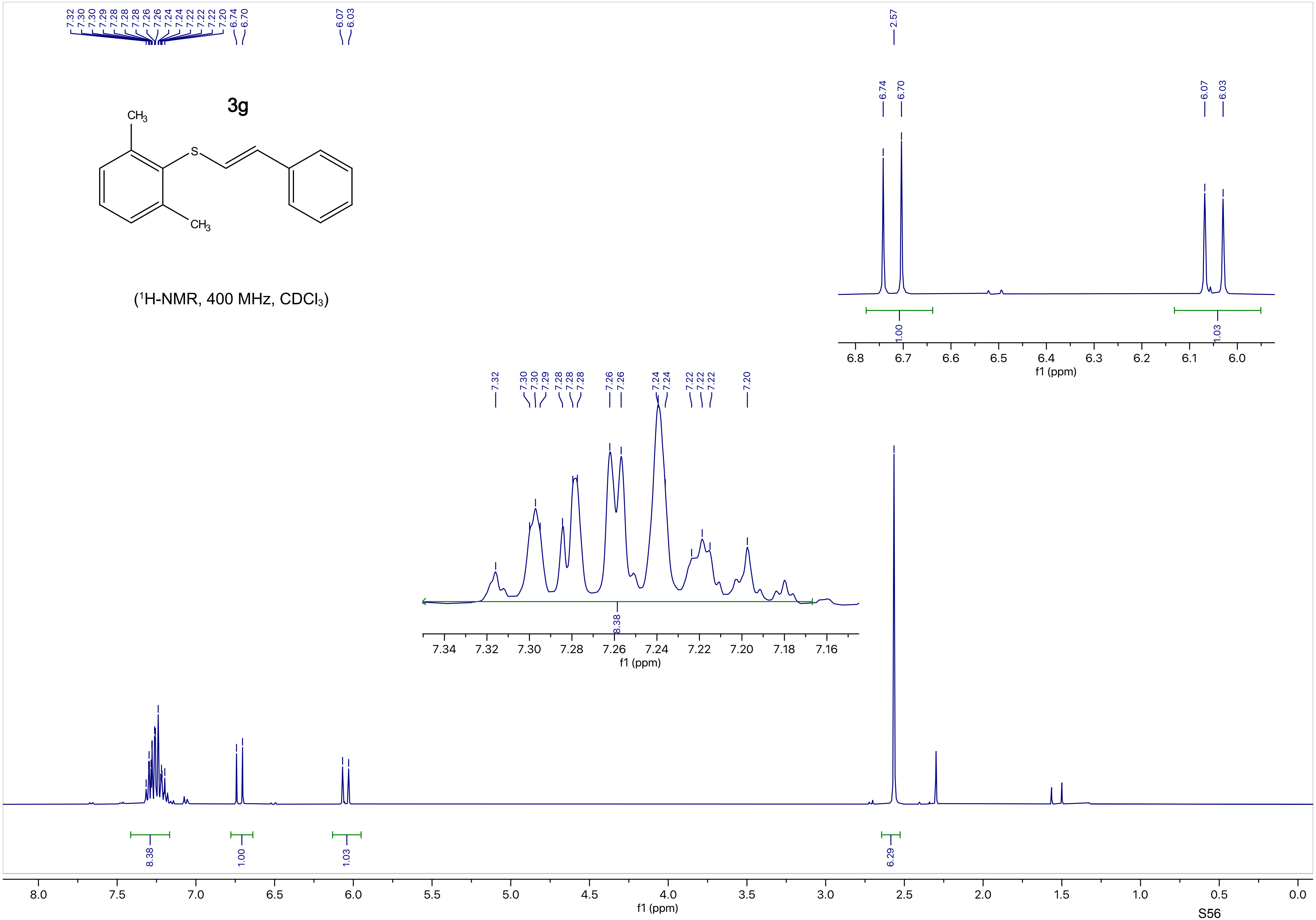
3g
CH3
S
CH3
(1H-NMR, 400 MHz, CDCl3)
7.32
7.30
7.30
7.29
7.28
7.28
7.28
7.26
7.26
7.24
7.24
7.22
7.22
7.22
7.20
6.74
6.70
6.07
6.03
2.57
8.38
1.00
1.03
6.29
f1 (ppm)

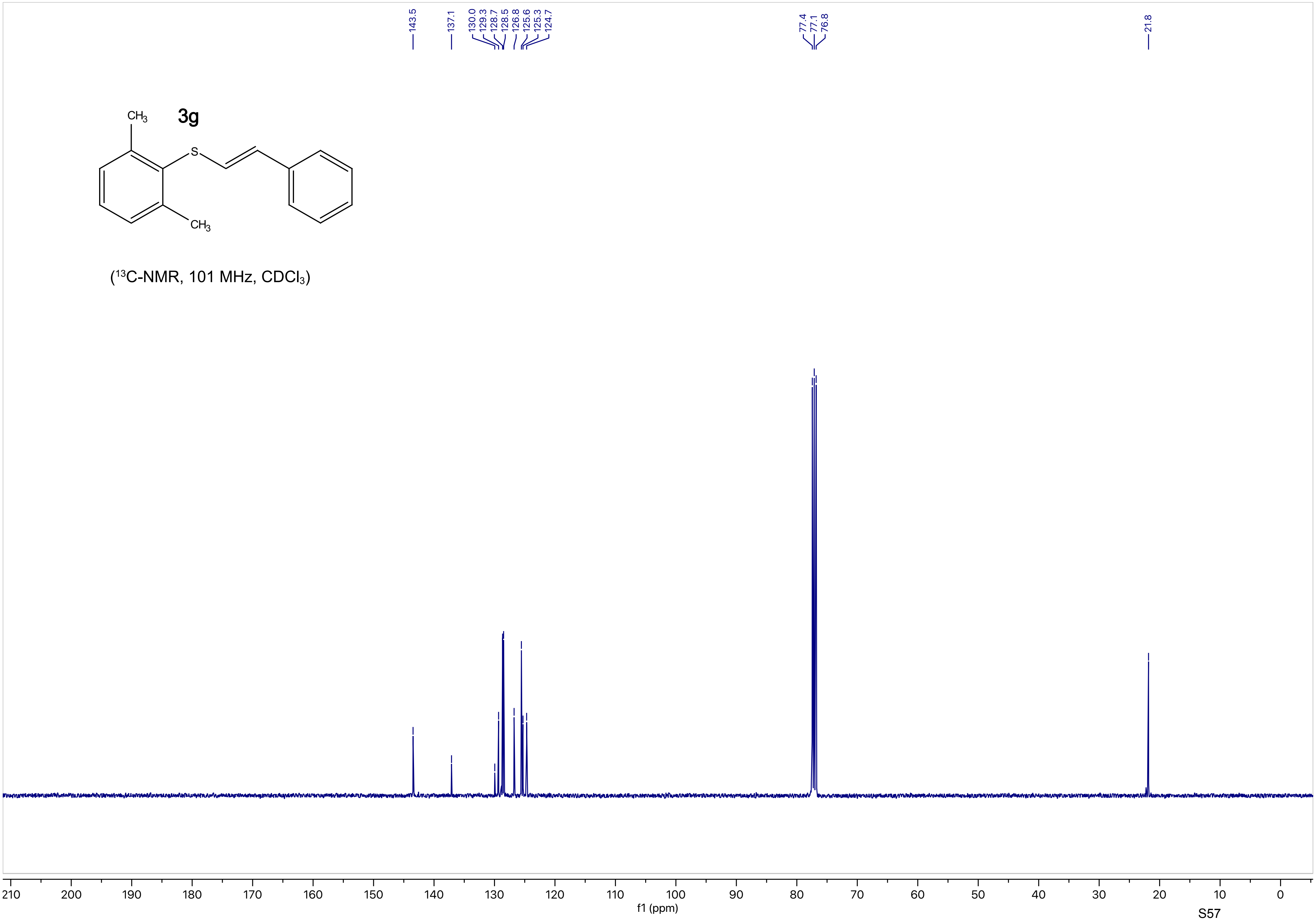
143.5
137.1
130.0
129.3
128.7
128.5
126.8
125.6
125.3
124.7
77.4
77.1
76.8
21.8
3g
(^{13}C-NMR, 101 MHz, $CDCl_3$)
f1 (ppm)

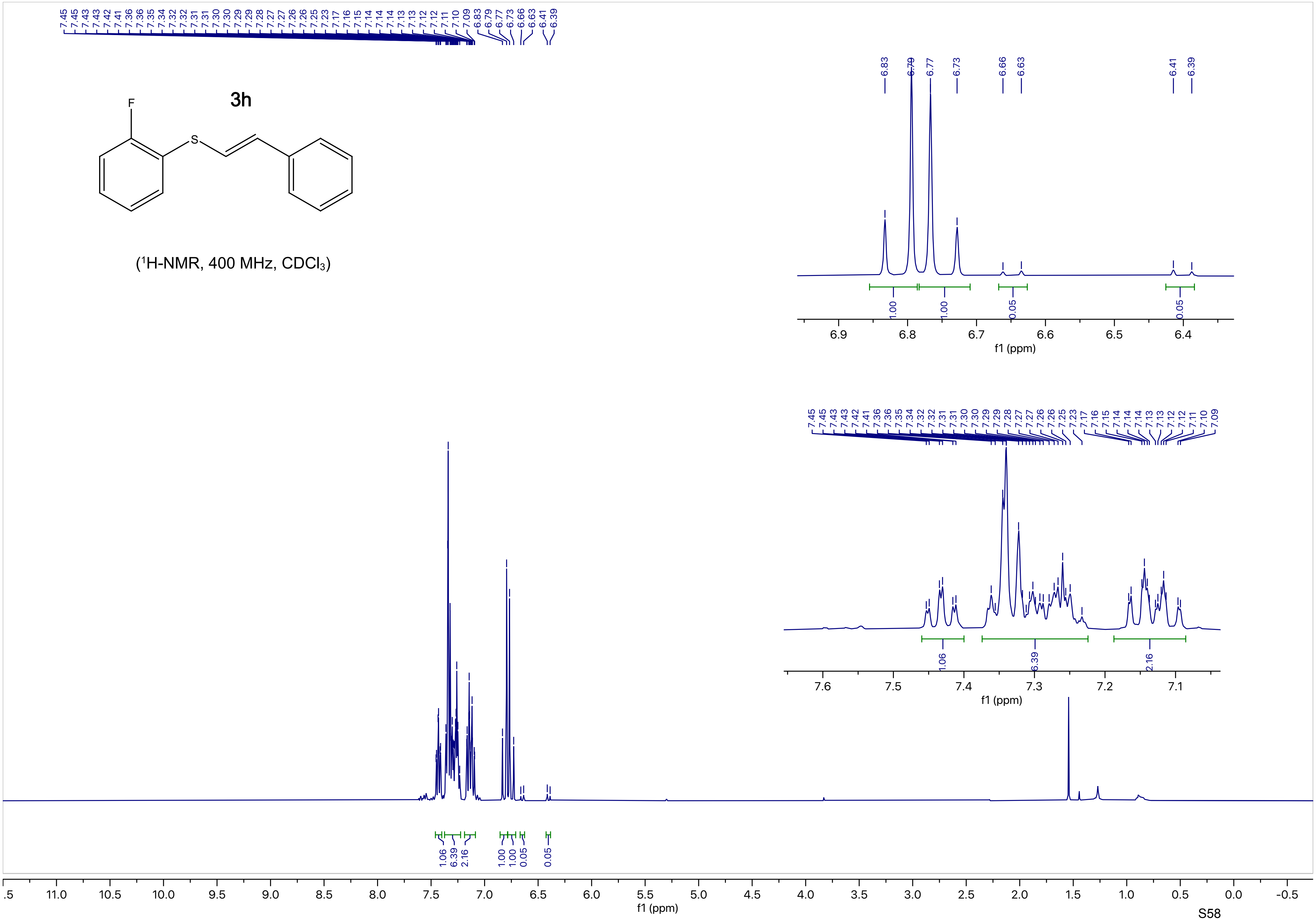

3h

(¹H-NMR, 400 MHz, $CDCl_3$)

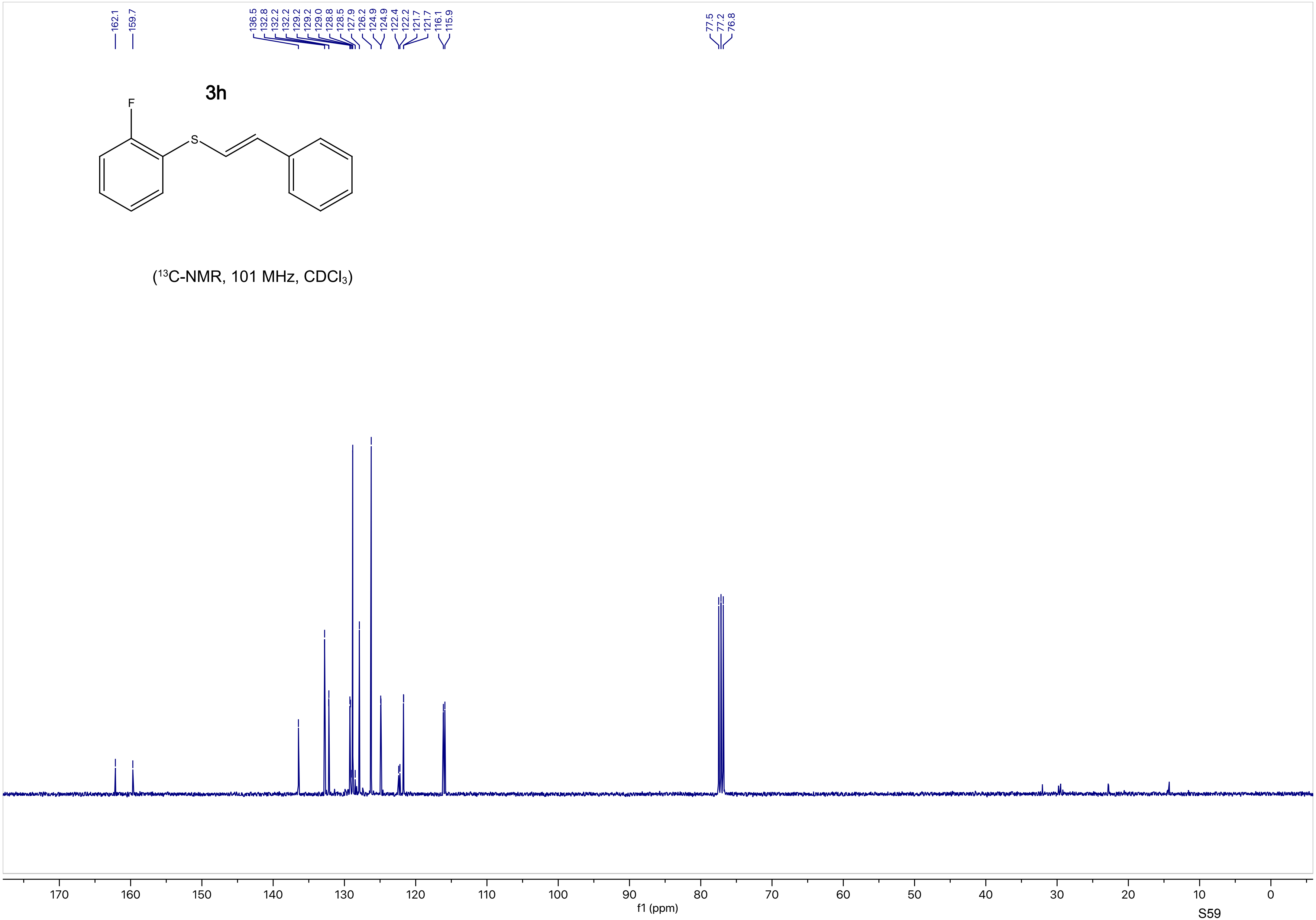

162.1
159.7
136.5
132.8
132.2
132.2
129.2
129.2
129.0
128.8
128.5
127.9
126.2
124.9
124.9
122.4
122.2
121.7
121.7
116.1
115.9
77.5
77.2
76.8
3h
F
S
(^{13}C-NMR, 101 MHz, $CDCl_3$)
170 160 150 140 130 120 110 100 90 80 70 60 50 40 30 20 10 0
f1 (ppm)

3i

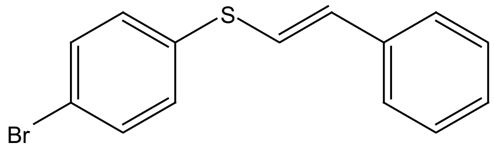

(^{1}H-NMR, 400 MHz, CDCl$_3$)

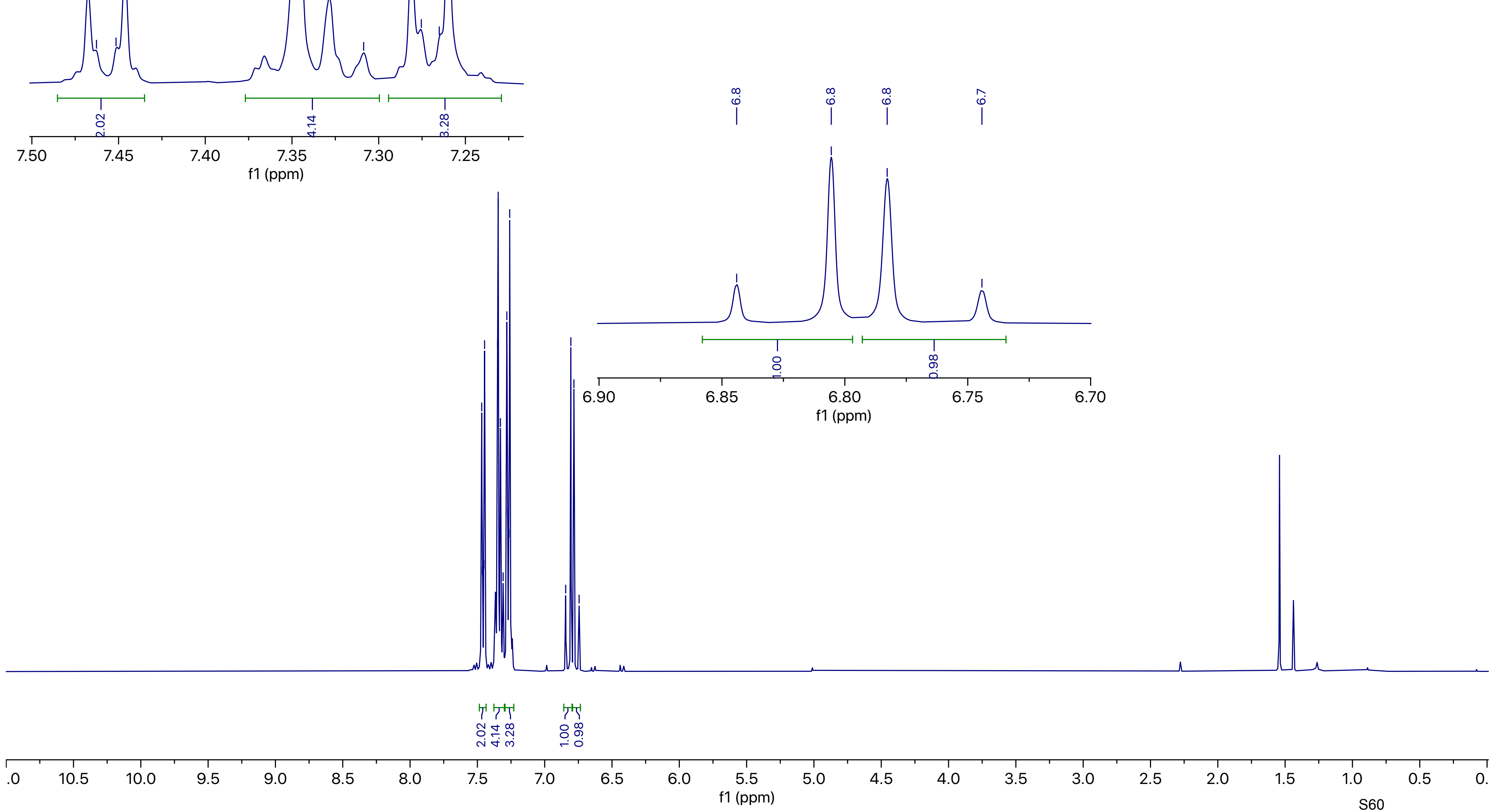

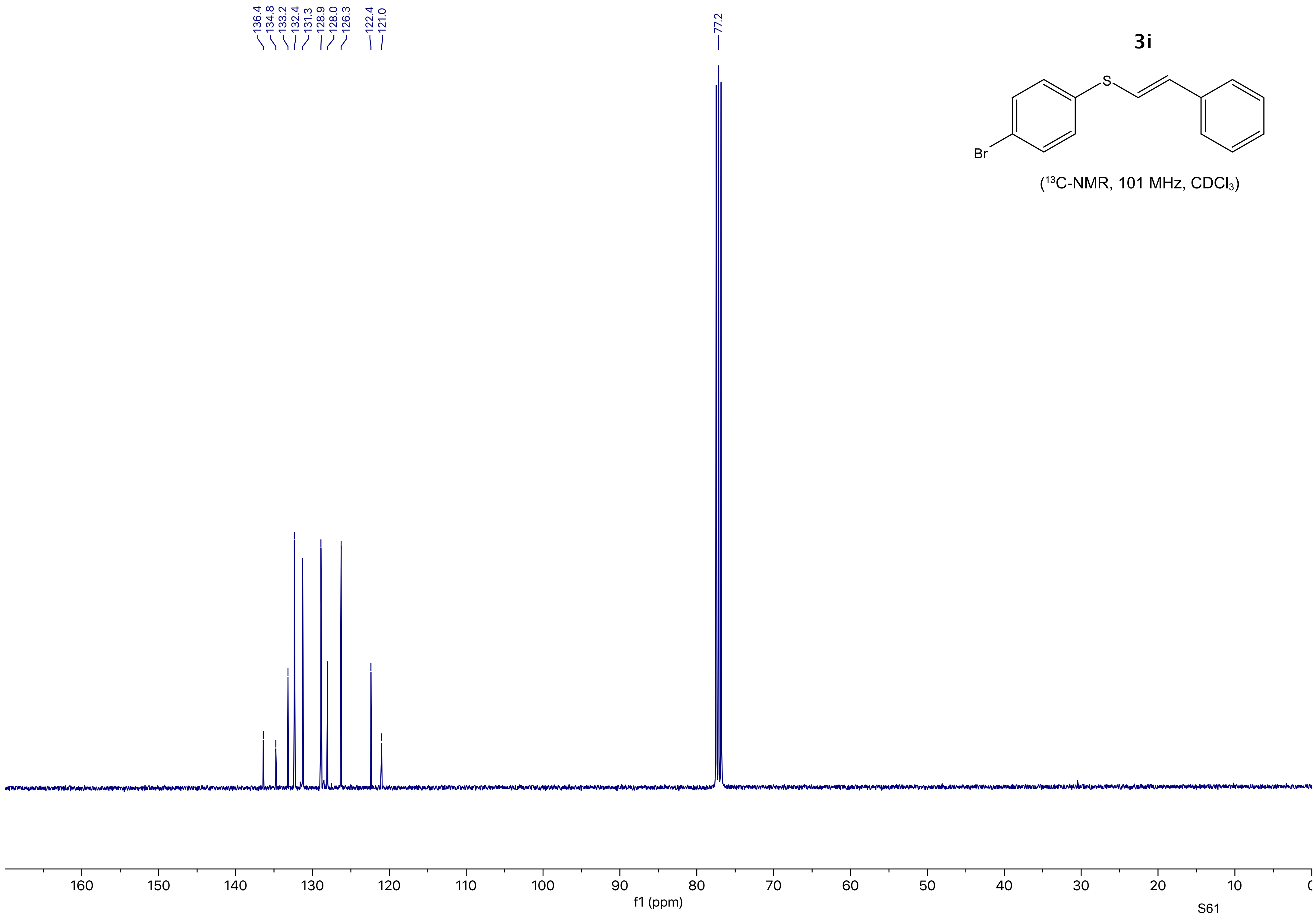
136.4
134.8
133.2
132.4
131.3
128.9
128.0
126.3
122.4
121.0
77.2
3i
S
Br
(13C-NMR, 101 MHz, CDCl3)
160
150
140
130
120
110
100
90
80
70
60
50
40
30
20
10
f1 (ppm)

3j

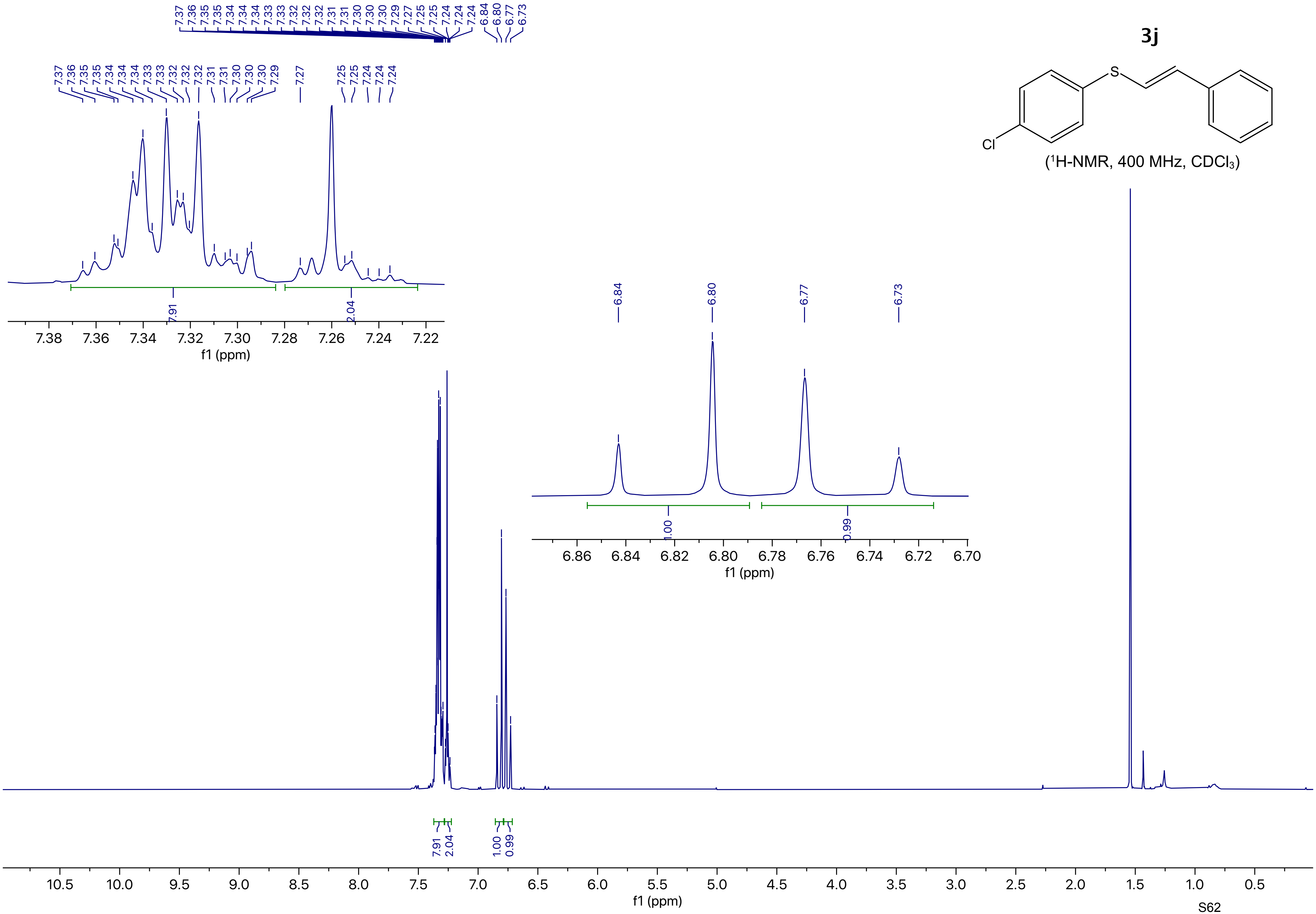

(^{1}H-NMR, 400 MHz, $CDCl_3$)

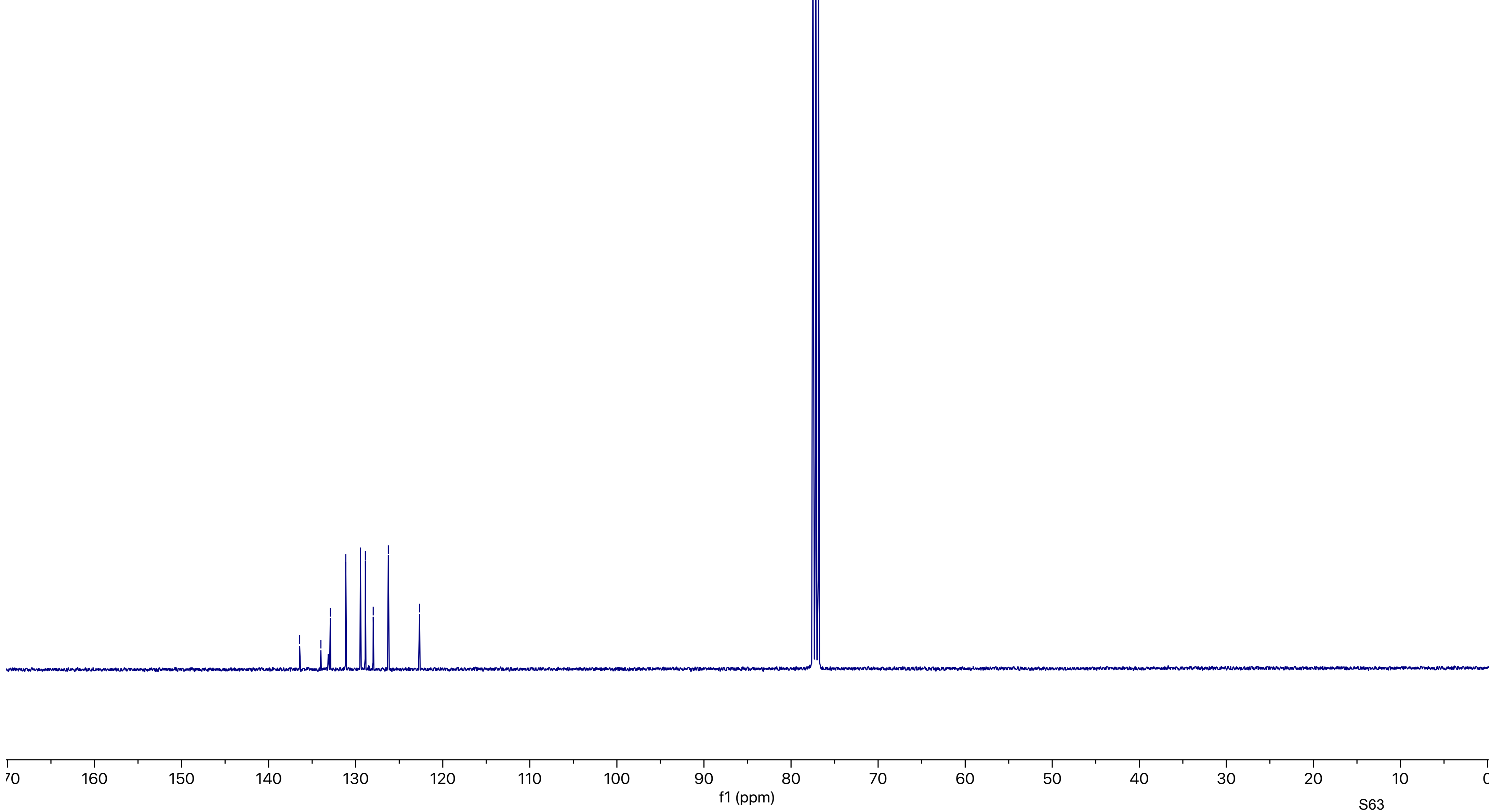

3j

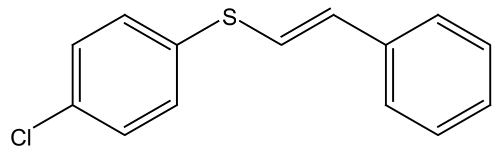

(^{13}C-NMR, 101 MHz, $CDCl_3$)

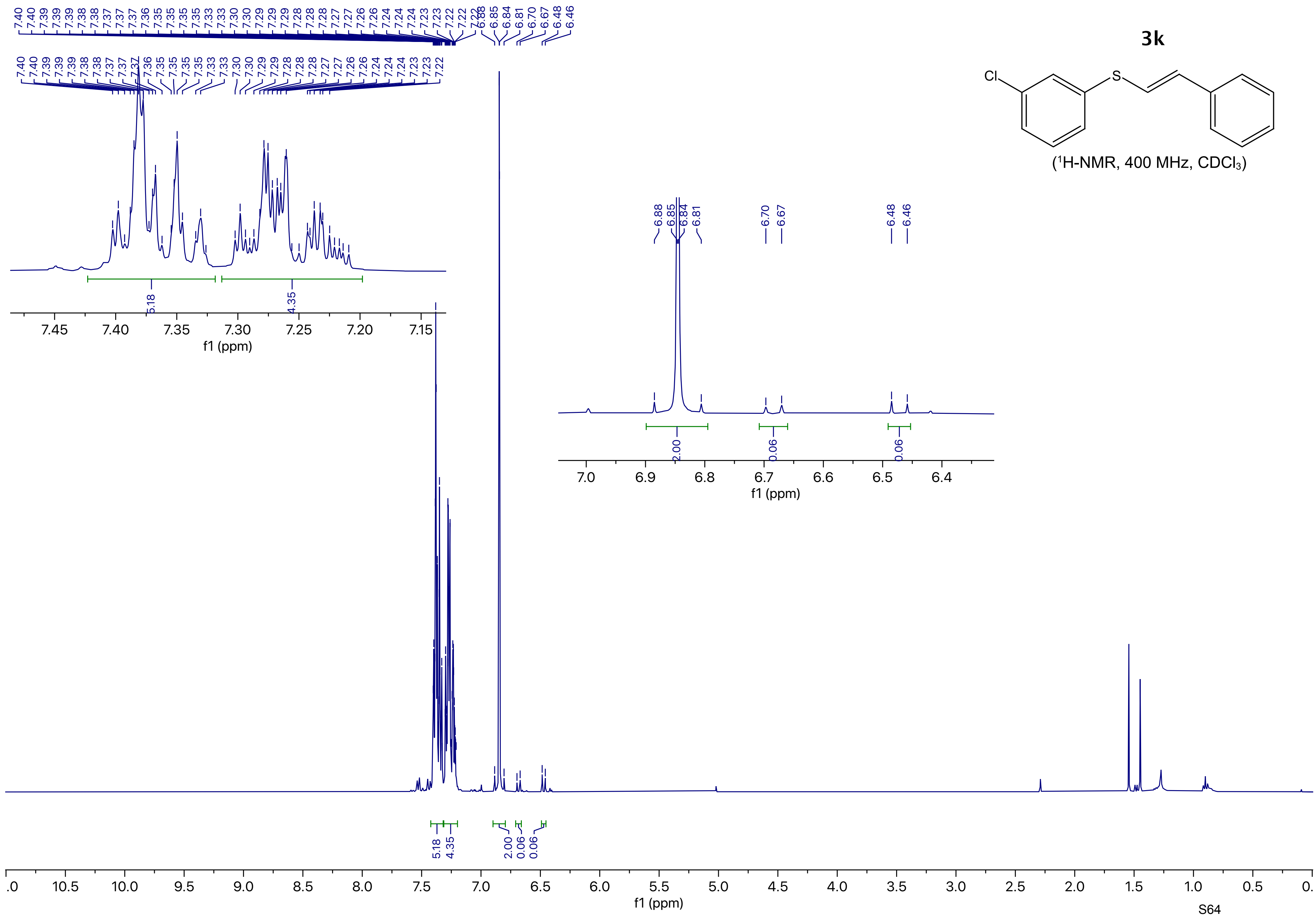
3k
Cl
S
(¹H-NMR, 400 MHz, CDCl₃)
f1 (ppm)

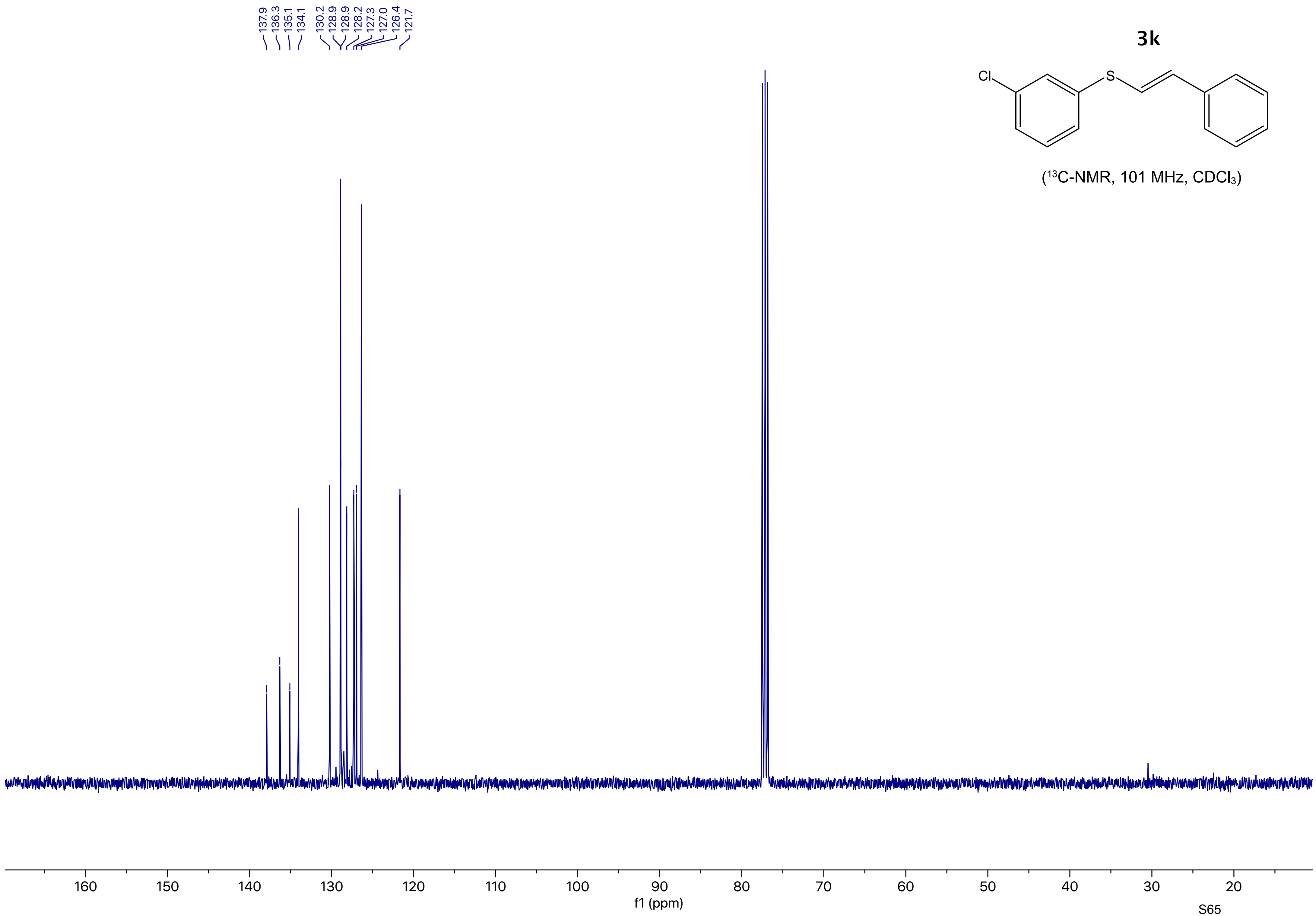
137.9
136.3
135.1
134.1
130.2
128.9
128.9
128.2
127.3
127.0
126.4
121.7
3k
Cl
S
(13C-NMR, 101 MHz, CDCl3)
160
150
140
130
120
110
100
90
80
70
60
50
40
30
20
f1 (ppm)

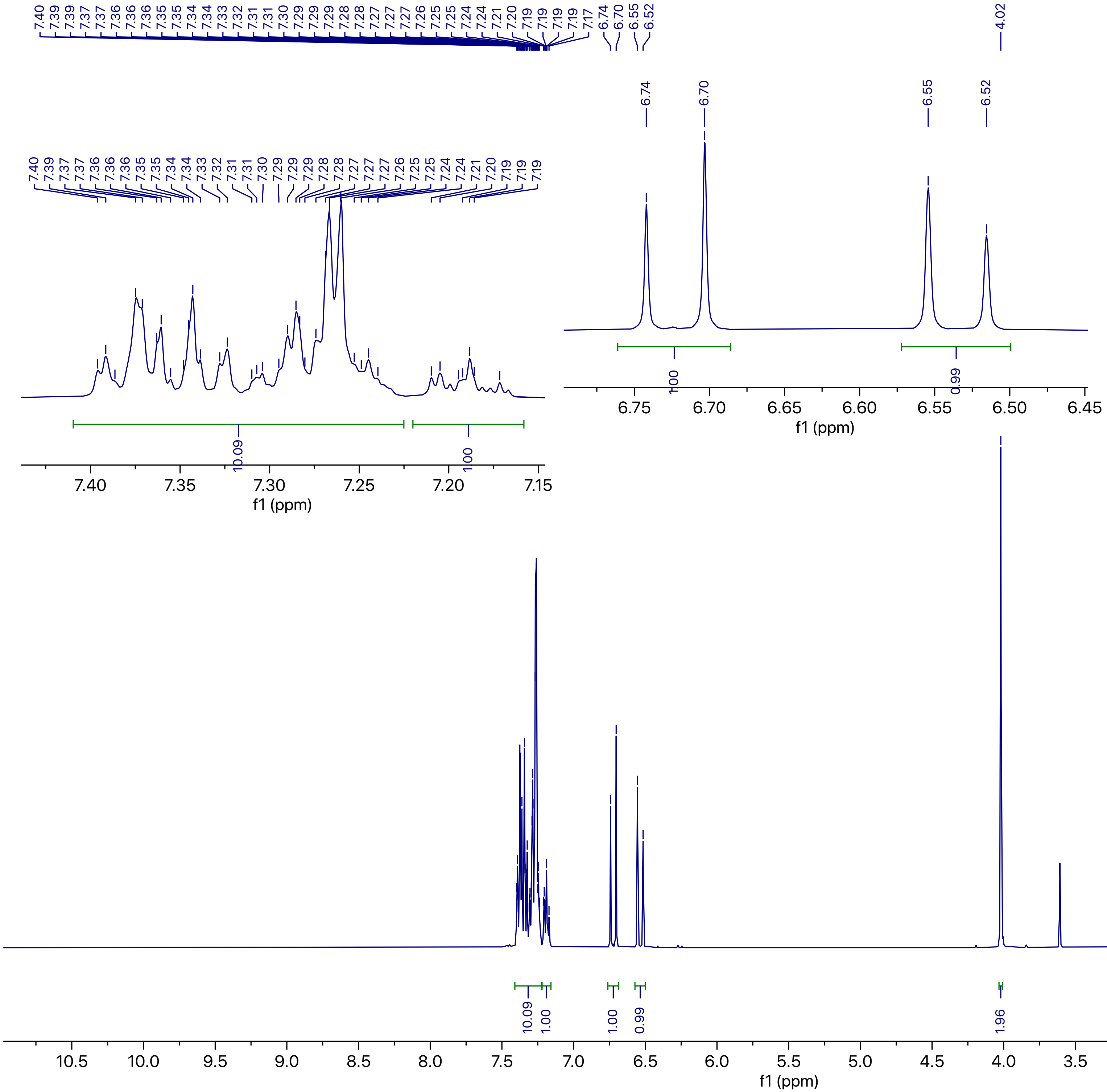

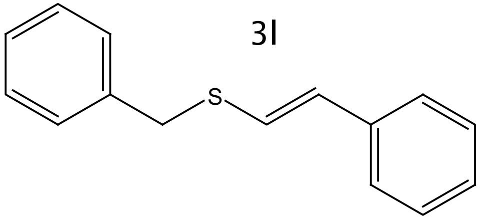

3l

(^{1}H-NMR, 400 MHz, $CDCl_3$)

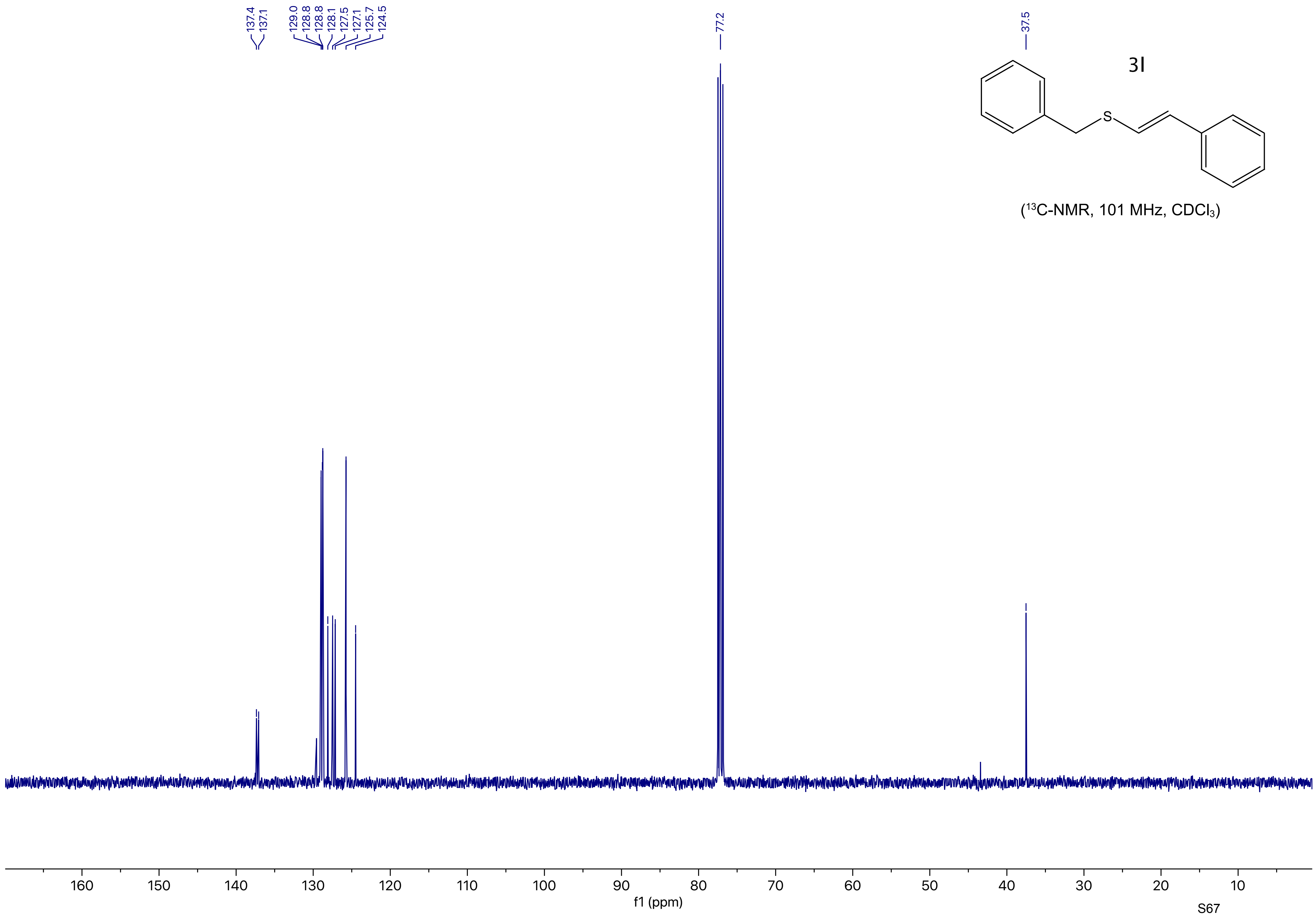
137.4
137.1
129.0
128.8
128.8
128.1
127.5
127.1
125.7
124.5
77.2
37.5
3l
S
(^{13}C-NMR, 101 MHz, $CDCl_3$)
160 150 140 130 120 110 100 90 80 70 60 50 40 30 20 10
f1 (ppm)

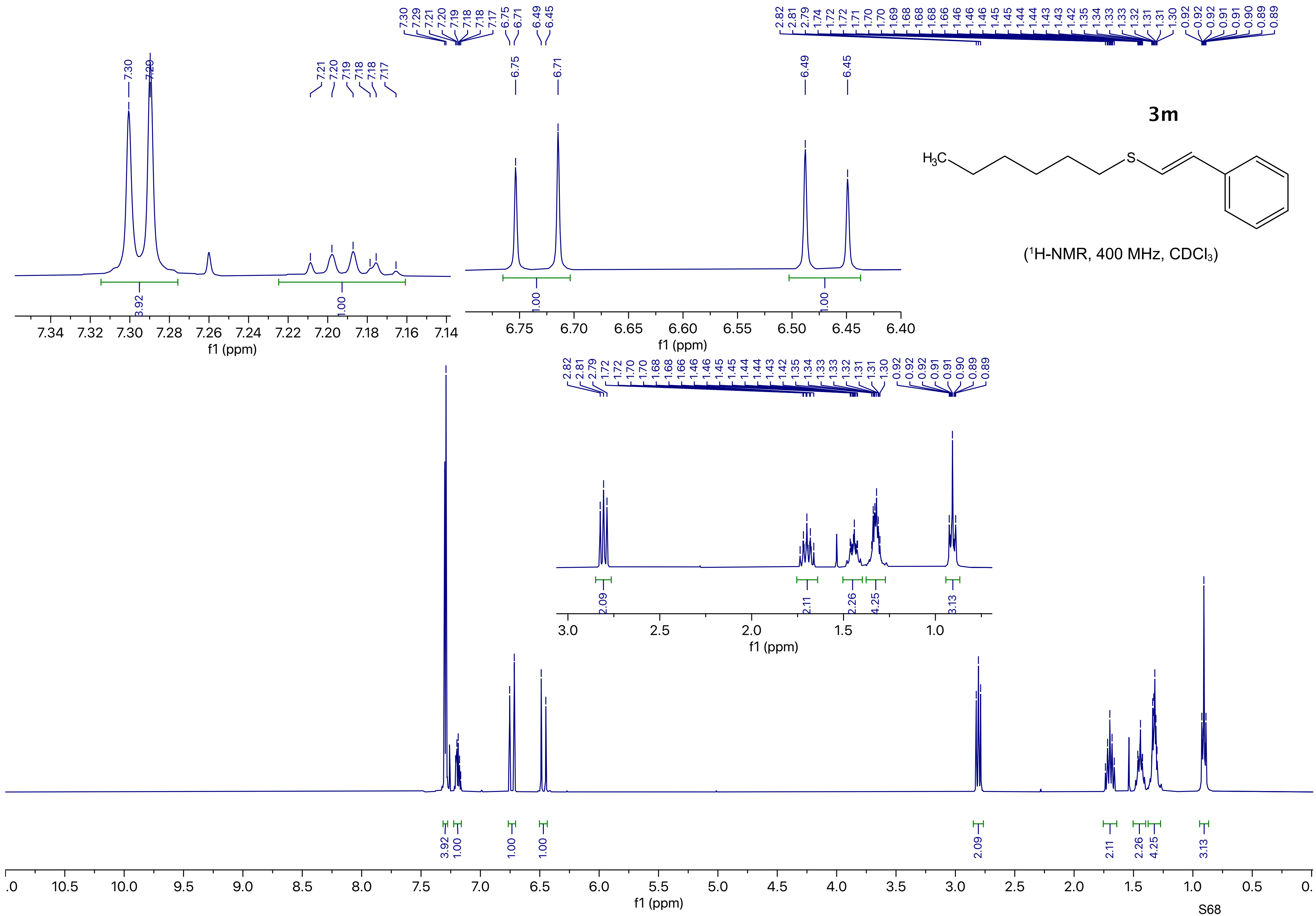

3m

(^{1}H-NMR, 400 MHz, $CDCl_3$)

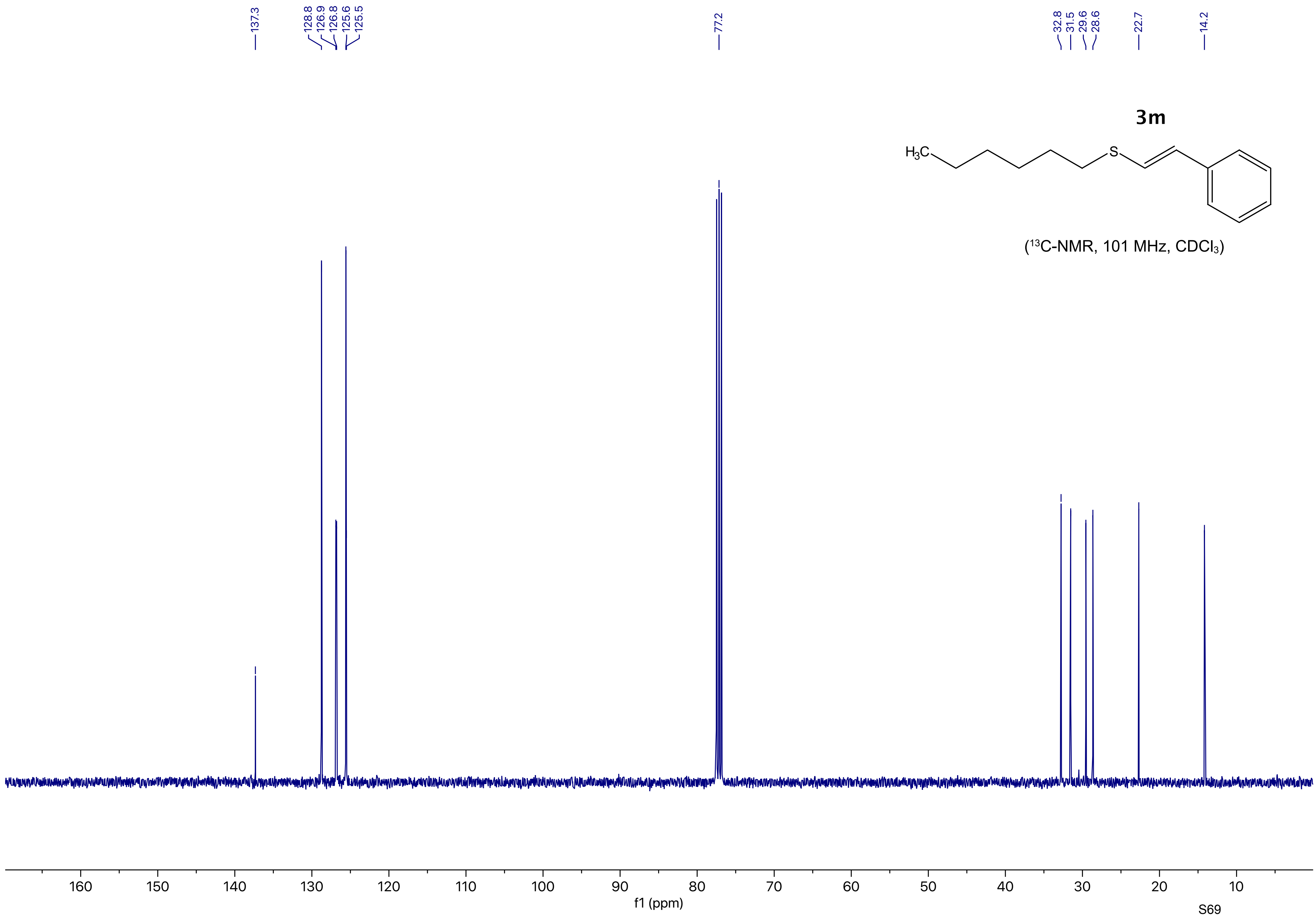

137.3
128.8
126.9
126.8
125.6
125.5
77.2
32.8
31.5
29.6
28.6
22.7
14.2
3m
H3C
S
(13C-NMR, 101 MHz, CDCl3)
160
150
140
130
120
110
100
90
80
70
60
50
40
30
20
10
f1 (ppm)

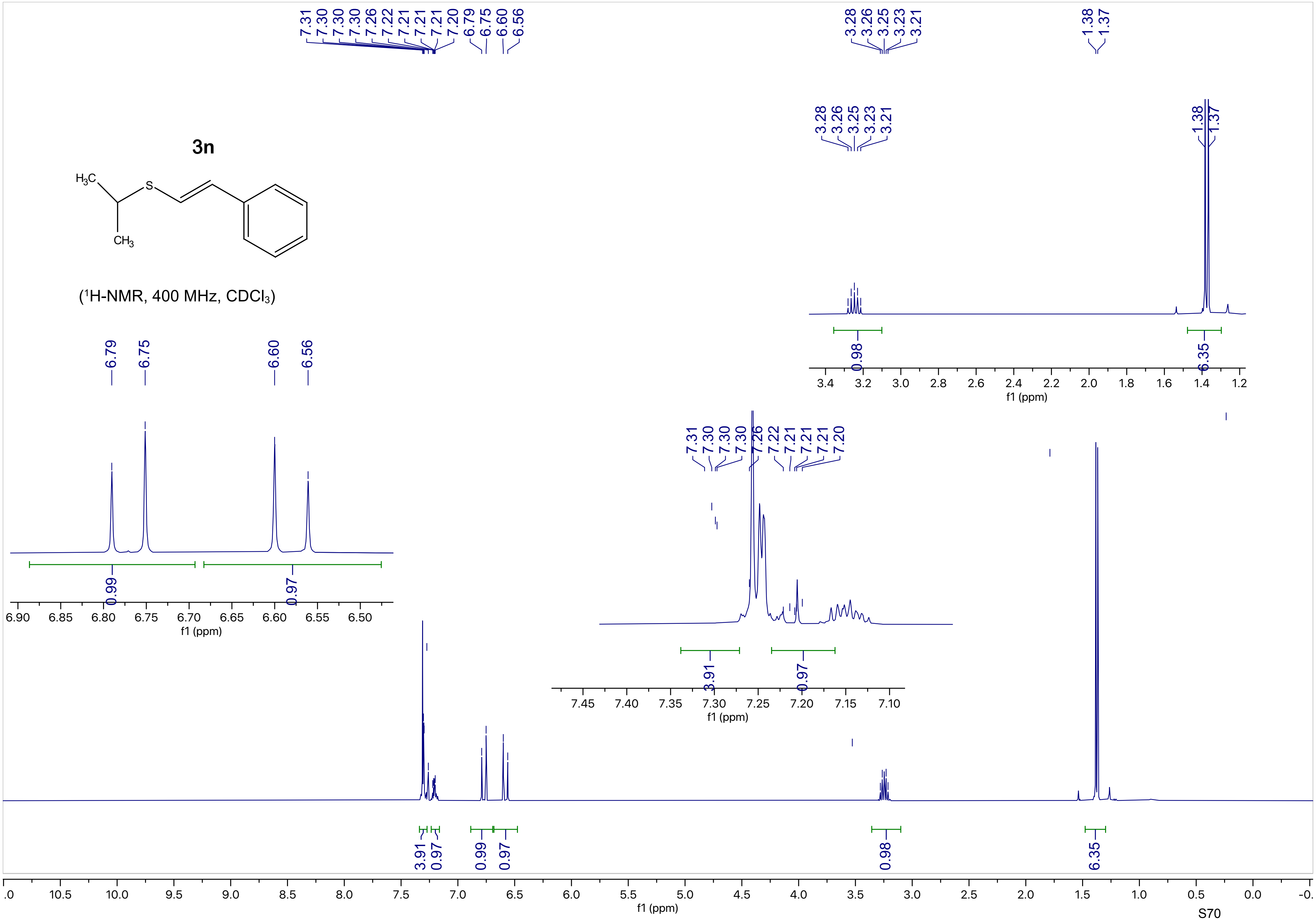

3n
H_3C
S
CH_3
(¹H-NMR, 400 MHz, $CDCl_3$)
7.31 7.30 7.30 7.30 7.26 7.22 7.21 7.21 7.21 7.20 6.79 6.75 6.60 6.56
3.28 3.26 3.25 3.23 3.21
1.38 1.37
3.91 0.97 0.99 0.97 0.98 6.35
f1 (ppm)

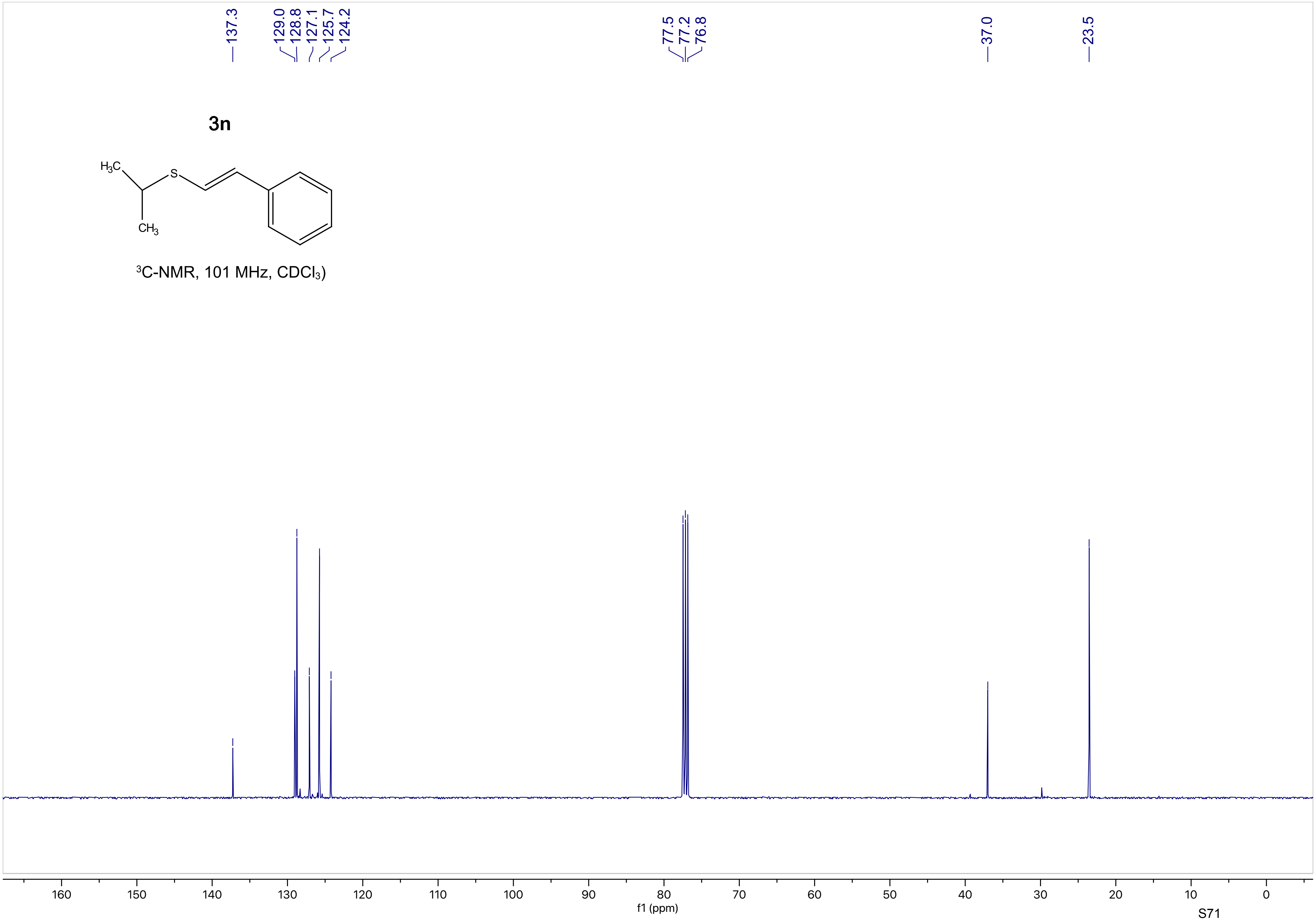
137.3
129.0
128.8
127.1
125.7
124.2
77.5
77.2
76.8
37.0
23.5
3n
H3C
S
CH3
³C-NMR, 101 MHz, CDCl₃)
160 150 140 130 120 110 100 90 80 70 60 50 40 30 20 10 0
f1 (ppm)

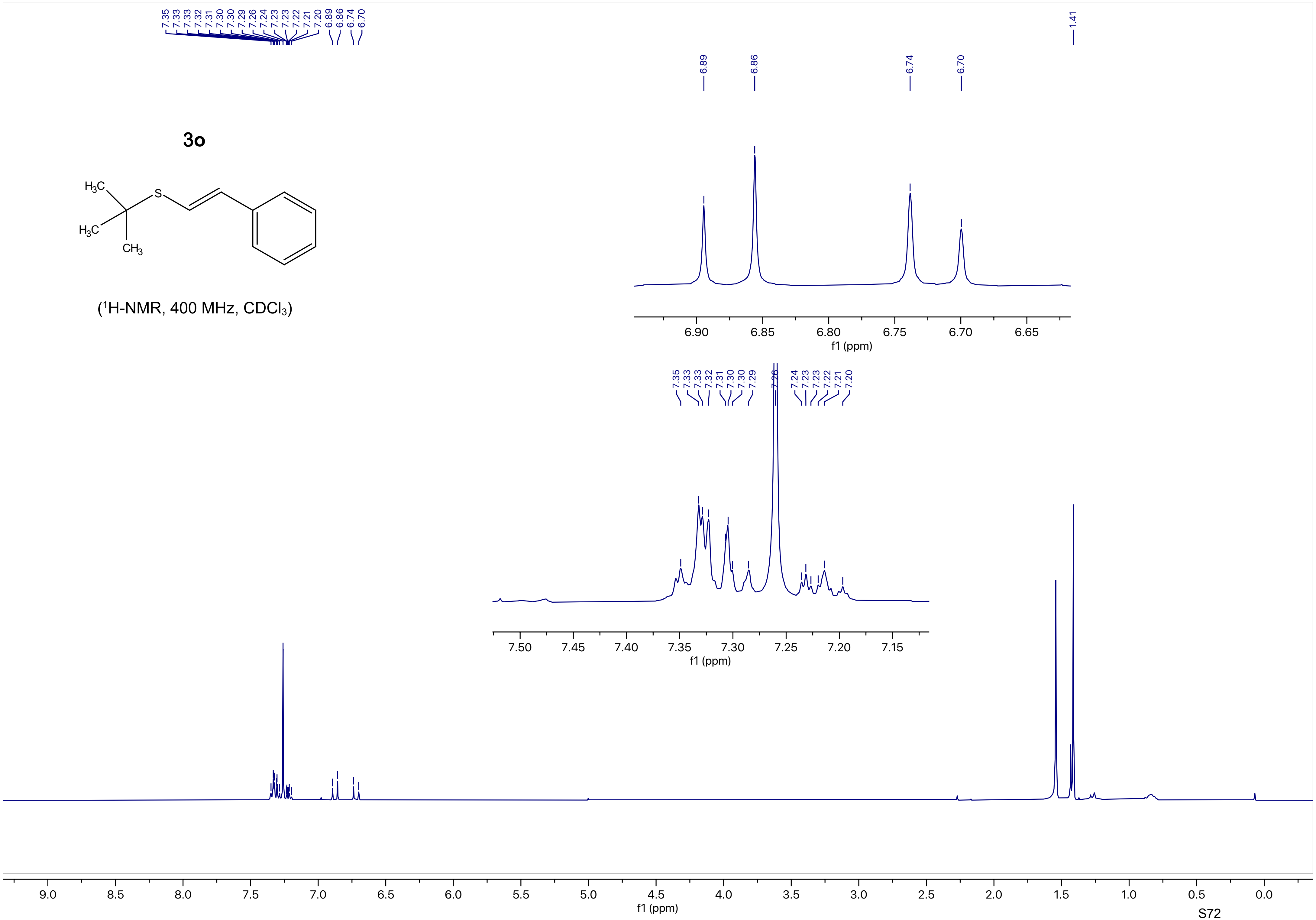
3o
H3C
S
H3C
CH3
(1H-NMR, 400 MHz, CDCl3)
7.35 7.33 7.33 7.32 7.31 7.30 7.30 7.29 7.26 7.24 7.23 7.23 7.22 7.21 7.20 6.89 6.86 6.74 6.70
1.41
6.89 6.86 6.74 6.70
6.90 6.85 6.80 6.75 6.70 6.65
f1 (ppm)
7.35 7.33 7.33 7.32 7.31 7.30 7.30 7.29 7.26 7.24 7.23 7.23 7.22 7.21 7.20
7.50 7.45 7.40 7.35 7.30 7.25 7.20 7.15
f1 (ppm)
9.0 8.5 8.0 7.5 7.0 6.5 6.0 5.5 5.0 4.5 4.0 3.5 3.0 2.5 2.0 1.5 1.0 0.5 0.0
f1 (ppm)

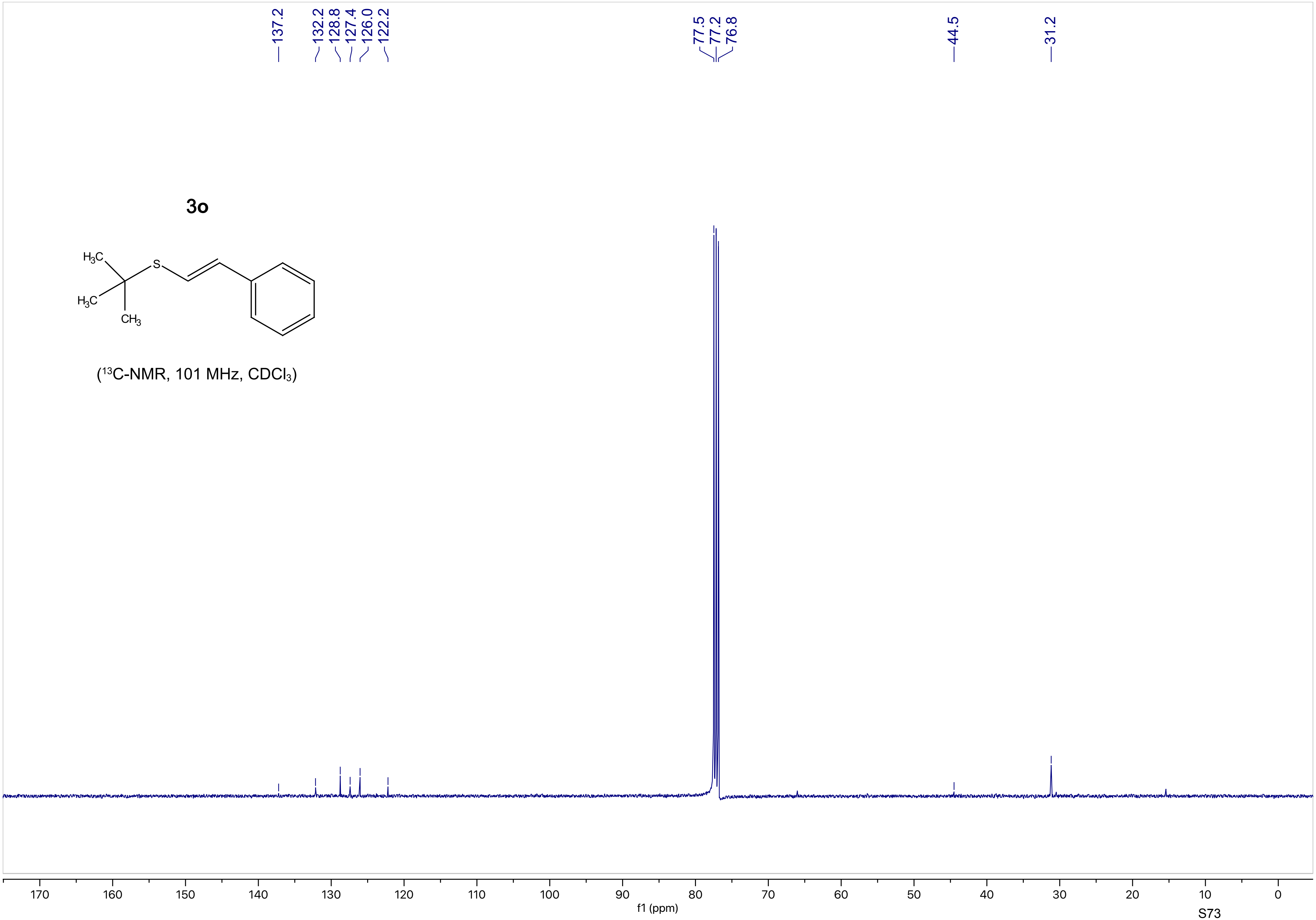
137.2
132.2
128.8
127.4
126.0
122.2
77.5
77.2
76.8
44.5
31.2
3o
H3C
S
H3C
CH3
(13C-NMR, 101 MHz, CDCl3)
170
160
150
140
130
120
110
100
90
80
70
60
50
40
30
20
10
0
f1 (ppm)

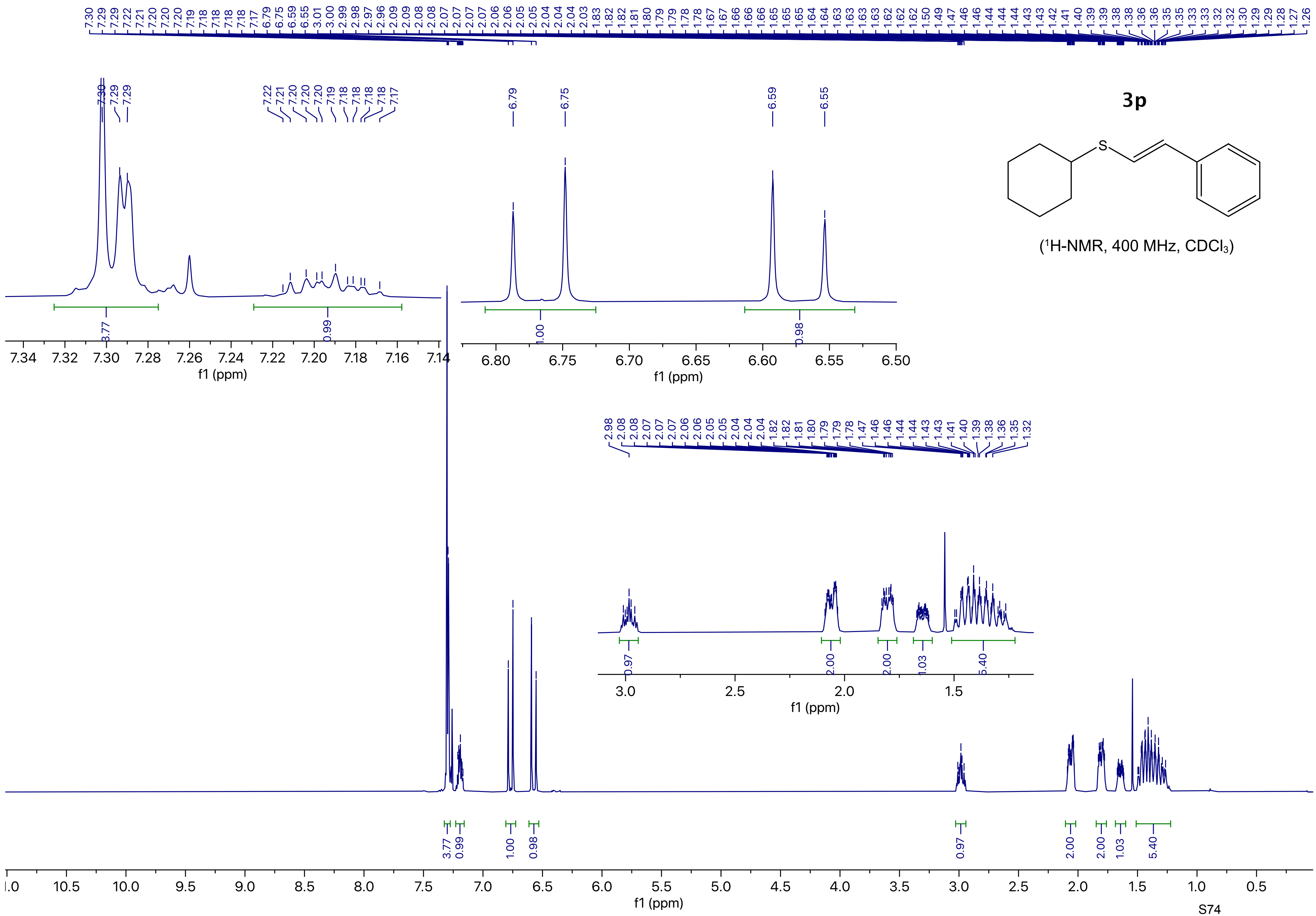

3p

(^{1}H-NMR, 400 MHz, $CDCl_3$)

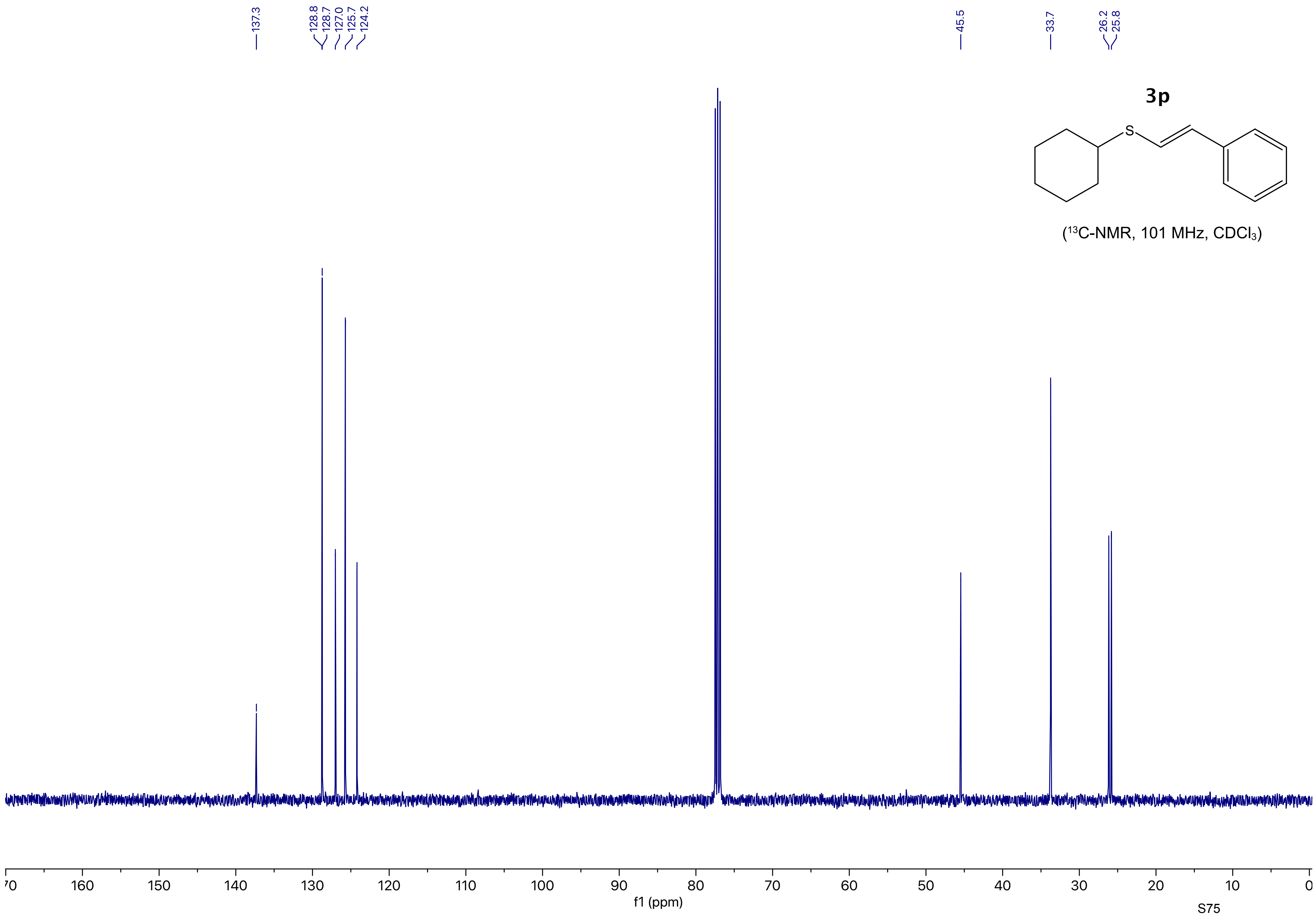
137.3
128.8
128.7
127.0
125.7
124.2
45.5
33.7
26.2
25.8
3p
S
(^{13}C-NMR, 101 MHz, $CDCl_3$)
70
160
150
140
130
120
110
100
90
80
70
60
50
40
30
20
10
0
f1 (ppm)

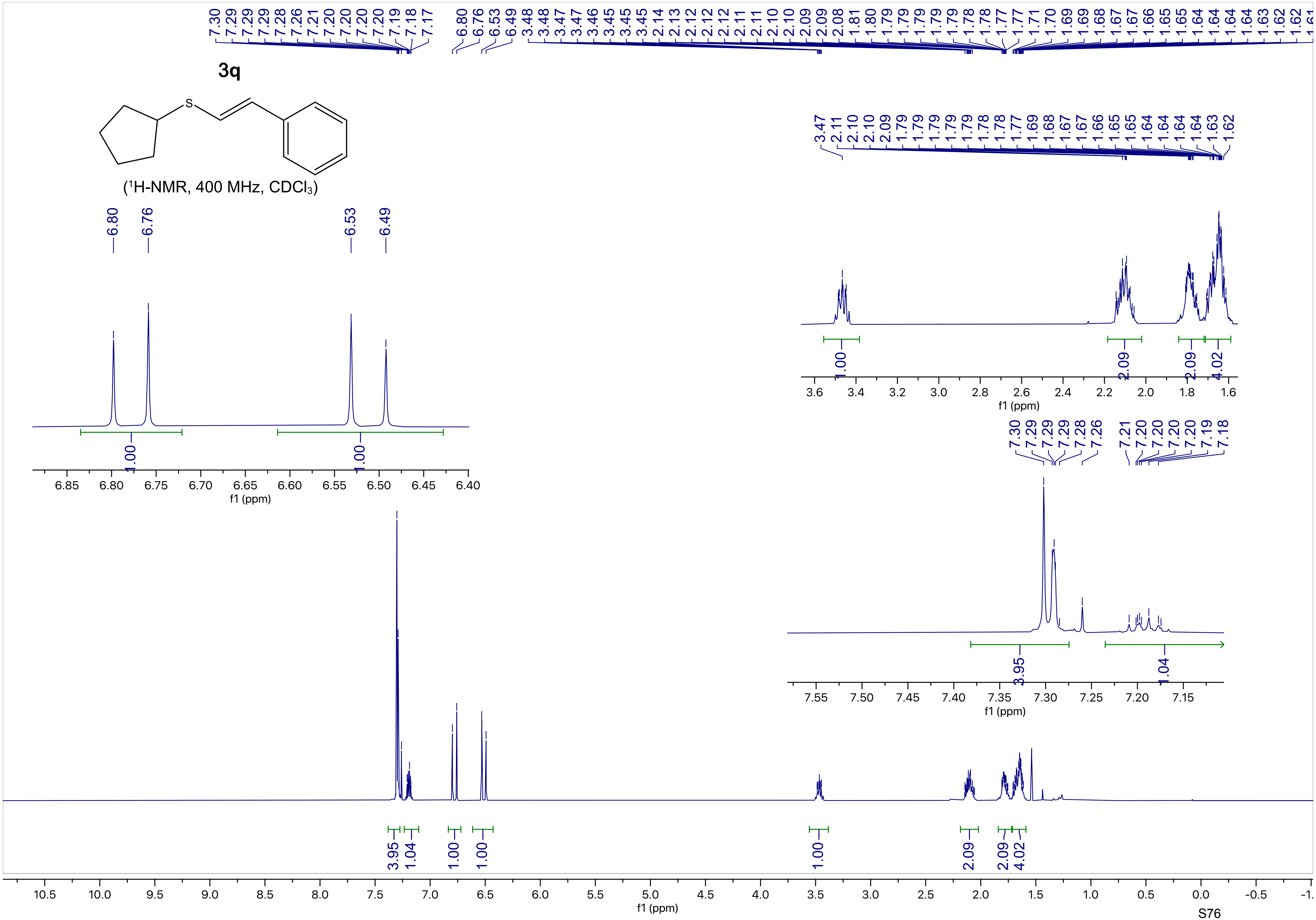
3q
S
(1H-NMR, 400 MHz, CDCl3)
f1 (ppm)

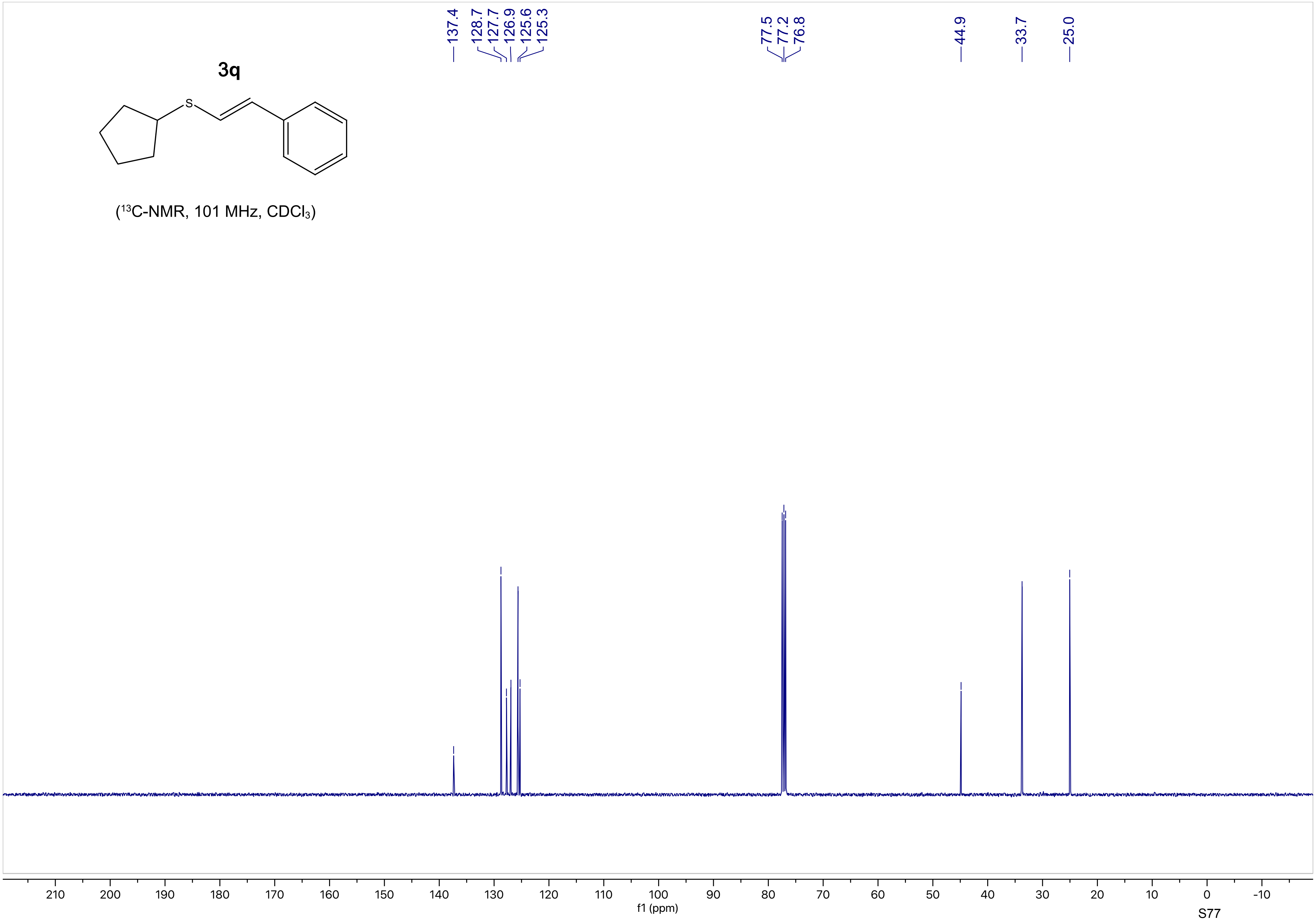
3q
S
(13C-NMR, 101 MHz, CDCl3)
137.4
128.7
127.7
126.9
125.6
125.3
77.5
77.2
76.8
44.9
33.7
25.0
210
200
190
180
170
160
150
140
130
120
110
100
90
80
70
60
50
40
30
20
10
0
-10
f1 (ppm)

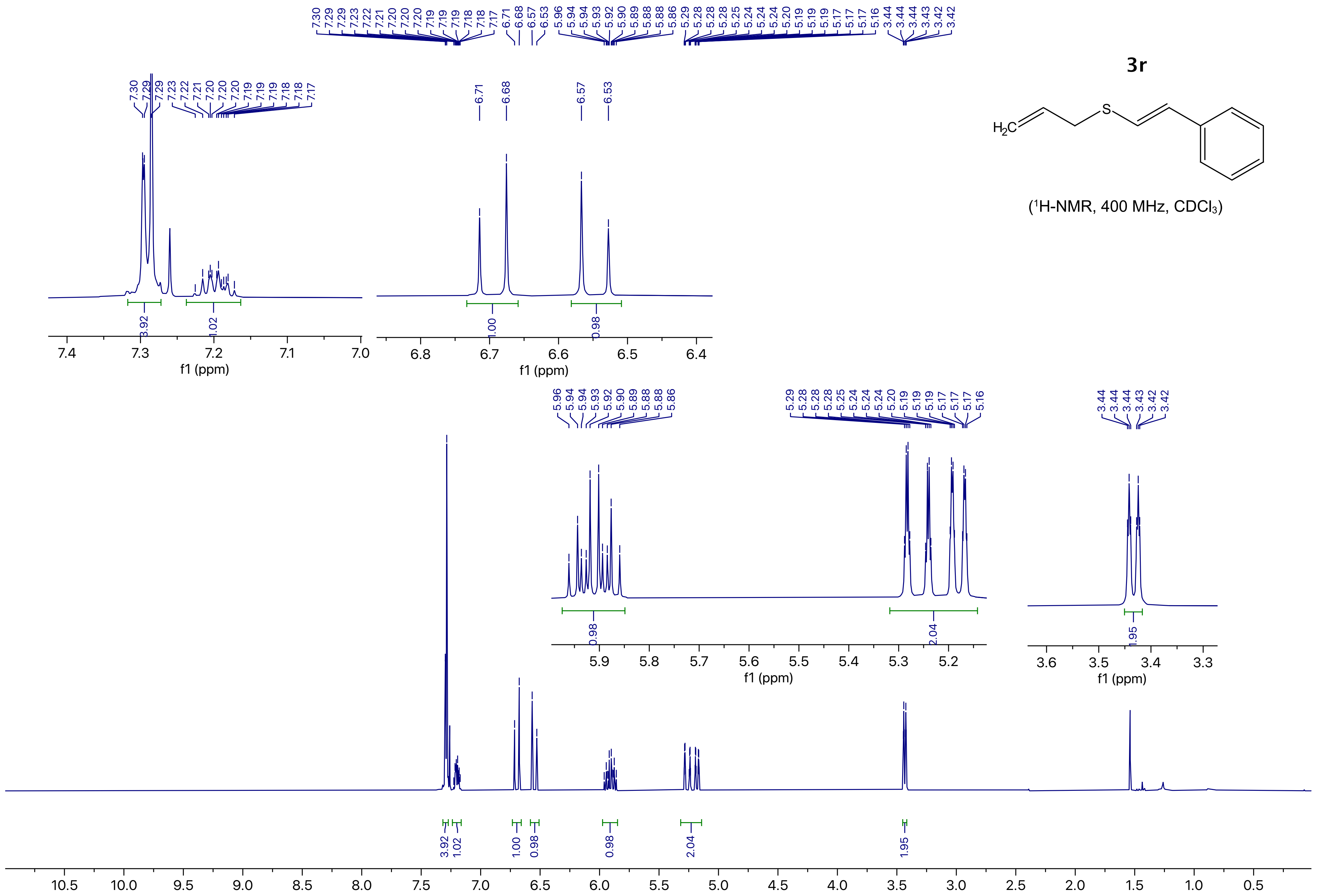
3r
H2C
S
(1H-NMR, 400 MHz, CDCl3)
f1 (ppm)
3.92
1.02
1.00
0.98
0.98
2.04
1.95

3r

(^{13}C-NMR, 101 MHz, CDCl$_3$)

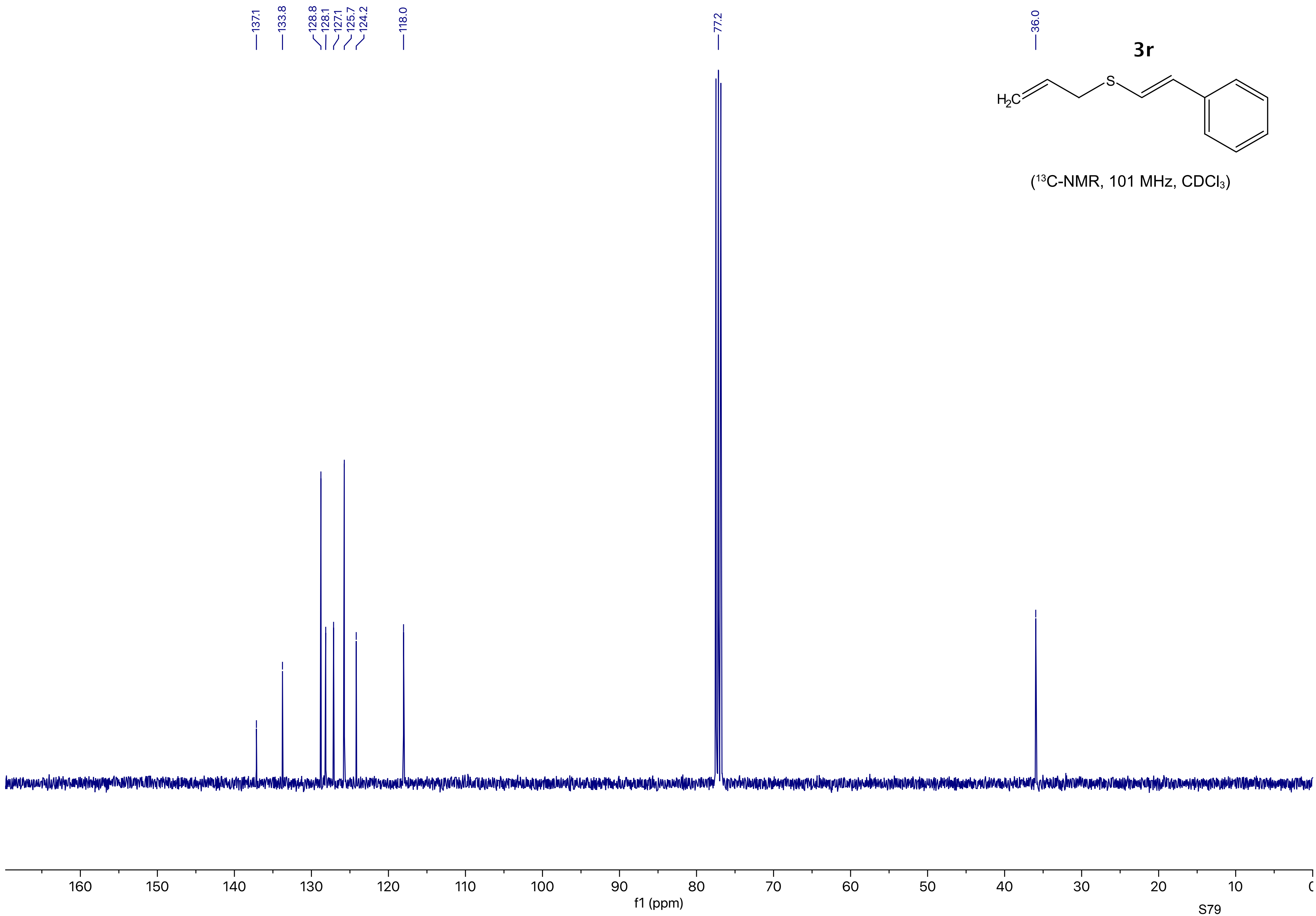

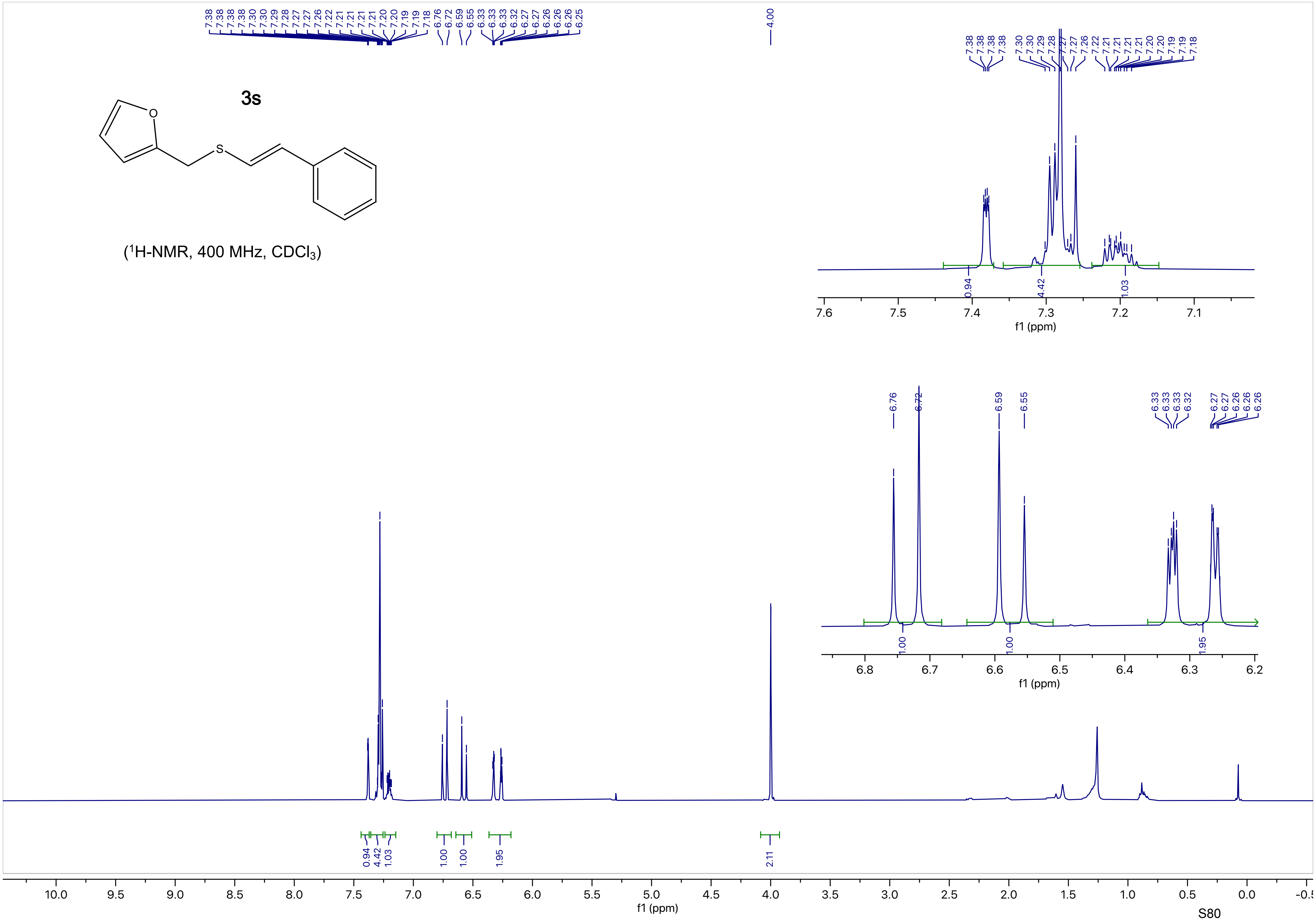
3s
(^{1}H-NMR, 400 MHz, CDCl$_3$)
f1 (ppm)

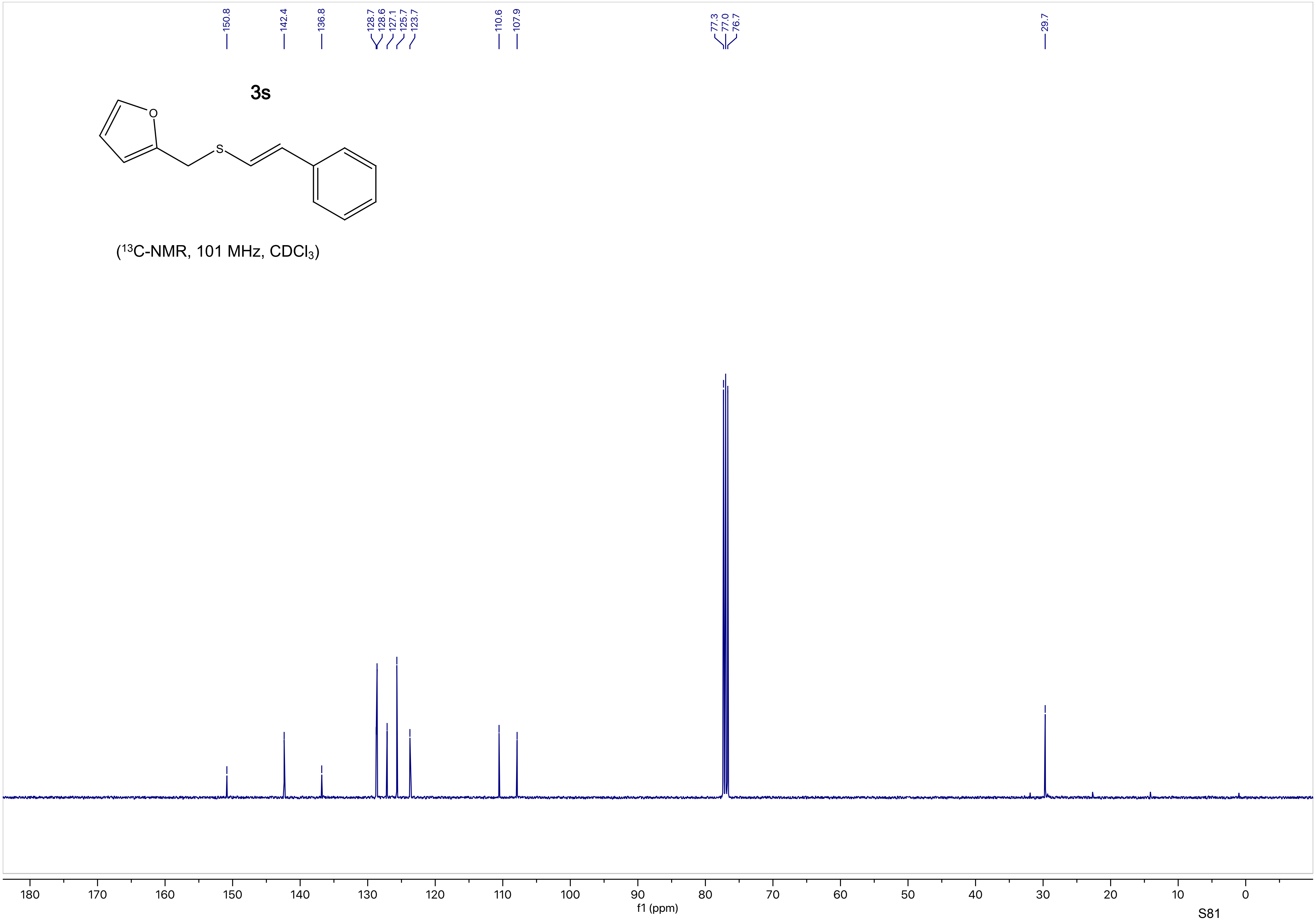
3s
(13C-NMR, 101 MHz, CDCl3)
150.8
142.4
136.8
128.7
128.6
127.1
125.7
123.7
110.6
107.9
77.3
77.0
76.7
29.7
180 170 160 150 140 130 120 110 100 90 80 70 60 50 40 30 20 10 0
f1 (ppm)

3t (*E*)

(^{1}H-NMR, 400 MHz, $CDCl_3$)

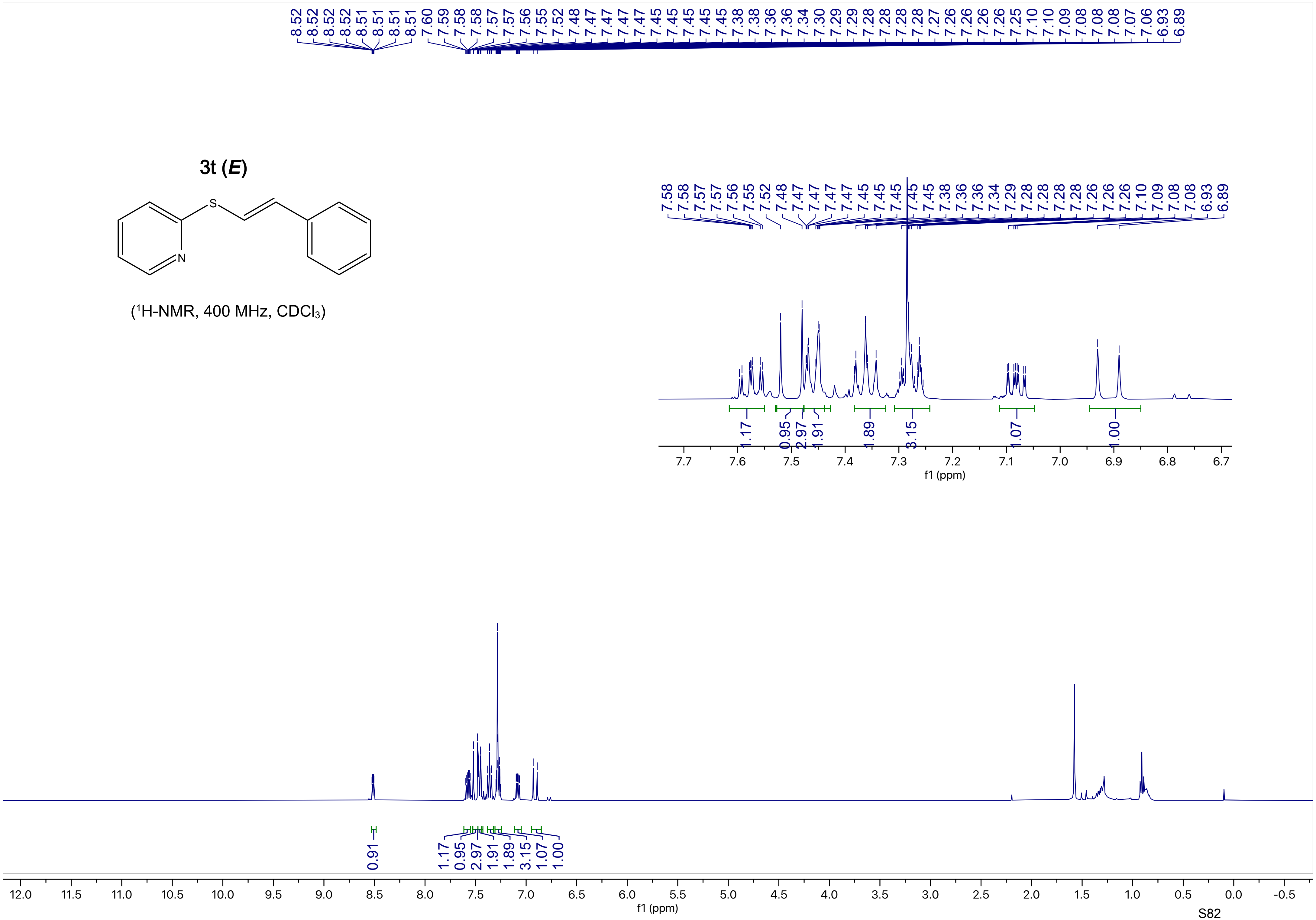

3t (*E*)

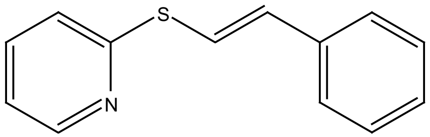

(^{13}C-NMR, 101 MHz, $CDCl_3$)

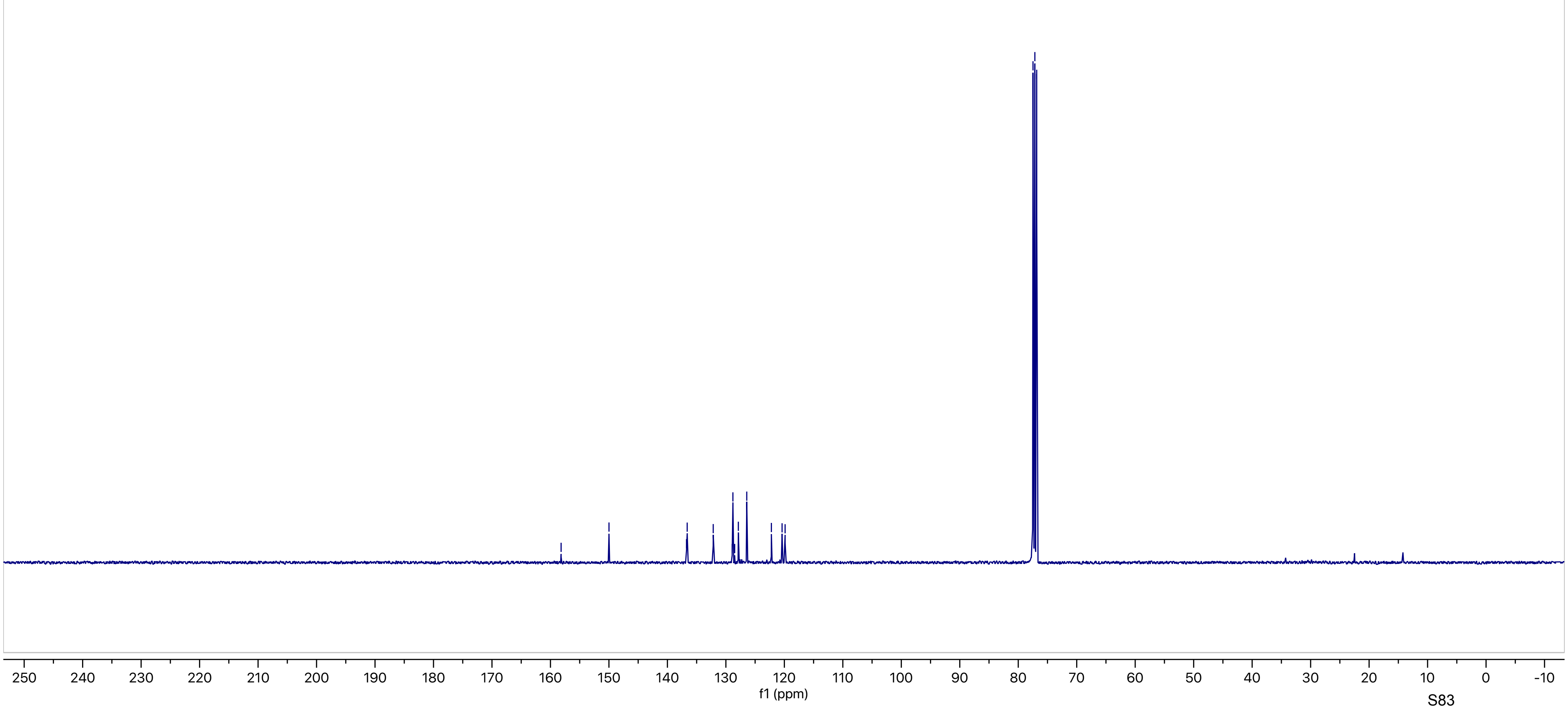

3t (*Z*)

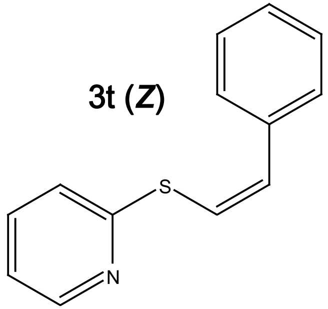

(^{1}H-NMR, 400 MHz, $CDCl_3$)

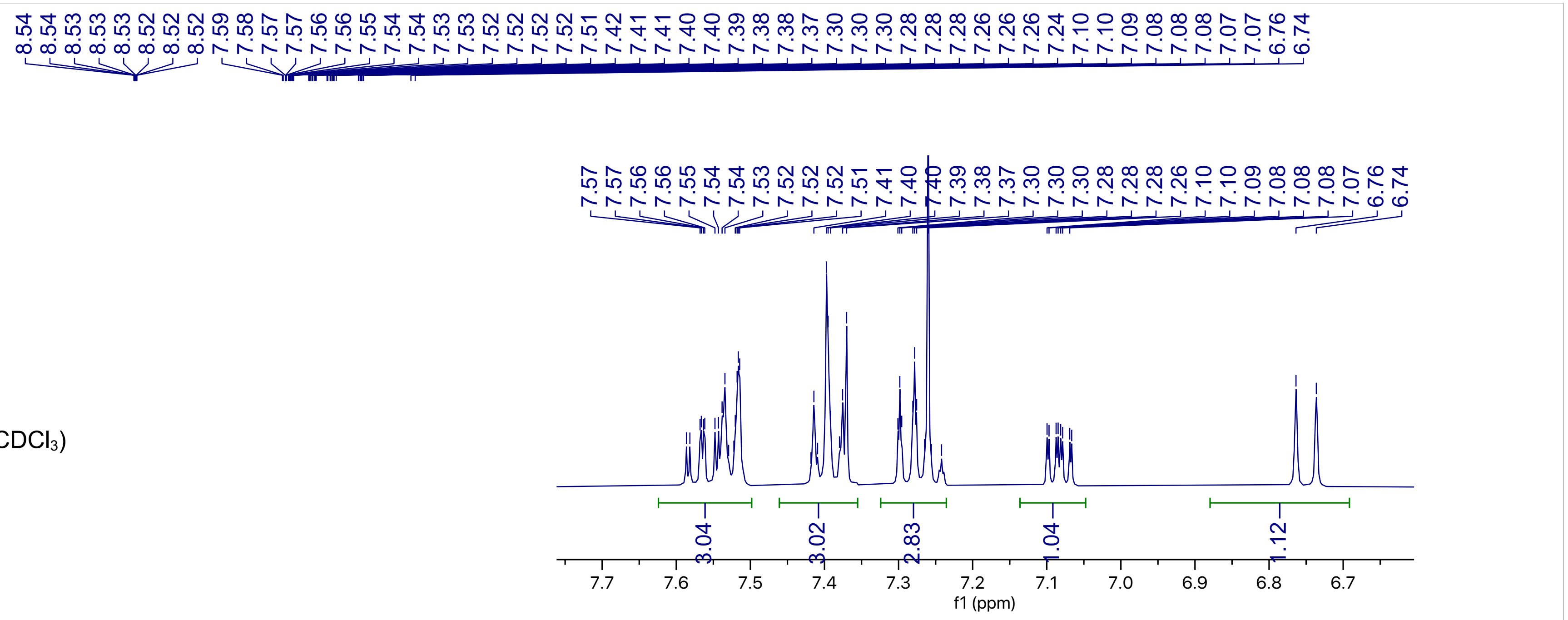

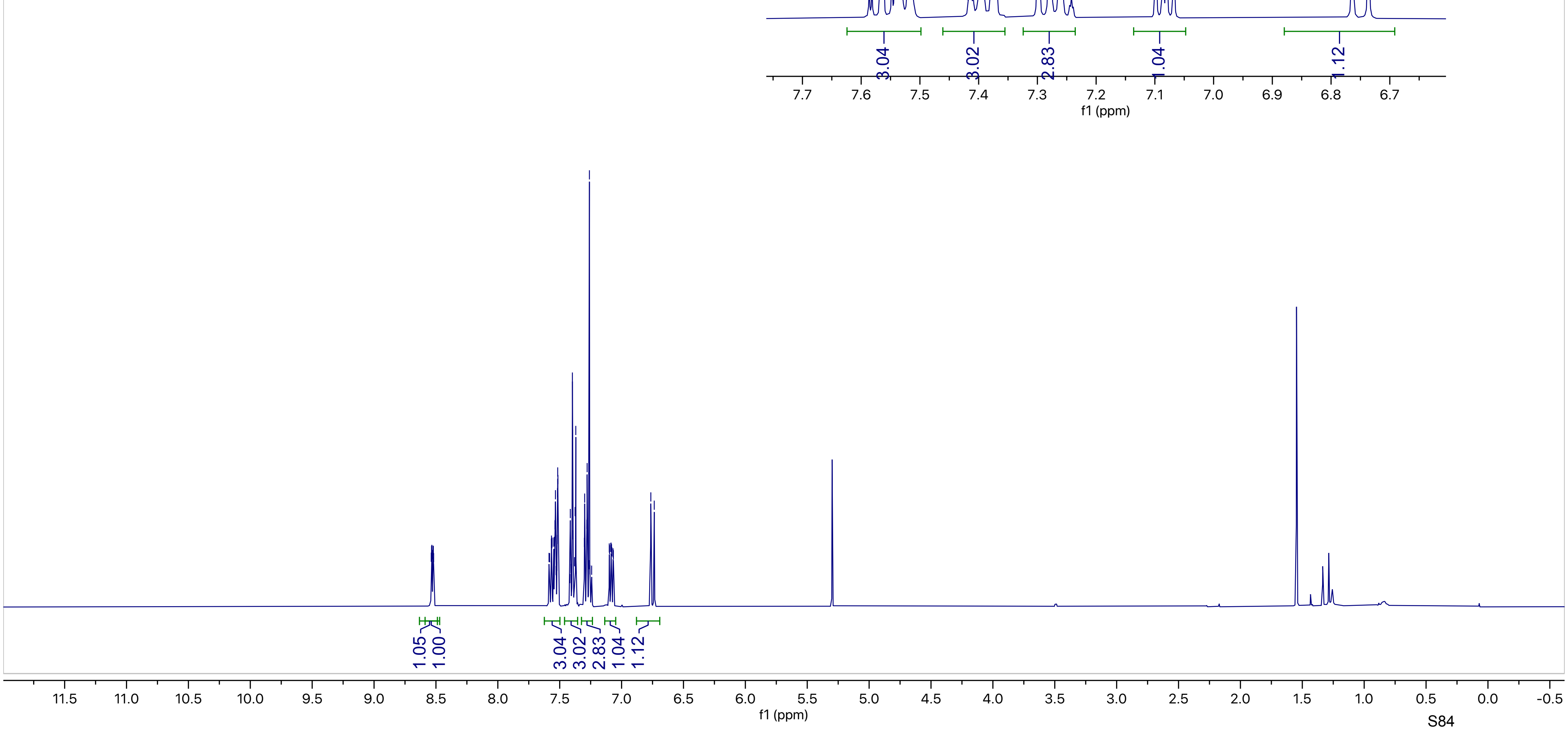

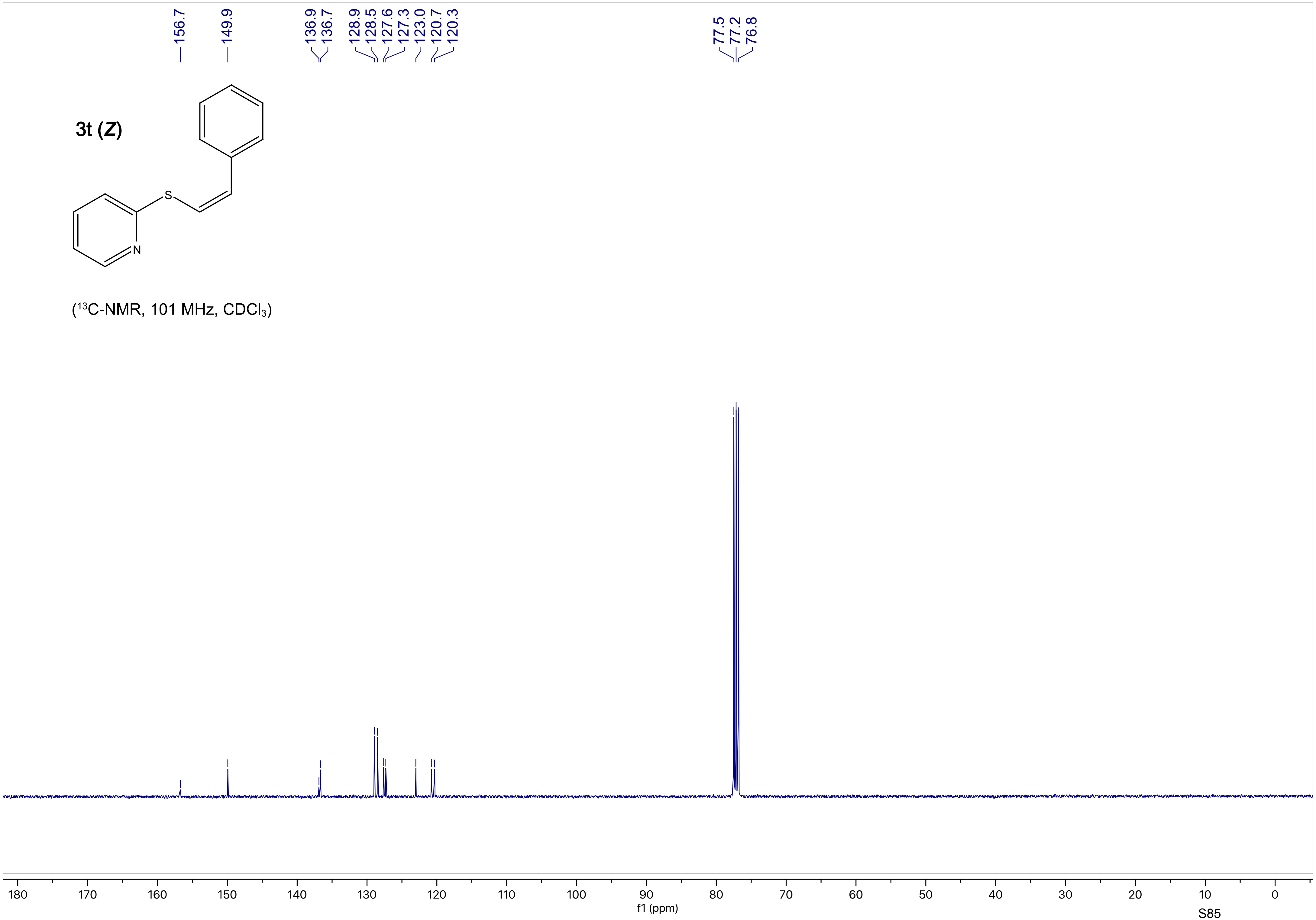
156.7
149.9
136.9
136.7
128.9
128.5
127.6
127.3
123.0
120.7
120.3
77.5
77.2
76.8
3t (Z)
S
N
(13C-NMR, 101 MHz, CDCl3)
180
170
160
150
140
130
120
110
100
90
80
70
60
50
40
30
20
10
0
f1 (ppm)

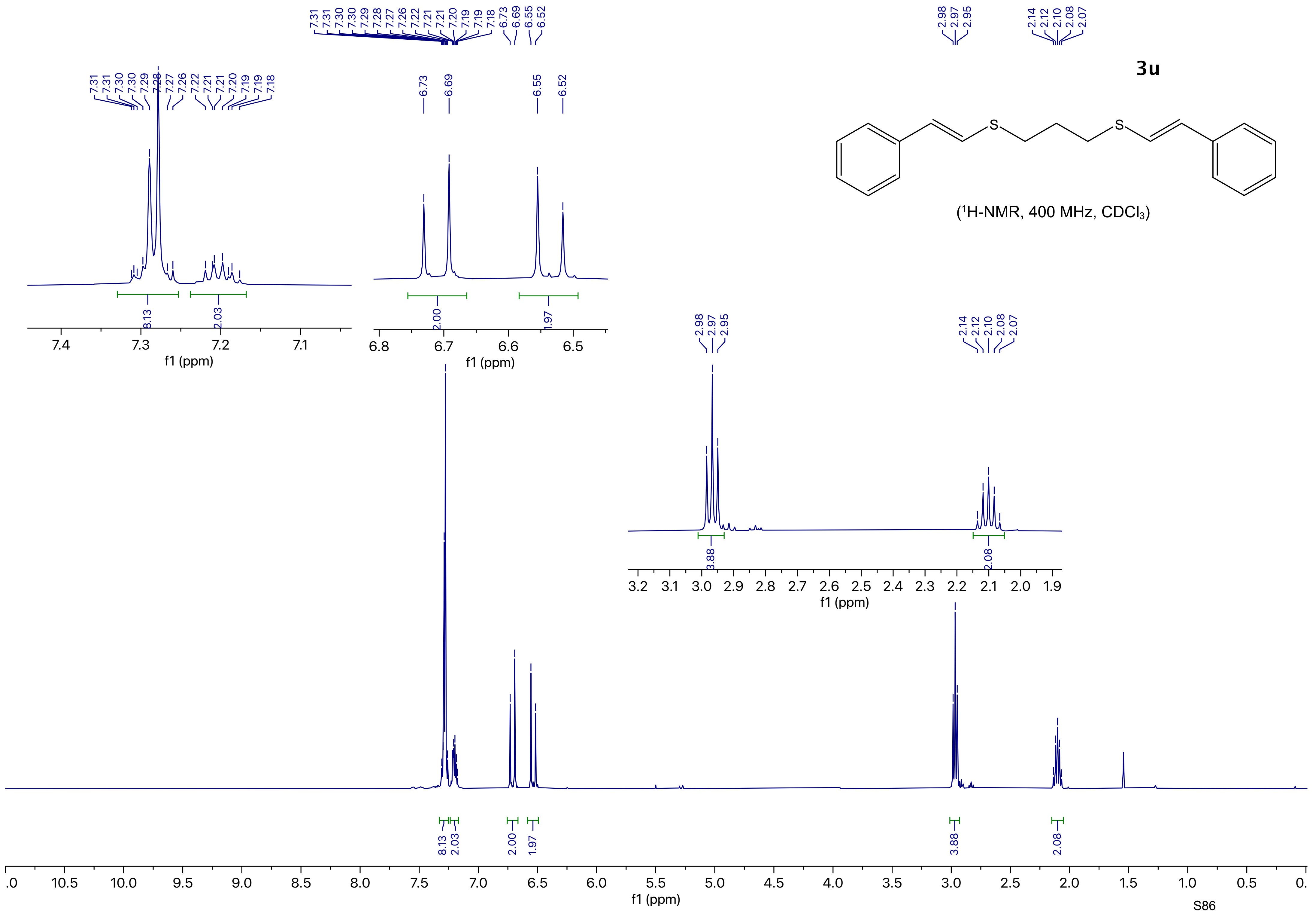

3u
S
S
(1H-NMR, 400 MHz, CDCl3)
7.31 7.31 7.30 7.30 7.29 7.28 7.27 7.26 7.22 7.21 7.21 7.20 7.19 7.19 7.18 6.73 6.69 6.55 6.52
2.98 2.97 2.95
2.14 2.12 2.10 2.08 2.07
8.13
2.03
2.00
1.97
3.88
2.08
f1 (ppm)

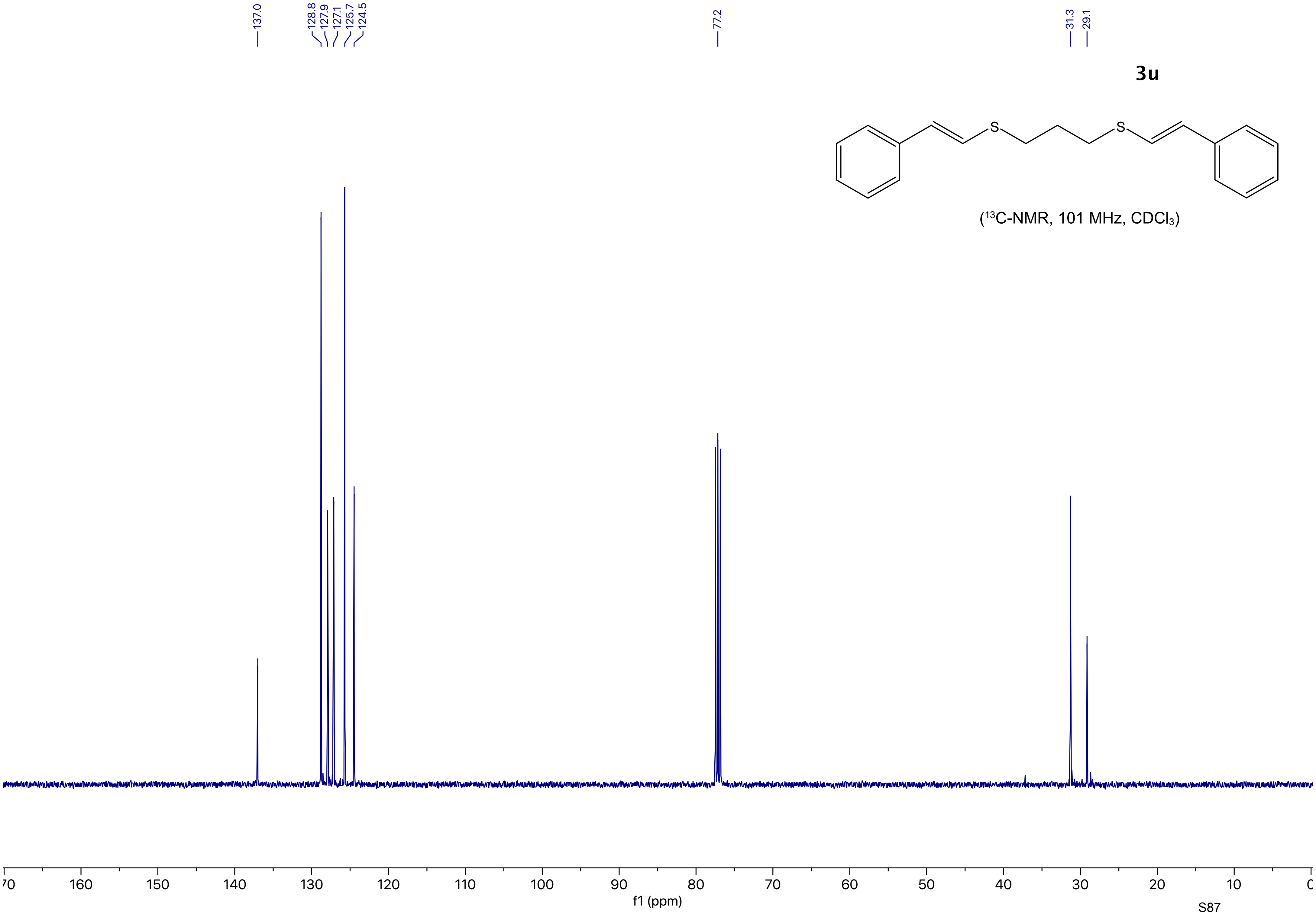
137.0
128.8
127.9
127.1
125.7
124.5
77.2
31.3
29.1
3u
S
S
(13C-NMR, 101 MHz, CDCl3)
70
160
150
140
130
120
110
100
90
80
70
60
50
40
30
20
10
0
f1 (ppm)

3v

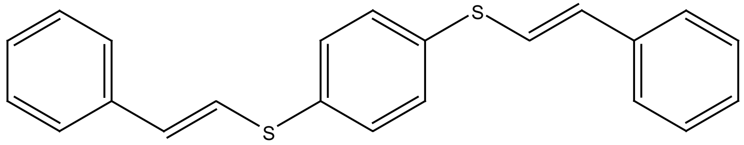

(^{1}H-NMR, 400 MHz, $CDCl_3$)

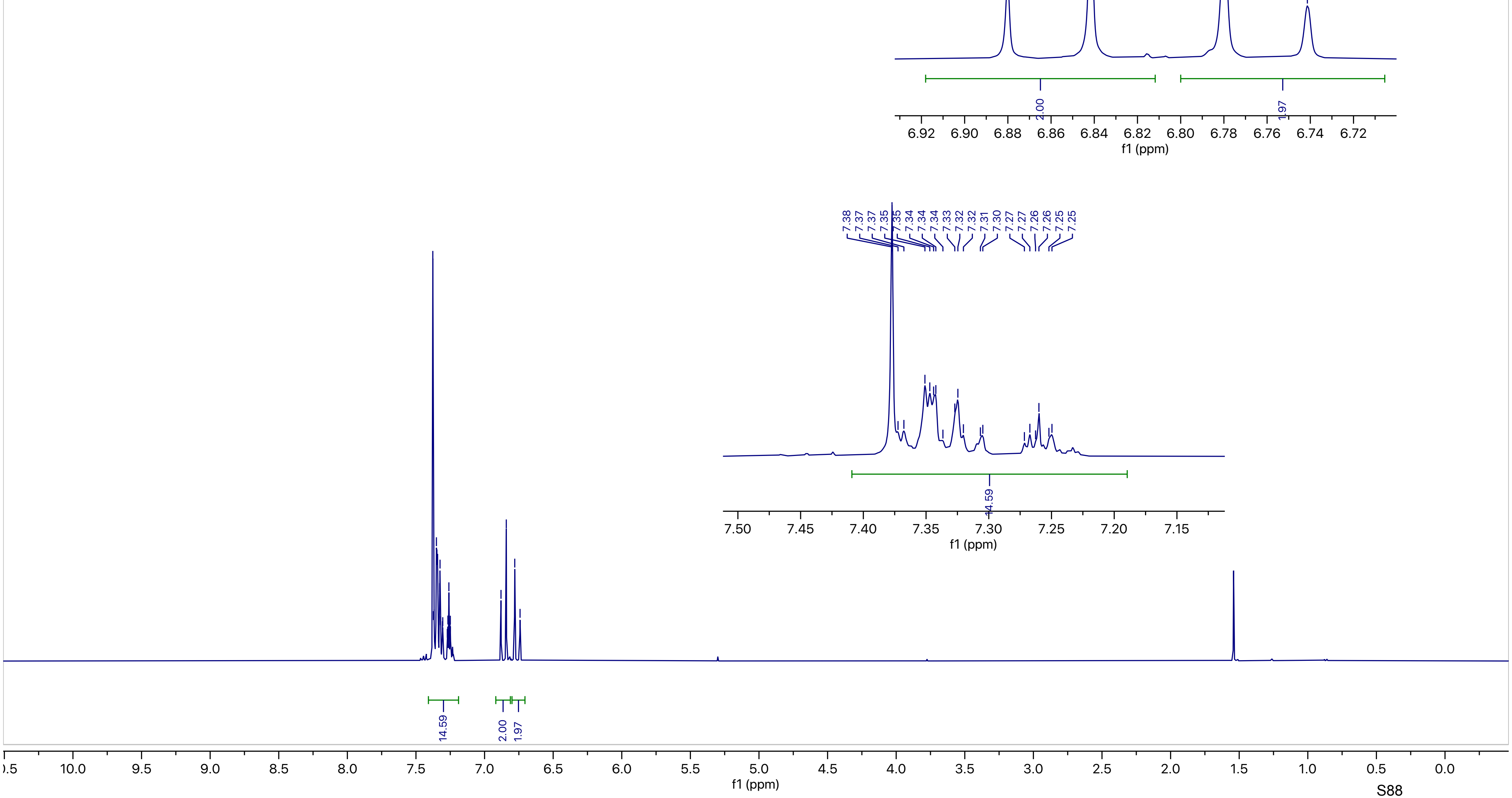

3v

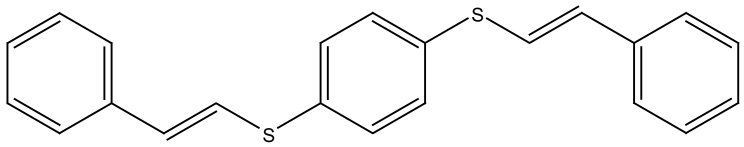

(¹³C-NMR, 101 MHz, $CDCl_3$)

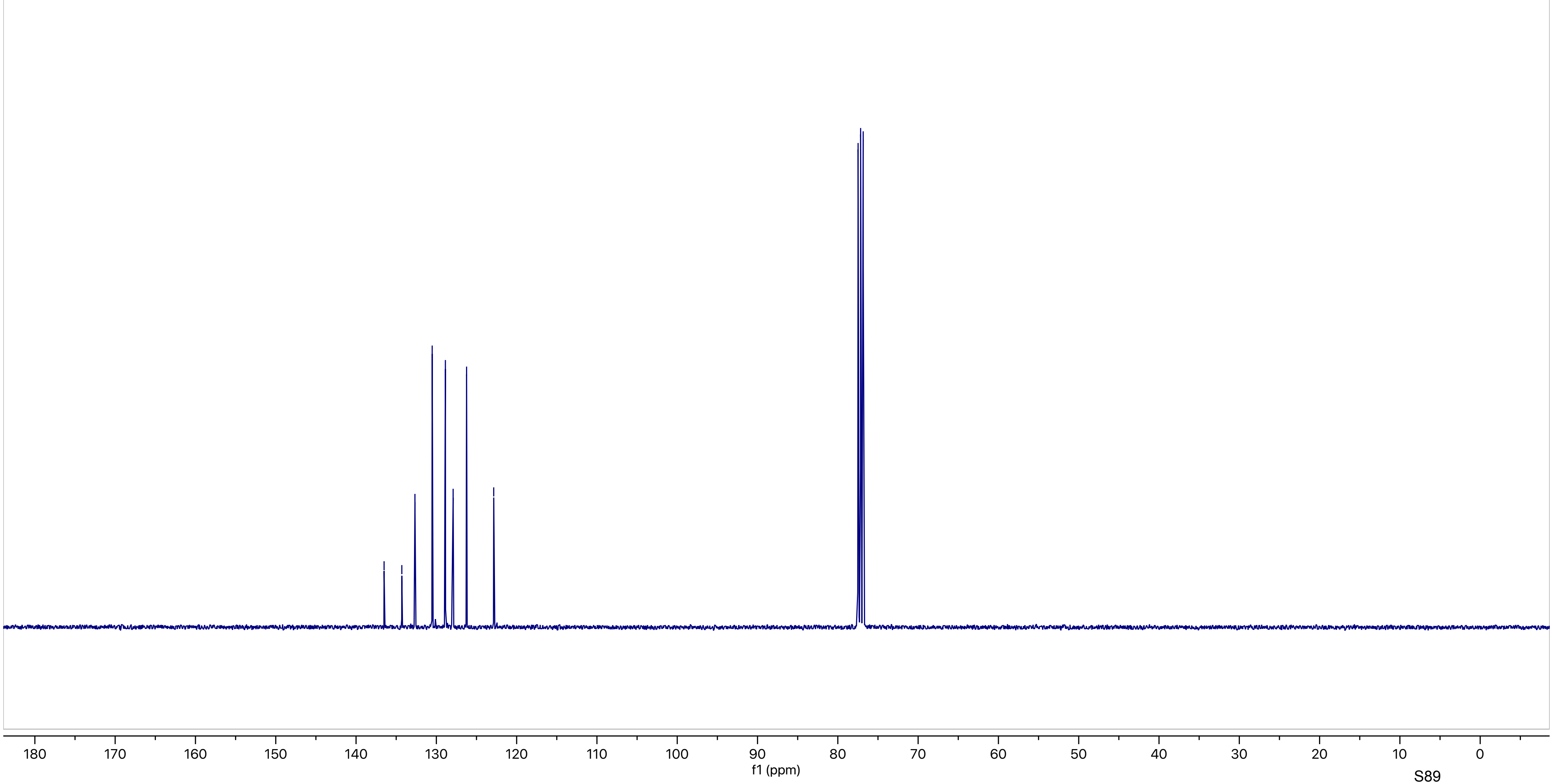

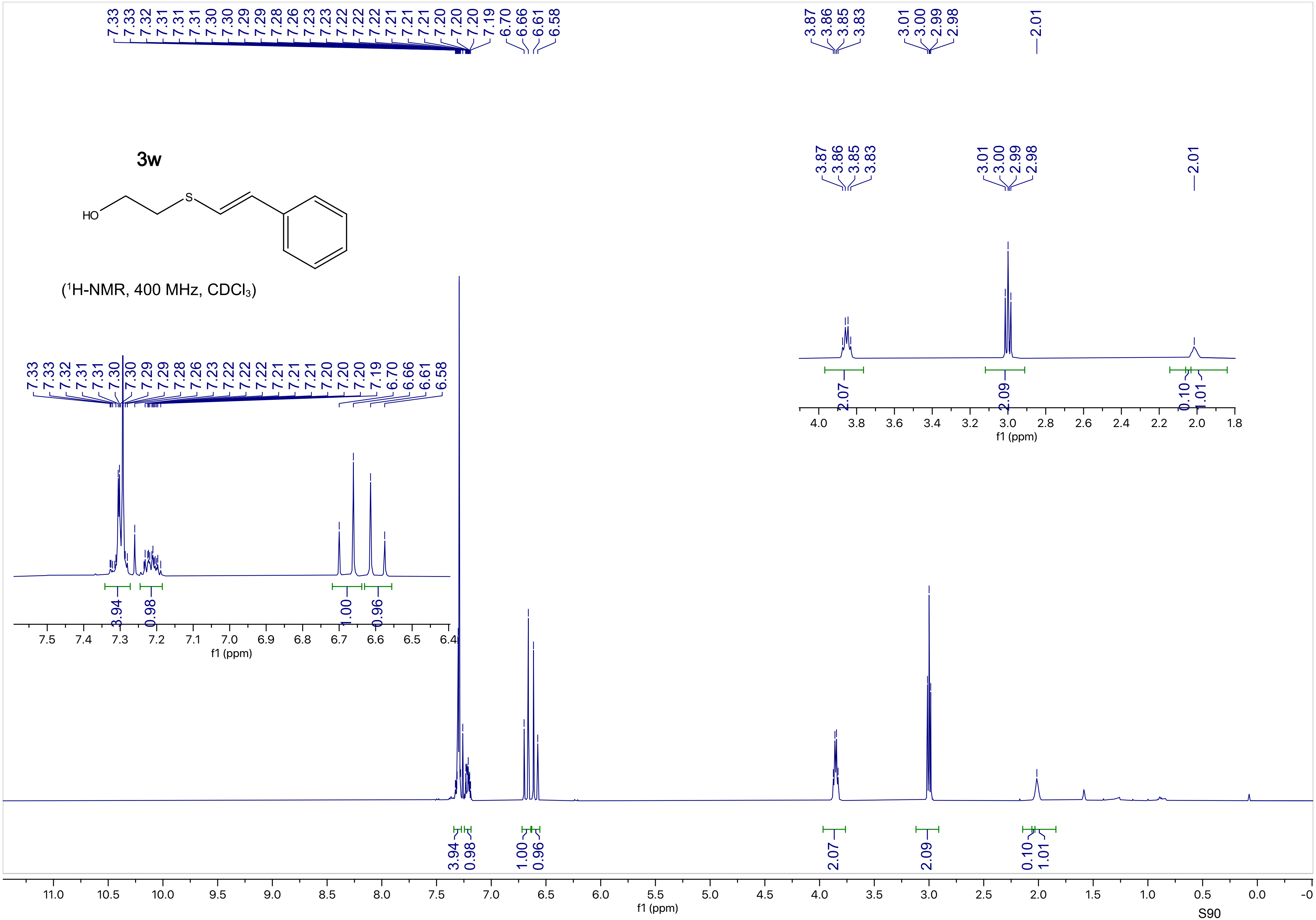
3w
HO
S
(1H-NMR, 400 MHz, CDCl3)
7.33 7.33 7.32 7.31 7.31 7.31 7.30 7.30 7.29 7.29 7.28 7.26 7.23 7.23 7.22 7.22 7.22 7.21 7.21 7.21 7.20 7.20 7.20 7.19 6.70 6.66 6.61 6.58
3.87 3.86 3.85 3.83 3.01 3.00 2.99 2.98 2.01
3.94 0.98 1.00 0.96 2.07 2.09 0.10 1.01
f1 (ppm)

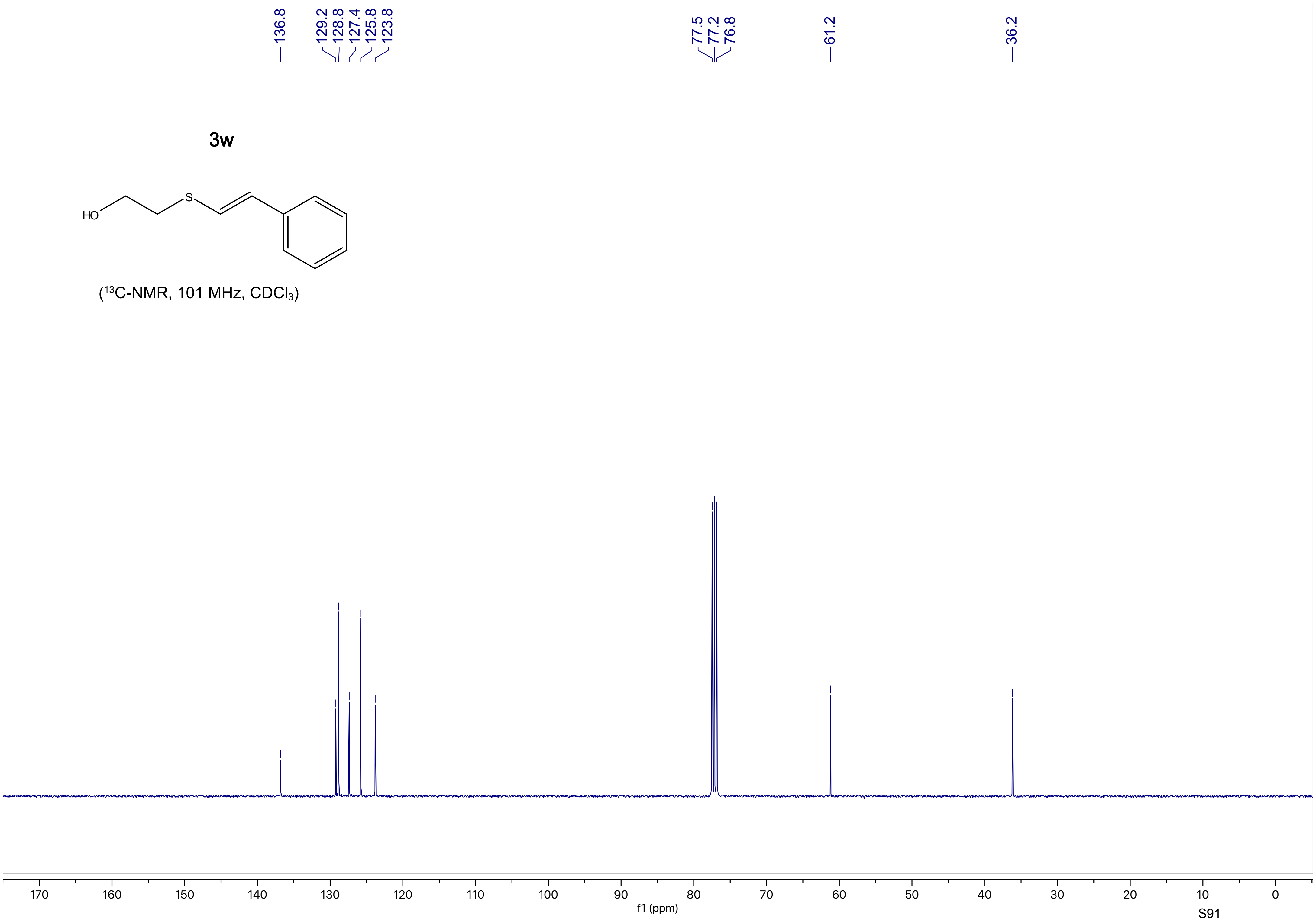

3w

(^{13}C-NMR, 101 MHz, $CDCl_3$)

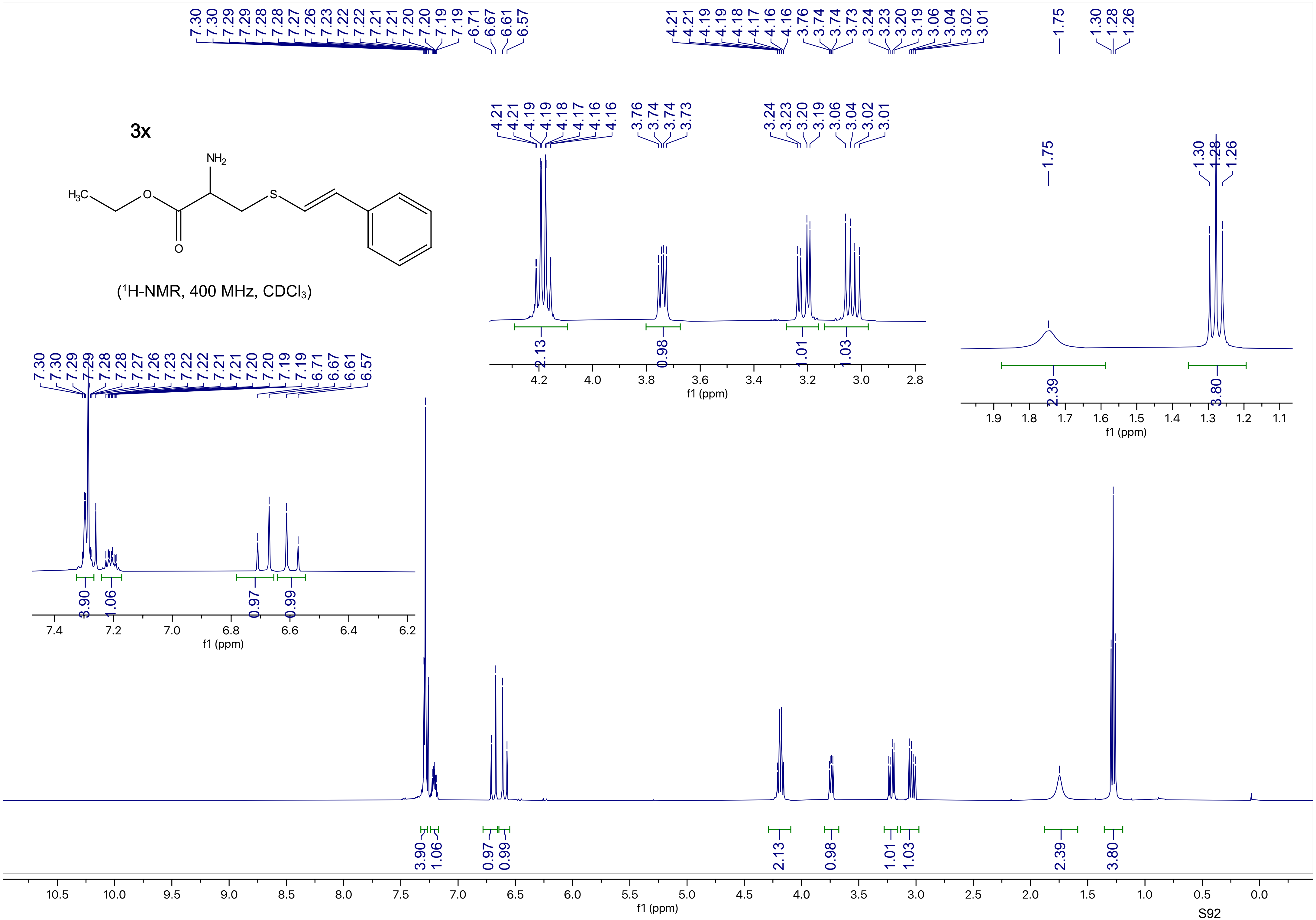

3x
NH2
H3C
O
S
O
(1H-NMR, 400 MHz, CDCl3)
7.30 7.30 7.29 7.29 7.28 7.28 7.27 7.26 7.23 7.22 7.22 7.21 7.21 7.20 7.20 7.19 7.19 6.71 6.67 6.61 6.57
4.21 4.21 4.19 4.19 4.18 4.17 4.16 4.16 3.76 3.74 3.74 3.73 3.24 3.23 3.20 3.19 3.06 3.04 3.02 3.01
1.75
1.30 1.28 1.26
3.90 1.06 0.97 0.99 2.13 0.98 1.01 1.03 2.39 3.80
f1 (ppm)

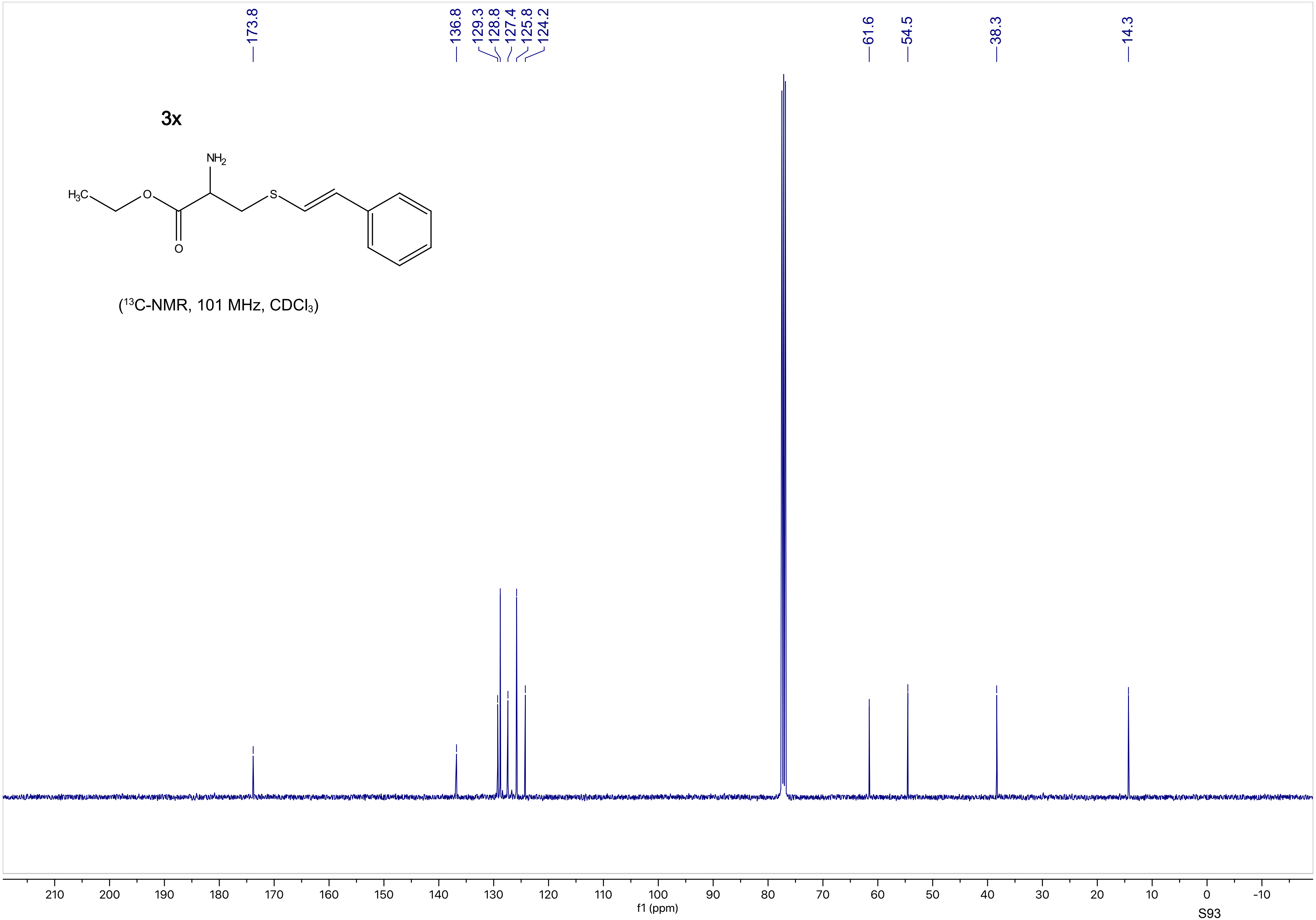
3x
NH2
H3C
O
S
O
(13C-NMR, 101 MHz, CDCl3)
173.8
136.8
129.3
128.8
127.4
125.8
124.2
61.6
54.5
38.3
14.3
210 200 190 180 170 160 150 140 130 120 110 100 90 80 70 60 50 40 30 20 10 0 -10
f1 (ppm)

SFC analysis **3x**

3x

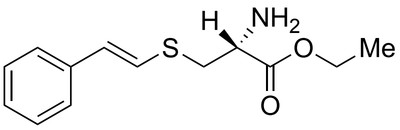

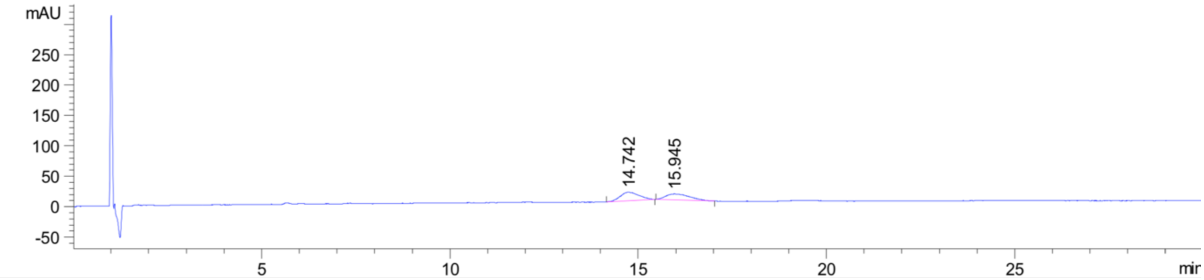

```
Signal 2: DAD1 B, Sig=230,4 Ref=off

Peak RetTime Type  Width     Area      Height     Area
  #   [min]        [min]   [mAU*s]     [mAU]        %
----|-------|----|-------|----------|----------|--------|
   1  14.742 BV R  0.4087  482.38065   13.97654  52.8368
   2  15.945 BV R  0.5200  430.58301    9.77252  47.1632

Totals :                   912.96365   23.74906
```

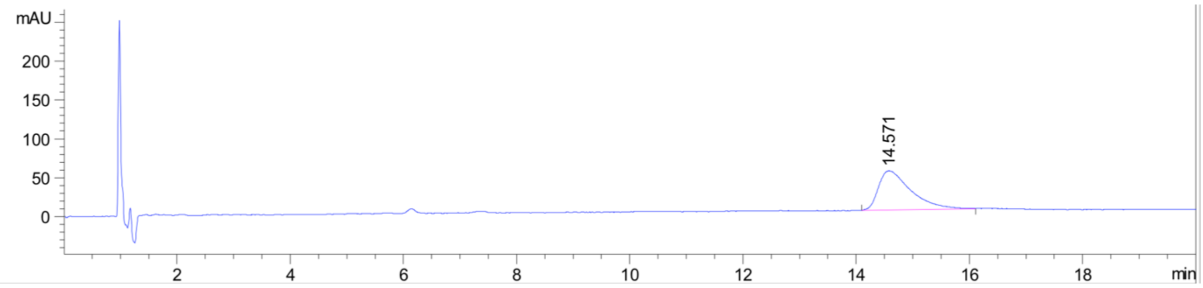

```
Signal 2: DAD1 B, Sig=230,4 Ref=off

Peak RetTime Type  Width     Area      Height     Area
  #   [min]        [min]   [mAU*s]     [mAU]        %
----|-------|----|-------|----------|----------|--------|
   1  14.571 BV R  0.5146 2012.49976   50.53609 100.0000

Totals :                  2012.49976   50.53609
```

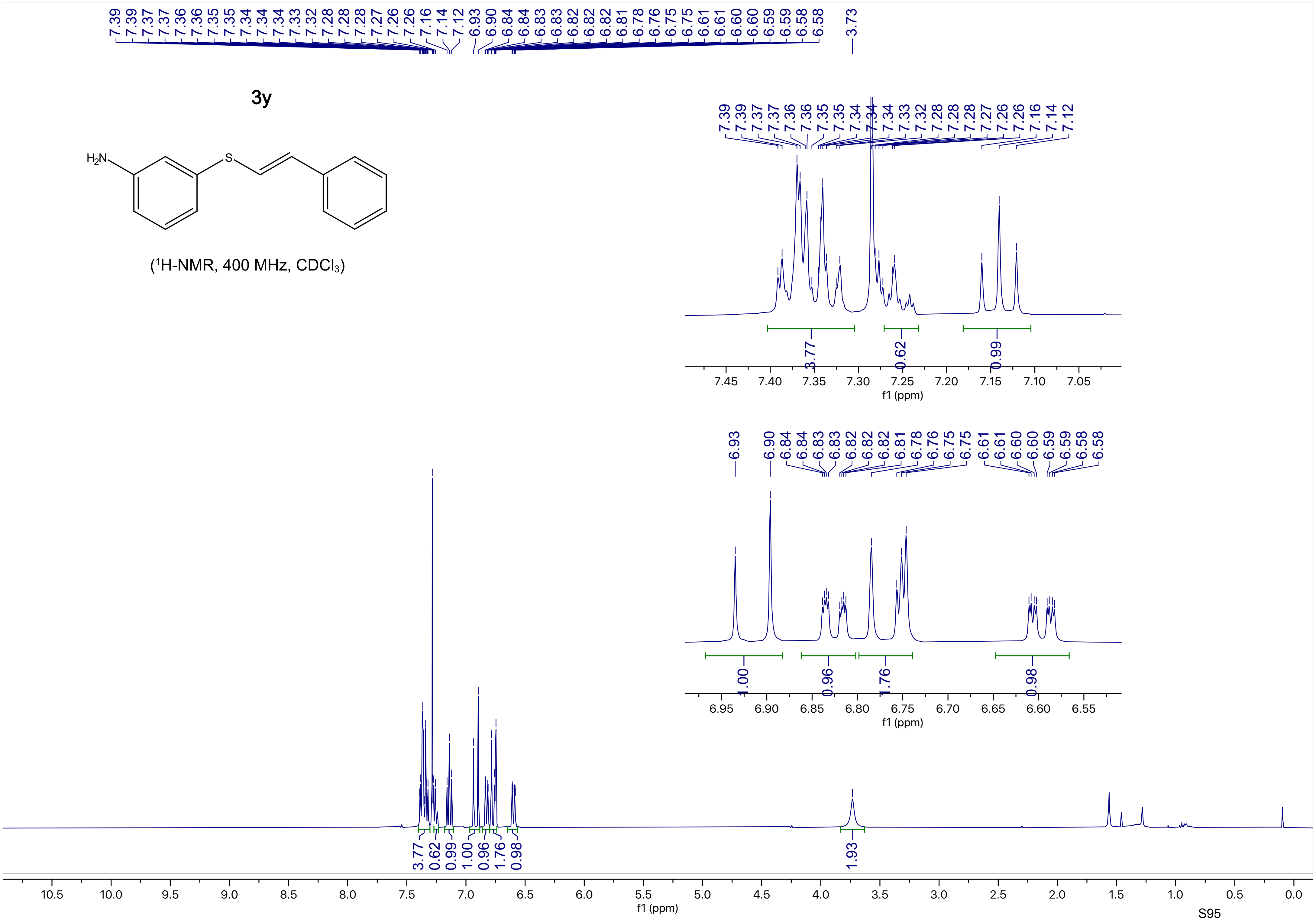

(¹H-NMR, 400 MHz, $CDCl_3$)

3y

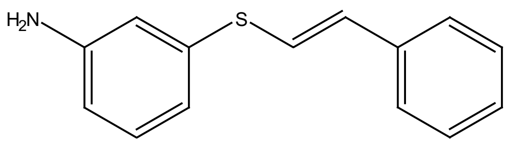

(13C-NMR, 101 MHz, $CDCl_3$)

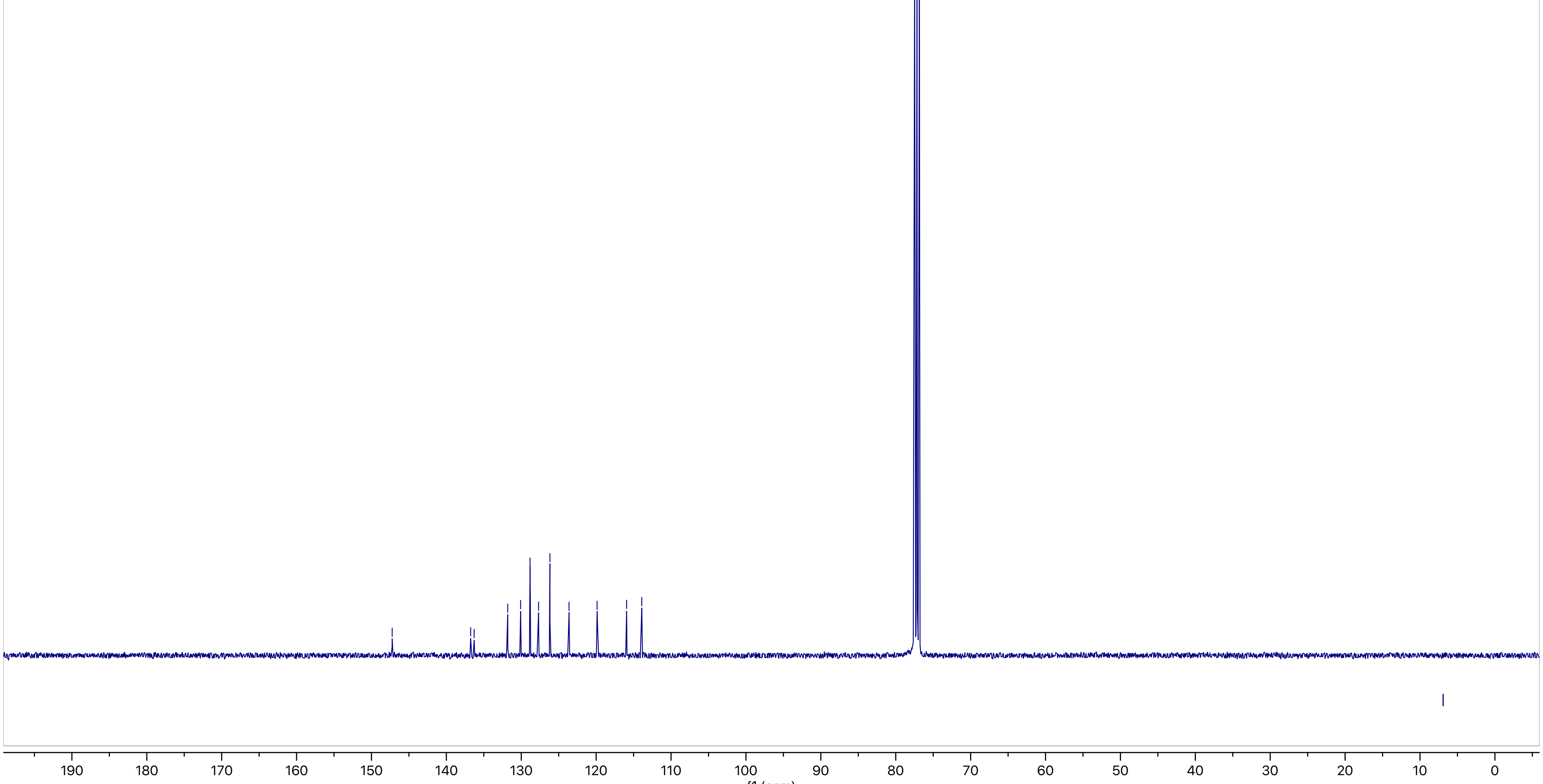

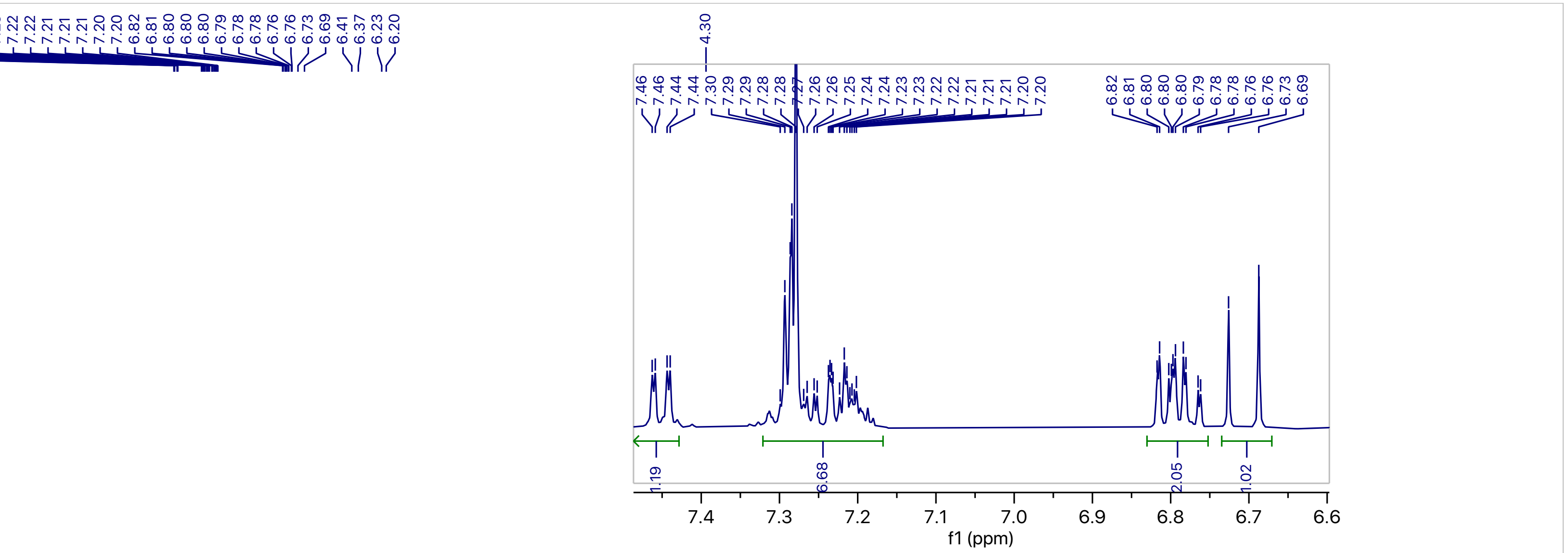

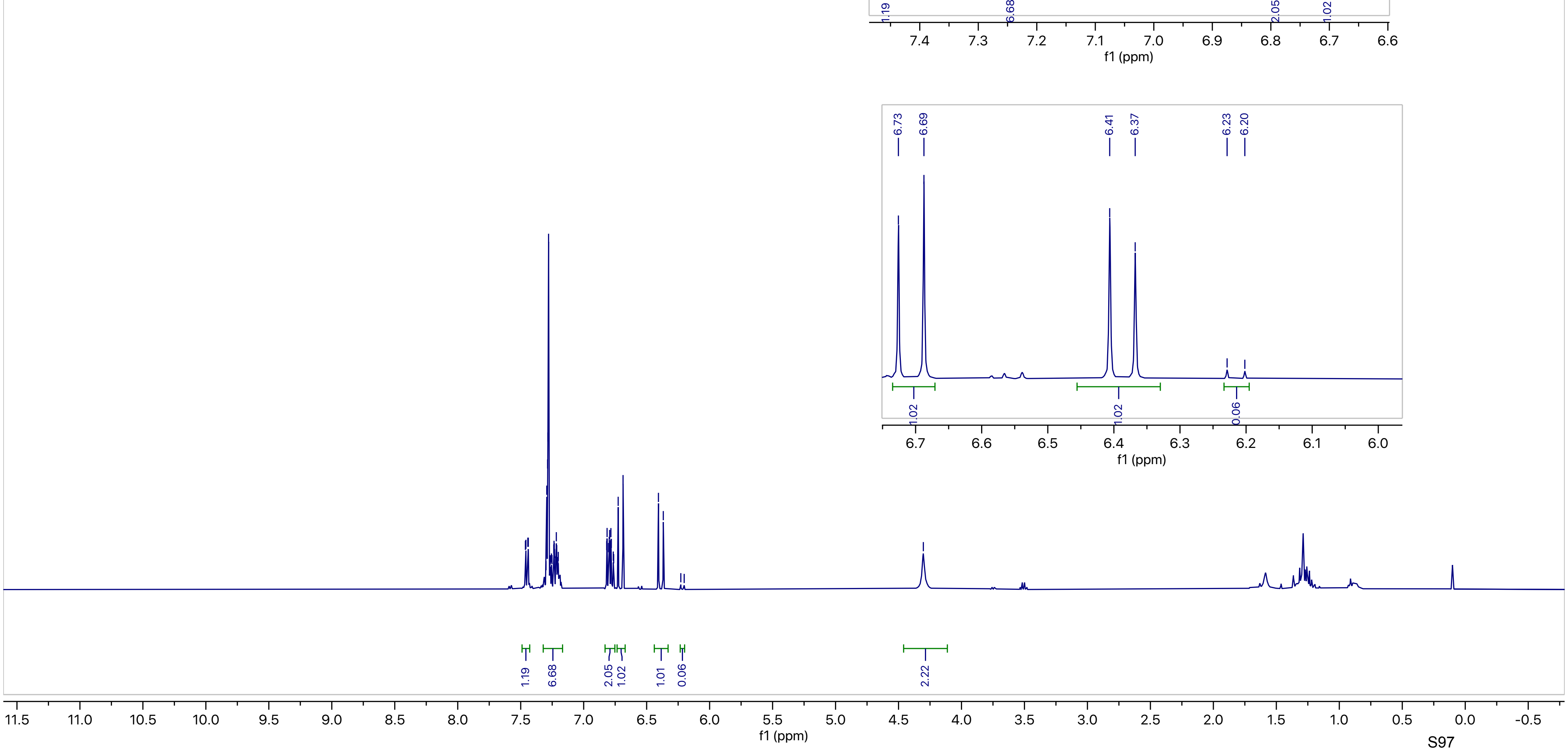

3z

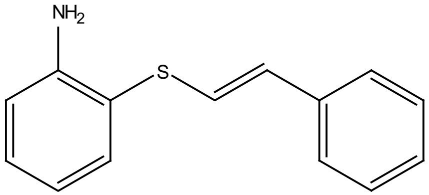

(^{1}H-NMR, 400 MHz, $CDCl_3$)

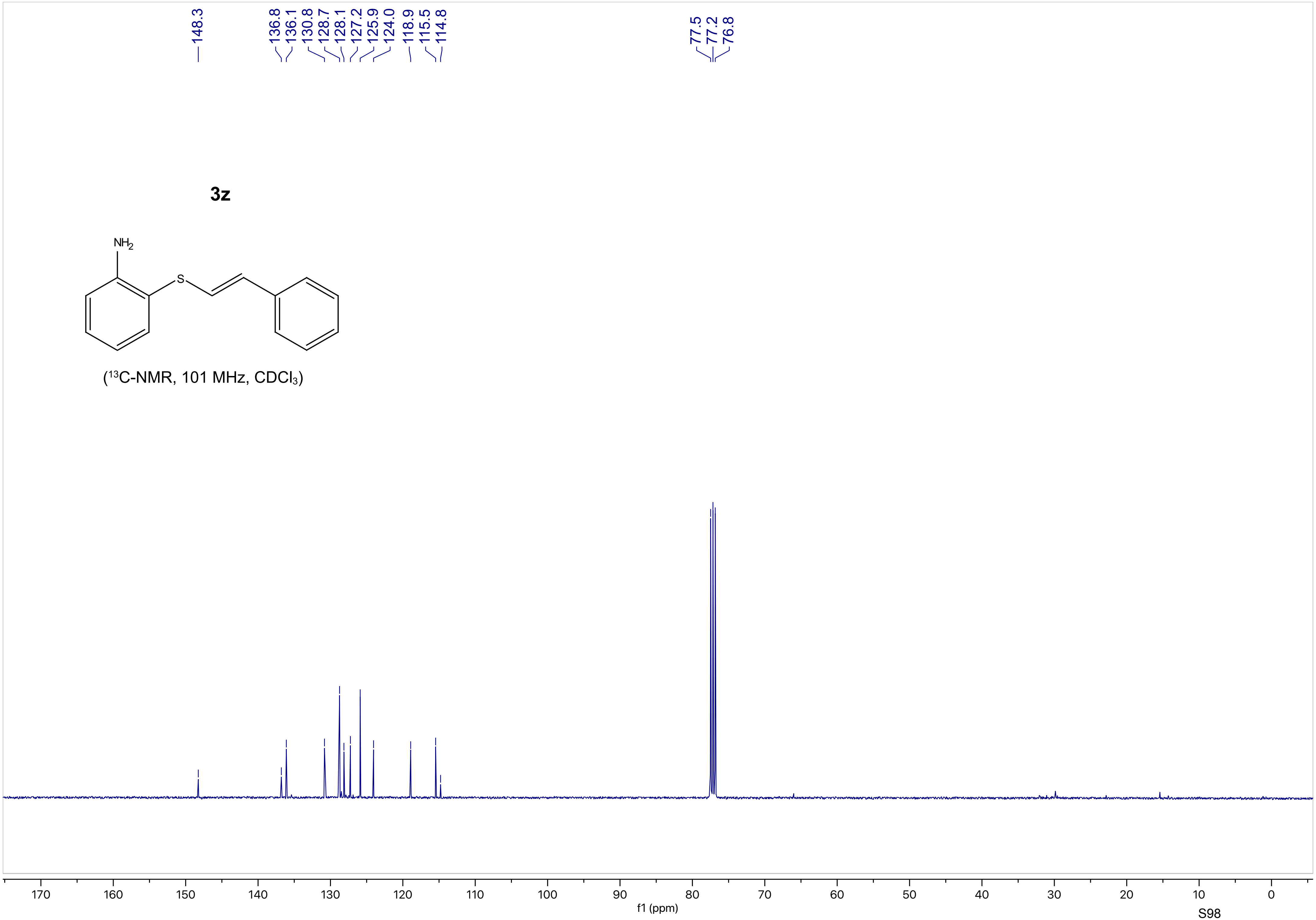
148.3
136.8
136.1
130.8
128.7
128.1
127.2
125.9
124.0
118.9
115.5
114.8
77.5
77.2
76.8
3z
NH2
S
(13C-NMR, 101 MHz, CDCl3)
170 160 150 140 130 120 110 100 90 80 70 60 50 40 30 20 10 0
f1 (ppm)

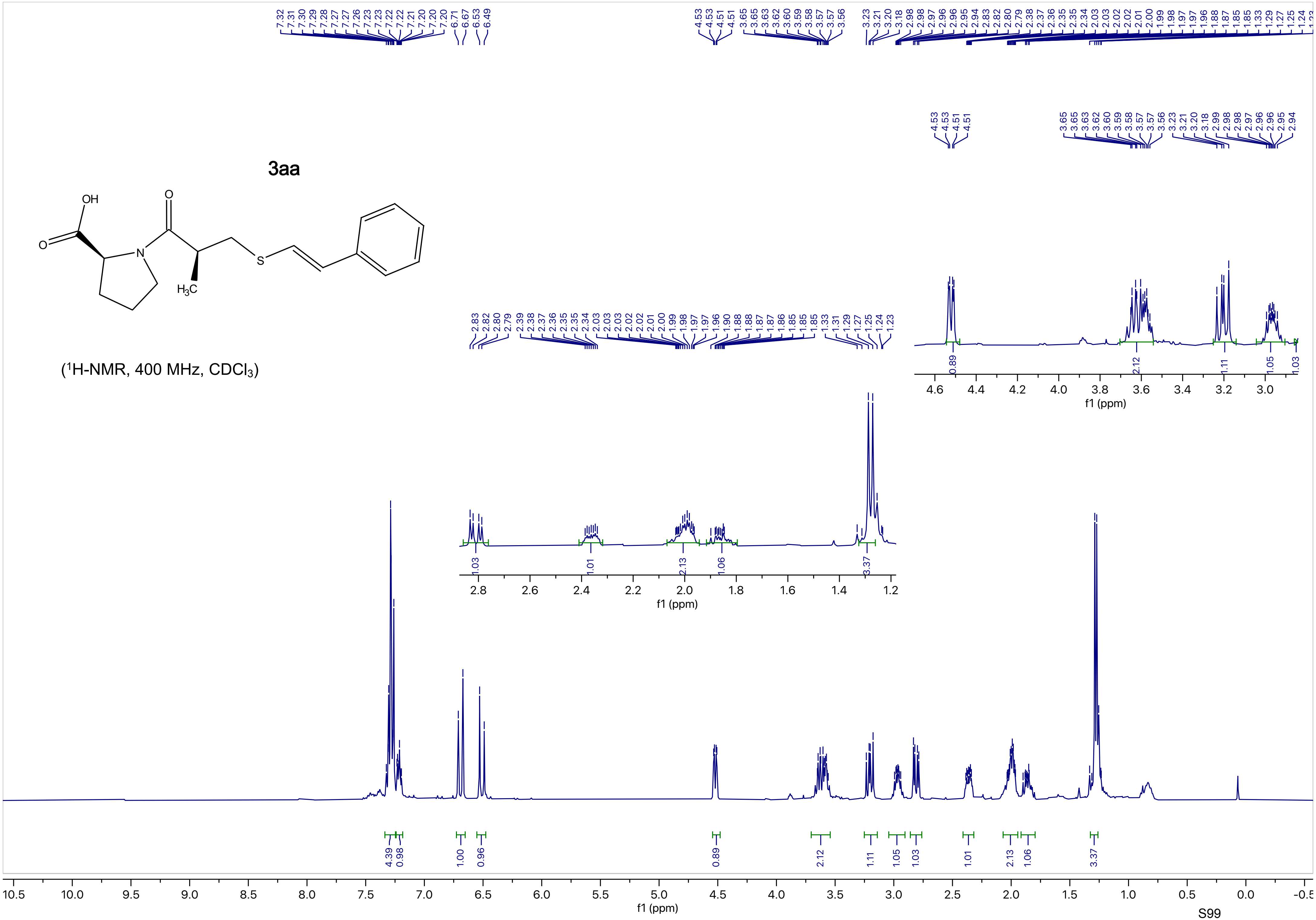
3aa
(¹H-NMR, 400 MHz, CDCl3)
f1 (ppm)

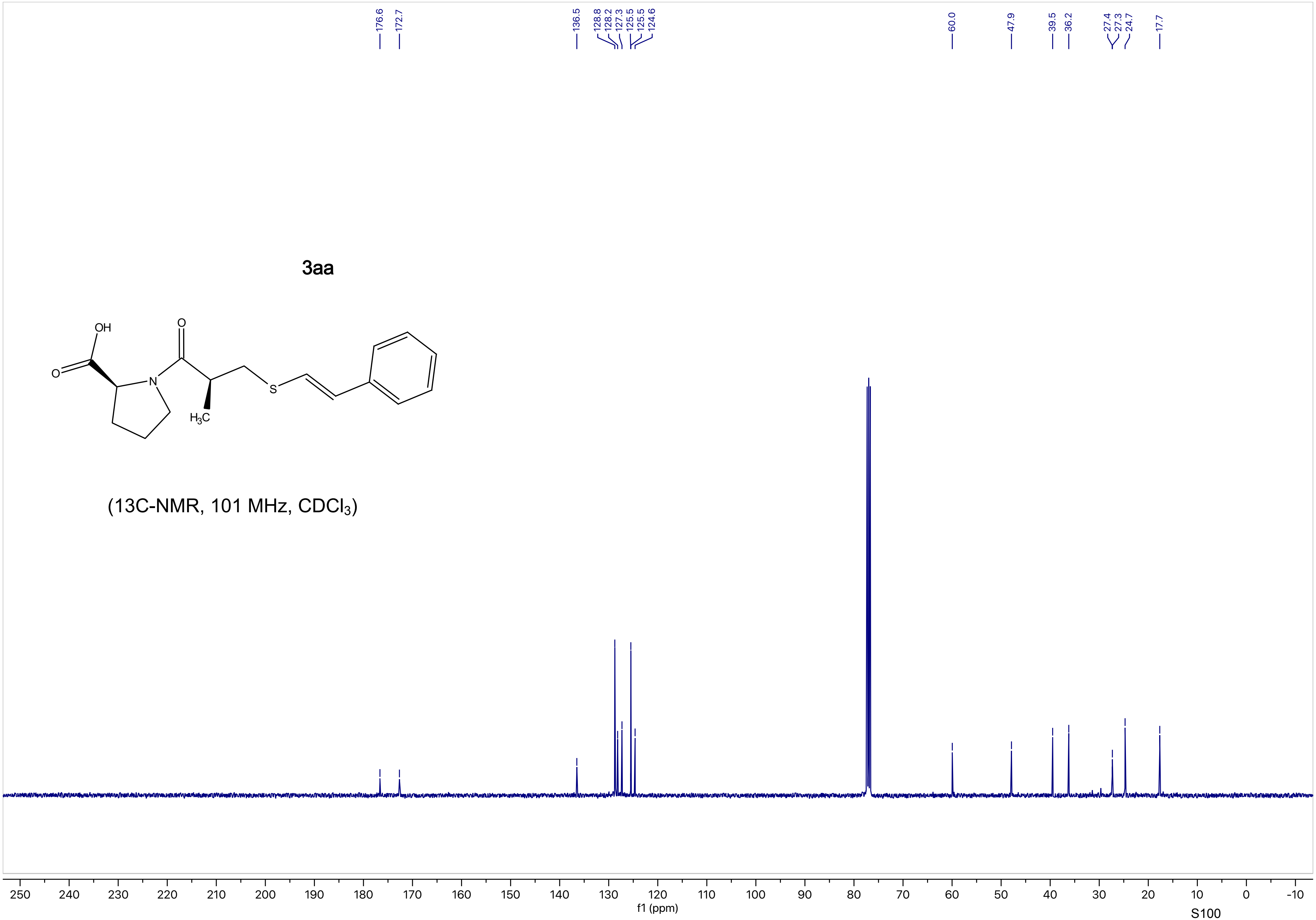
176.6
172.7
136.5
128.8
128.2
127.3
125.5
125.5
124.6
60.0
47.9
39.5
36.2
27.4
27.3
24.7
17.7
3aa
OH
O
O
N
S
H_3C
(13C-NMR, 101 MHz, $CDCl_3$)
250 240 230 220 210 200 190 180 170 160 150 140 130 120 110 100 90 80 70 60 50 40 30 20 10 0 -10
f1 (ppm)

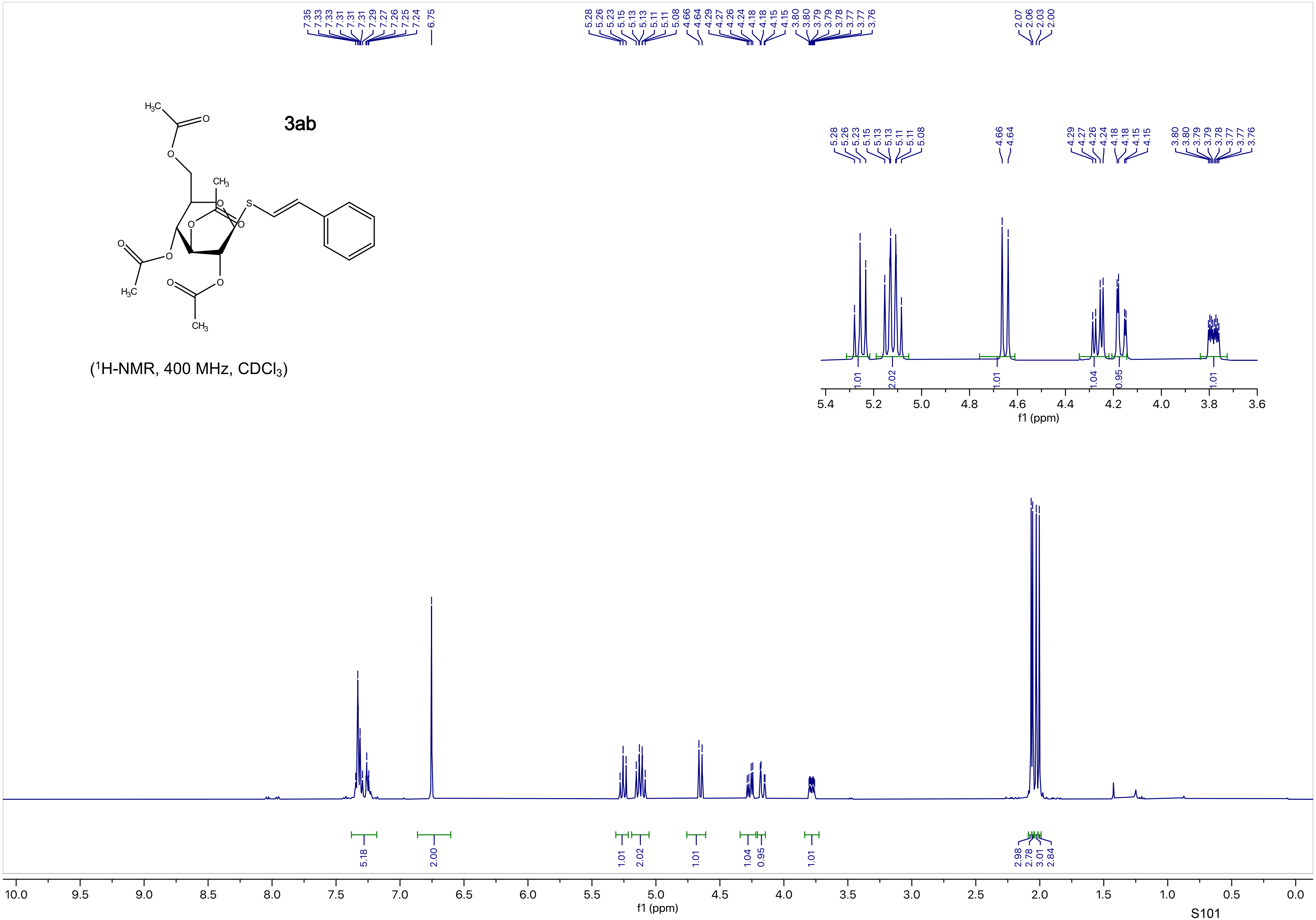
3ab
(1H-NMR, 400 MHz, CDCl3)
7.35 7.33 7.33 7.31 7.31 7.31 7.29 7.27 7.26 7.25 7.24 6.75
5.28 5.26 5.23 5.15 5.13 5.13 5.11 5.11 5.08 4.66 4.64 4.29 4.27 4.26 4.24 4.18 4.18 4.15 4.15 3.80 3.80 3.79 3.79 3.78 3.77 3.77 3.76
2.07 2.06 2.03 2.00
5.18 2.00 1.01 2.02 1.01 1.04 0.95 1.01 2.98 2.78 3.01 2.84
f1 (ppm)

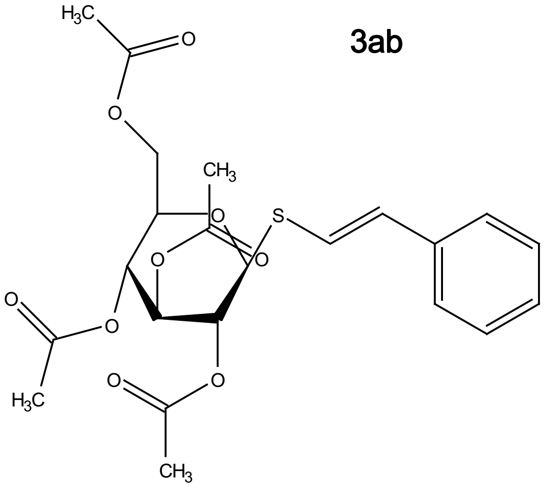

3ab

(^{13}C-NMR, 101 MHz, $CDCl_3$)

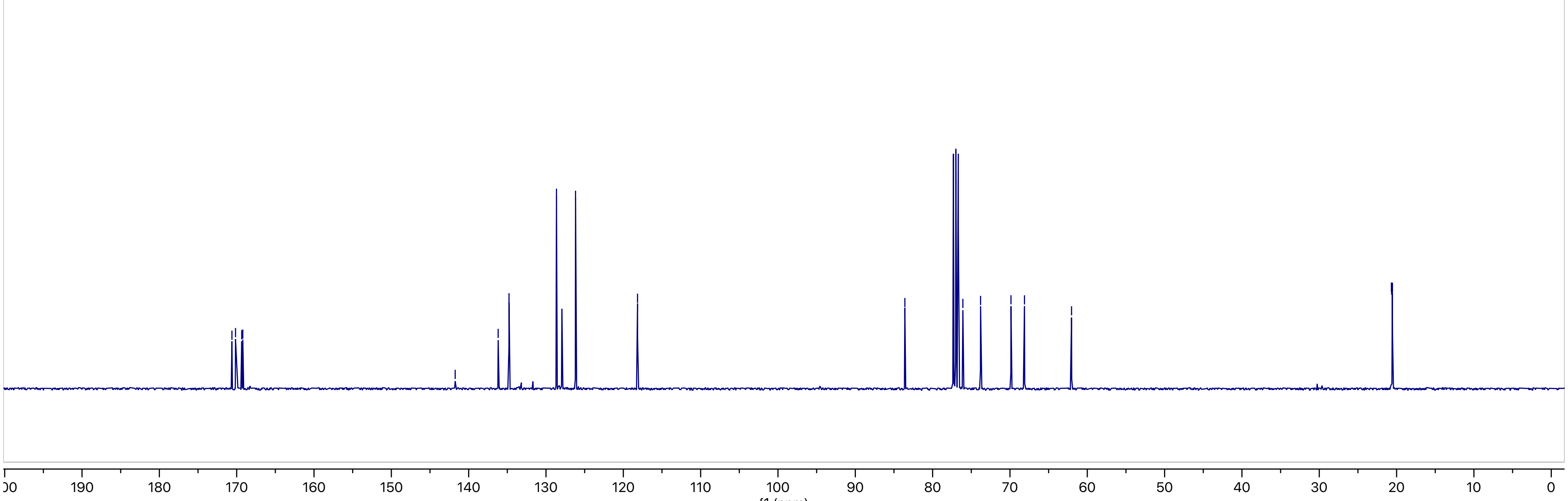

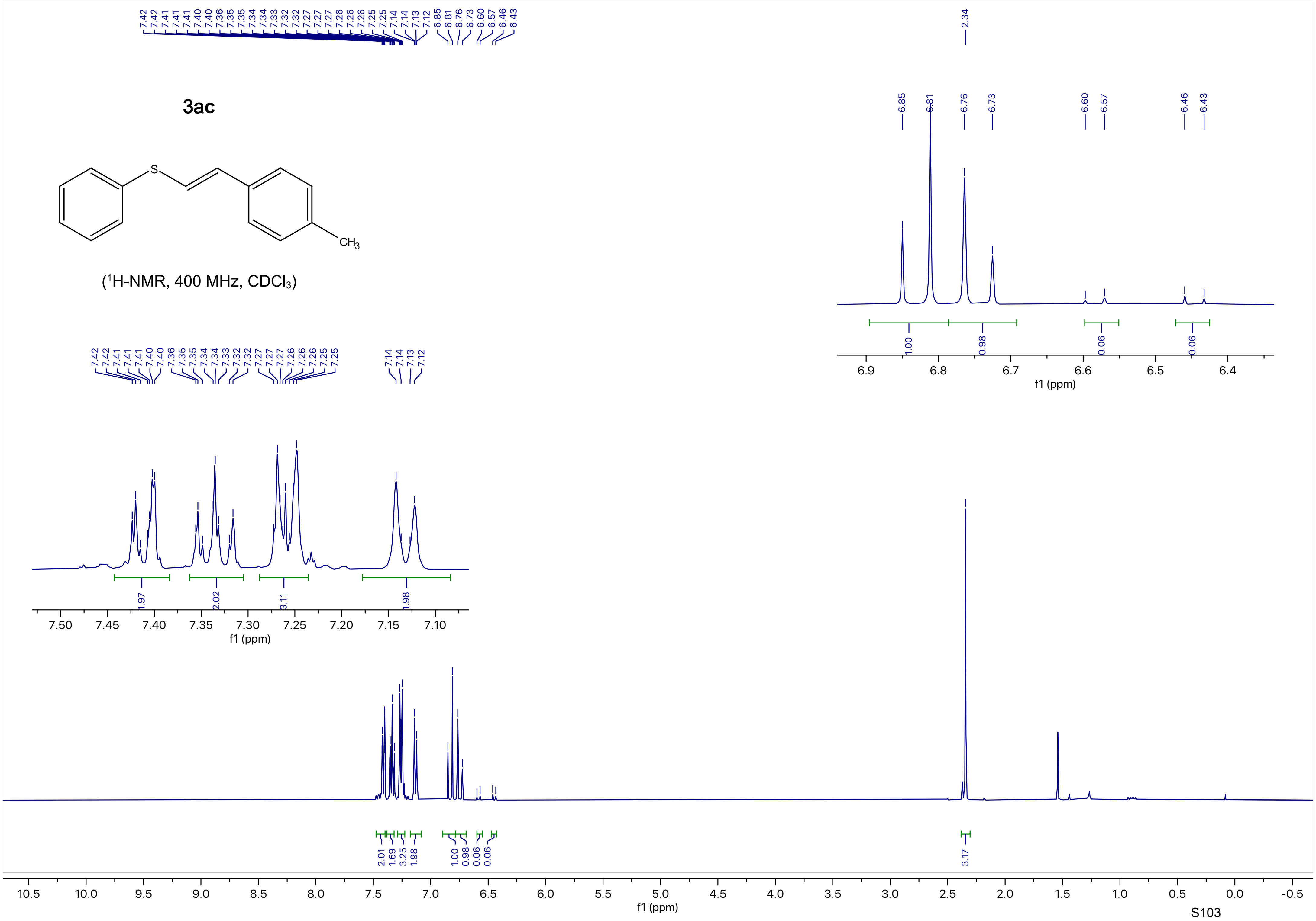
3ac
S
CH3
(1H-NMR, 400 MHz, CDCl3)
7.42 7.42 7.41 7.41 7.41 7.40 7.40 7.36 7.35 7.35 7.34 7.34 7.33 7.32 7.32 7.27 7.27 7.27 7.26 7.26 7.26 7.25 7.25 7.14 7.14 7.13 7.12 6.85 6.81 6.76 6.73 6.60 6.57 6.46 6.43
2.34
1.97 2.02 3.11 1.98
1.00 0.98 0.06 0.06
2.01 1.69 3.25 1.98 1.00 0.98 0.06 0.06 3.17
f1 (ppm)

3ac

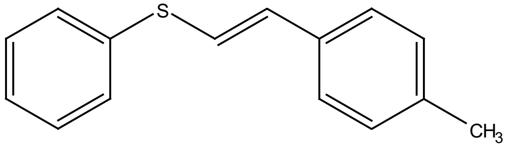

(^{13}C-NMR, 101 MHz, $CDCl_3$)

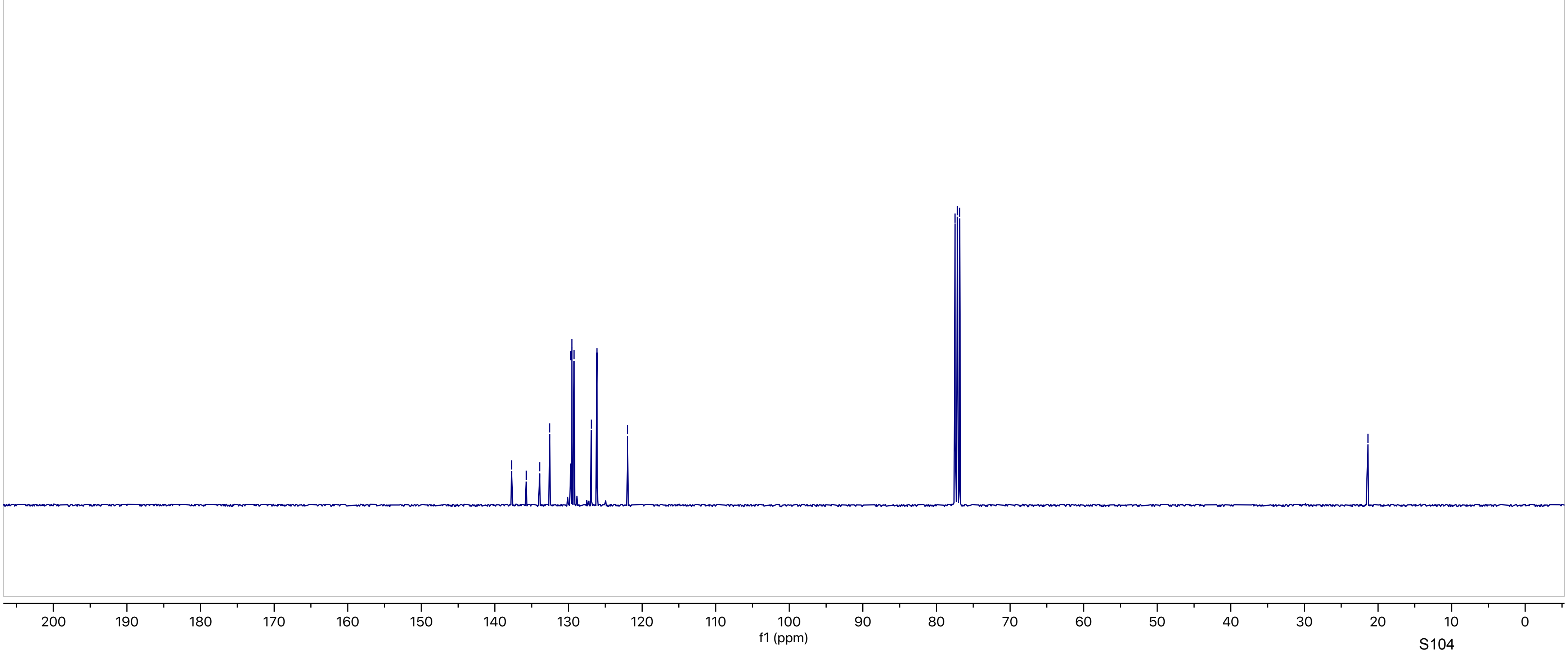

3ad

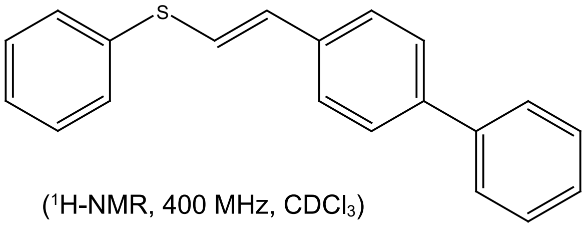

(^{1}H-NMR, 400 MHz, $CDCl_3$)

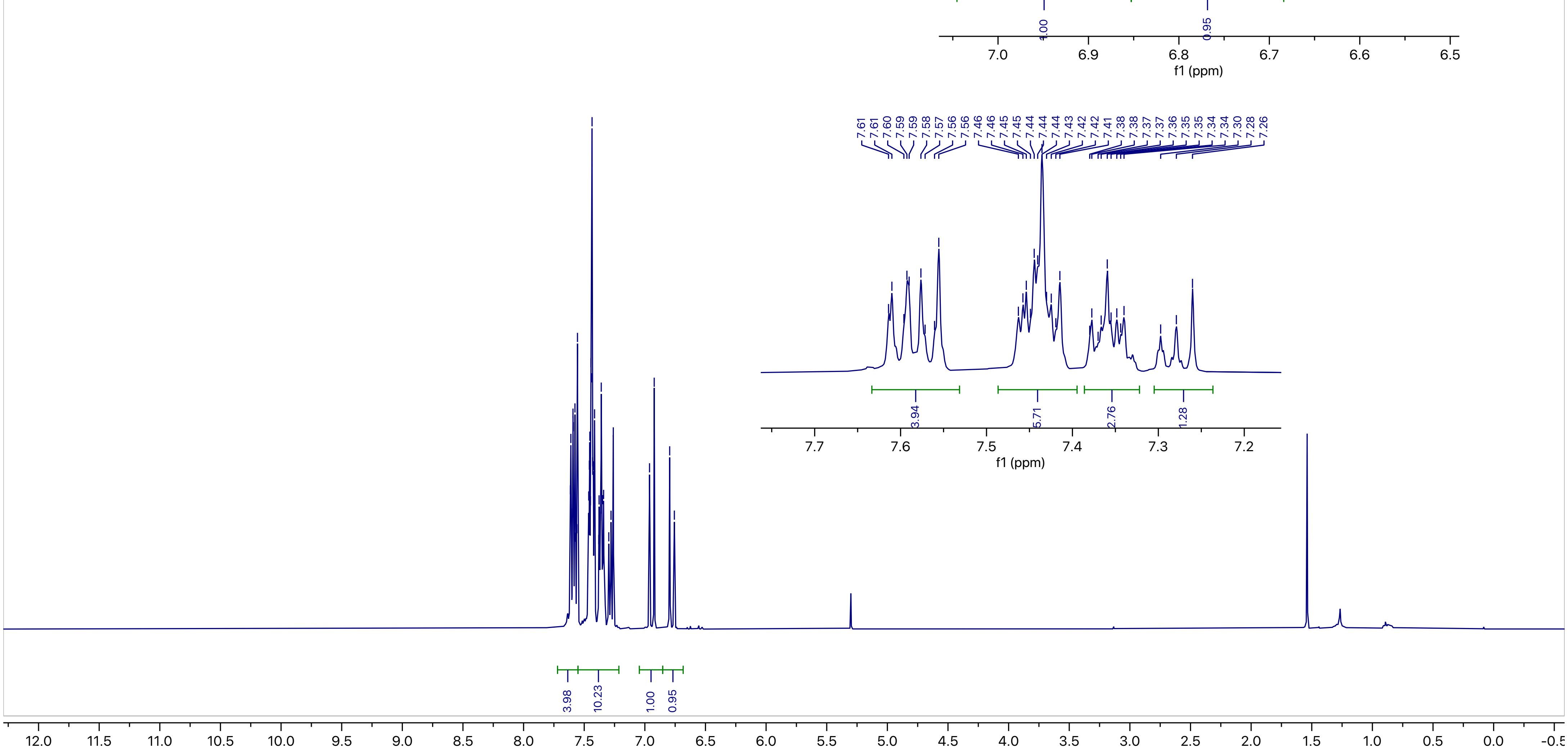

3ad

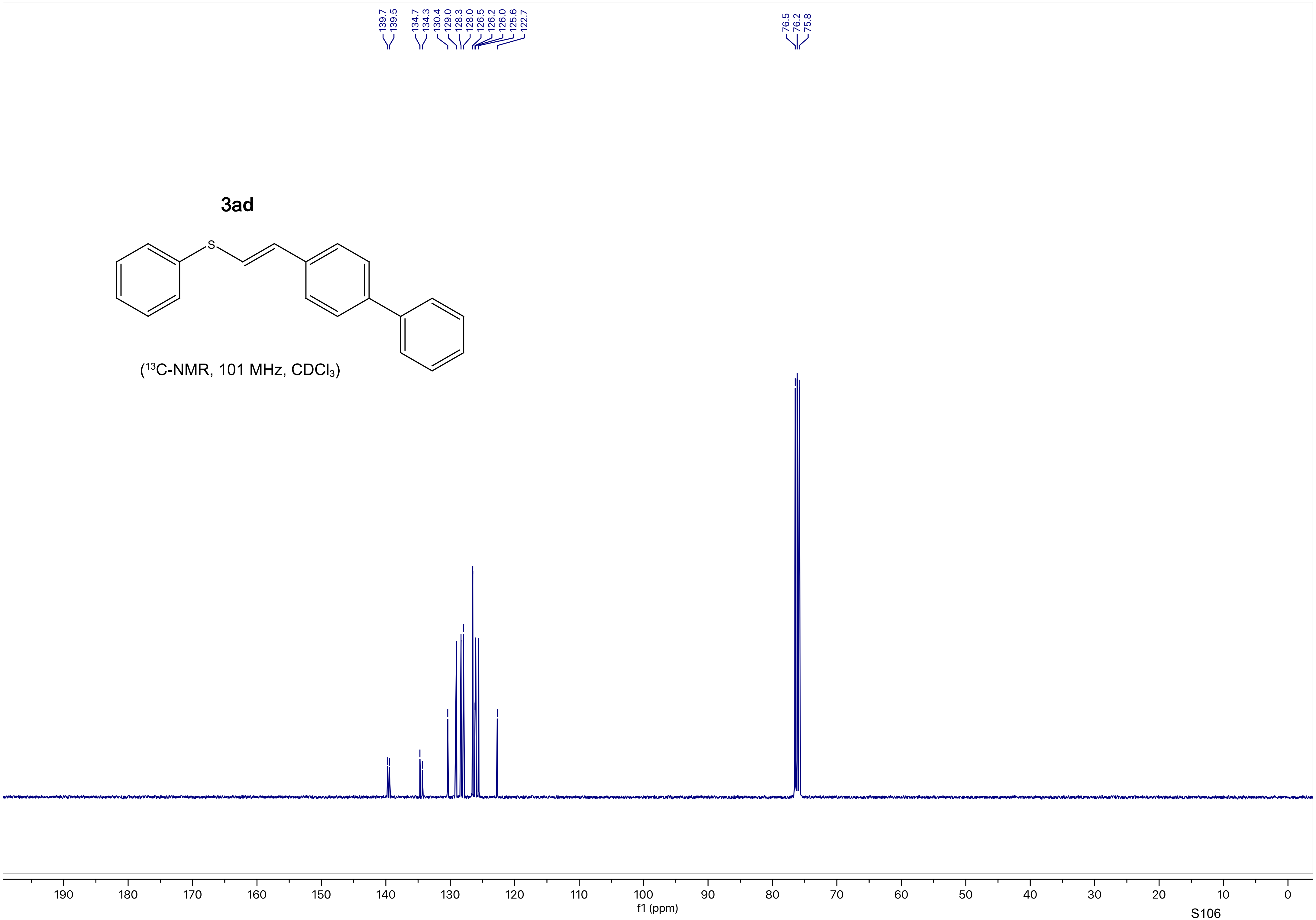

(^{13}C-NMR, 101 MHz, CDCl$_3$)

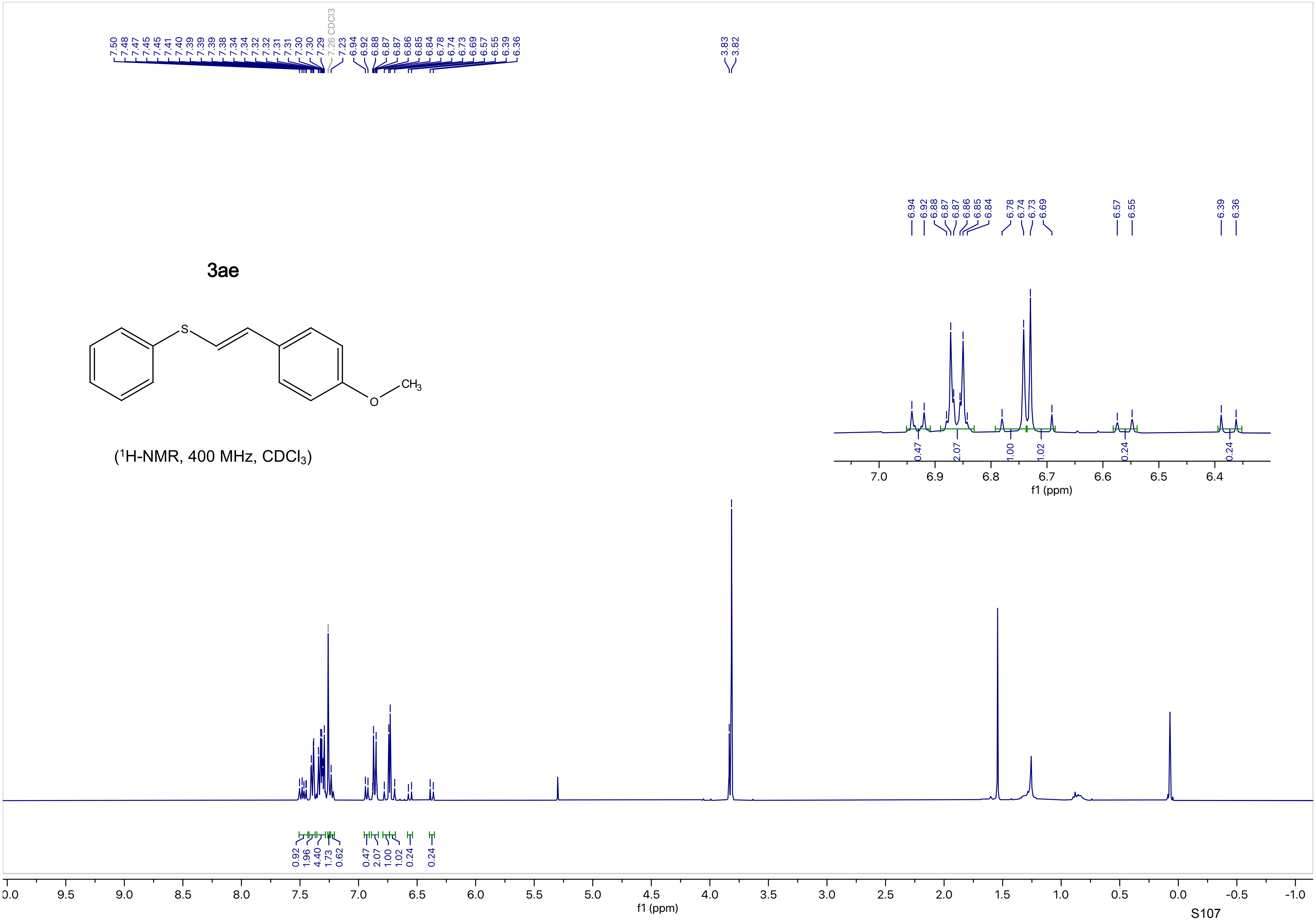
3ae
(¹H-NMR, 400 MHz, CDCl₃)
S
O
CH3
CDCl3
3.83
3.82
f1 (ppm)

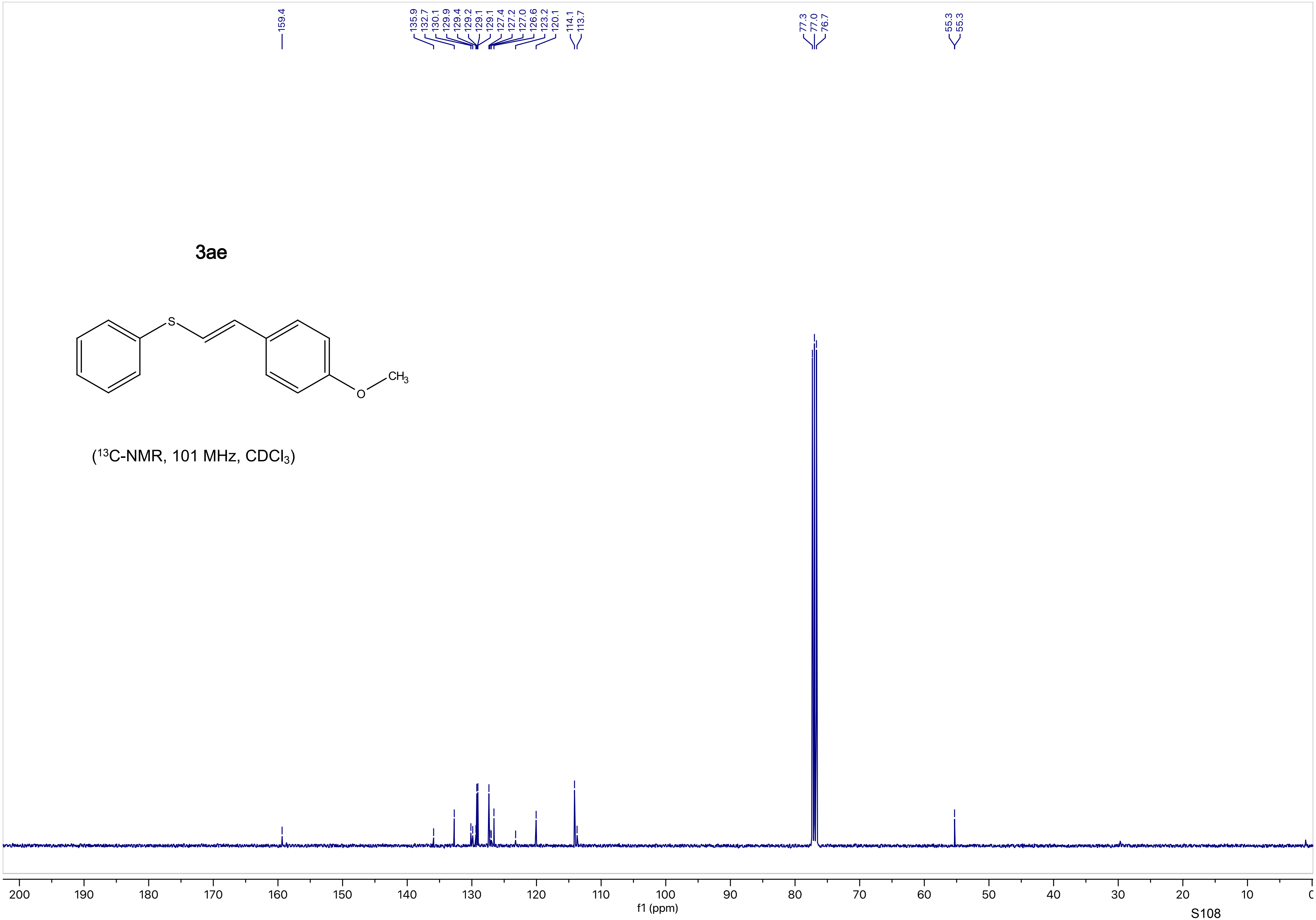
159.4
135.9
132.7
130.1
129.9
129.4
129.2
129.1
129.1
127.4
127.2
127.0
126.6
123.2
120.1
114.1
113.7
77.3
77.0
76.7
55.3
55.3
3ae
S
O
CH3
(13C-NMR, 101 MHz, CDCl3)
200
190
180
170
160
150
140
130
120
110
100
90
80
70
60
50
40
30
20
10
0
f1 (ppm)

3af

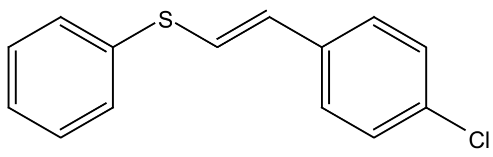

(¹H-NMR, 400 MHz, $CDCl_3$)

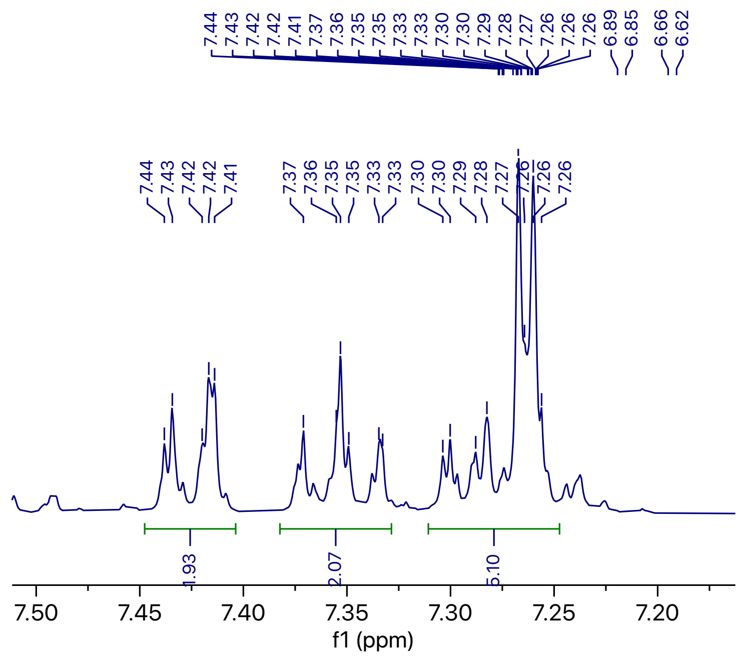

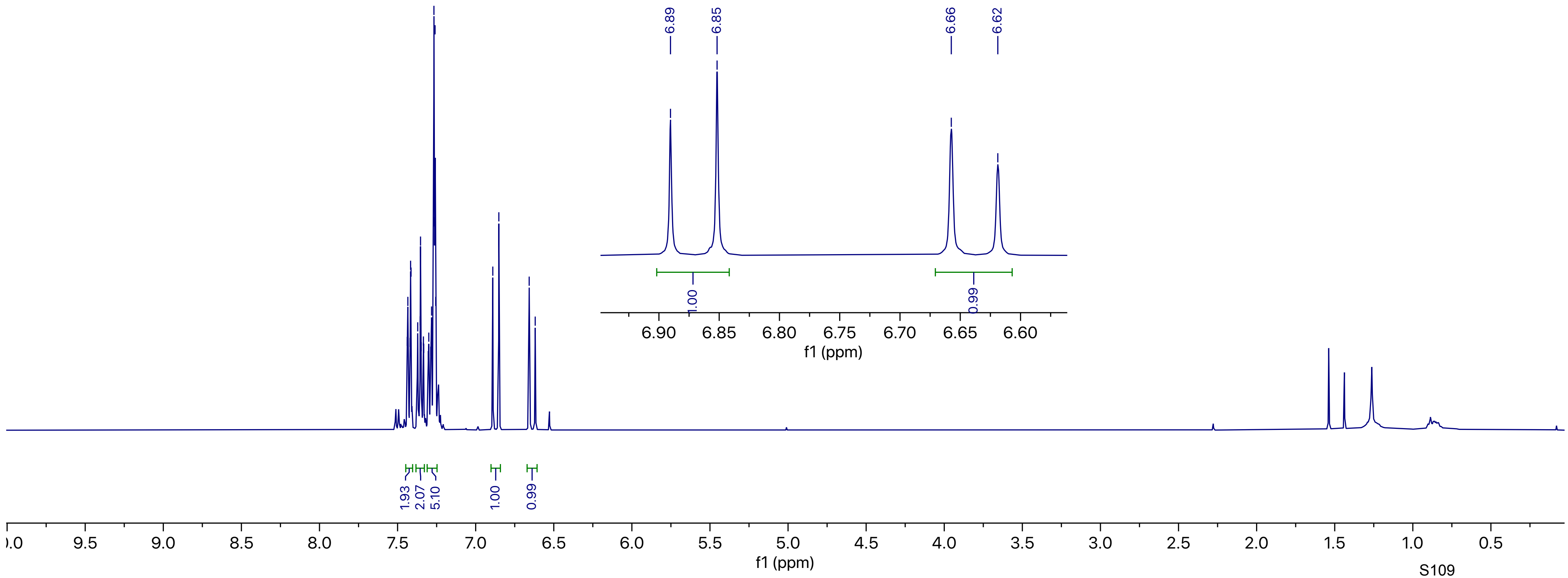

3af

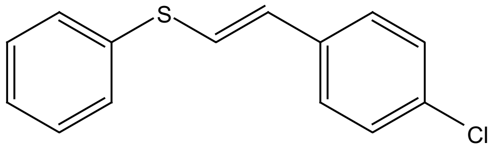

(¹³C-NMR, 101 MHz, CDCl$_3$)

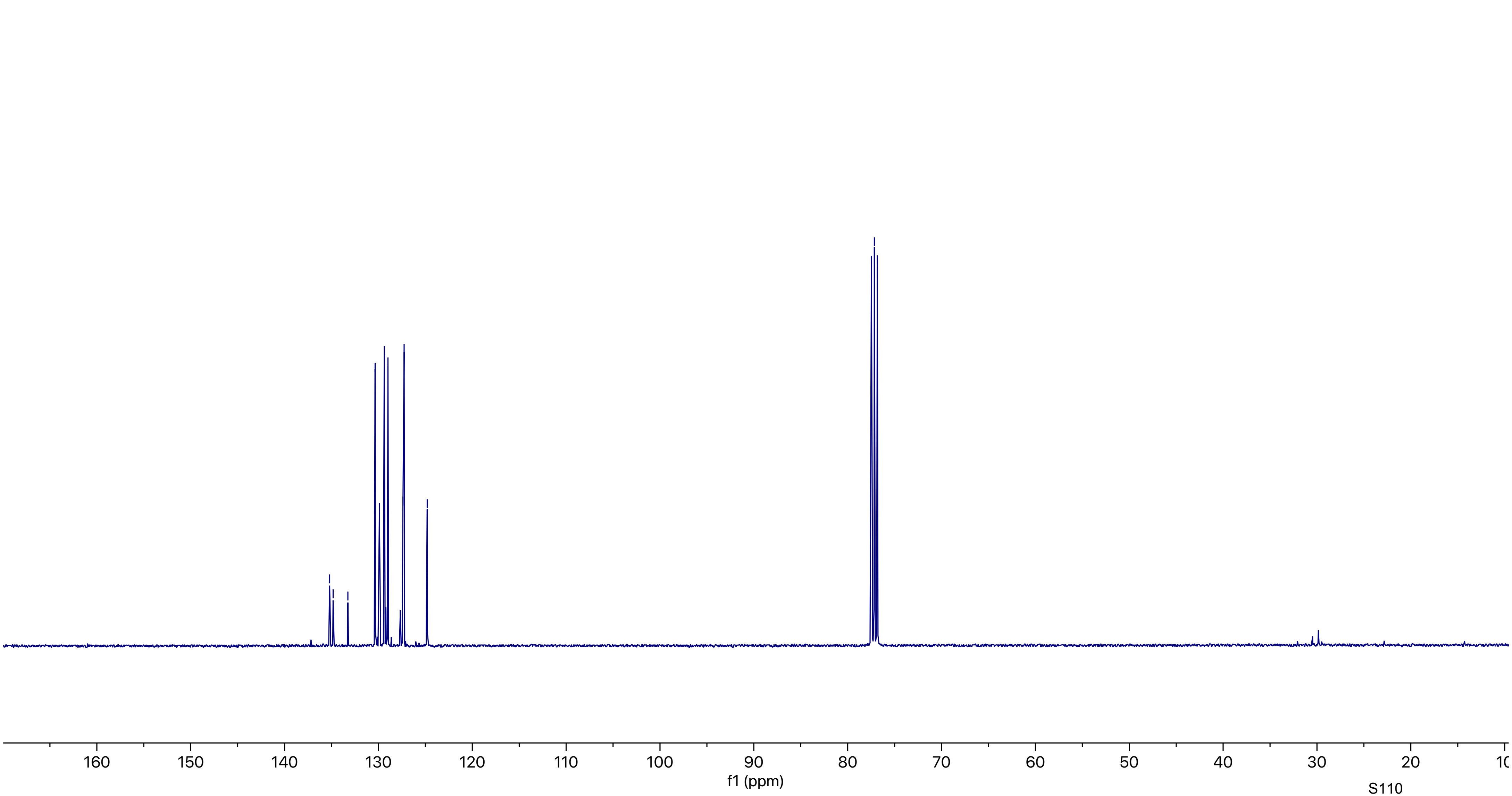

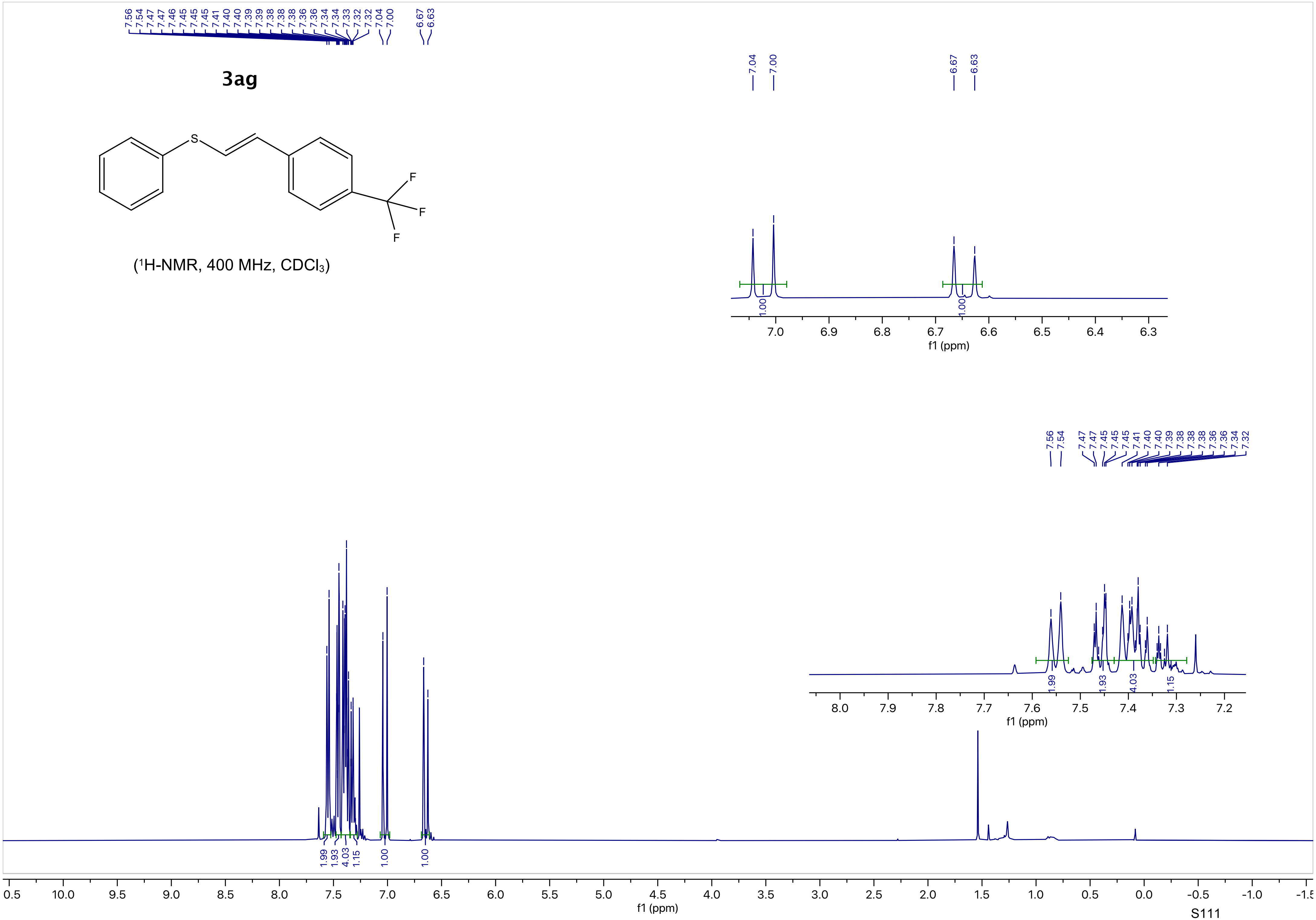

3ag

(^{1}H-NMR, 400 MHz, $CDCl_3$)

3ag

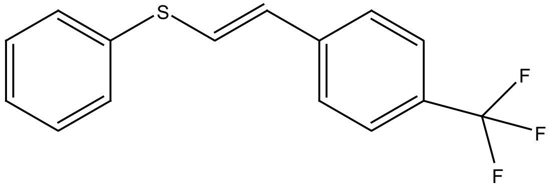

(^{13}C-NMR, 101 MHz, $CDCl_3$)

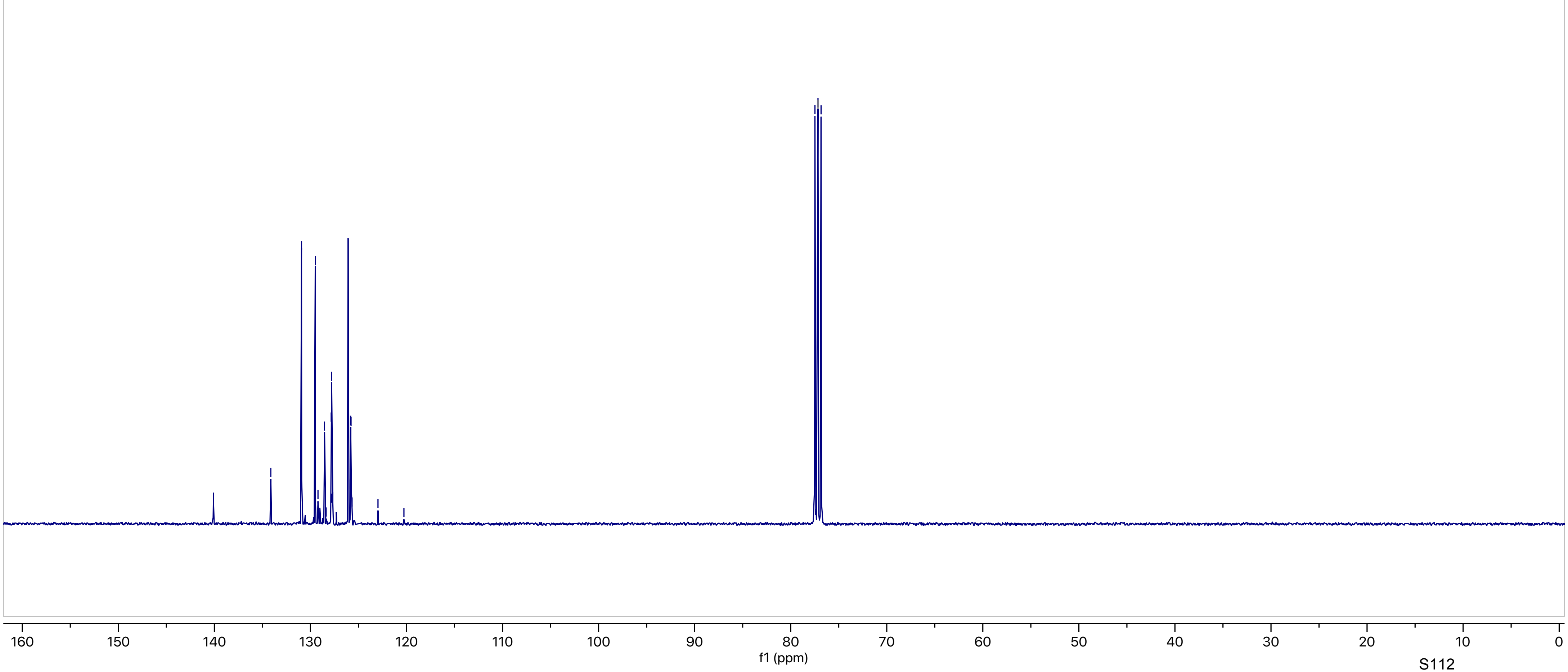

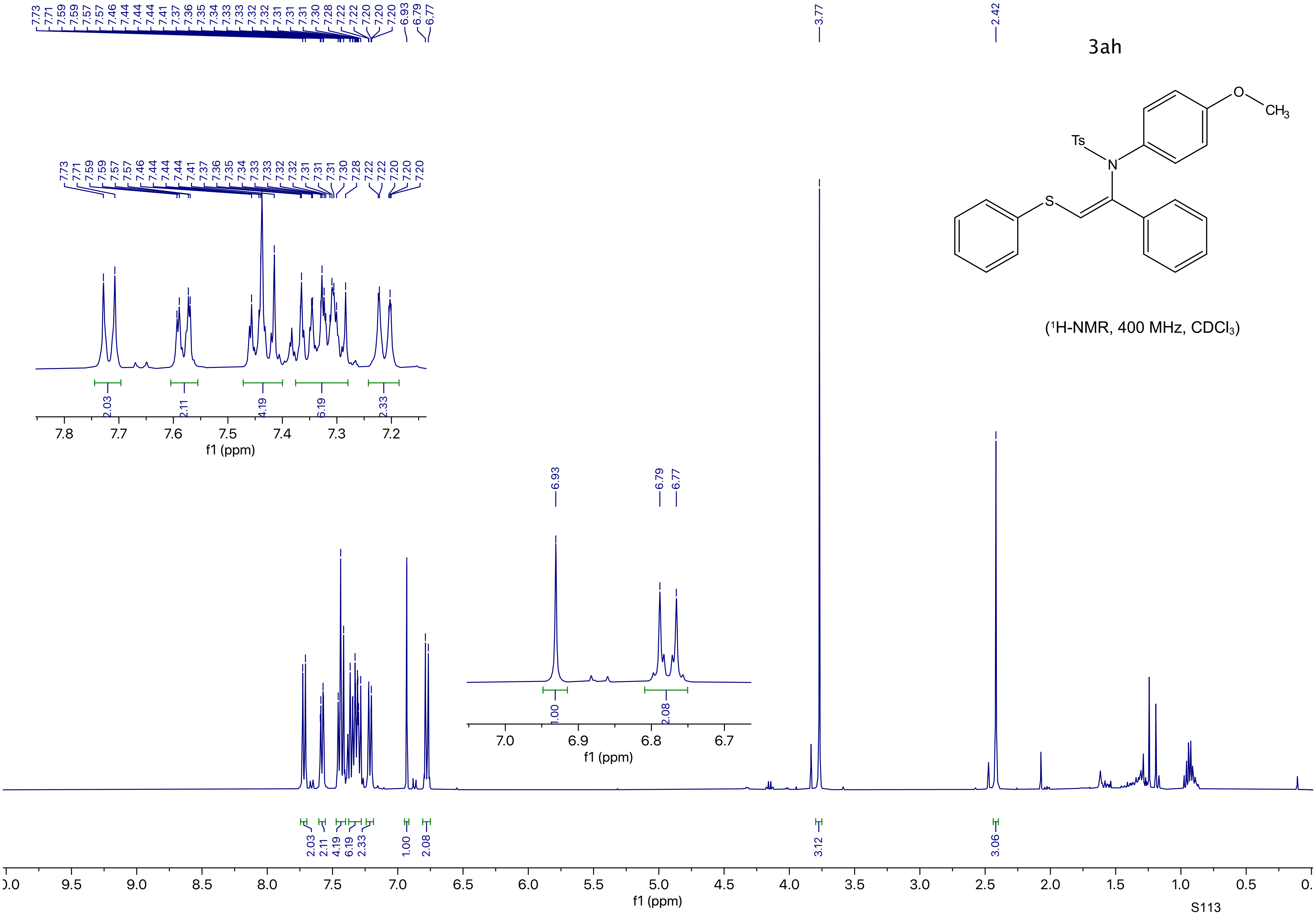
3ah
Ts
N
O
CH3
S
(1H-NMR, 400 MHz, CDCl3)
7.73 7.71 7.59 7.59 7.57 7.57 7.46 7.44 7.44 7.44 7.41 7.37 7.36 7.35 7.34 7.33 7.33 7.32 7.32 7.31 7.31 7.31 7.30 7.28 7.22 7.22 7.20 7.20 7.20 6.93 6.79 6.77
3.77
2.42
2.03 2.11 4.19 6.19 2.33 1.00 2.08 3.12 3.06
f1 (ppm)

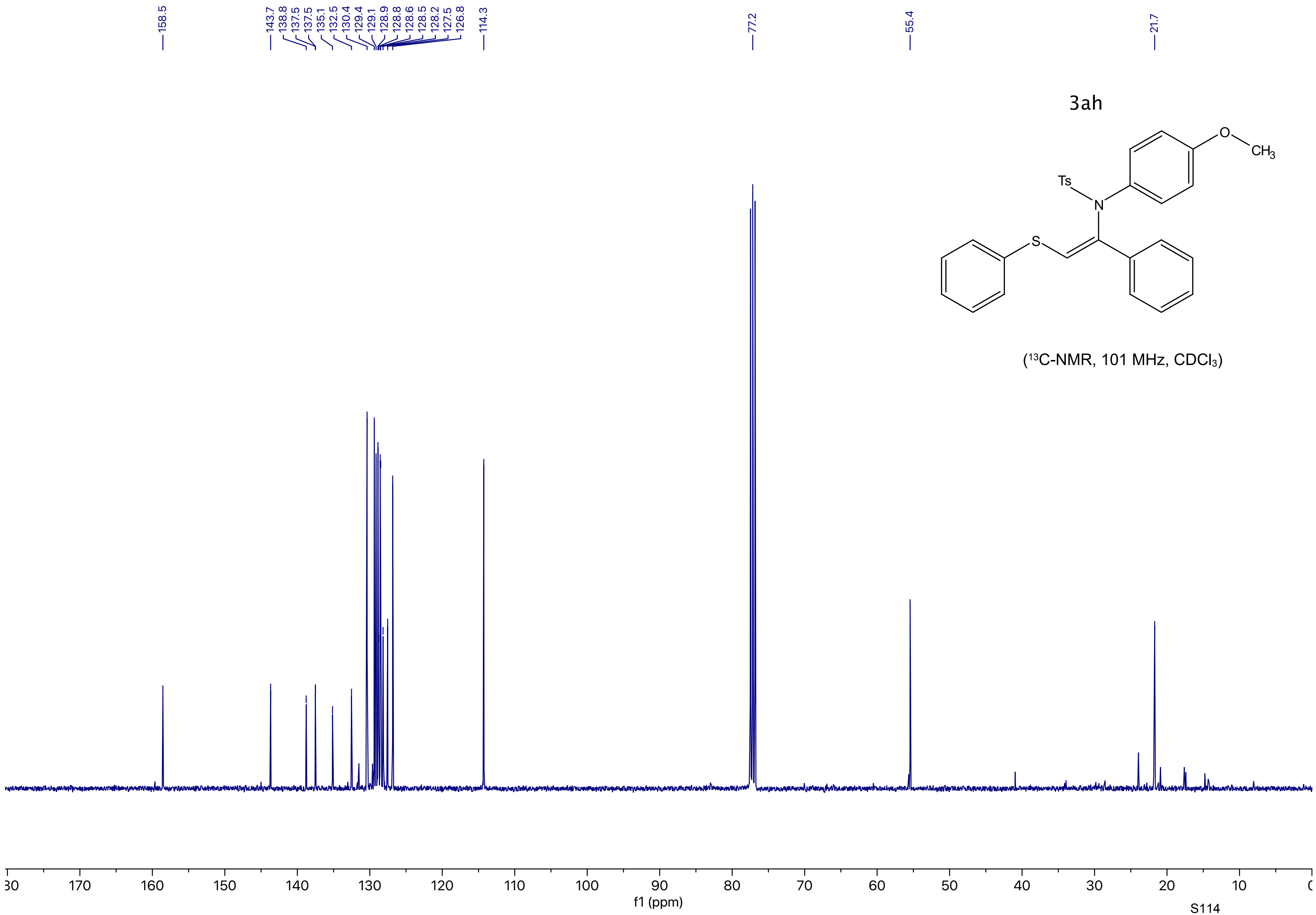
158.5
143.7
138.8
137.5
137.5
135.1
132.5
130.4
129.4
129.1
128.9
128.8
128.6
128.5
128.2
127.5
126.8
114.3
77.2
55.4
21.7
3ah
Ts
N
O
CH3
S
(13C-NMR, 101 MHz, CDCl3)
f1 (ppm)

3ai

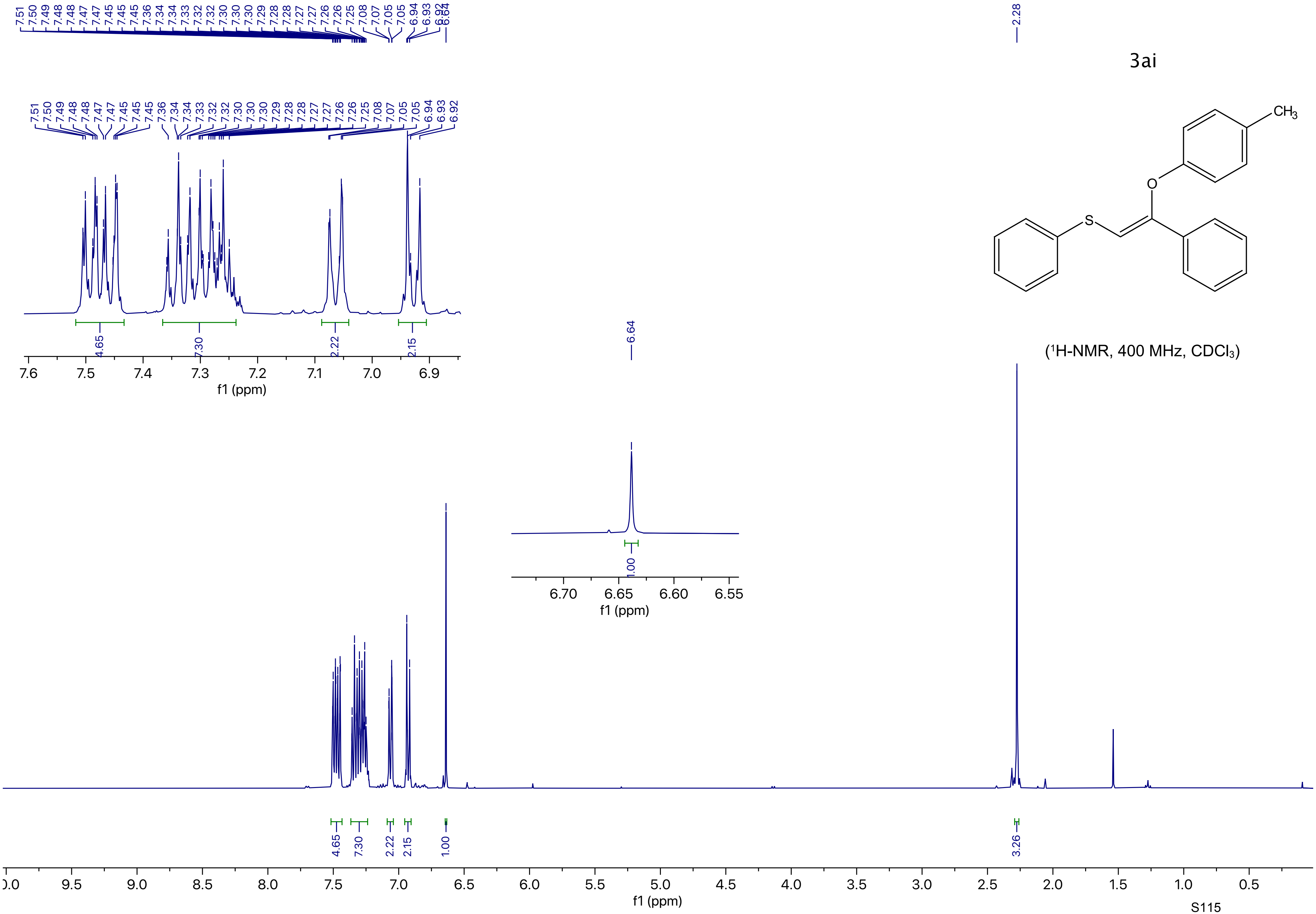

(^{1}H-NMR, 400 MHz, $CDCl_3$)

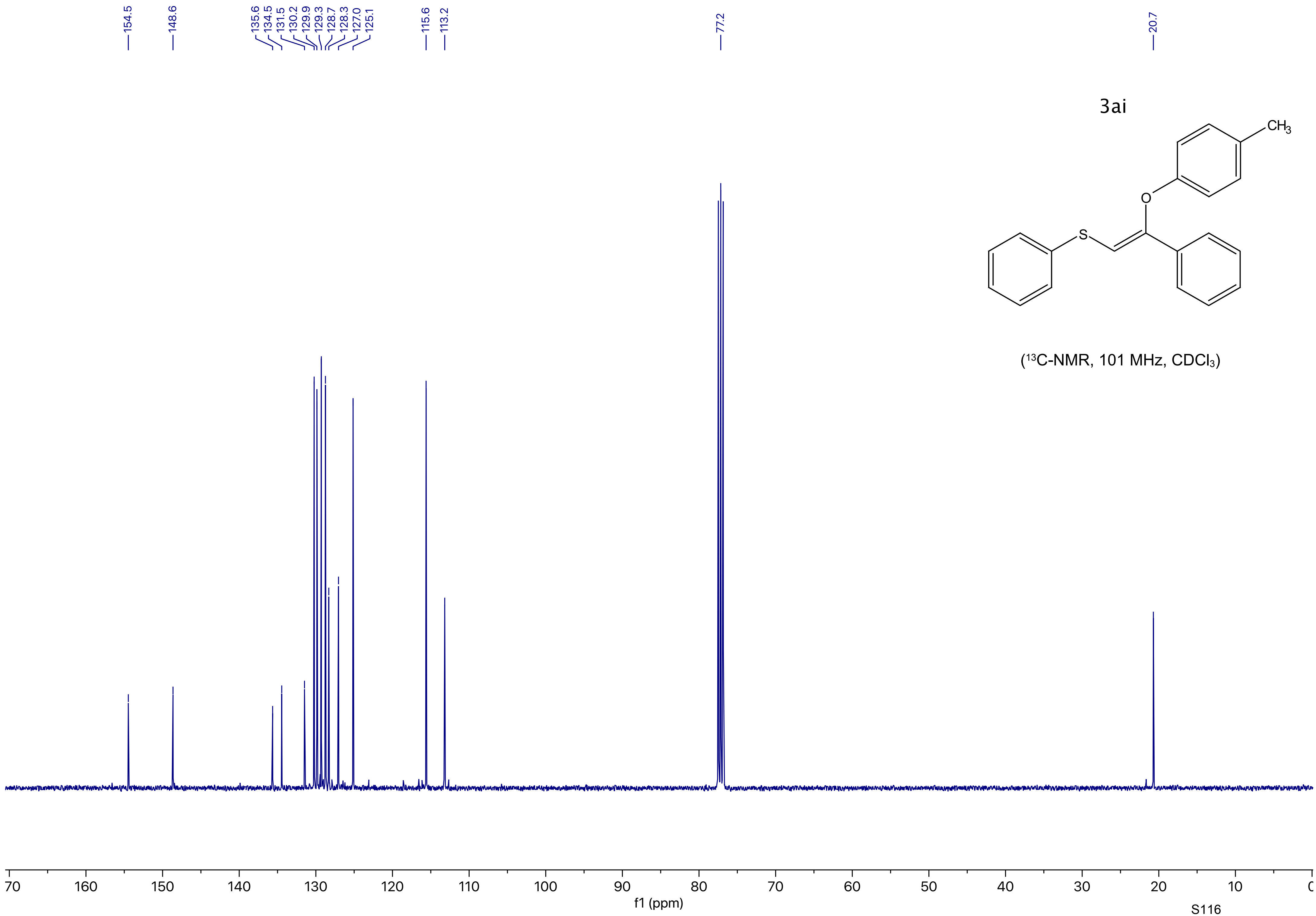

3ai

(^{13}C-NMR, 101 MHz, $CDCl_3$)

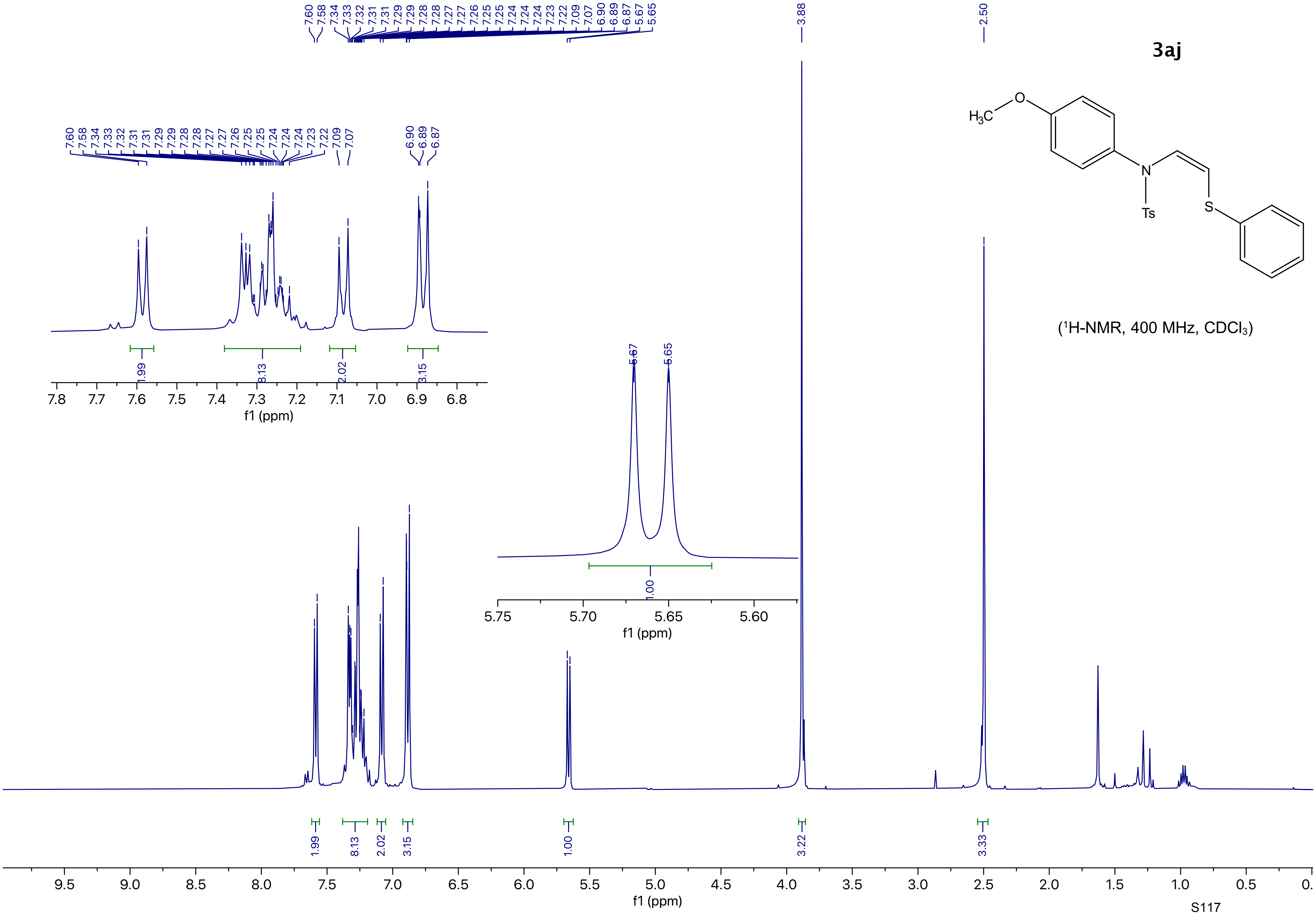

3aj
(¹H-NMR, 400 MHz, CDCl₃)
f1 (ppm)

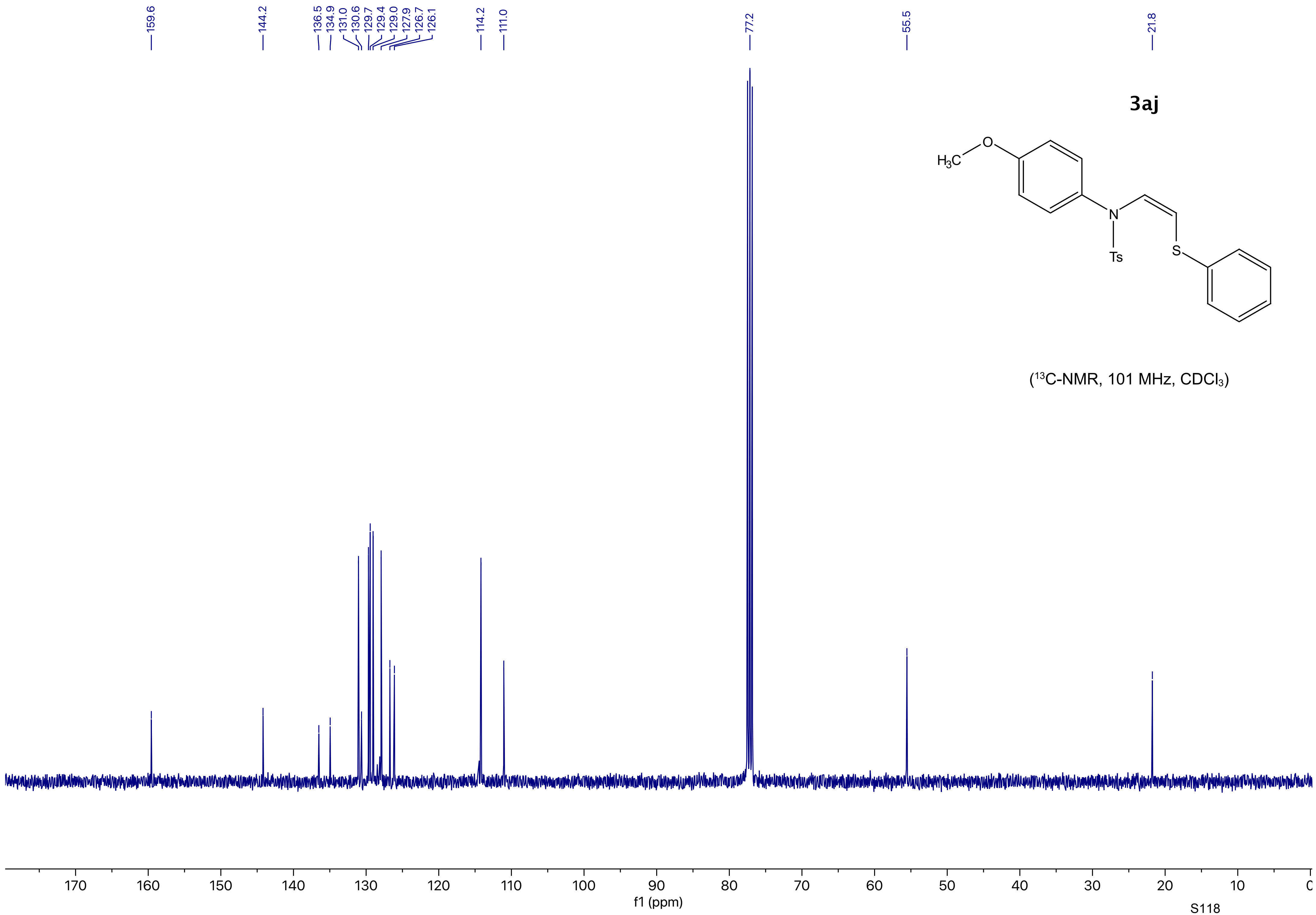

159.6
144.2
136.5
134.9
131.0
130.6
129.7
129.4
129.0
127.9
126.7
126.1
114.2
111.0
77.2
55.5
21.8
3aj
H3C
O
N
Ts
S
(13C-NMR, 101 MHz, CDCl3)
170
160
150
140
130
120
110
100
90
80
70
60
50
40
30
20
10
0
f1 (ppm)

9a

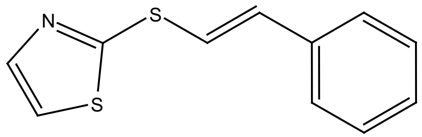

(^{1}H-NMR, 400 MHz, $CDCl_3$)

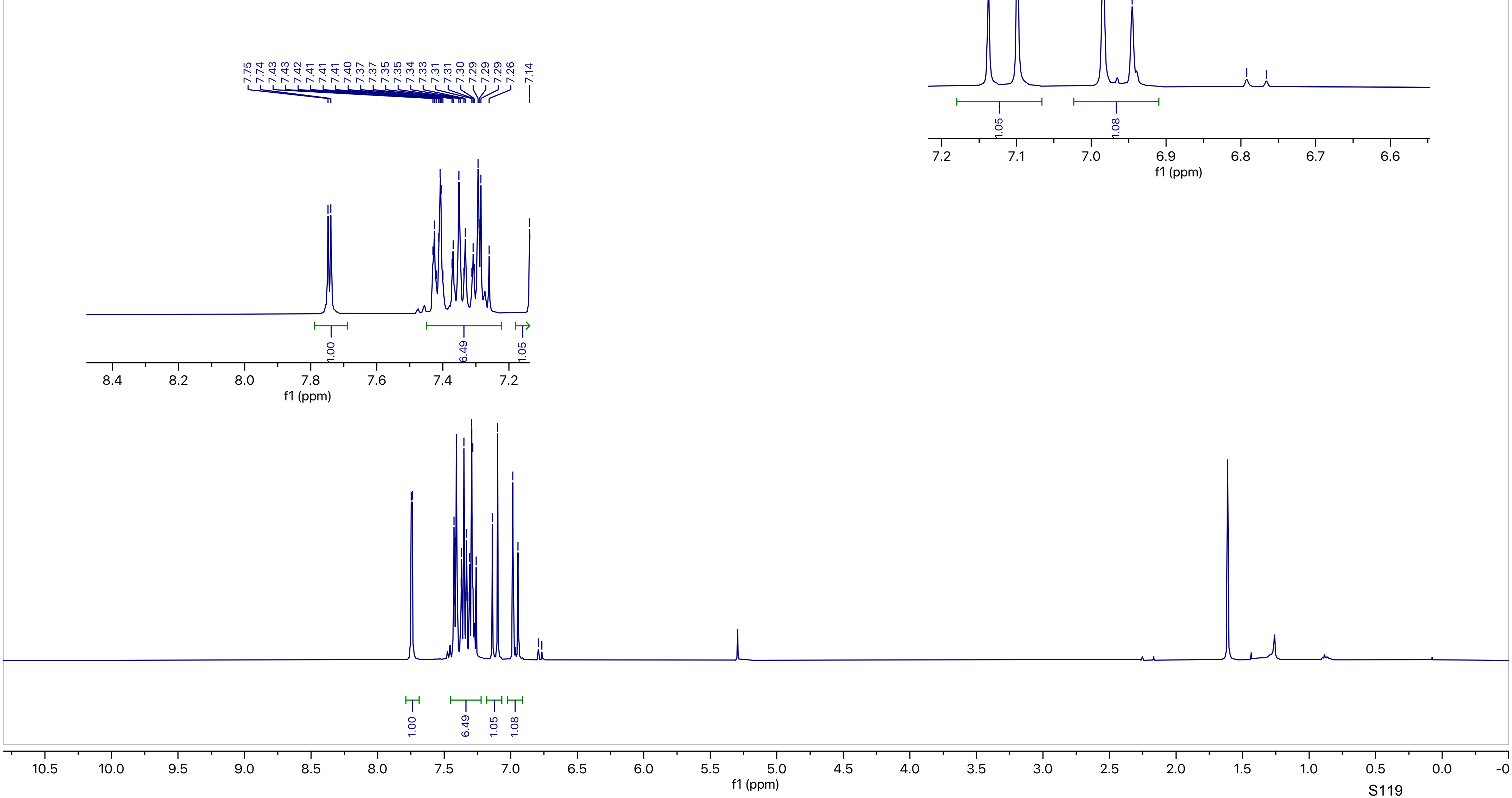

9a

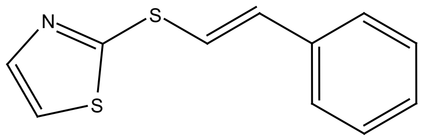

(^{13}C-NMR, 101 MHz, CDCl$_3$)

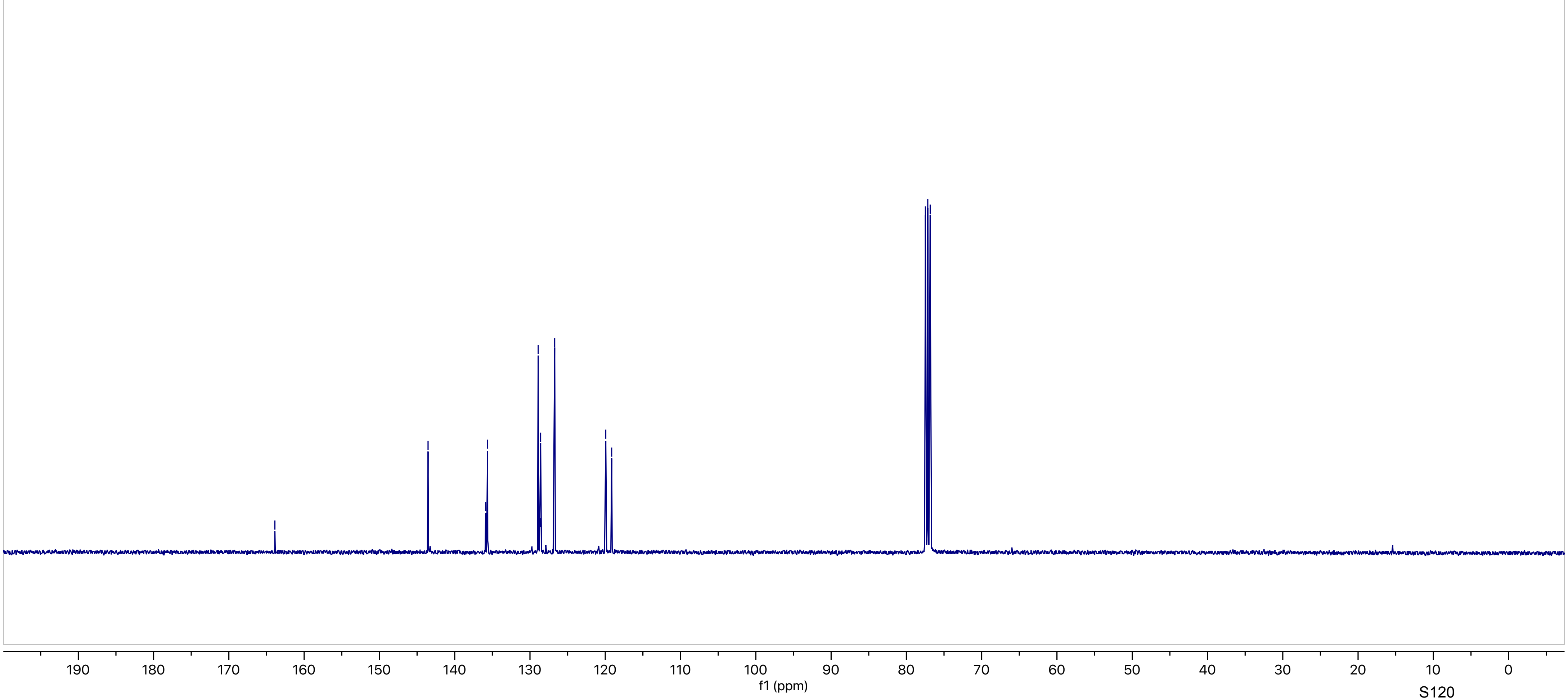

9b

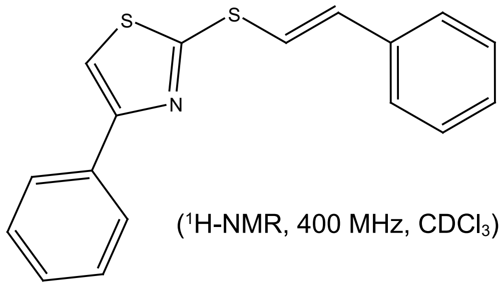

(¹H-NMR, 400 MHz, $CDCl_3$)

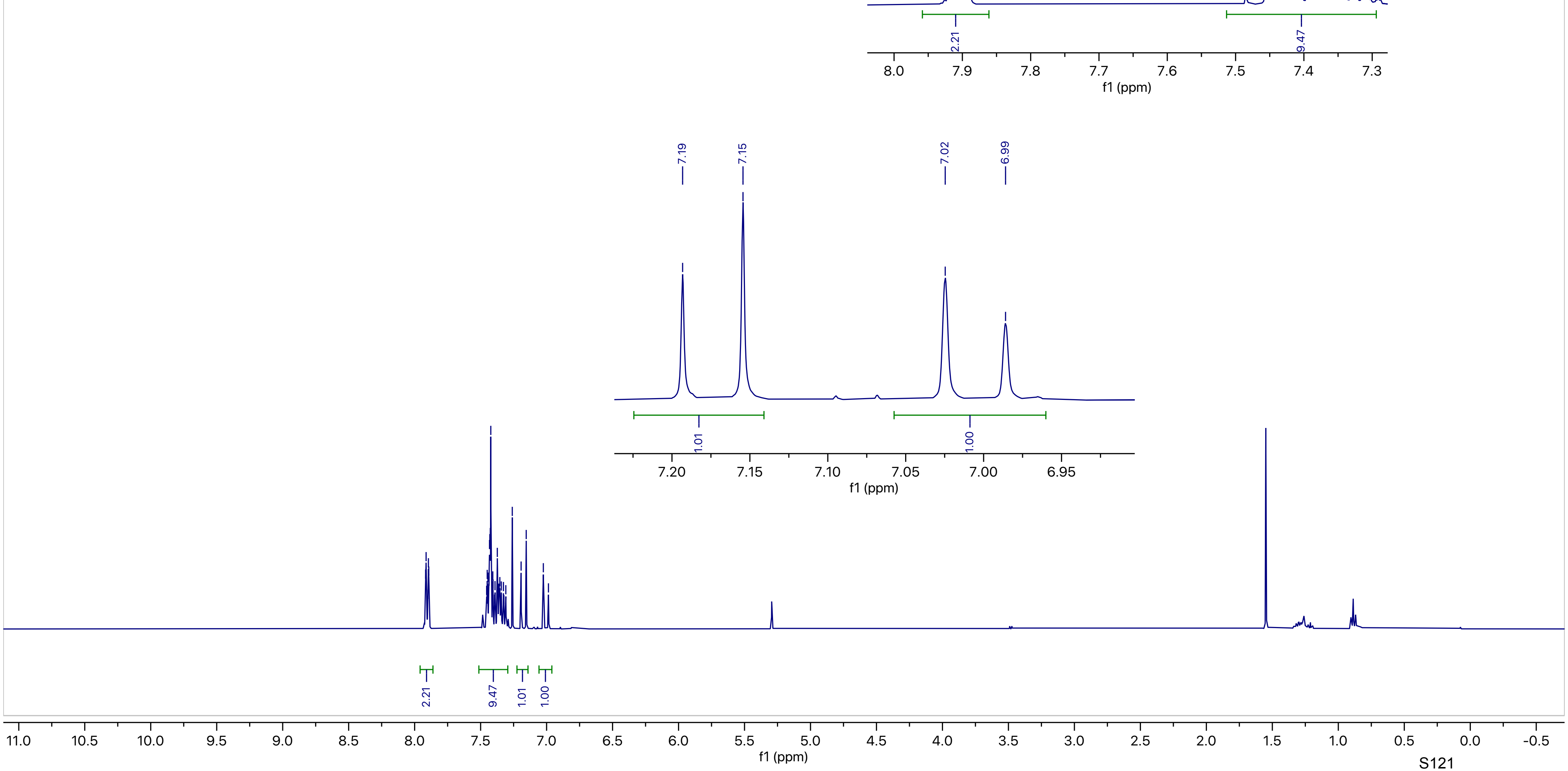

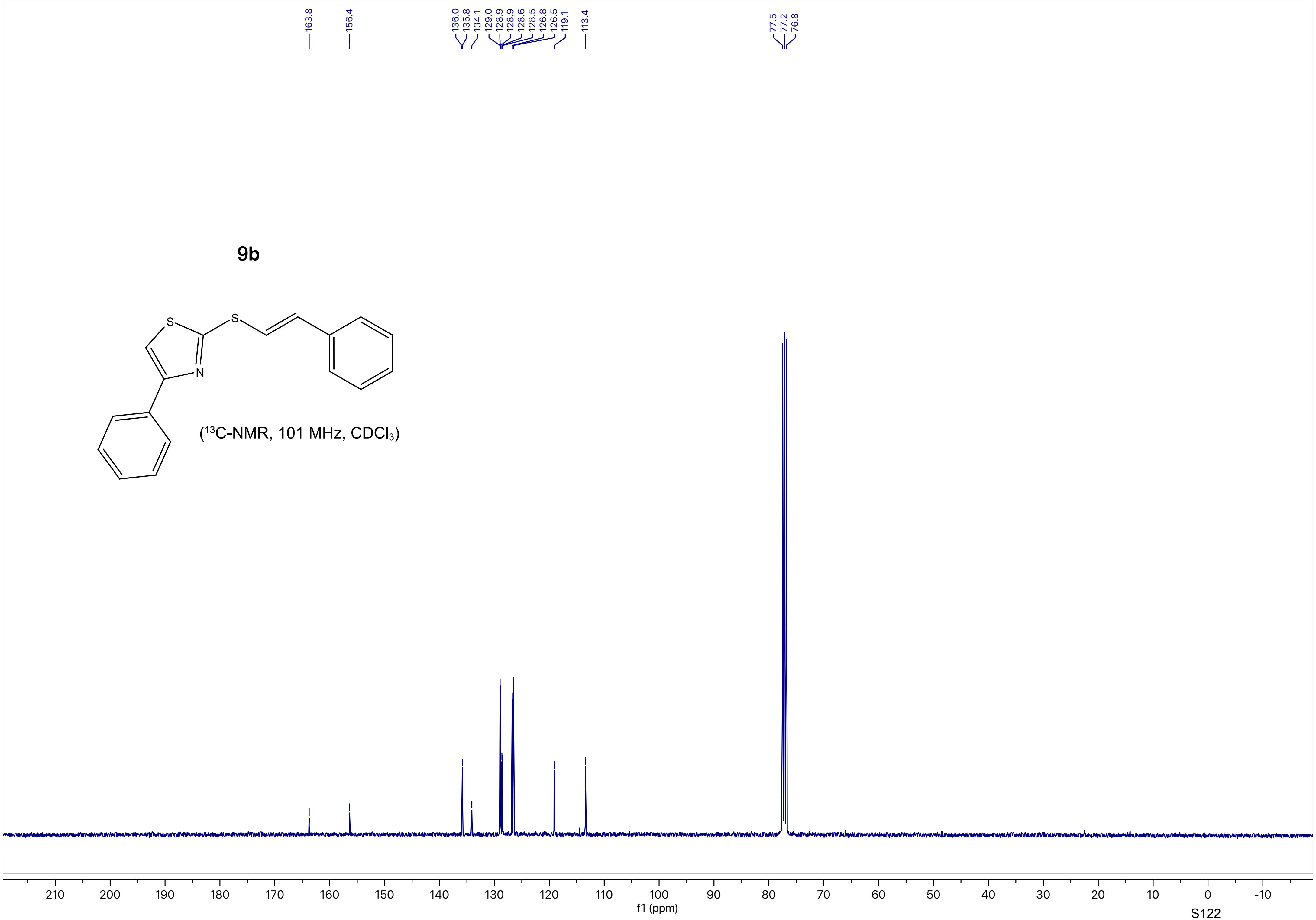
163.8
156.4
136.0
135.8
134.1
129.0
128.9
128.9
128.6
128.5
126.8
126.5
119.1
113.4
77.5
77.2
76.8
9b
S
S
N
(^{13}C-NMR, 101 MHz, $CDCl_3$)
210 200 190 180 170 160 150 140 130 120 110 100 90 80 70 60 50 40 30 20 10 0 -10
f1 (ppm)

9c

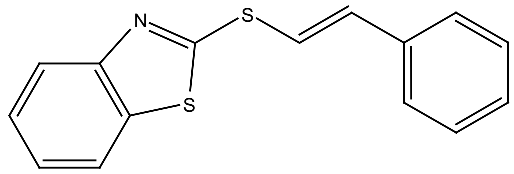

(^{1}H-NMR, 400 MHz, $CDCl_3$)

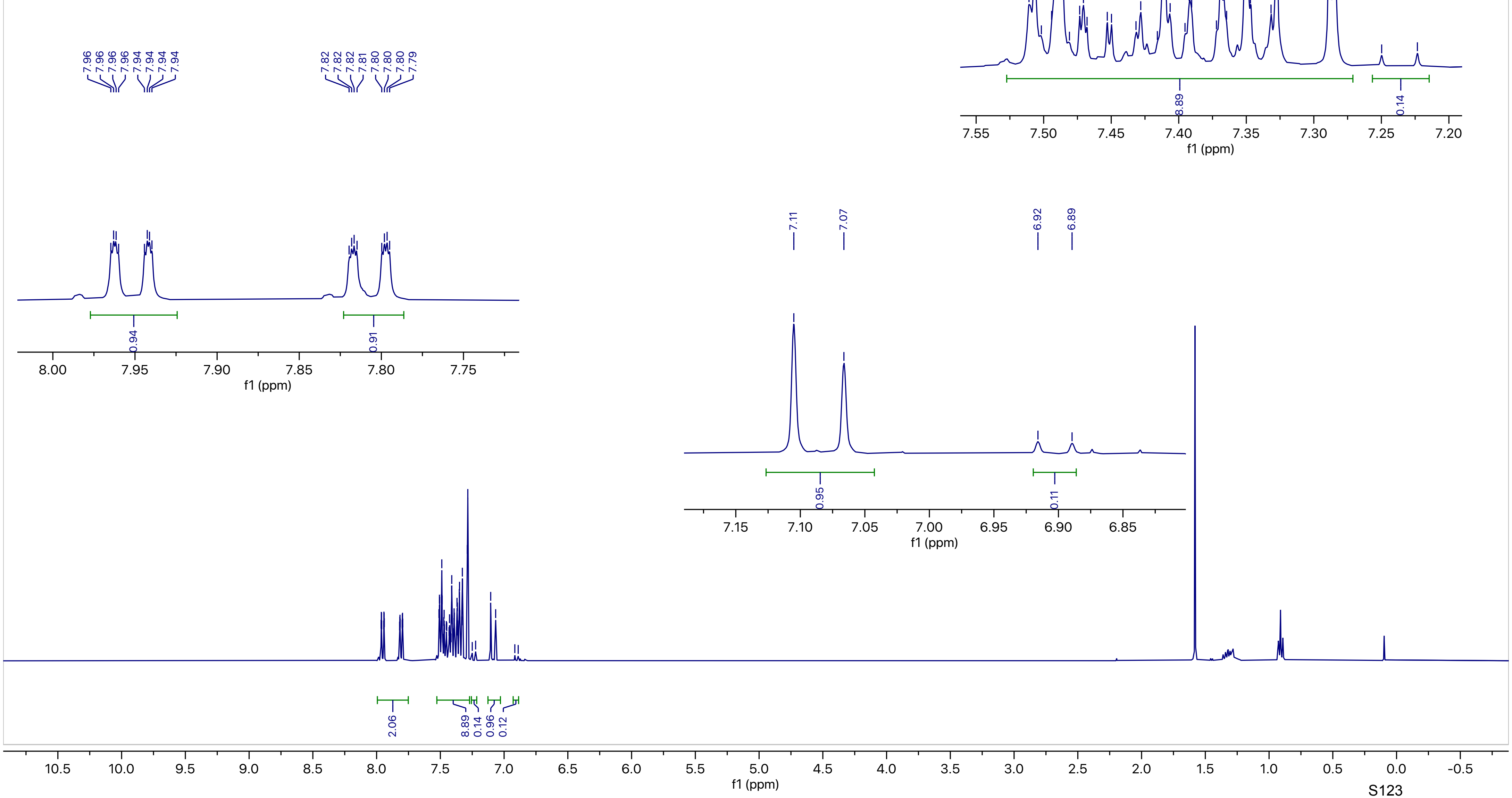

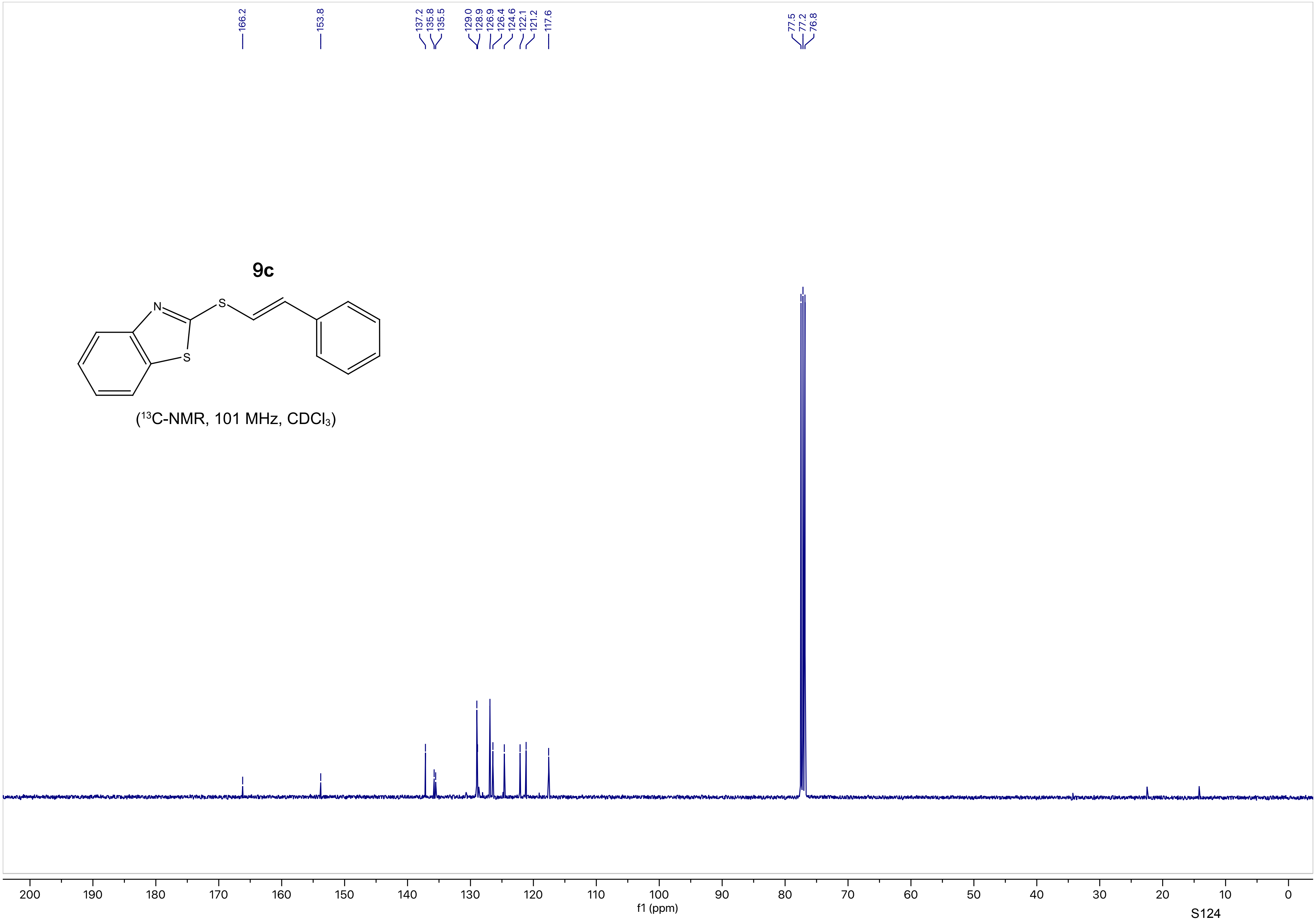
166.2
153.8
137.2
135.8
135.5
129.0
128.9
126.9
126.4
124.6
122.1
121.2
117.6
77.5
77.2
76.8
9c
N
S
S
(¹³C-NMR, 101 MHz, CDCl₃)
200 190 180 170 160 150 140 130 120 110 100 90 80 70 60 50 40 30 20 10 0
f1 (ppm)

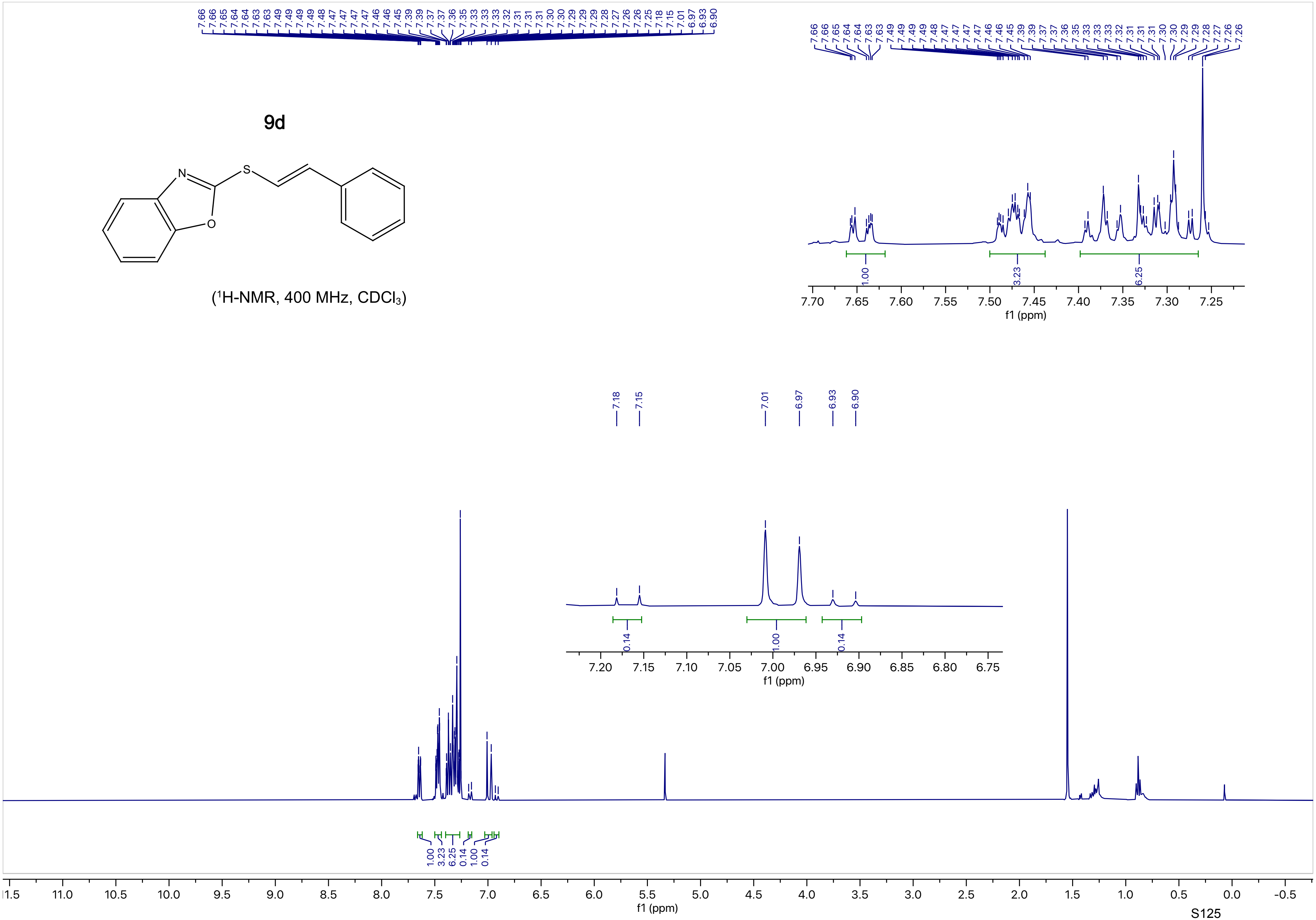
9d
N
S
O
(1H-NMR, 400 MHz, CDCl3)
f1 (ppm)

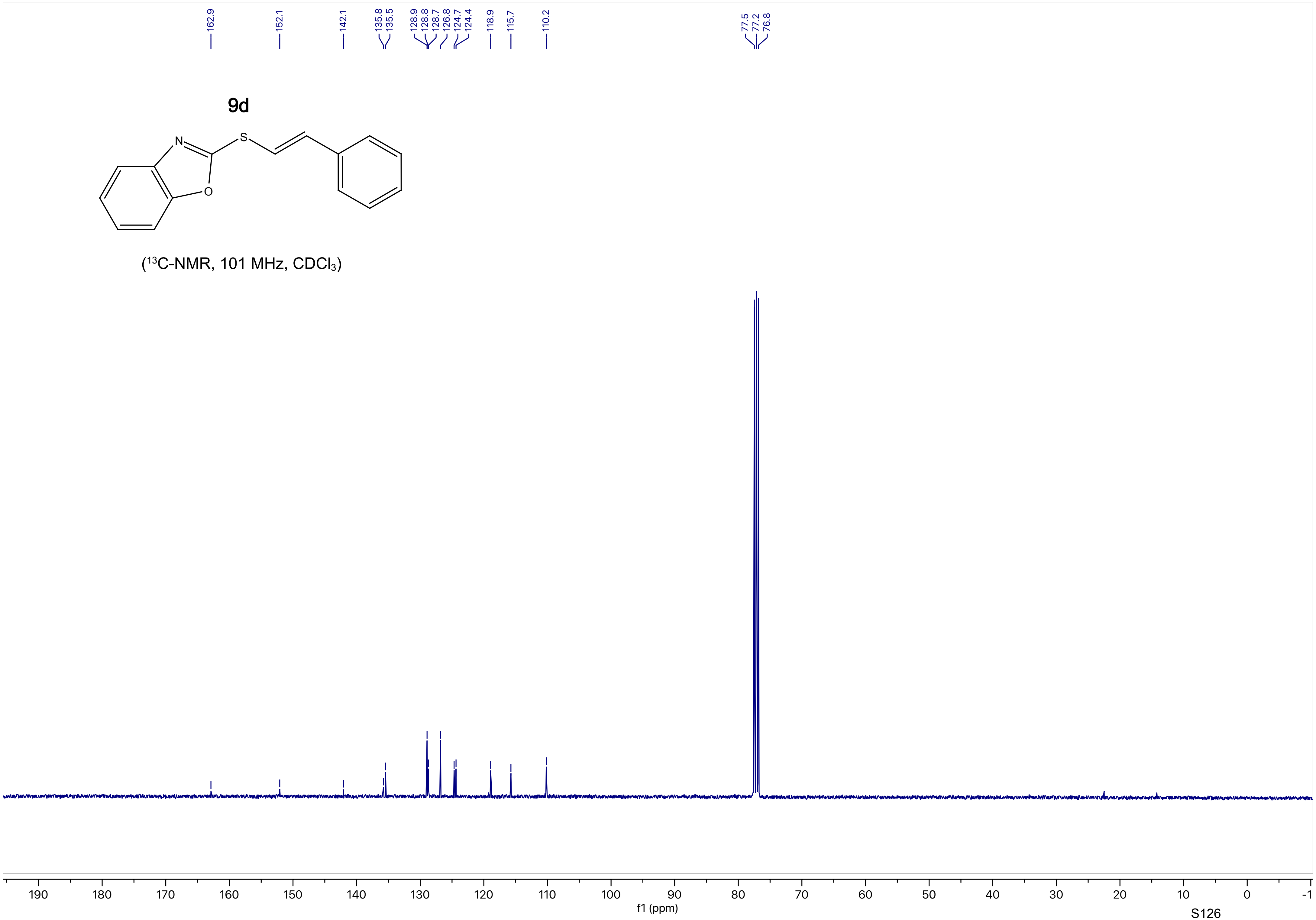
162.9
152.1
142.1
135.8
135.5
128.9
128.8
128.7
126.8
124.7
124.4
118.9
115.7
110.2
77.5
77.2
76.8
9d
N
S
O
(13C-NMR, 101 MHz, CDCl3)
f1 (ppm)
